# Interface-engineered iron single-atom biohybrids for efficient CO_2_-to-bioplastic conversion

**DOI:** 10.1038/s41467-026-76902-9

**Published:** 2026-08-20

**Authors:** Sulin Ni, Dong Xia, Can Chen, Cheng Qiu, Youzhi Li, Bin Yang, Yang Hou, Lecheng Lei, Zhengwei Mao, Zhongjian Li

**Affiliations:** 1 College of Chemical and Biological Engineering, Key Laboratory of Biomass Chemical Engineering of Ministry of Education, Zhejiang University, Hangzhou, China; 2 State Key Laboratory of Green and Efficient Development of Phosphorus Resources, School of Chemical Engineering and Pharmacy, Wuhan Institute of Technology, Wuhan, China; 3 College of Life Science, Zhejiang University, Hangzhou, China; 4 Institute of Zhejiang University – Quzhou, Quzhou, China; 5 MOE Key Laboratory of Macromolecular Synthesis and Functionalization, Department of Polymer Science and Engineering, Zhejiang University, Hangzhou, China

**Keywords:** Electrocatalysis, Environmental biotechnology

## Abstract

A hybrid system combining water electrolysis and H_2_ autotrophic microorganism enables sustainable CO_2_ valorization, but is hindered by low H_2_ bioavailability and sluggish hydrogenase kinetics. Here, we report an interface-engineered inorganic–biological biohybrid, constructed by covalently anchoring iron single-atom catalysts (ISA) onto *Cupriavidus necator* (*C.N*@ISA) via click chemistry. The ISA anchored interface generates a localized H_2_-rich microenvironment, accelerates H_2_ dissociation, while the synergy between ISA and polyethylene glycol-phenylboronate linker stabilizes the inorganic-biological hybrid interface and promotes electron/proton transfer across microbial membrane. These coupled effects boost reduced form of nicotinamide adenine dinucleotide (NADH) regeneration and adenosine triphosphate (ATP) synthesis. In addition, ISA exhibits nanozyme-like activity, scavenging reactive oxygen species to protect cell viability. As a result, *C.N*@ISA achieves CO_2_-to-bioplastic poly-*β*-hydroxybutyrate production of 1058.8 mg L^−1^ with a Faradaic efficiency of 42.0%. Integrating theoretical calculations, electrochemical analysis, and transcriptomics confirms that ISA simultaneously enriches and activates H_2_ while reinforcing intracellular metabolism, offering a generalizable strategy for carbon-negative biomanufacturing.

## Abstract (web summary)

The integration of inorganic–microbial systems offers promise for CO_2_-to-chemical conversion but faces efficiency bottlenecks. Here, the authors report an engineering strategy of anchoring iron single-atom catalysts onto bacterial membranes to achieve enhanced bioplastic production.

## Introduction

The efficient conversion of CO_2_ into value-added fuels and chemicals using renewable energy represents a pivotal technology for the transition towards a sustainable future. Despite substantial advances in electrochemical and thermochemical CO_2_ reduction, these approaches remain limited in both efficiency and product diversity compared with biological CO_2_ fixation^1^. Increasing attention has focused on integrating inorganic catalysis with microbial metabolism, where photo- or electro-driven electrons drive microbial CO_2_ fixation processes^2,3^. Among them, an integrated inorganic-biological system of electricity-driven microbial CO_2_ reduction has emerged as a representative platform, in which electrode-supplied electrons are harnessed by microorganisms to synthesize NADH and ATP, enabling CO_2_ assimilation^4^. Such systems can biosynthesize diverse products, including acetate, malate, and lycopene, underscoring their potential in sustainable biomanufacturing. In particular, H_2_ from water electrolysis has proven an effective and energy-efficient electron shuttle, enhancing NADH and ATP generation while overcoming the limitations of direct extracellular electron transfer^5,6^.

In the integrated inorganic-biological systems mediated by H_2_, efficiency is largely governed by H_2_ mass transfer and bioavailability. However, the intrinsically low solubility of H_2_ (~1 mM) limits its diffusion across the electrode–electrolyte–microorganism interfaces^7,8^. Moreover, hydrogen autotrophic microorganisms, *e.g., Cupriavidus necator* (*C.N*), a model industrial organism for poly-*β*-hydroxybutyrate (PHB) production, suffer from sluggish H_2_ oxidation due to the limited efficiency of hydrogenases, leading to poor CO_2_-to-PHB conversion^9–11^. Unlike strict anaerobes, *C.N* is a facultative “Knallgas” bacterium that requires O_2_ as a terminal electron acceptor for oxidative phosphorylation to meet the high ATP demands of the Calvin–Benson–Bassham (CBB) cycle. This metabolic requirement creates a paradoxical challenge: while O_2_ is essential for energy generation, its electrochemical reduction at the cathode inevitably generates toxic reactive oxygen species (ROS), which suppresses microbial viability and efficiency. These bottlenecks—restricted H_2_ mass transfer, suboptimal microbial H_2_ uptake, and pervasive oxidative stress from electro-generated ROS—result in low overall efficiency. Genetic engineering efforts, such as hydrogenase overexpression or reprogramming metabolic pathways^12,13^, are often limited by complex regulatory networks and off-target effects. Thus, a broadly applicable, genetically agnostic strategy is needed^14–17^.

Nanomaterials with strong H_2_ capture and activation ability offer a promising solution. Transition metal-loaded carbon nanomaterials facilitate H_2_ adsorption through metal atoms' *d* orbitals-H_2_ σ bond interactions, while nitrogen doping modulates the coordination environments and electronic structures to improve H_2_ binding affinity^18^. Single-atom metal catalysts further lower the H_2_ dissociation energy barrier, releasing protons and electrons that enter the microbial electron transport chain (ETC)^19^ and drive ATP synthesis^20^. In addition, transition metal nanomaterials often display intrinsic nanozyme activity^21,22^, scavenging ROS and protecting microorganisms during electrochemical operation. Given the microbial membrane’s pivotal role in H_2_ uptake and electron transfer, engineering microbial membranes with inorganic nanomaterials provides a synergistic pathway to enhance H_2_ transport, dissociation and metabolism. Advances in click chemistry enable stable, well-defined interfaces by anchoring nanomaterials to cis-diol-containing polysaccharides on cell surfaces^23,24^. This strategy offers a robust, site-specific platform for microbial surface modification and sets the stage for integrating single-atom catalysts with microbial cells.

**Fig. 1:**
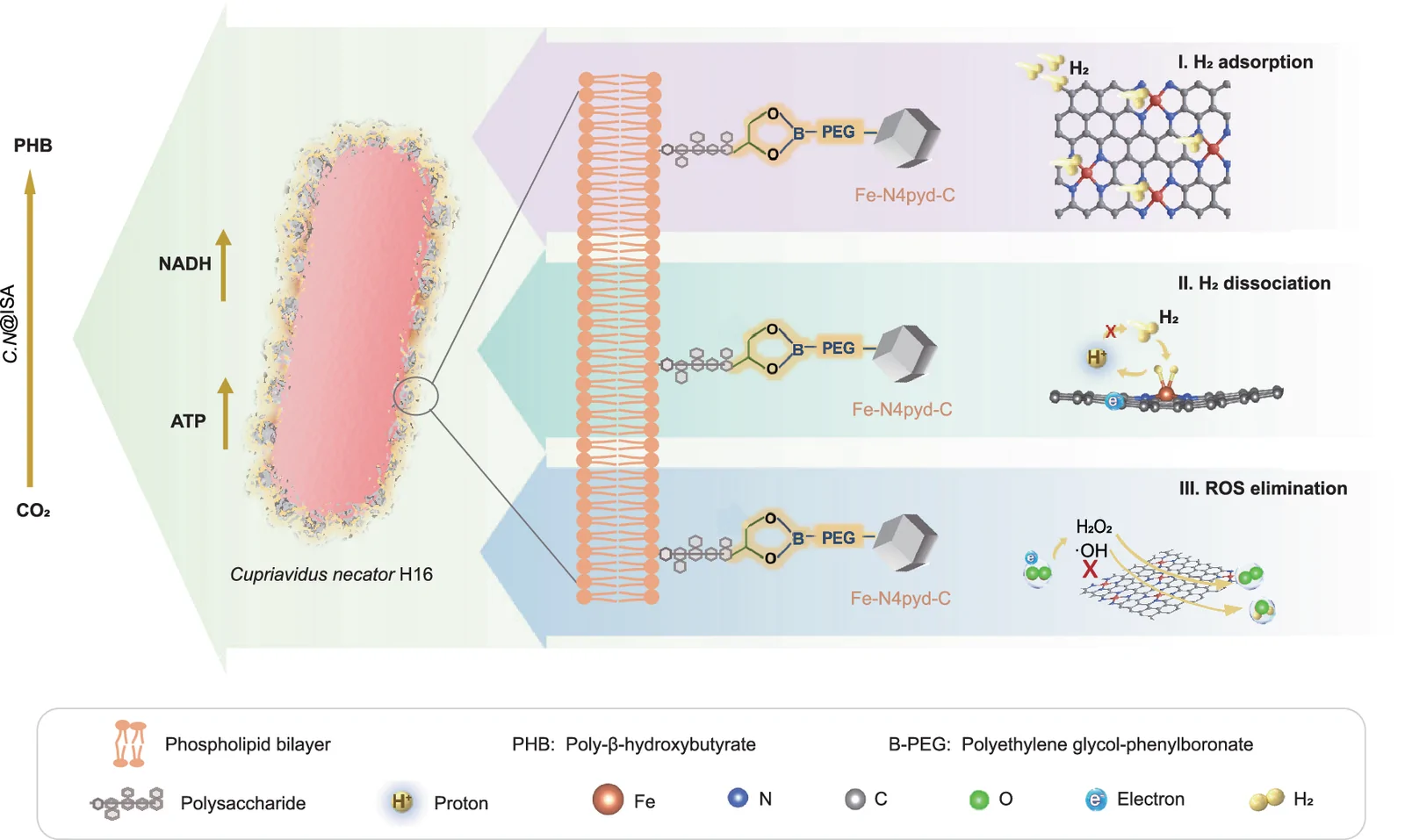
Schematic illustration of the construction of a *C.N*@ISA biohybrid. ISA (Fe-N4pyd-C, isolated Fe atoms are anchored within a nitrogen-doped porous carbon matrix in a fourfold coordination by pyridinic N atoms) are covalently anchored to *C.N* membranes via a B-PEG linker through click chemistry, forming a stable inorganic–biological interface. The anchored ISA acts as an artificial hydrogenase, enriching and dissociating H₂ to accelerate electron generation. Together with enhanced PMF and membrane permeability, this promotes NADH and ATP synthesis and enables efficient CO₂-to-PHB conversion, while ISA also scavenges ROS to protect microbial metabolism.

Herein, we report a microbial interface engineering strategy by covalently anchoring the iron single-atom catalysts (ISA) onto the membrane of *C.N* via click chemistry, successfully constructing *C.N*@ISA biohybrids for PHB microbial electrosynthesis from CO_2_ (Fig. 1). The Fe-N-C sites in ISA enhance H_2_ adsorption and dissociation at the engineered microbial interface; the synergistic effects of ISA and polyethylene glycol-phenylboronate (B-PEG) linker not only promoted extracellular electron transfer inward but also generated a strong proton motive force (PMF); and the nanozyme-like ROS-scavenging activity of the anchored ISA improved cell viability. Collectively, these effects led to efficient CO_2_-to-PHB conversion. Furthermore, the underlying interactions between the anchored Fe-N-C moieties and the bacterial surface were insightfully elucidated by advanced characterization techniques and theoretical calculations. This work establishes a generalizable microbial surface engineering strategy for improving the efficiency of the integrated inorganic-biological system and provides a robust platform for the renewable energy-driven biosynthesis of long-chain carbon products from CO_2_ reduction.

## Results

### Synthesis and characterization of ISA

**Fig. 2:**
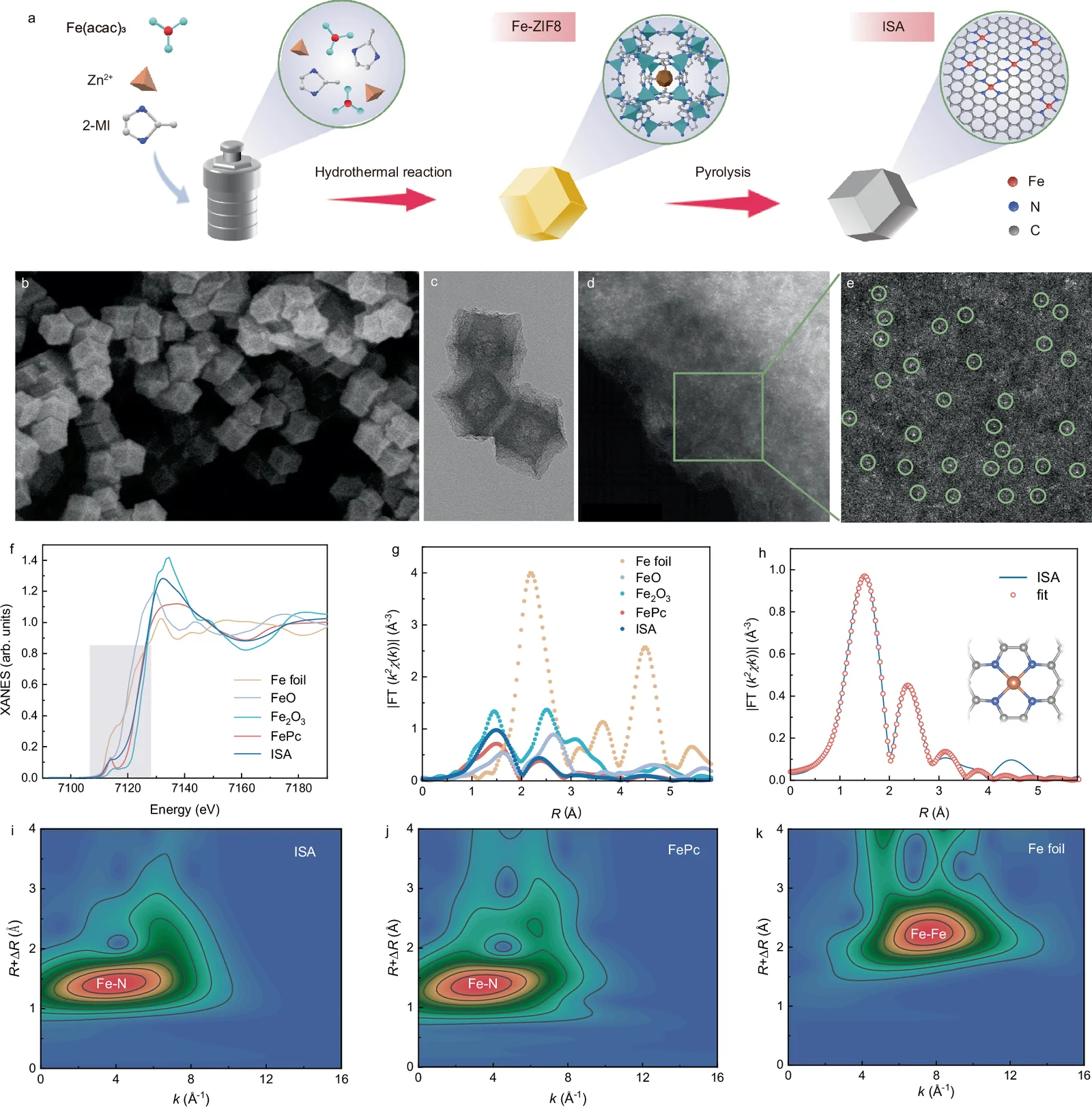
Characterization of the as-synthesized ISA. **a** Schematic illustration of the synthesis process of ISA. Notes: Fe(acac)_3_ represents Fe(III) acetylacetonate. 2-MI represents 2-methylimidazole. SEM (**b**) and TEM (**c**) images of ISA. Spherical aberration-corrected HAADF-STEM (**d**) and enlarged images (**e**) of ISA. **f** XANES spectra of ISA, FePc, Fe foil, Fe_2_O_3_ and FeO (the purple area highlights the near-edge absorption energy). **g** FT of the Fe K-edge of ISA, FePc, Fe foil, Fe_2_O_3_ and FeO. **h** Corresponding EXAFS *R* space fitting curves of ISA. Inset: the ligand structure obtained from fitting, Fe (red), N (blue), and C (gray). WT-EXAFS contour plots of ISA (**i**), FePc (**j**) and Fe foil (**k**). Source data for **f**–**k** are provided as a Source Data file.

To construct such an inorganic-biological biohybrid, ISA was synthesized by pyrolyzing iron-containing ZIF8 (Fig. 2a, Fe-ZIF8, ZIF: zeolitic imidazolate framework). Optimization of pyrolysis temperature indicated 900 °C as optimal, yielding ISA with the highest activity (Unless otherwise specified, ISA refers to the sample obtained by pyrolysis of Fe-ZIF8 at 900 °C). Scanning electron microscopic (SEM) and transmission electron microscopic (TEM) images revealed a regular rhombic dodecahedral structure with an average particle size of 85 ± 6 nm (Fig. 2b, c). Energy dispersive spectroscopy (EDS) confirmed uniform distribution of N and Fe elements (Figs. S1 and 2). X-ray diffraction (XRD) displayed no diffraction peaks of Fe-related crystals, but a broad peak near 26° assigned to the (002) plane of graphitic carbon, suggesting Fe doped into the carbon scaffold (Fig. S3)^25^. High-resolution annular dark-field scanning transmission electron microscopy (HAADF-STEM) further confirmed the atomic dispersion of Fe species (Fig. 2d, e), where the single-atom configuration prevents aggregation and facilitates charge modulation and catalytic activity. Raman spectroscopy indicated a high degree of structural defects (Fig. S4). Nitrogen adsorption-desorption measurements showed a high specific surface area of 1007.93 m^2^ g^−1^ (Fig. S5), supporting strong H₂ adsorption capacity. X-ray photoelectron spectroscopy (XPS) confirmed pyridinic N as the dominant nitrogen species in the N 1*s* spectrum and a peak at ~710 eV in Fe 2*p* spectrum, consistent with Fe-N_x_ structure (Figs. S6–10)^26^.

The coordination state of Fe in ISA was further probed by X-ray absorption near-edge structure (XANES) and extended X-ray absorption fine structure (EXAFS) analyzes. The Fe K-edge XANES spectrum of ISA located between FeO and Fe_2_O_3_, indicating an average valence between Fe^2+^ and Fe^3+^ (Fig. 2f). Fourier transform (FT) of the Fe K-edge EXAFS spectrum (FT-EXAFS) displayed a prominent peak at 1.48 Å attributed to Fe-N coordination scattering, with no Fe-Fe coordination signal (Fig. 2g). Wavelet transform of the Fe K-edge EXAFS (WT-EXAFS) further confirmed Fe-N bonding, showing a single intensity maximum at 4 Å^−1^, similar to iron phthalocyanine (FePc, Figs. 2i–k and S11). Quantitative fitting gave an average Fe-N coordination number of 3.6, close to fourfold coordination, with an average bond length of 2.02 Å. Outer-shell signals suggested the presence of Fe-N-C structure (Figs. 2h and S12, 13, and Table S1). These results confirmed atomic-level dispersion of Fe species in ISA. The physicochemical properties of the materials obtained at different pyrolysis temperatures were evaluated, with particular emphasis on their electrical conductivity, which is a critical factor for constructing an inorganic-biological interface. The sample obtained at a pyrolysis temperature of 900 °C exhibited great electrical conductivity (Fig. S14). Then, the electrochemical stability of ISA was evaluated. After 120 h continuous electrolysis, the morphology of ISA remained unchanged, and negligible Fe leaching was detected, demonstrating the electrochemical stability of the as-synthesized ISA (Fig. S15). Collectively, these characterizations confirm that isolated Fe atoms are anchored in a nitrogen-doped porous carbon matrix, coordinated by four N atoms, providing abundant active sites and robust stability for catalytic applications.

### Construction of the inorganic-biological interfaces via click chemistry

**Fig. 3:**
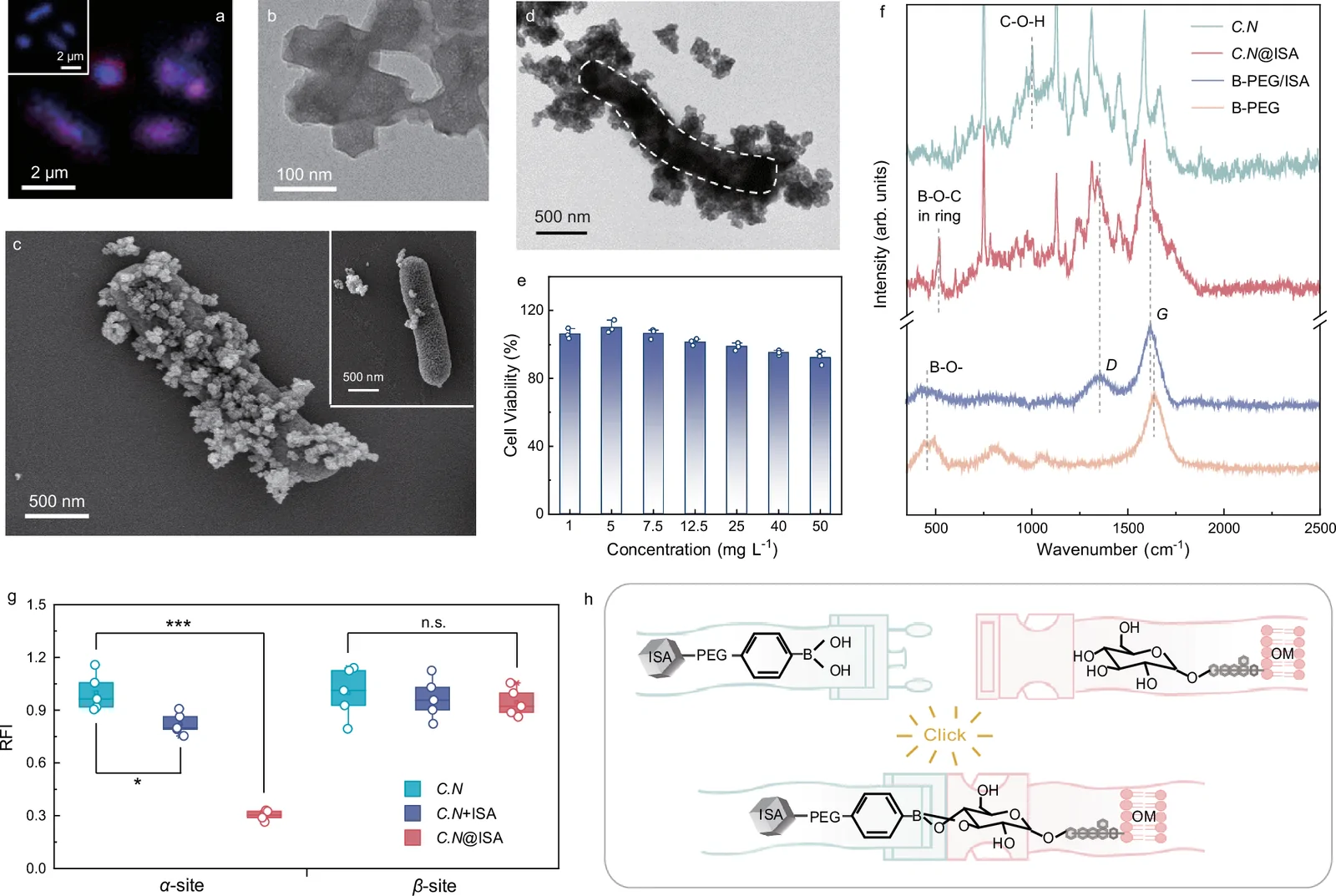
Characterization of the constructed *C.N*@ISA biohybrid. **a** CLSM image of B-PEG modified *C.N*. Inset: CLSM image of *C.N*. **b** TEM image of B-PEG/ISA. **c** SEM image of *C.N*@ISA. Inset: SEM of *C.N* mixed with ISA, in the absence of B-PEG. **d** TEM image of *C.N*@ISA. **e** Cytotoxicity of B-PEG/ISA to *C.N* cells after 24 h incubation. **f** Raman spectra of B-PEG, B-PEG/ISA, *C.N* and *C.N*@ISA. **g** Relative fluorescence intensity (RFI) for *α*-sites and *β*-sites of polysaccharide staining. Statistical significance: n.s., *, **, *** represent *p* > 0.05, *p* < 0.05, *p* < 0.01, *p* < 0.001, respectively. Data are *p*resented as mean ± s. d. from *n* = 5 independent experiments. *p* values were determined by an unpaired two-sided *t*-test. **h** Schematic diagram of interface construction between B-PEG/ISA and *C.N* cell membrane. OM: Outer membrane. PEG: Polyethylene glycol. Source data for e–**g** are provided as a Source Data file.

The key to building a stable inorganic-biological biohybrid is engineering a durable interface between the inorganic nanomaterial and microbial cell. B-PEG was used as a molecular linker to bridge *C.N* and ISA. The long polyethylene glycol chain and benzene ring mitigate reversible hydrolysis of the boronic ester and promote electronic coupling between ISA and the boronic ester. The ability of B-PEG to bind both *C.N* and ISA was examined. Confocal laser scanning microscopy (CLSM) images showed blue emission from *C.N* cells encircled by red fluorescence from B-PEG (Fig. 3a), confirming its strong binding to *C.N*. In parallel, TEM image confirmed successful B-PEG functionalization of ISA (denoted as B-PEG/ISA), as the ISA surface appeared blurred after coating (Fig. 3b). In addition, B-PEG/ISA also exhibited improved colloidal stability (Fig. S16). B-PEG/ISA was subsequently used to modify the *C.N*’s cell membrane, generating a biohybrid (*C.N*@ISA). SEM and TEM imaging (Figs. 3c, d and S17) confirmed successful *C.N*@ISA formation. Cell viability assays showed over 95% viability at B-PEG/ISA concentrations below 25 μg mL^−1^ (Fig. 3e), indicative of high biocompatibility.

The ISA–cell interface in *C.N*@ISA was formed through a click reaction between B-PEG and hydroxyl groups on the cell membrane, as confirmed by Raman spectra and fluorescence labeling. Compared with *C.N*, the Raman spectrum of *C.N*@ISA exhibited additional peaks for cyclic B-O-C bonds, graphitic (*G*) carbon and disordered (*D*) carbon, along with reduced hydroxyl-related peaks (Fig. 3f). Fourier-transform infrared (FTIR) spectroscopy also showed weakened stretching vibrations of hydroxyl groups (3000–3650 cm^−1^, Fig. S18), indicating B-PEG/ISA binding via hydroxyl groups originating from lipids, proteins, and polysaccharides on the cell membrane.

To validate the binding specificity of the *C.N*@ISA interface, a competitive binding assay was conducted using D-fructose as a molecular blocker. Given the high affinity of phenylboronic acid for D-fructose, pre-incubation with this sugar effectively masks the active binding sites and prevents their interaction with cell surface polysaccharides. As illustrated by the SEM characterization (Fig. S19), the D-fructose-treated catalyst failed to anchor onto the bacteria and appeared only as isolated aggregates on the substrate. This successful competitive inhibition definitively confirms that the biohybrid assembly is driven by specific covalent interactions with polysaccharide motifs rather than non-specific physical adsorption or membrane protein interference. Fluorescence staining further revealed *α*-sites of polysaccharides as the major binding site; compared with *C.N*, the *α*-site signal of *C.N*@ISA decreased by 91%, while the *β*-site signal remained unchanged (Figs. 3g and S20). These results confirm that B-PEG/ISA selectively conjugates with hydroxyl groups on the *α*-sites of polysaccharide (e.g., vicinal diol sites on glucose), enabling stable *C.N*@ISA construction for CO_2_ reduction (Fig. 3h).

### Evaluation of ISA-mediated improvement of H_2_ bioavailability and dissociation

**Fig. 4:**
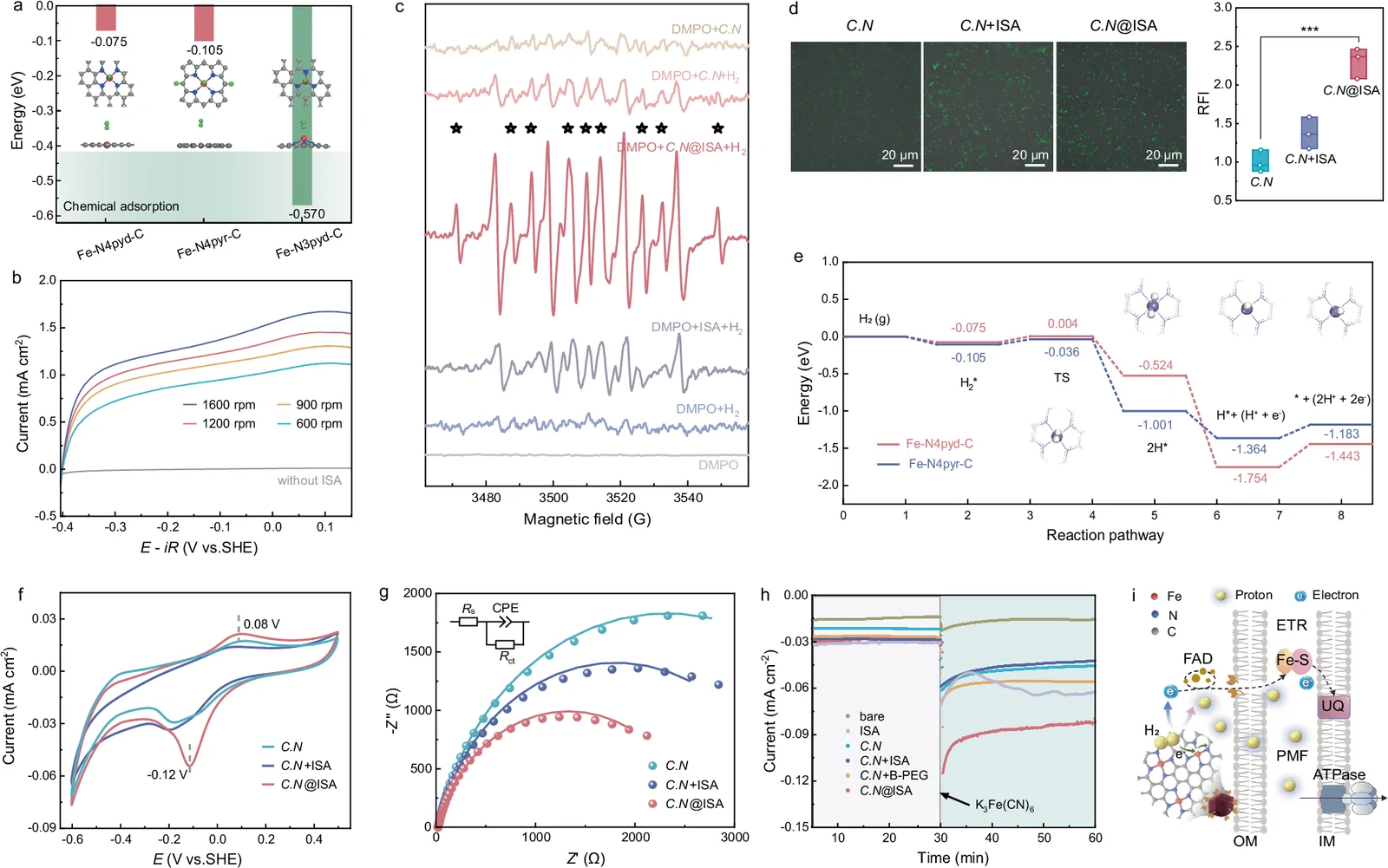
Evaluation of H_2_ activation and electron/proton transfer in *C.N*@ISA biohybrid. **a** Adsorption energy of H_2_ on the modelling configurations of Fe-N4pyd-C, Fe-N4pyr-C, and Fe-N3pyd-C. Inset: Fe (red), N (blue), C (gray), and H (green). **b** Polarization curves of ISA recorded at rotation speeds from 600–1600 rpm. The *x*-axis shows the potential after 85% *iR* correction and the electrode surface area is 0.1256 cm^2^. The solution resistance: 9.85 ± 0.03 Ω. The gray line shows the polarization curve of glassy carbon electrode without ISA at 1600 rpm in a H_2_-saturated electrolyte. Since the final experimental system is a microbial system, the electrolyte used for testing is a neutral 0.1 M PBS (pH = 7.0 ± 0.04). **c** EPR spectra of spin-trapped adducts. Star symbol (★) indicates DMPO-H signal. **d** CLSM images and RFI of cells labeled with pH-sensitive indicators. Data are presented as mean ± s. d. from *n* = 3 biological replicates. **e** Free energy profiles of H_2_ dissociation on Fe-N4pyd-C and Fe-N4pyr-C configurations. The illustration profiling the configurations of intermediates on Fe-N4pyd-C. TS: transition state. H*: adsorbed hydrogen. **f** CV curves of the operating integrated system (72 h). *C.N*@ISA, *C.N* + ISA, *C.N* are all suspended in the electrolyte. **g** Nyquist plots and equivalent circuit fitting plots of the operating integrated system. *C.N*@ISA, *C.N* + ISA, *C.N* are all suspended in the electrolyte. **h** Chronoamperometric responses at −0.2 V vs. SHE, and 0.8 mM K_3_Fe(CN)_6_ added at ~30 min. **i** Schematic illustration of H_2_ dissociation at ISA and subsequent proton/electron transfer pathways. Notes: ETR: Electron transport rate. UQ: Ubiquinone. OM: Outer membrane. IM: Inner membrane. Fe-S: Fe-S clusters. ATPase: ATP synthase. Source data for **a**–**h** are provided as a Source Data file.

To obtain theoretical understanding, density functional theory (DFT) calculations were performed, using Fe-N4pyd-C to represent ISA. The density of states and differential charge density indicated electronic coupling between H_2_ and Fe center (Fig. S21), consistent with strong orbital hybridization between H_2_ and Fe. H_2_ adsorption energy on Fe-N4pyd-C was further compared with Fe-N4pyr-C and Fe-N3pyd-C (Fig. 4a, and Supplementary Data 1). Fe-N3pyd-C exhibited strong adsorption with H_2_ (−0.57 eV), implying “hydrogen poisoning” effects that hinder H_2_ desorption. In contrast, Fe-N4pyd-C and Fe-N4pyr-C showed weaker adsorption energies of −0.075 eV and −0.105 eV, values with a magnitude regarded as optimal (<0.1 eV) for reversible H_2_ storage^27^. These results indicate that ISA can serve as an H_2_ carrier, creating a localized H_2_-rich environment that enhances H_2_ bioavailability, distinct from previous strategies relying on gas-liquid mass transfer or nanoemulsions^28^.

To establish a catalyst-level benchmark for the intrinsic H_2_-dissociation competence of ISA—decoupled from biological complexity—rotating ring-disk electrode (RRDE) measurements were performed on ISA-modified glassy carbon electrodes prior to the in-cell analyzes, with no intent to replicate the suspended biohybrid environment^29^. Relative to the ISA-free control and Ar-saturated electrolyte, ISA-loaded disks displayed a pronounced anodic current under H_2_ saturation (Fig. S22a); the current scaled with rotation rate, and the corresponding Koutecký–Levich plot was linear (Figs. 4b and S22b), confirming a mass-transport-coupled H_2_ oxidation process. The extracted Tafel slope of ~28.6 mV dec⁻¹ (Fig. S23) is reported solely as an electrode-level kinetic descriptor, and no mechanistic claim regarding the in-cell H_2_-activation pathway is drawn from it. Critically, this assay operates under an externally imposed overpotential with ISA immobilized on a solid electrode, whereas in the operating biohybrid ISA is conjugated to the cell envelope without any applied bias; the driving force for H_2_ oxidation is therefore metabolic rather than electrical, as electrons abstracted at ISA under microaerophilic conditions are channeled through the envelope into intracellular NAD(P)⁺ and flavin pools, the respiratory chain, and ultimately into O_2_ reduction and CBB cycle-mediated CO_2_ fixation. This metabolic network functions as a continuous electron/proton sink, rendering the overall process thermodynamically downhill and supplanting the role of an applied potential, such that ISA acts kinetically as a synthetic analog of a [NiFe]-hydrogenase—lowering the activation barrier for H–H cleavage rather than supplying the thermodynamic driving force.

To directly probe H_2_ activation under the biologically relevant conditions, electron paramagnetic resonance (EPR) spectroscopy was conducted using 5,5-dimethyl-1-pyrroline-N-oxide (DMPO) as a spin-trap to capture reactive species generated after H_2_ bubbling in *C.N* or *C.N*@ISA group. As shown in Fig. 4c, both DMPO alone and DMPO + H_2_ exhibited flat baselines, ruling out background signals or contamination. In contrast, a pronounced DMPO-H signal was detected in the *C.N*@ISA group, indicating that the membrane-anchored ISA promotes the formation of reactive intermediates with radical-like (H•-like) characteristics^30^. As a downstream consequence, CLSM imaging of cells with pH-sensitive fluorescent indicators showed stronger fluorescence in *C.N*@ISA, suggesting elevated proton concentration at the cell surface (Fig. 4d)^31^. Based on the convergence of RRDE, EPR, and CLSM data, the ISA-mediated H_2_ activation is proposed as a systemic interfacial flux. While the initial molecular dissociation on the Fe-N_4_ site proceeds via a homolytic-like pathway (H_2_ → 2H^*^), the subsequent efficient coupling with the microbial respiratory chain enables the effective partitioning of these intermediates into protons (H^+^) and electrons (e^-^). This “functional heterolysis” facilitates the maintenance of the PMF and drives intracellular metabolism, thereby functionally mimicking the role of natural hydrogenases within an engineered interface.

The free energy profile provides thermodynamic support for the observed experimental phenomena. DFT calculations were performed using an implicit solvation model (COSMO, *ε* = 78.54), and the data show that the calculated free energy for H_2_ dissociation on Fe-N4pyd-C (0.079 eV, Fig. 4e) is substantially lower than that of natural hydrogenases (~0.52–0.72 eV)^11,32–34^. Beyond H_2_ dissociation free energy, the Fe-N4pyd-C and Fe-N4pyr-C configurations exhibit reorganization energies of −1.230 eV and −0.363 eV, respectively (Fig. 4e and Supplementary Data 1), showing pyridine nitrogen coordination facilitates H* intermediate formation^35^. Moreover, Fe-N4pyd-C displayed a higher energy barrier for H_2_ recombination (1.754 eV) compared with Fe-N4pyr-C (1.364 eV), thereby reducing the likelihood of H* reverting to H_2_^36^.

To distinguish the contributions of interfacial electrochemical effects from bulk H_2_ supply, we compared microbial growth in an electrochemical system (in situ H_2_ electro-generation) versus a direct H_2_ bubbling system under equivalent saturation. The optical density (OD_600_) of the *C.N*@ISA group was significantly higher than that of the *C.N* group in both the electrochemical system and the direct H_2_ bubbling system (Fig. S24). This disparity indicates that the anchored ISA creates a localized H_2_-rich microenvironment that overcomes mass-transport limitations and provides a high-flux supply of active hydrogen species. The internalization of this interface-activated hydrogen was further substantiated by isotopic tracing employing D_2_ gas. The gas chromatography-mass spectrometry (GC-MS) results of PHB extracted from the D_2_-fed *C. necator* exhibited a characteristic shift to higher molecular weights (e.g., around *m/z* 119–120) compared to the H_2_ control (Fig. S25). Such a pronounced mass shift in the polymer fragments confirms that the protons/electrons derived from D_2_ dissociation are successfully integrated into the microbial metabolic pathways for PHB synthesis. These findings provide a kinetic link between the abiotic H_2_ activation at the ISA sites and the biotic CO_2_ fixation process, demonstrating that the ISA-activated hydrogen species are utilized for biological metabolism rather than simple diffusion.

### Evaluation of electron transfer

To evaluate the electron transfer at the inorganic–biological interface, we integrated our experimental results with recent advances in *C. necator* biohybrids. As the inorganic–biological interface is constructed via B-PEG/ISA, we first examined the surface charge properties of B-PEG/ISA to evaluate its physicochemical compatibility with the bacterial envelope. Zeta potential measurements showed that B-PEG/ISA carried a near-neutral positive charge of +2.2 mV (Fig. S26), indicating minimal electrostatic repulsion with the negatively charged cell surface and thereby facilitating close interfacial contact^37^. Importantly, the stable association of ISA with the cell membrane is governed by covalent bonding between the phenylboronic acid moieties of B-PEG and cis-diol groups on extracellular polysaccharides rather than by electrostatic interactions. Although polysaccharides and B-PEG are non-conductive, they do not obstruct energy flux in our system because electron transfer may be mediated by redox-active species present at the interface, which can readily diffuse through these molecular matrices. To further examine this hypothesis, we performed cyclic voltammetry (CV) measurements on the bacterial suspension from the reactor and observed a pair of redox signals with a reduction peak at −0.12 V and an oxidation peak at 0.08 V (vs. SHE), which can be tentatively assigned to flavin-related redox couples (Fig. 4f). High-performance liquid chromatography (HPLC) analysis further detected a peak with a retention time matching that of the flavin adenine dinucleotide (FAD). The slight retention time shift observed in the biohybrid samples relative to the standard is attributed to matrix effects from the complex minimal medium, which contains high salt concentrations and cellular byproducts. Standard-addition experiments confirmed the assignment of this signal to FAD (Fig. S27). These results indicate that extracellular flavin species are present in the biohybrid system and may function as diffusible redox mediators^38,39^. Electrochemical impedance spectroscopy (EIS) results reflect mediator-modulated interfacial Faradaic processes in the biohybrid electrolyte. (Fig. 4g, and Table S2). The fitted charge-transfer resistance decreased in the order *C.N* > *C.N* + ISA > *C.N*@ISA, consistent with the increasing abundance of extracellular flavin species detected by CV and HPLC. Since the impedance spectra show a pronounced charge-transfer semicircle without a clear Warburg diffusion feature, the observed decrease in *R*_ct_ is more reasonably attributed to the contribution of soluble redox mediators and interfacial electrochemical processes, rather than hydrogen evolution reaction kinetics or direct electron transfer from the electrode to the cells. We also note that secondary factors, including bacterial coverage and extracellular polymeric substances on the electrode surface, may contribute to the measured impedance response. This aligns with recent studies showing that *C. necator* utilizes secreted flavins as diffusible redox mediators to facilitate interfacial electron uptake from inorganic materials^38,39^. To further evaluate the electron transfer kinetics at the bio-inorganic interface, K_3_Fe(CN)_6_ was employed as a highly reversible redox probe in chronoamperometry (Fig. 4h). While electrodes modified with ISA or *C.N* alone exhibited moderate current increases compared to the bare carbon cloth, the *C.N*@ISA hybrid displayed a significantly enhanced current response. This synergistic effect suggests that the current is not merely an additive result of individual components but originates from accelerated mediator-assisted electron uptake by the cells. By establishing a covalent microscopic interface, the system facilitates efficient charge transfer between the ISA catalyst and the microbial respiratory chain. Comparison of growth under in situ electrochemical H_2_ generation versus direct H_2_/O_2_ bubbling (Fig. S24) indicates that ISA-mediated interfacial H_2_ activation constitutes the dominant electron-input route. Consequently, the combination of ISA-facilitated H_2_ oxidation and FAD-mediated electron transfer increases the electron flux toward microbial metabolism^40,41^, driving efficient CO_2_-to-PHB conversion (Fig. 4i).

### Assessment of strengthened microbial metabolism

**Fig. 5:**
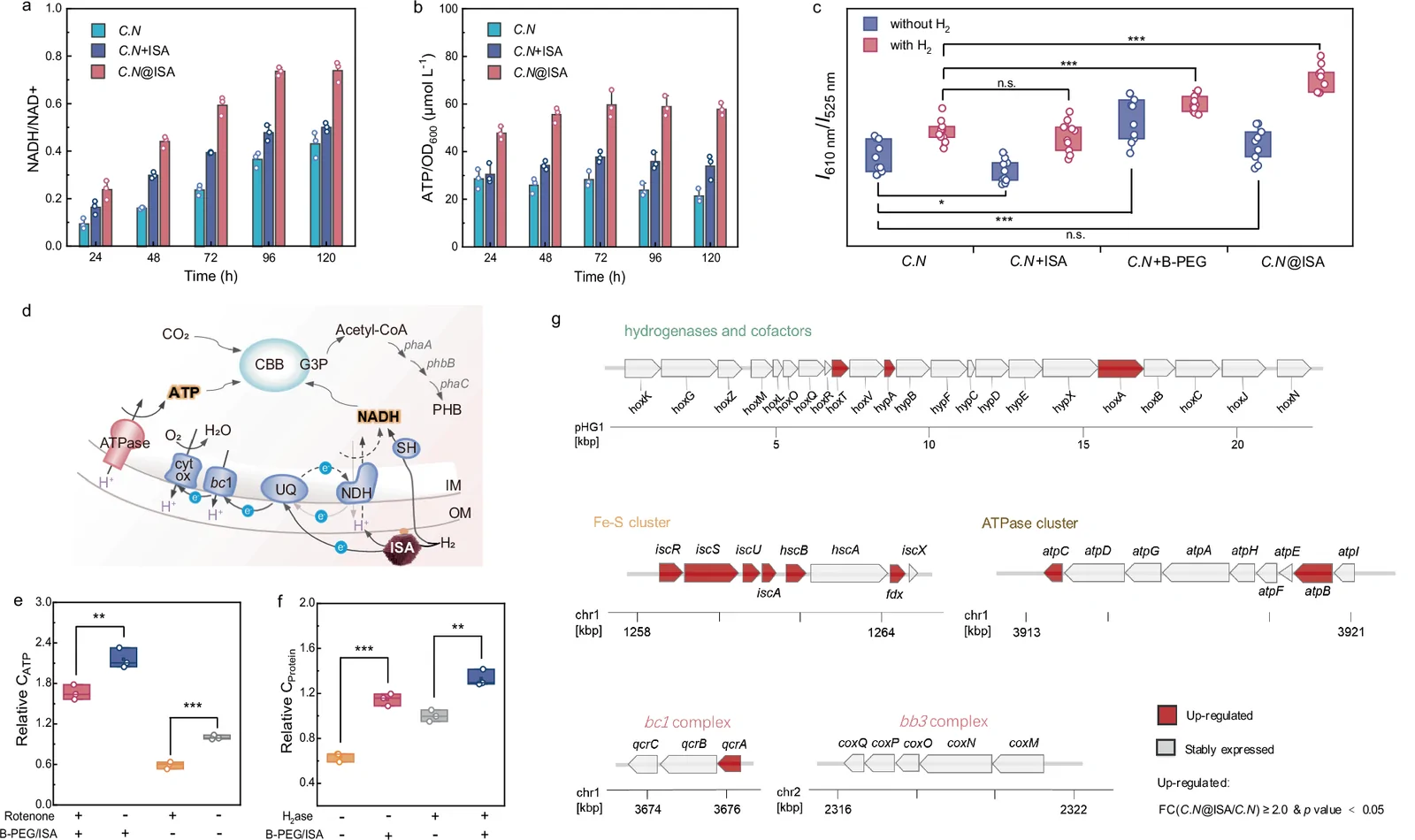
ISA-enhanced bioenergetics and metabolic regulation in *C.N*@ISA biohybrid. **a** NADH/NAD+ ratio in cells with different treatments. **b** ATP content in cells with different treatments. Data are presented as mean ± s. d. from *n* = 3 independent experiments. **c** Membrane potential measured by the ratio of aggregate and monomer fluorescence intensities. Data are presented as mean ± s. d. from *n* = 10 independent experiments. **d** Schematic illustration of electron transport chain, oxidative phosphorylation, CBB cycle, and PHB production in *C. necator*. G3P: Glyceraldehyde 3-phosphate. Acetyl-CoA: Acetyl coenzyme A. **e** ATP content of cells upon addition of rotenone. **f** Total protein content of *C.N*ΔMBHΔSH. Data are presented as mean ± s. d. from *n* = 3 independent experiments. H_2_ase: Hydrogenase. **g** Expression of gene clusters of interest. Hydrogenases and cofactors clusters contain genes for membrane-bound hydrogenase (*hoxKGZ*), accessory proteins (*hoxMLOQRTV*), metallo-cofactor assembly (*hypABCDEFX*) and H_2_ sensor+trans regulator (*hoxABCJN*). The electron transport chain complexes clusters contain genes for *bc1* complex (*qcrABC*) and *bb3* complex (*coxMNOPQ*). The Fe-S cluster genes (*iscARSUX, hscAB, fdx*). The ATPase cluster contains genes for ATP synthase (*atpABCDEFGHI*). FC: Fold change. Source data for **a**–**c** and **e**–**g** are provided as a Source Data file.

After exploring that B-PEG/ISA promotes H_2_ enrichment and dissociation at the inorganic–microbial interface, we next evaluated whether this enhanced H_2_ availability would stimulate microbial metabolism. As such, the impact of ISA on intracellular NADH/NAD+ and ATP levels was accordingly evaluated. Compared with *C.N*, *C.N*@ISA exhibited a higher NADH/NAD+ ratio as expected (Fig. 5a). Meanwhile, intracellular ATP levels were also largely elevated in *C.N*@ISA (Fig. 5b). These results suggest that ISA anchored at the microbial membrane provides additional reducing power and energy, likely functioning analogously to a membrane-bound hydrogenase that enriches H_2_ around the cell and, in turn, accelerates its oxidation.

Furthermore, we evaluated the effect of B-PEG/ISA on the membrane potential, a component of PMF. Under H_2_-free conditions, *C.N* + B-PEG group exhibited a higher fluorescence ratio compared to both the *C.N* and the *C.N* + ISA groups, supporting the hypothesis that B-PEG can alter cell membrane permeability (Fig. 5c). Once upon H_2_ supplementation, compared to the *C.N* group, the *C.N* + ISA group did not show a discernable increase in fluorescence ratio. Distinctly, the *C.N*@ISA group displayed a marked increase, indicating that ISA anchored to the cell membrane via B-PEG promoted membrane potential hyperpolarization^42^. As a consequence, the elevated membrane potential and the higher proton gradient (Fig. 4d) collectively enhanced the PMF, thereby favoring NADH regeneration and ATP synthesis (Fig. 5d)^43,44^. The fidelity of the membrane potential measurements was further validated by protonophore carbonyl cyanide 3-chlorophenylhydrazone (CCCP) induced depolarization of the membrane potential, validating the fluorescent reporters for monitoring membrane potential (Fig. S29). Rotenone, an inhibitor of NADH dehydrogenase^45^, decreased ATP levels in both *C.N*@ISA and *C.N* groups. But the decline was less pronounced in *C.N*@ISA than that in *C.N*, suggesting that the membrane-bound ISA provides an alternative electron entry point independent of NADH dehydrogenase (Fig. 5e). To support this conclusion, we utilized a hydrogenase-deficient mutant strain (*C.N* ΔMBHΔSH). Under chemoautotrophic conditions, while the growth of the mutant was severely impaired, the covalent anchoring of ISA largely restored its metabolic activity (Fig. 5f). This suggests that ISA functionally mimics membrane-bound hydrogenases, facilitating the entry of reducing equivalents into the cell. Furthermore, the ATP levels and membrane potential of *C.N*@ISA under electrochemical and chemoautotrophic conditions were compared^46^. As shown in Fig. S28, both ATP levels and membrane potential in *C.N*@ISA are higher under electrochemical conditions than those observed under chemoautotrophic conditions, revealing an additional contribution from the electrochemical environment. This enhancement likely arises from modulation of the local proton electrochemical gradient and subtle influences on membrane-associated redox processes in the near-electrode region, while the ISA interface plays a primary role in promoting interfacial H_2_ activation and local proton supply.

To evaluate the biocompatibility of ISA, the ISA’s effect on hydrogen peroxide decomposition and hydroxyl radical scavenging was further investigated^47^. Results indicated that ISA enabled rapid catalysis of hydrogen peroxide decomposition, achieving a marked removal rate of 80% within 30 min (Fig. S30) along with suppressed hydroxyl radical signal in EPR spectra (Fig. S31). These findings indicate that ISA exhibits nanozyme-like activity and can reduce exogenous ROS.

Finally, transcriptomic analysis revealed alterations in microbial metabolism. Compared with *C.N*, *C.N*@ISA showed significant upregulation (fold change ≥2 and *p* value < 0.05) of genes encoding hydrogenase accessory proteins (*hoxAT*, *hypA*), Fe-S cluster biosynthesis (*iscARSU*, *hscB*, *fdx*), and ETC complexes (*qcrA*)^48^. Genes encoding ATP synthase subunits (*atpBC*) and NADH dehydrogenase (*nuo*) were also upregulated (Figs. 5g, S32 and 33). These results collectively infer that *C.N*@ISA utilizing B-PEG/ISA modification facilitates electron transfer from the extracellular H_2_ to the interior of the cell, thus strengthening PMF, in turn promoting ATP synthesis to render the efficient CO₂-to-PHB bioconversion^41^.

### The boosted CO_2_ to PHB conversion with *C.N*@ISA

**Fig. 6:**
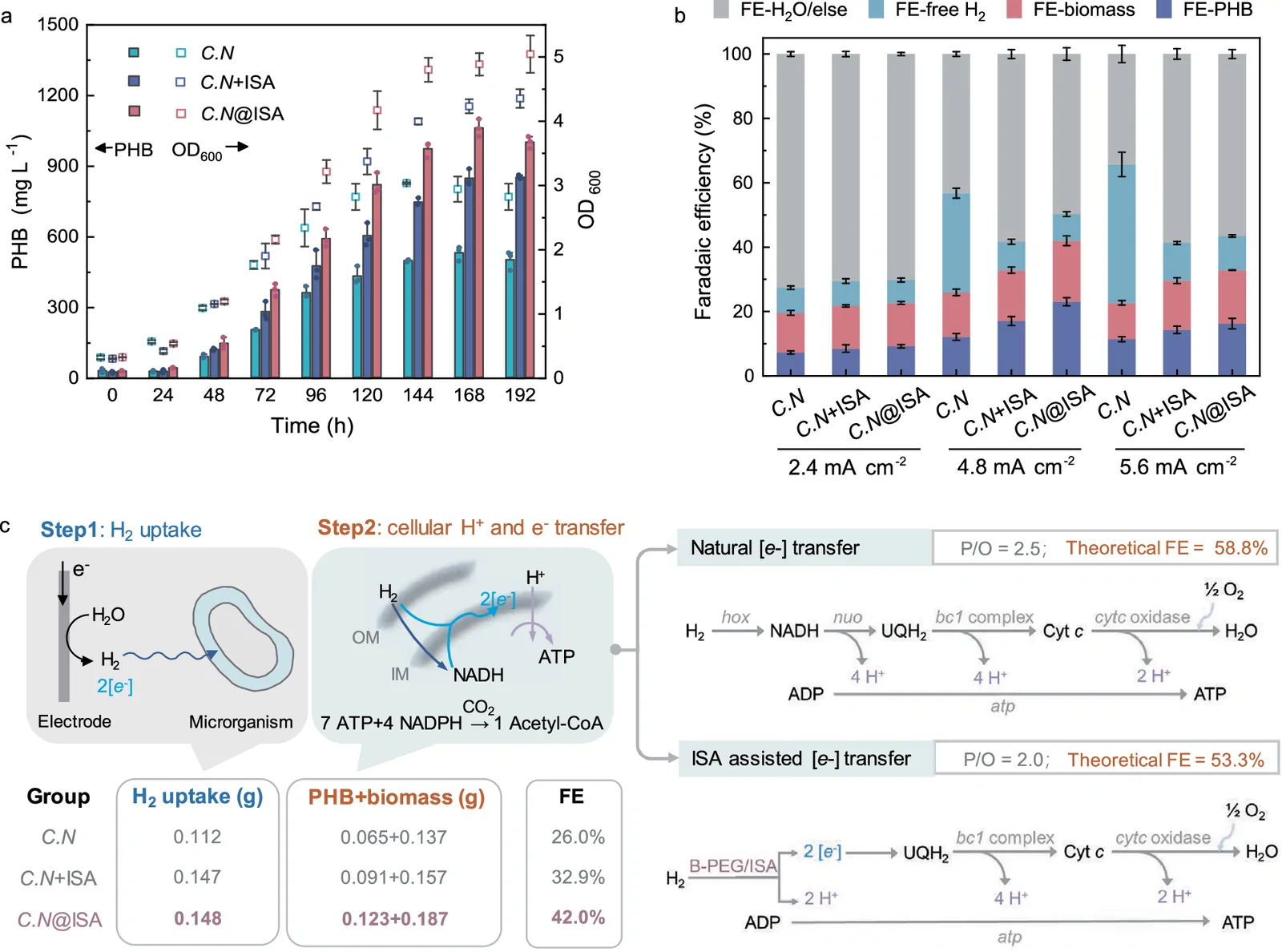
Performance evaluation and electron utilization in *C.N*@ISA biohybrid. **a** Time-dependent OD_600_ values and PHB yields of varied integrated inorganic-biological systems. **b** Distribution of cathodic electron utilization toward H_2_ release, residual H_2_, biomass formation, and PHB synthesis at different current densities. Data are presented as mean ± s. d. and error bars represent the standard deviation of three independent experiments. **c** Analysis of electron allocation pathway. NADPH: Reduced form of nicotinamide adenine dinucleotide phosphate. Source data for a and b are provided as a Source Data file.

Proof-of-concept experiments were conducted using the biohybrid in a membrane-free single-chamber configuration (Fig. S34), a design choice specifically intended to minimize ohmic losses while accommodating the facultative nature of *C.N*, which requires anodic O_2_ for oxidative phosphorylation to meet the high ATP demands of the CBB cycle. We strategically employed a stainless-steel mesh cathode to ensure electrochemical stability and eliminate cytotoxicity. In this H_2_-centric architecture, the ISA primarily serves as an interfacial catalytic hub that enriches and activates H_2_ to address the primary metabolic bottleneck. Simultaneously, the anchored ISA provides an auxiliary protective layer by scavenging secondary ROS (Fig. S35), maintaining cellular viability (Fig. S36), thereby ensuring that the energy-efficient single-chamber design is not compromised by interfacial oxidative stress. The *C.N* group produced an OD_600_ of 2.94 and a PHB yield of 529 mg L^−1^. Upon adding ISA (*C.N* + ISA), OD_600_ increased by 44% to 4.23, with PHB yield improving by 60% to 849 mg L^−1^, confirming the role of ISA in enhancing CO_2_-to-PHB conversion. Notably, *C.N*@ISA further raised OD_600_ to 4.87 and doubled PHB production to 1058.8 mg L^−1^ (Fig. 6a). This PHB yield was obtained using 12.5 mg L^−1^ B-PEG/ISA (Fig. S37). The PHB-to-dry cell weight (PHB/DCW) ratio in *C.N*@ISA approached 46% (Fig. S38), higher than those reported in several previous studies (Table S3). The energy conversion efficiency (ECE) of *C.N*@ISA reached 12.29%, higher than *C.N* + ISA (9.76%) and *C.N* (7.67%) (Fig. S39).

The operational robustness of the *C.N*@ISA biohybrid was further evaluated through an extended fed-batch cultivation lasting 360 h (15 days). Upon nutrient replenishment at the 168-h mark, the hybrid system maintained stable OD_600_ values with no discernible decrease for an additional 192 h, indicating that the bacteria retained relatively stable metabolic activity throughout the prolonged operation (Fig. S40). To verify the durability of the bio-inorganic interface across successive cellular generations, morphological characterization was conducted via SEM after the prolonged operation (Fig. S40). The results revealed that the surfaces of daughter cells remained persistently decorated with ISA despite active bacterial proliferation and division, providing direct evidence that our anchoring strategy mediated by click chemistry ensures the sustained integrity of the interface throughout the bioproduction cycle. Although the substantial intracellular accumulation of PHB (approximately 46% of dry cell weight) necessitates cell harvesting, the demonstrated structural stability of the *C.N*@ISA platform highlights its broad potential for continuous operations, particularly in bio-inorganic systems targeting extracellularly secreted products. To further probe the structural stability of the catalytic centers, ISA samples recovered after operation (denoted as ISA-after) were analyzed by XANES and EXAFS analyzes (Fig. S41). The XANES results show that the Fe oxidation state remains largely unchanged, with no clear evidence of irreversible redox transformation. EXAFS analysis yields a coordination number of 3.7, consistent with a predominantly Fe–N_4_-like configuration. No obvious Fe–Fe scattering signal is observed, suggesting the absence of significant aggregation (Table S4). These findings provide microscopic evidence supporting the long-term stability of the bio–inorganic interface.

The effect of current density on the performance of CO_2_-to-PHB conversion was further studied. Increasing current density from 2.4 mA cm^−2^ to 4.8 mA cm^−2^ led to increased OD_600_ and PHB yield, with *C.N* + ISA and *C.N*@ISA showing more pronounced improvements (Figs. 6a and S42). However, at 5.6 mA cm^−2^, PHB yield decreased (Fig. S43), likely due to excessive oxygen generation disrupting electron balance and inhibiting Rubisco activity in the CBB cycle^49^. The Faradaic efficiency (FE), representing the actual percentage of electrons recovered in the final product (PHB), was used to evaluate electron utilization efficiency. This metric reflects both the effectiveness of the inorganic–biological biohybrid and the intracellular electron allocation for CO_2_ fixation. To accurately quantify the active biomass and exclude optical interference from surface-anchored ISA and variations in cultivation conditions, we strictly avoided using general empirical equations. Instead, we performed a parameter calibration specific to our hybrid system. By decoupling the optical contributions of cells and PHB granules, we derived a specific biomass coefficient of 401 mg biomass L^−1^ OD^−1^ and a PHB scattering coefficient of 785 mg PHB L^−1^ OD^−1^ (Figs. S44 and 45). Based on the recalibrated formula, the FE of *C.N*@ISA reached up to 42.0% at 4.8 mA cm^−2^ (Fig. 6b). Beyond electrons involved in direct CO_2_ reduction, biological CO_2_ fixation requires electrons consumed through oxidative phosphorylation to generate a proton gradient for ATP production. Consequently, the theoretical FE (FE_T_) defines the stoichiometric upper limit of electron conversion efficiency, determined strictly by the specific electron entry point into the ETC and the resulting P/O ratio (ATP generated per electron pair) under ideal conditions^50^. For *C.N*, the P/O ratio is 2.5, corresponding to a FE_T_ of 58.8%. In contrast, for *C.N*@ISA, direct electron delivery to the quinone pool coupled with extracellular H_2_-provided proton input resulted in a P/O ratio of 2.0, giving a FE_T_ of 53.3% (Fig. 6c). Experimentally, FE values were 26.0%, 32.9%, and 42.0% for *C.N*, *C.N* + ISA, and *C.N*@ISA, respectively (Fig. 6b, c). Notably, *C.N*@ISA exhibited a FE of 42.0%, achieving 78.8% of its FE_T_ (53.3%). In contrast, *C.N* reached only 44.2% of its FE_T_. These results collectively demonstrate that anchoring ISA on the microbial cell membrane significantly improves electron and energy utilization efficiency, thereby enhancing biomass accumulation and PHB production in the integrated inorganic-biological system.

## Discussion

In this work, we developed an integrated inorganic-biological biohybrid by covalently anchoring ISA onto the membrane of *C.N* via click chemistry. This interface engineering strategy overcame two key bottlenecks in H_2_-mediated microbial electrosynthesis: restricted H_2_ availability and sluggish hydrogenase kinetics. ISA enriched a localized H_2_ microenvironment and catalyzed H_2_ dissociation with low energy barriers. Meanwhile, the B-PEG linker stabilized the inorganic-biological interface and favored electron/proton transfer. Combined with the electrochemical environment, these effects effectively increased PMF, boosted NADH regeneration and ATP synthesis, meeting both thermodynamic and kinetic demands for CO_2_ fixation via the CBB cycle. Moreover, ISA also exhibited nanozyme-like ROS-scavenging activity, protecting cells from oxidative stress and ensuring microbial viability. This synergistic function—simultaneous H_2_ activation and ROS elimination—further reinforced intracellular energy metabolism. Consequently, *C.N*@ISA achieved a PHB yield of 1058.8 mg L^−1^ with a FE of 42.0%, nearly double that of native *C.N*. Transcriptomic analysis revealed upregulation of Fe-S cluster and ATP synthase genes, confirming enhanced metabolism for CO_2_ to PHB conversion. However, we note that the precise mechanism by which the dissociated hydrogen species (H atoms/protons and electrons) are transferred from the ISA surface to the intracellular cofactor pool warrants further dedicated investigation. Overall, this work demonstrates a microbial surface engineering strategy integrating H_2_ enrichment, accelerated electron/proton transfer, and ROS detoxification. The proposed approach provides a versatile platform for carbon-negative biomanufacturing and holds promise for renewable energy-driven CO_2_ utilization into high-value chemicals and sustainable applications.

## Methods

### Chemicals

2-Methylimidazole (98.0%), zinc nitrate hexahydrate (98.0%), FAD (97.0%) and rotenone (95.0%) were obtained from Aladdin. Fe(III) acetylacetonate (99.9%) was purchased from Sigma-Aldrich. Acetone (99.5%), anhydrous ethanol (99.9%) and D‑fructose were obtained from Sinopharm Chemical Reagent Co., Ltd. B-PEG (total molecular weight 2400 ± 200, 95%) was purchased from Ruixibio Co., Ltd. Methanol (99.9%) was purchased from TEDIA. Phosphate-buffered solution (PBS) was purchased from Yuanye Bio Co., Ltd and stored at room temperature (25 °C). K_3_Fe(CN)_6_ (99.5%) and gentamicin sulfate (98.0%) were purchased from Macklin. Chloroform (99.0%) was obtained from Yonghua Chemical Co., Ltd. DMPO (97%) was purchased from J&K Co., Ltd and aliquoted in a water/acetonitrile solution (0.1 g/mL) as a stock solution. The concentration of DMPO in the stock solution was 0.1 mol/L. Cyanine 5 (95.0%) and 2’,7’-dichlorodihydrofluorescein diacetate (DCFH-DA, 97.0%) were purchased from Yuanye Bio Co., Ltd. Deuterium gas (high purity) was purchased from Minxing Chemical Co., Ltd. Nitrogen gas (high purity) was purchased from Jingong Special Gas Co., Ltd. Hydrogen gas was generated by a hydrogen generator.

### Synthesis of ISA nanomaterials and B-PEG/ISA conjugates

To synthesize ISA, 2-methylimidazole (1314 mg, 0.016 mol) was dissolved in 15 mL of methanol (Solution A). Separately, zinc nitrate hexahydrate (1190 mg, 0.004 mol) and Fe(III) acetylacetonate (70 mg, 0.2 mmol) were dissolved in 30 mL of methanol (Solution B). Solution B was added dropwise to Solution A under continuous magnetic stirring (500 rpm). The mixture was transferred into a 50 mL polytetrafluoroethylene liner, sealed in an autoclave, and heated at 120 °C for 4 h. After cooling to room temperature (25 °C), the precipitate was collected via centrifugation (11,000 rpm, 8 min), washed three times with methanol, and vacuum-dried at 60 °C. The dried solid was ground into a fine powder to obtain Fe-ZIF8 (ZIF: zeolitic imidazolate framework). Pyrolysis of Fe-ZIF8 was carried out under a N_2_ atmosphere at 800 °C, 900 °C, 1000 °C (5 °C min^−1^ ramp, 3 h dwell) to yield ISA-800, ISA-900 and ISA-1000. As a comparison, NC-900 (NC: nitrogen-doped carbon) was synthesized following the same procedures as that of ISA-900, except that Fe(III) acetylacetonate was not added to the precursors. For B-PEG/ISA preparation, a mechanical adsorption method was employed^51,52^. Briefly, pre-synthesized ISA was dispersed in a 5 mg mL^−1^ B-PEG solution prepared in PBS (0.1 M, pH = 7.0 ± 0.04). The mixture was ultrasonicated in an ice-water bath for 30 min to produce a stable B-PEG/ISA dispersion, washed with PBS three times for subsequent use.

### Preparation of *Cupriavidus necator* strain and growth media

The strain *C.N* (ATCC 17699) was obtained from the American Type Culture Collection and stored at −80 °C. Prior to inoculation, a seed culture was derived from a Luria-Bertani (LB) medium maintained at 30 °C for 12 h. The seed culture (2% v/v inoculum) was subsequently transferred into minimal medium (MM) supplemented with 10 g L^−1^ D-fructose for amplification before cell modification^53^. The MM (pH = 7.2 ± 0.1) was composed of Na_2_HPO_4_·12H_2_O (9 g L^−1^), KH_2_PO_4_ (1.5 g L^−1^), (NH_4_)_2_SO_4_ (0.2 g L^−1^), NaHCO_3_ (0.2 g L^−1^), MgSO_4_·7H_2_O (0.08 g L^−1^), CaSO_4_·2H_2_O (1 mg L^−1^), NiSO_4_·7H_2_O (0.56 mg L^−1^). The ingredients were obtained from Sinopharm Chemical Reagent, Shanghai. The prepared solution was sterilized at 121 °C for 15 min.

### Functionalization of cell surface with B-PEG/ISA

[3-(4,5-dimethylthiazol-2-yl)-2,5-diphenyl tetrazolium bromide] (MTT), was used to assess the effect of different concentrations of B-PEG/ISA (1, 5, 7.5, 12.5, 25, 40, 50 μg mL^−1^, 1:1 mass ratio of B-PEG to ISA) on cellular activity. Specifically, *C.N* cells were seeded in 96-well plates at a density of 1 × 10^5^ cells per well and incubated overnight to allow for adherence. Fresh culture medium containing varying concentrations of B-PEG/ISA was introduced, and the cells were incubated for an additional 24 h. Subsequently, 100 μL of MTT solution (0.5 mg mL⁻¹) was added to each well, and the plates were incubated for 6 h. After removing the medium, 100 μL of dimethyl sulfoxide was added to dissolve the formazan product. Cell viability was quantified by measuring absorbance at 570 nm using a microplate reader (Varioskan LUX, Thermo Fisher). For functionalization, a bacterial suspension of *C.N* (OD_600_ ~ 1.0, 50 mL) was centrifuged at 5000 rpm for 5 min. The bacterial precipitate was washed with 0.1 M PBS (pH = 7.0 ± 0.04) to remove residual fructose, and resuspended in PBS. To functionalize the cell surface with B-PEG/ISA, 150 mL bacterial suspension of *C.N* was mixed with varying volumes, 0.03–1.2 mL, of a 5 mg mL^−1^ B-PEG/ISA solution (1:1 mass ratio of B-PEG to ISA). The mixture was incubated at 30 °C under gentle agitation (100 rpm) for 10 min, followed by multiple washes with fresh PBS to remove unbound nanoparticles. The resulting pellet (*C.N*@ISA) was resuspended in fresh PBS for subsequent experiments.

### Reactor configuration and operation

A single-chamber electrochemical reactor (working volume: 150 mL) was assembled using a stainless-steel mesh cathode (3 × 2.5 cm^2^) and a platinum mesh anode (1 × 1 cm^2^). The reactor was filled with MM and inoculated with *C.N* or *C.N*@ISA at an initial OD_600_ of 0.3. A control group in which suspended ISA was added exogenously after inoculation was designated as *C.N* + ISA. Long-term electrolysis was conducted at constant current densities of 2.4, 4.8, and 5.6 mA cm^−2^ with periodic sampling (WMPG1000K8, WonATech). At 24 h intervals, OD_600_, NADH/NAD^+^, ATP concentrations, and PHB yields were measured. Then CO_2_ was aerated through a rotameter at a flow rate of 80 mL min^−1^ for 15 min. Additionally, to assess the impact of ISA modification on cell viability, intracellular ROS levels and cell viability were measured at regular time intervals. For intracellular ROS detection, 0.5 mL of bacterial culture from the mixed reactor was mixed with 0.5 mL of a 20 μM 2’,7’-dichlorodihydrofluorescein diacetate (DCFH-DA) solution. The mixture was incubated in the dark at 30 °C for 20 min. After incubation, the culture was washed three times with 0.1 M PBS. The resulting bacterial suspension was then transferred to a 96-well plate for fluorescence measurement (excitation *λ*_ex_ = 488 nm, emission *λ*_em_ = 525 nm). Cell viability was determined by using dual staining with SYTO 9 nucleic acid stain and propidium iodide (PI). Viable cells emitted green fluorescence (*λ*_ex_ = 485 nm, *λ*_em_ = 530 nm), whereas dead cells emitted red fluorescence (*λ*_ex_ = 485 nm, *λ*_em_ = 630 nm). Cell viability was calculated using the following equation:

1 $${{{\rm{Cell\,viability}}}}\,(\%)=\frac{{I}_{G}}{{I}_{G}+{I}_{R}}\times 100\%$$

where *I*_R_ and *I*_G_ represent the fluorescence intensities of PI and SYTO, respectively. Cells collected during the operation were collected for RNA sequencing to analyze differential gene expression among experimental groups.

### Physicochemical characterization of ISA nanomaterials

The morphology and elemental distribution of ISA were examined via SEM (SU-8010, Hitachi) and TEM (HT-7700, Hitachi) equipped with energy-dispersive EDS (JEM-2100plus, JEOL). HAADF-STEM (Titan 80-300, Thermo Fisher) was used to resolve single-atom structures of ISA. XRD (XRD-6000, Shimadzu). Raman spectroscopy (DXR SmartRaman, Thermo Fisher) was employed to probe the composition and crystalline structure of ISA. The BET method was applied to measure the specific surface area using a surface area analyzer (ASAP2460, Micromeritics) through N_2_ adsorption-desorption experiments. XPS (Escalab 250Xi, Thermo Fisher) was employed to characterize the elemental composition, surface valence states, and chemical structure. The X-ray absorption fine structure (XAFS, 1W1B beamline, Beijing Synchrotron Radiation Facility) spectra were collected in transmission mode using the ionization chamber for Fe foil, FeO, Fe_2_O_3_ standards, and in fluorescence excitation mode using the Lytle detector for ISA and FePc. The EXAFS spectra were obtained by subtracting the post-edge background and normalizing to the edge-jump step. FT of *χ*(*k*) into *R* space was extracted by least-squares curve fitting using the ARTEMIS module of IFEFFIT software packages. WT of *χ(k)* was processed with the Hama Fortran code.

### Characterization of B-PEG/ISA-cell surface conjugation

Zeta potentials of ISA and B-PEG/ISA were measured using dynamic light scattering (DLS, Zetasizer Nano-ZS, Malvern). Functional groups were identified by FTIR (Nicolet iS10, Thermo Fisher) and Raman spectroscopy (LabRAM, Horiba). Interaction among B-PEG, ISA, and *C.N* cells was further examined using SEM, TEM (HT-7820, Hitachi) and CLSM (LSM 780, Zeiss). For fluorescence visualization, live *C.N* cells and B-PEG were stained with 4’,6-diamidino-2-phenylindole (DAPI, Beyotime Biotechnology) and Cyanine5 (Cy5, 2 mg L⁻¹, Yuanyebio), respectively. In addition, *α*-sites of polysaccharide were stained using Concanavalin A lectin (ConA, 2 g L^−1^, Aladdin), while *β*-sites of polysaccharides were stained with Calcofluor White (Coolaber Technology) to localize the specific binding sites of B-PEG/ISA on the cell membrane. CLSM images were processed using ImageJ to calculate the mean fluorescence intensity (MFI) of cells. Background fluorescence intensity was subtracted from the measured values, and the corrected MFI reflected the relative abundance of fluorescently labeled *α*-sites and *β*-sites of polysaccharides (with the *C.N* group data as the reference). All statistical evaluations in this work were performed using unpaired two-sided *t*-test analysis.

### Competitive binding assay

B PEG/ISA (5 mg mL^−1^, 1 mL) was mixed with 1 mL of a D-fructose solution (0.4 M). The mixture was incubated at 30 °C with gentle shaking (100 rpm) for 10 min, followed by centrifugation. The pellet was washed with PBS (0.1 M, pH = 7.0 ± 0.04) and then resuspended in 1 mL of PBS to obtain fructose-treated B PEG/ISA. A bacterial suspension of *C.N* from an expanded culture (OD_600_ ≈ 1.0, 50 mL) was centrifuged. The bacterial pellet was collected, washed, and then resuspended in PBS. Subsequently, 150 mL of the aforementioned *C.N* suspension was mixed with 0.375 mL of the fructose-treated B PEG/ISA and incubated at 30 °C with gentle shaking (100 rpm) for 10 min. The bacterial mixture was centrifuged, washed with PBS, and the resulting pellet was preserved for electron microscopy imaging.

### Evaluation of hydrogen availability and ISA-facilitated hydrogen activation

The intrinsic catalytic activity of ISA toward H_2_ activation was preliminarily evaluated using RRDE measurements (RRDE-3A, ALS)^28^. A glassy carbon disk electrode (4 mm diameter, surface area of 0.1256 cm^2^), either unmodified or coated with ISA (dispersed by ultrasonication in isopropanol containing 0.5 wt% Nafion, with an ISA loading of 0.4 mg cm^−2^), was used as the working electrode, with Pt wire and saturated Ag/AgCl electrodes as counter and reference electrodes, respectively. H_2_-saturated PBS (0.1 M, pH = 7.0 ± 0.04) was used as the electrolyte. Linear sweep voltammetry (LSV) was performed from −0.41 to 0.15 V vs. SHE (standard hydrogen electrode, after 85% *iR* correction) at a scan rate of 10 mV s^−1^, and the ring potential was set to −0.51 V vs. SHE. The electrochemical tests were carried out at room temperature (25 °C). The bacterial culture after expansion was collected, washed with PBS, and then diluted with MM medium to an OD_600_ of approximately 0.2. The diluted bacterial suspension was mixed with a B-PEG/ISA solution (12.5 mg L^−1^) and incubated on a shaker at 30 °C for 10 min to obtain *C.N*@ISA. Aliquots (1 mL each) of *C.N*, *C.N*@ISA (OD_600_ ≈ 0.2), and ISA (12.5 mg L⁻¹) were prepared. DMPO (final concentration 0.1 mol L^−1^) was added, followed immediately by bubbling 15 mL of H_2_. The samples were then transferred into quartz capillary tubes for EPR detection. The three experimental groups carrying the mechanistic conclusions—DMPO + H_2_ + *C.N*, DMPO + H_2_ + ISA, and DMPO + H_2_ + *C.N*@ISA—were all prepared in the identical MM matrix to eliminate any matrix-dependent artefacts. Two parallel experimental controls were included in the same MM matrix to rule out background contributions from H_2_ bubbling alone (DMPO + H_2_) and from the bacteria alone without H_2_ (DMPO + *C.N*). In addition, a reagent quality-control sample (DMPO only in deionized water) was measured prior to each batch of experiments to verify the integrity of the spin trap. EPR acquisition parameters: resonance frequency, 9.85 GHz; microwave power, 20.43 mW; modulation frequency, 100.00 kHz; modulation amplitude, 1.00 G; time constant, 81.92 ms; sweep time, 20.00 s; receiver gain, 1.00 × 10^4^. Direct H_2_ bubbling systems with *C.N* or *C.N*@ISA were supplied with a continuous flow of gases: H_2_, CO_2_, and O_2_, in a ratio of 16:1:3 (V_H2_:V_CO2_:V_O2_). In parallel, deuterium labeling experiments were conducted by replacing H_2_ with D_2_. After 96 h of gas cultivation, 10 mL of bacterial culture was collected and centrifuged (4 °C, 8000 rpm, 5 min). The supernatant was discarded, and the cell pellet was washed twice with deionized water, followed by overnight drying at 55 °C. The dried samples were reacted in an acidified methanol solution (a mixture of 1.98 mL methanol, 0.02 mL H_2_SO_4_, and 2 mL chloroform) at 100 °C for 4 h. After the reaction, 1 mL of deionized water was added to each reaction mixture and thoroughly vortexed. Extracted PHB was analyzed using a triple quadrupole gas chromatography-mass spectrometry system (7890B/7000C, Agilent). DFT calculations were employed to investigate the capability of ISA in adsorbing and dissociating H_2_ molecules.

### Evaluation of electron transfer in the biohybrids

Bacterial suspensions from the electrochemical–biological integrated reactors (*C.N*, *C.N* + ISA, and *C.N*@ISA groups) operated for 72 h were collected for CV measurements. The reactor broth was used directly as the electrolyte. A stainless-steel electrode, a Pt mesh, and a saturated Ag/AgCl electrode were employed as the working, counter, and reference electrodes, respectively. The potential was scanned from −0.6 to 0.5 V vs. SHE at a scan rate of 10 mV s^−1^. The EIS measurements were performed using the same setup as that for the CV tests, at a potential of −0.05 V vs. SHE, over a frequency range of 0.01–100,000 Hz. The potentials can be converted to the RHE potential scale using *E* (vs. RHE) = *E* (vs. Ag/AgCl) + 0.197 V + 0.0591 × pH. In addition, the bacterial suspensions collected from the reactor were analyzed by HPLC (1260 infinity, Agilent). Specifically, the cultures were gently vortexed and then centrifuged at 4000 rpm for 3 min, and the supernatants were subjected to analysis. Separation was performed on a C18 column (Eclipse XDB-C18, Agilent, 250 × 4.6 mm, 5 μm). The mobile phase consisted of 70% sodium acetate solution (6.8 g L^−1^, pH = 5.0 ± 0.05) and 30% methanol. The injection volume was 20 μL, the flow rate was 1 mL min^−1^, and the column temperature was maintained at 35 °C. Detection was carried out using a variable-wavelength UV detector (VWD) with the absorption wavelength set at 284 nm.

### DFT calculations and simulations

Free energy and transition state (TS) calculations were performed using the spin-unrestricted DFT method as implemented in the DMol3 code within the Materials Studio package^54^. The electron exchange and correlation effects were described by the generalized gradient approximation based on the Perdew–Burke–Ernzerhof, and a double numerical plus polarization (DNP) basis set was adopted^55^. The convergence tolerance for energy change, max force, and max displacement were 2.0 × 10^−5^ Ha, 0.004 Ha Å^−1^, and 0.005 Å, respectively. To avoid artificial interactions, a vacuum layer with a thickness of 15 Å was used. Based on the Monkhorst-Pack scheme, a 3 × 3 × 1 k-point grid was employed to perform the Brillouin-zone integrations^56^. The complete linear synchronous transit/quadratic synchronous transit (LST/QST) method and mode-eigenvector following approach were adopted to probe the TS of elementary reactions. For each subsequent elementary step, the free energy *G* of the computational model is defined as:

2 $$G=E+{E}_{{{{\rm{ZPE}}}}}+\int {C}_{{{{\rm{p}}}}}{{{\rm{d}}}}T-{TS}$$

where *E* is the electronic energy, ZPE is the zero-point energy correction to the Gibbs free energy. *C*_p_ and *S* are the heat capacity and entropy, respectively. The room temperature *T* = 298.15 K is used in the calculations to take temperature influence into consideration. The optimized models for calculations are provided in Supplementary Data 1. To account for solvation effects in the aqueous environment, the calculations of free energies and transition states were carried out using the conductor-like screening model (COSMO) as an implicit solvation model, with water as the solvent (dielectric constant ε = 78.54). Van der Waals interactions were treated using Grimme’s DFT-D method. It is important to acknowledge the inherent limitations of the current theoretical models. DFT calculations are conducted under idealized conditions and thus may not fully reflect the complexity of the actual catalytic interface under operating conditions. Therefore, theoretical computations primarily serve as a complementary explanation to experimental observations.

### Membrane potential measurement

Bacterial suspensions from different groups (*C.N*, *C.N* + B-PEG, *C.N* + ISA, and *C.N*@ISA, with an OD_600_ of approximately 0.1) were incubated with 4 μM diethyloxacarbocyanine iodide (DiOC₂(3)) at 37 °C for 15 min to allow dye loading. The suspensions were then gassed with H₂ for 15 min. A negative control was included by adding the protonophore CCCP (60 μM, 5 min incubation) between the dye loading and H_2_ exposure steps. Fluorescence intensity was measured using a microplate reader (Varioskan Lux, Thermo Fisher) at excitation/emission wavelengths of 484 nm/525 nm (green) and 484 nm/610 nm (red). Membrane potential was quantified as the ratio of red fluorescence (610 nm) to green fluorescence (525 nm). The PMF can be expressed as:

3 $${{{\rm{PMF}}}}={\Delta }_{\psi }-\frac{2.303{RT}}{F}\times \Delta {{{\rm{pH}}}}$$

where Δ*ψ* is the membrane potential and ΔpH is the transmembrane proton gradient. 2.3*RT*/*F* ≈ 60 mV at 30 °C.

### Performance evaluation of integrated inorganic-biological system

OD_600_ values were measured using an ultraviolet spectrophotometer (Evolution 201, Thermo Fisher). NADH/NAD⁺ values were quantified using the NADH/NAD⁺ assay kit (Nanjing Jiancheng Bioengineering Institute), and ATP levels were measured with an ATP assay kit (Beyotime Biotechnology). PHB yield was quantified through high-performance liquid chromatography (HPLC 1260, Agilent), based on the detection of crotonic acid, which is produced by the heated reaction of PHB with concentrated H_2_SO_4_ (98%). Before the HPLC analysis, the sediment of centrifuged samples was digested with 98 wt.% H_2_SO_4_ at 90 °C for 60 min. The digested solution was diluted with deionized water and passed through a 0.22 μm filter. Samples were analyzed under the following conditions: column, Aminex HPX-87H; mobile phase, 2.5 mM H_2_SO_4_ aqueous solution; flow rate, 0.5 mL min^−1^; injection volume, 100 µL; column temperature, 30 °C; detection at 210 nm. The interval between two injections was 40 min. Dry cell weight (DCW) was estimated using an empirical calibration curve established in preliminary experiments, correlating OD_600_ with experimentally measured dry cell weight. Briefly, cells were harvested at different OD_600_ values, washed, dried to constant weight, and used to construct a standard OD_600_–DCW calibration curve. This yielded a conversion formula of DCW = 471 mg L^−1^·OD_600_. Throughout the study, this calibration curve and its corresponding empirical equation were applied to calculate DCW from the measured OD_600_ values.

### ECE and FE calculations

*C.N* converts CO_2_ into PHB and biomass via the reactions:

PHB production:

$$\begin{array}{c}4{{{\rm{n}}}}\,{{{{\rm{CO}}}}}_{2}\left({{{\rm{g}}}}\right)+3{{{\rm{n}}}}\,{{\mbox{H}}}_{2}{{{\rm{O}}}}\left({{{\rm{l}}}}\right)\to {\left({{{{\rm{C}}}}}_{4}{{{{\rm{H}}}}}_{6}{{{{\rm{O}}}}}_{2}\right)}_{{{{\rm{n}}}}}\left({{{\rm{s}}}}\right)+4.5{{{\rm{n}}}}\,{{{{\rm{O}}}}}_{2}({{{\rm{g}}}})\\ {\Delta }_{{{{\rm{r}}}}}{G}^{\theta } \sim {\Delta }_{{{{\rm{r}}}}}{H}^{\theta }=1903\,{{{\rm{kJ}}}}\,{{{{\rm{mol}}}}}^{-1}{{{\rm{per\,monomer}}}}\end{array}$$

Biomass formation:

$$\begin{array}{c}{{{{\rm{CO}}}}}_{2}\left({{{\rm{g}}}}\right)+0.24\,{{{{\rm{NH}}}}}_{3}\left({{{\rm{aq}}}}\right)+0.525\,{{{{\rm{H}}}}}_{2}{{{\rm{O}}}}\left({{{\rm{l}}}}\right)\to {{{{\rm{CH}}}}}_{1.77}{{{{\rm{O}}}}}_{0.49}{{{{\rm{N}}}}}_{0.24}\left({{{\rm{s}}}}\right)+1.02{{{{\rm{O}}}}}_{2}({{{\rm{g}}}})\\ {\Delta }_{{{{\rm{r}}}}}{G}^{\theta }=479\,{{{\rm{kJ}}}}\,{{{{\rm{mol}}}}}^{-1}\end{array}$$

To accurately quantify the active biomass of *C. necator* in our specific system, we performed a parameter calibration experiment following the methodology established by Liu et al.^7^, using *C. necator* cells without ISA. This decoupled the optical contributions of cellular biomass and intracellular PHB granules.

Determination of the biomass coefficient: By analyzing early-stage samples (0–24 h, with negligible PHB), we correlated OD_600_ with DCW and derived a specific biomass coefficient of 401 mg biomass/L/OD; Determination of the PHB scattering coefficient: Using late-stage samples (48–120 h) with varying PHB content (quantified by HPLC), we isolated the optical density contribution of PHB (OD_PHB_) and derived a specific coefficient of 785 mg PHB/L/OD_PHB_.

The biomass calculation formula was ultimately established as:

4 $${c}_{{{{\rm{biomass}}}}}\left({{{\rm{mg}}}}{{{{\rm{L}}}}}^{-1}\right)=\left\{{{{{\rm{OD}}}}}_{600}-\frac{{c}_{{{{\rm{PHD}}}}}\left({{{\rm{mg}}}}\cdot {{{{\rm{L}}}}}^{-1}\right)}{785}\right\}\times 401$$

Where *c*_PHB_ is the concentration determined by HPLC.

The ECE was calculated as:

5 $${{{\rm{ECE}}}}\left(\%\right)=\frac{{E}_{{{{\rm{biomass}}}}}+{E}_{{{{\rm{PHB}}}}}}{\int \left({V}_{{{{\rm{MM}}}}}\times I\right){{{\rm{d}}}}t}\times 100\%$$

6 $${E}_{{{{\rm{PHB}}}}}=\Delta {c}_{{{{\rm{PHB}}}}}\times {\Delta }_{{{{\rm{r}}}}}{G}_{{{{\rm{PHB}}}}}^{0}\times {V}_{{{{\rm{MM}}}}}$$

7 $${E}_{{{{\rm{biomass}}}}}={\Delta c}_{{{{\rm{biomass}}}}}\times {\Delta }_{{{{\rm{r}}}}}{G}_{{{{\rm{biomass}}}}}^{0}\times {V}_{{{{\rm{MM}}}}}$$

Where the *V*_MM_ represents the volume of the medium in the reactor. *I* represents the current applied to the reactor, and *t* indicates the operation time. $\Delta {c}_{{\mbox{PHB}}}$ and $\Delta {c}_{{\mbox{biomass}}}$ denote the variations in PHB concentration and biomass concentration, respectively (mg L^−1^). Δ_r_${G}_{{\mbox{PHB}}}^{0}$ and Δ_r_${G}_{{\mbox{biomass}}}^{0}$ are the Gibbs free energy of PHB and biomass, respectively (J).

Quantification of residual H_2_ from the integrated system during operation was performed using gas chromatography (GC9790II, Fuli). High-purity nitrogen gas served as the carrier gas, with a thermal conductivity detector integrated into the system. The chromatographic columns consisted of a 5 Å molecular sieve, with a 1 mL quantitative loop. The external standard method was applied to determine the H_2_ concentration. Based on the assumption of 100% cathodic H_2_ evolution efficiency, the system’s uptaken H_2_ amount can be calculated using the formula below:

8 $${m}_{{{{\rm{H}}}}2}\left({{{\rm{g}}}}\right)=\frac{\int I{{{\rm{d}}}}t}{F}-\frac{{c}_{{{{\rm{H}}}}2}\times {V}_{{{{\rm{g}}}}}}{1000}$$

The FE for PHB production (FE_PHB_) and FE for biomass production (FE_biomass_) were determined using:

9 $${{FE}}_{{{{\rm{PHB}}}}}\left(\%\right)=\frac{{\Delta c}_{{{{\rm{PHB}}}}}\times {V}_{{{{\rm{MM}}}}}\times 26\times F}{86\times \int I{{{\rm{dt}}}}}\times 100\%$$

10 $${{FE}}_{{{{\rm{biomass}}}}}\left(\%\right)=\frac{{\Delta c}_{{{{\rm{biomass}}}}}\times {V}_{{{{\rm{MM}}}}}\times 4.07\times F}{24.97\times \int I{{{\rm{dt}}}}}\times 100\%$$

Where *c*_H2_ represents the H_2_ concentration obtained via GC detection (mg L^−1^), *V*_g_ denotes the volume of gas collected during the operation (L), and *F* is the Faradaic constant (96,500 C mol^−1^). The coefficient 26 refers to the electron molar number required to synthesize 1 mol of PHB monomer, and 4.07 corresponds to the electron molar number required to synthesize 1 mol of biomass^57^. The relative molecular weight of the PHB monomer is 86, while that of the biomass is 24.97.

The FE_T_ is defined as the percentage of reducing equivalents required for yielded products achievable by the biochemical machinery relative to the uptaken electron. The calculation of FE_T_ only considers the consumption of reducing equivalents directly involved in the net redox reaction but not the other consumptions in microbial metabolic processes. Therefore, the consumption of reducing power by peripheral reactions essential for maintaining microbial activity is excluded from consideration here^50^.

The FE_T_ remains below unity because the CO₂ fixation pathway requires not only NAD(P)H for CO₂ reduction but also consumes reducing equivalents (electrons or NAD(P)H) through oxidative phosphorylation to generate ATP. In the case of CO₂ fixation, we confine our discussion to the CBB cycle in *C.N* that converts CO_2_ and coenzyme A (HS-CoA) into one equivalent of acetyl coenzyme A (acetyl-CoA) with the following net reaction:

$$\begin{array}{c}{2\,{{{\rm{CO}}}}}_{2}+7\,{{{\rm{ATP}}}}+4\,{{{\rm{NAD}}}}\left({{{\rm{P}}}}\right){{{\rm{H}}}}+{{{\rm{HS}}}}-{{{\rm{CoA}}}}+4\,{{{{\rm{H}}}}}^{+}\\ \to {{{\rm{Acetyl}}}}-{{{\rm{CoA}}}}+7\,{{{\rm{ADP}}}}+7\,{{{\rm{Pi}}}}+4\,{{{\rm{NAD}}}}{({{{\rm{P}}}})}^{+}\end{array}$$

In this work, the P/O ratios of 2.5 and 2 are derived based on the number of protons pumped per pair of electrons transferred into O, which ultimately reflects the ATP yield. In acknowledged oxidative phosphorylation, one pair of electrons starting from NADH passes through the ETC, pumping 10 protons upon reduction of O, thereby generating 2.5 (10/4) ATP. After B-PEG/ISA modification, H_2_ dissociation produces 2 protons and one pair of electrons. This pair of electrons enters the quinone pool and subsequently passes through the ETC to reduce O, pumping 6 protons. In total, 8 external protons are available, generating 2 (8/4) ATP. The 7 ATP in the aforementioned net reaction would thus require 2.8 (7/2.5) or 3.5 (7/2) pairs of electrons. The corresponding FE_T_ are 58.8% (4/(4 + 2.8)) and 53.3% (4/(4 + 3.5)), respectively.

### Reporting summary

Further information on research design is available in the Nature Portfolio Reporting Summary linked to this article.

## Supplementary information

Supplementary Information

Description of Additional Supplementary Files

Supplementary Data 1

Reporting Summary

Transparent Peer Review file

## Source data

Source Data

## Data Availability

The data supporting this study have been provided in the Supplementary Information. All data underlying this study are available from the corresponding author upon request. The RNA-seq data are deposited to National Center for Biotechnology Information (NCBI) under the BioProject number PRJNA1478773. Source data are provided with this paper.
