# Supplementary material for: Interface-engineered iron single-atom biohybrids for efficient CO2-to-bioplastic conversion
Source: Nat Commun. 2026 Aug 20;17:9946. doi: 10.1038/s41467-026-76902-9 (PMC13586381; doi:10.1038/s41467-026-76902-9)
Supplement: Supplementary file 1 [file 41467_2026_76902_MOESM1_ESM.pdf]

Supplementary Information

Interface-engineered iron single-atom biohybrids for efficient CO_2-to-bioplastic conversion

Sulin Ni[1,#], Dong Xia[2,#], Can Chen[3], Cheng Qiu[1], Youzhi Li[4], Bin Yang[1,4], Yang Hou[1], Lecheng Lei[1,4], Zhengwei Mao[5,*], Zhongjian Li[1,4,*]

[1] College of Chemical and Biological Engineering, Key Laboratory of Biomass Chemical Engineering of Ministry of Education, Zhejiang University, Hangzhou 310027, China

[2] State Key Laboratory of Green and Efficient Development of Phosphorus Resources, School of Chemical Engineering and Pharmacy, Wuhan Institute of Technology, Wuhan 430205, China

[3] College of Life Science, Zhejiang University, Hangzhou 310027, China

[4] Institute of Zhejiang University – Quzhou, Quzhou 324000, China

[5] MOE Key Laboratory of Macromolecular Synthesis and Functionalization, Department of Polymer Science and Engineering, Zhejiang University, Hangzhou 310027, China

[#] These authors contributed equally to this work.

* Corresponding authors: Zhongjian Li (Email: zdlizj@zju.edu.cn), Zhengwei Mao (Email: zwmao@zju.edu.cn)

Table-of-contents

1. Supplementary Figures

Section 1.1 | Structural characterization (Fig. S1–S15).

Section 1.2 | Inorganic-biological interface characterization (Fig. S16–S20).

Section 1.3 | Thermodynamic and kinetic evaluation of H_2 activation on ISA (Fig. S21-S23).

Section 1.4 | Proton transport and electron transfer characterization (Fig. S24–S29).

Section 1.5 | ROS elimination detection (Fig. S30 and S31).

Section 1.6 | Transcriptomic analysis (Fig. S32 and S33).

Section 1.7 | Operation of the integrated inorganic-biological system (Fig. S34–S45).

2. Supplementary Tables

Supplementary Table 1 | EXAFS data fitting results of samples.

Supplementary Table 2 | Parameter estimates and fitting errors for the EIS data.

Supplementary Table 3 | Performance comparison with previous studies.

Supplementary Table 4 | EXAFS data fitting results of samples after operation (ISA-after).

1. Supplementary Figures

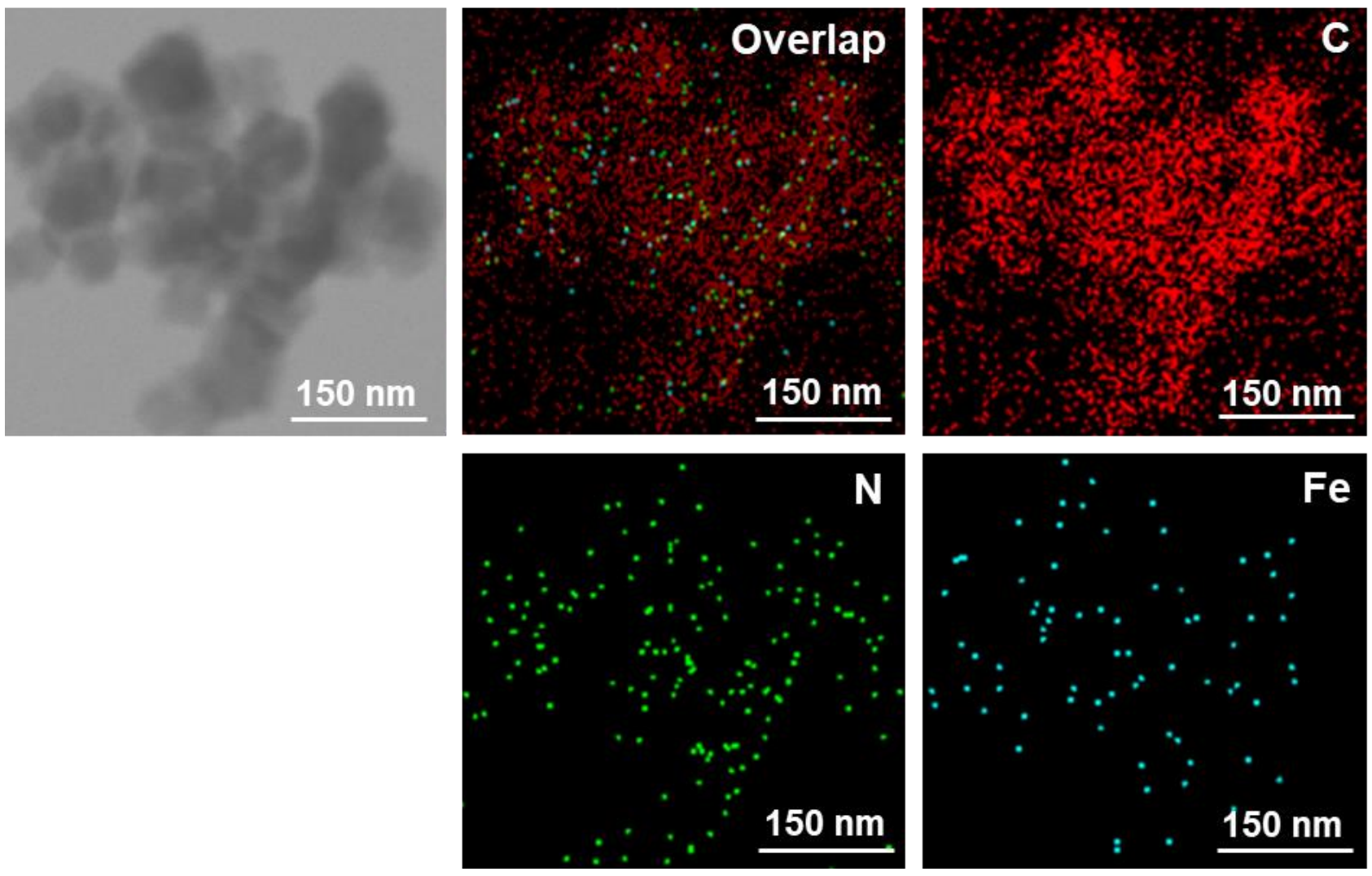

Fig. S1. EDS image of ISA, corresponding element maps showing the distribution of C (red), N (green), and Fe (blue).

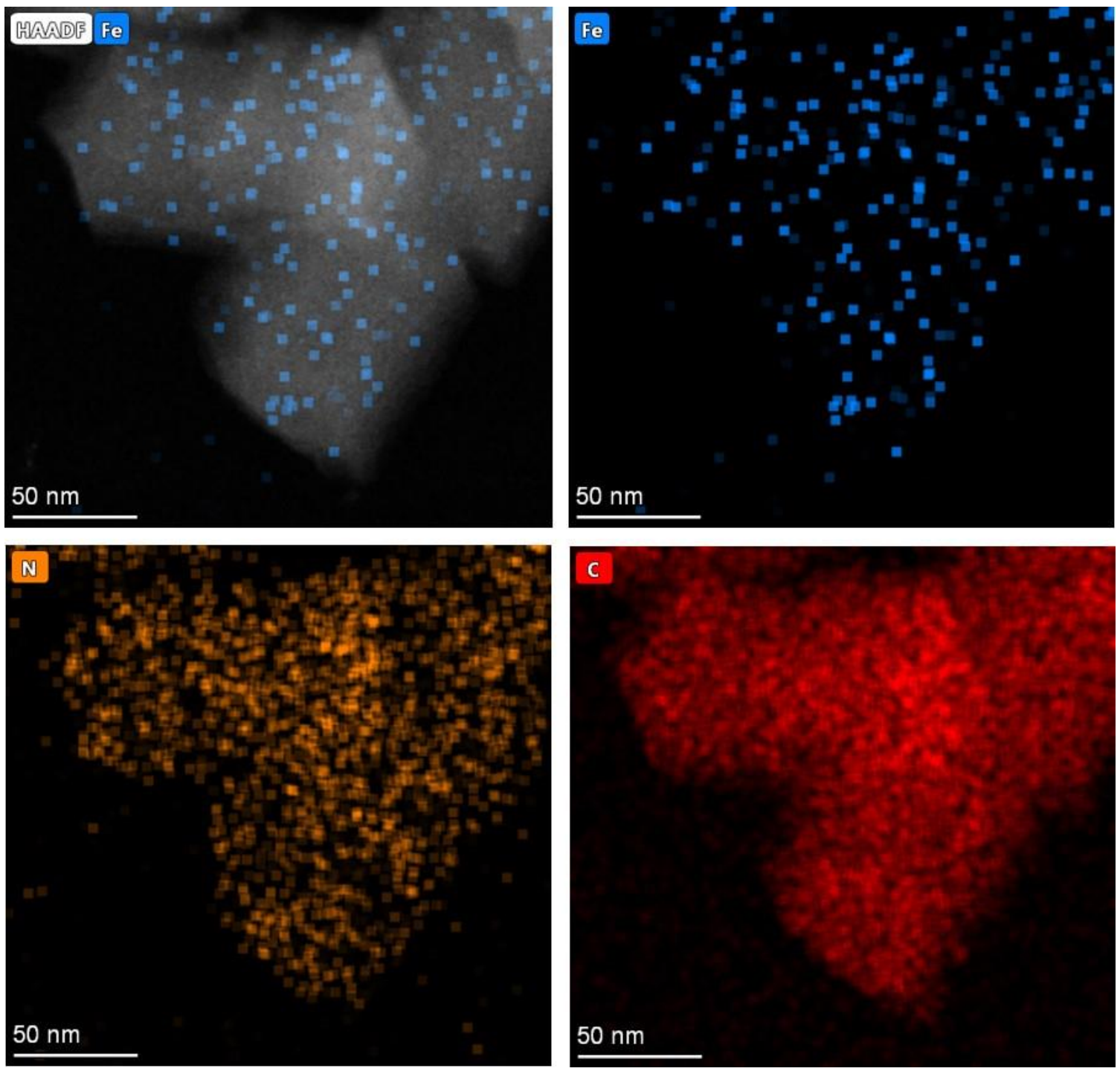

Fig. S2. Dark-field STEM image and the EDS elemental mappings for ISA.

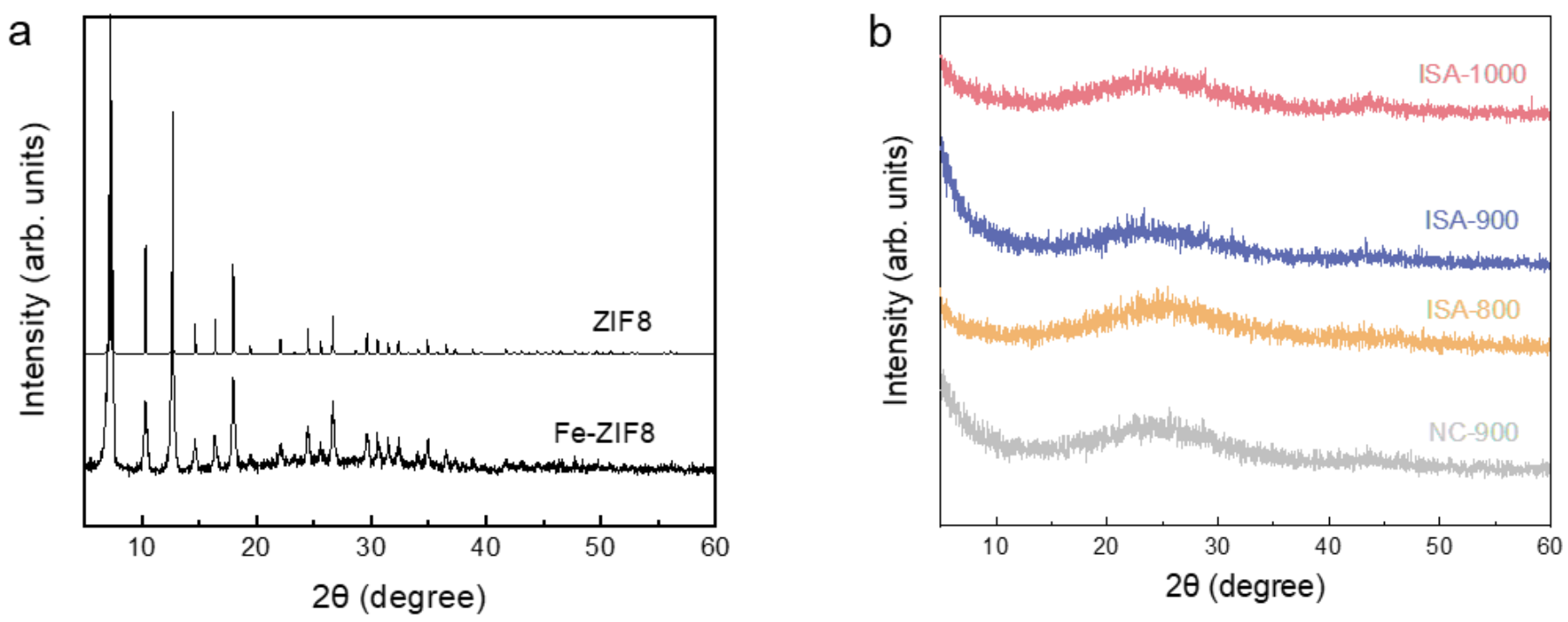

Fig. S3. (a) XRD patterns of the Fe-ZIF8 and ZIF8. (b) XRD patterns of the NC-900, ISA-800, ISA-900, and ISA-1000. Source data are provided as a Source Data file.

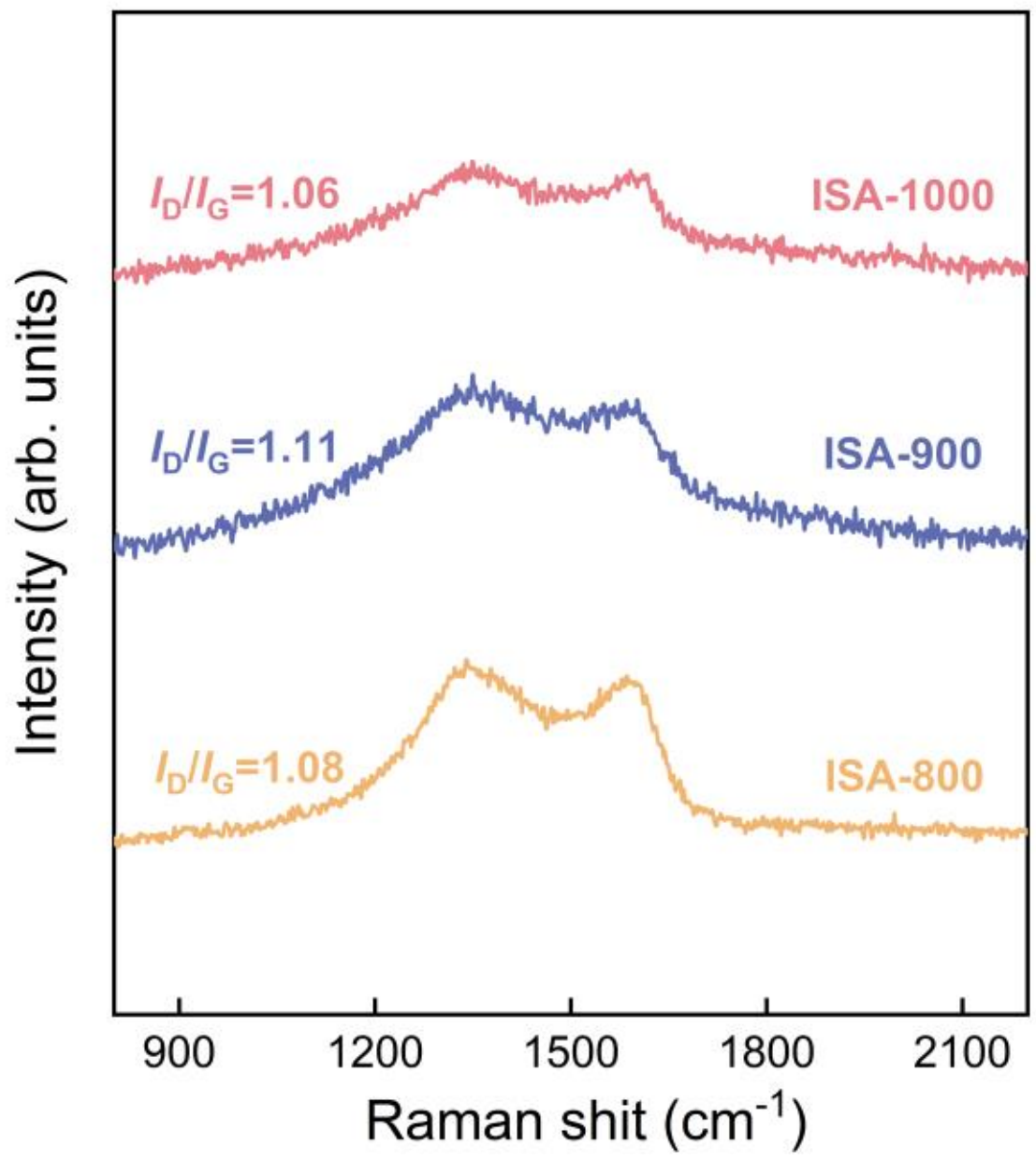

Fig. S4. Raman spectra of ISA-800, ISA-900, and ISA-1000. Source data are provided as a Source Data file.

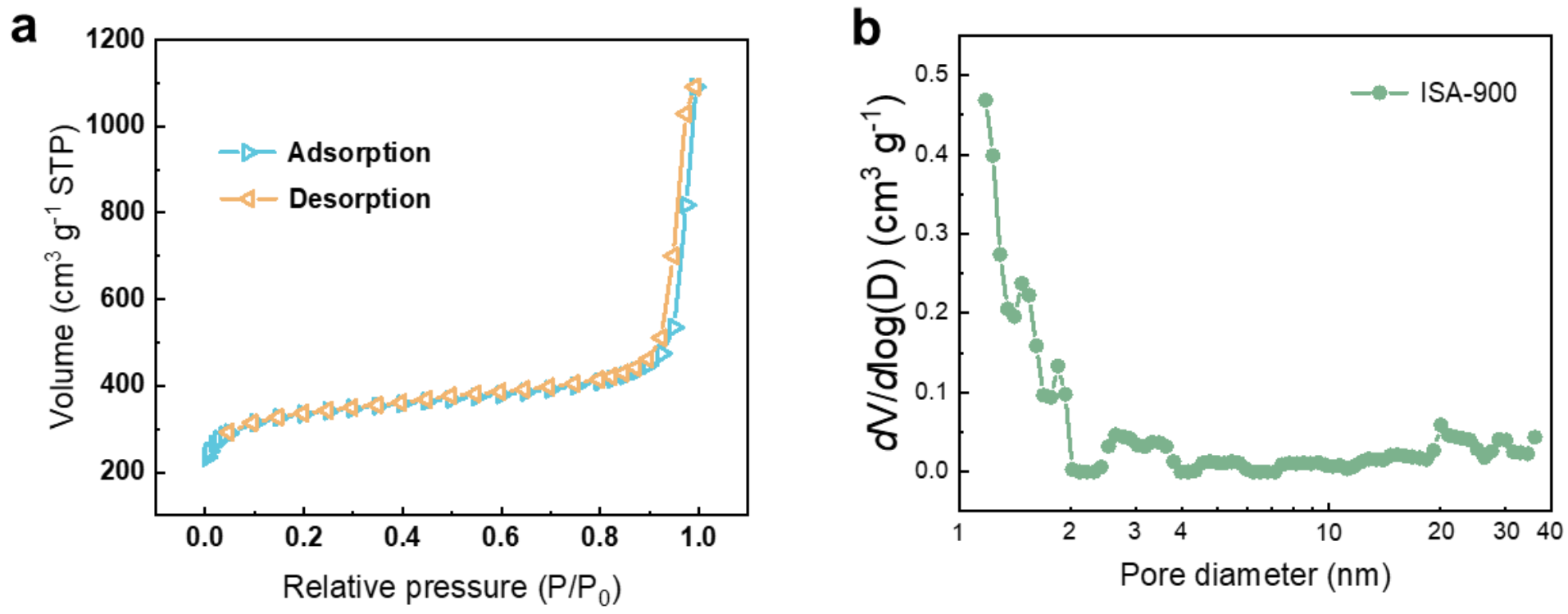

Fig. S5. (a) N_2 adsorption/desorption isotherms and (b) pore size distribution of ISA. Source data are provided as a Source Data file.

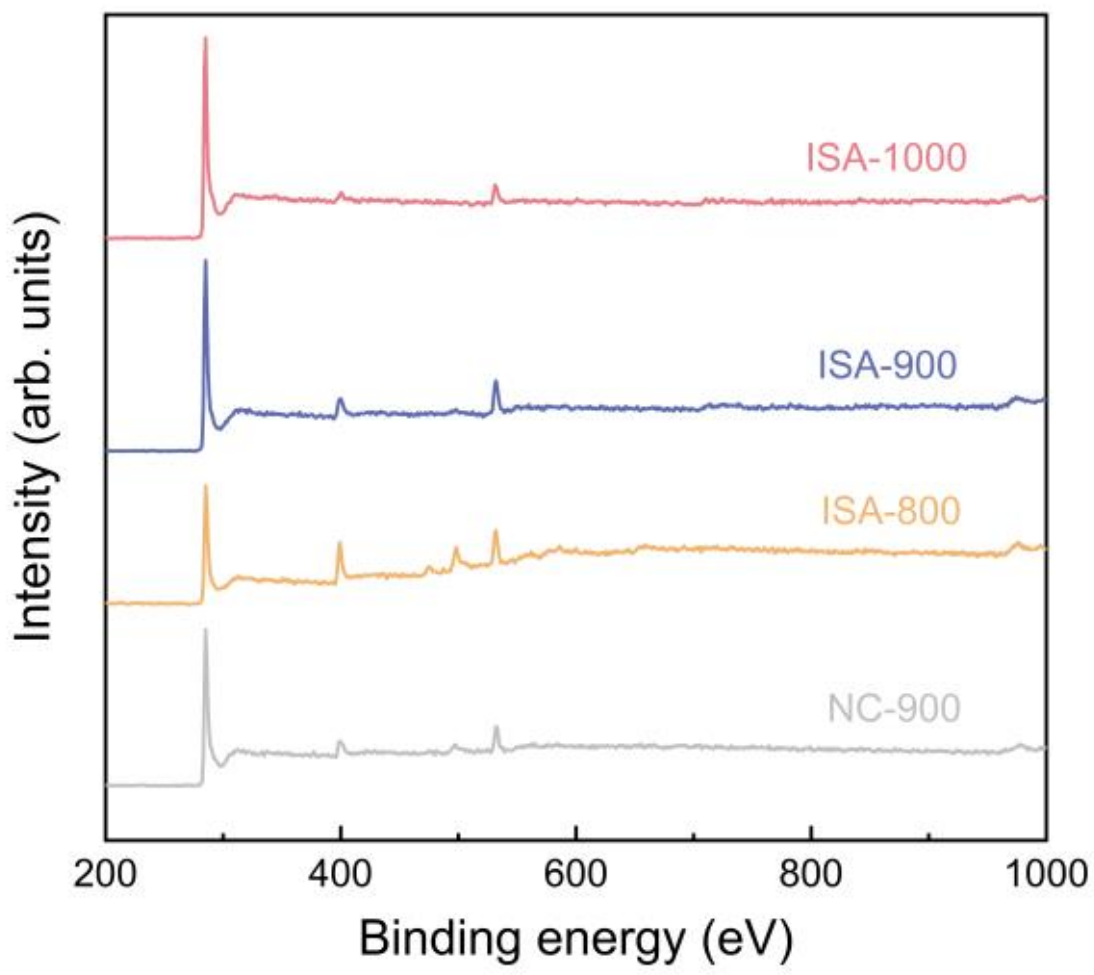

Fig. S6. Survey XPS spectra of NC-900, ISA-800, ISA-900, and ISA-1000. Source data are provided as a Source Data file.

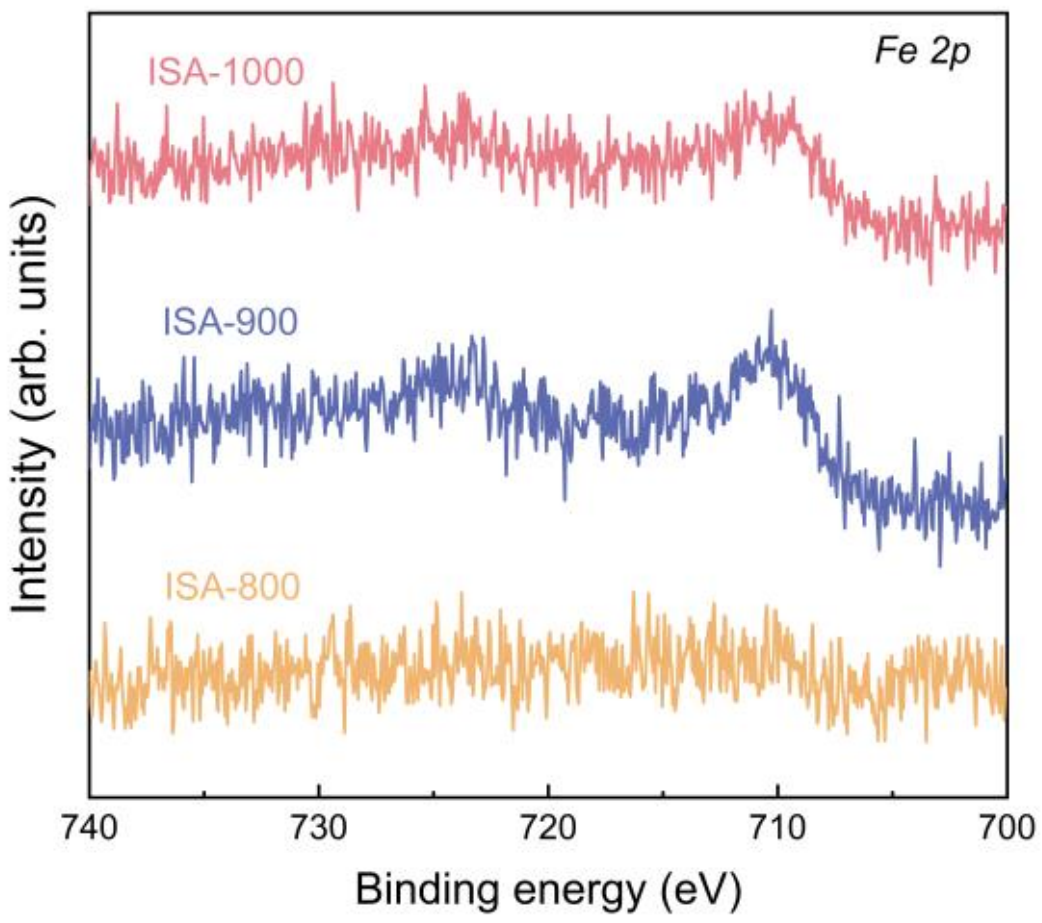

Fig. S7. Fe 2p XPS spectra of ISA-800, ISA-900, ISA-1000. Source data are provided as a Source Data file.

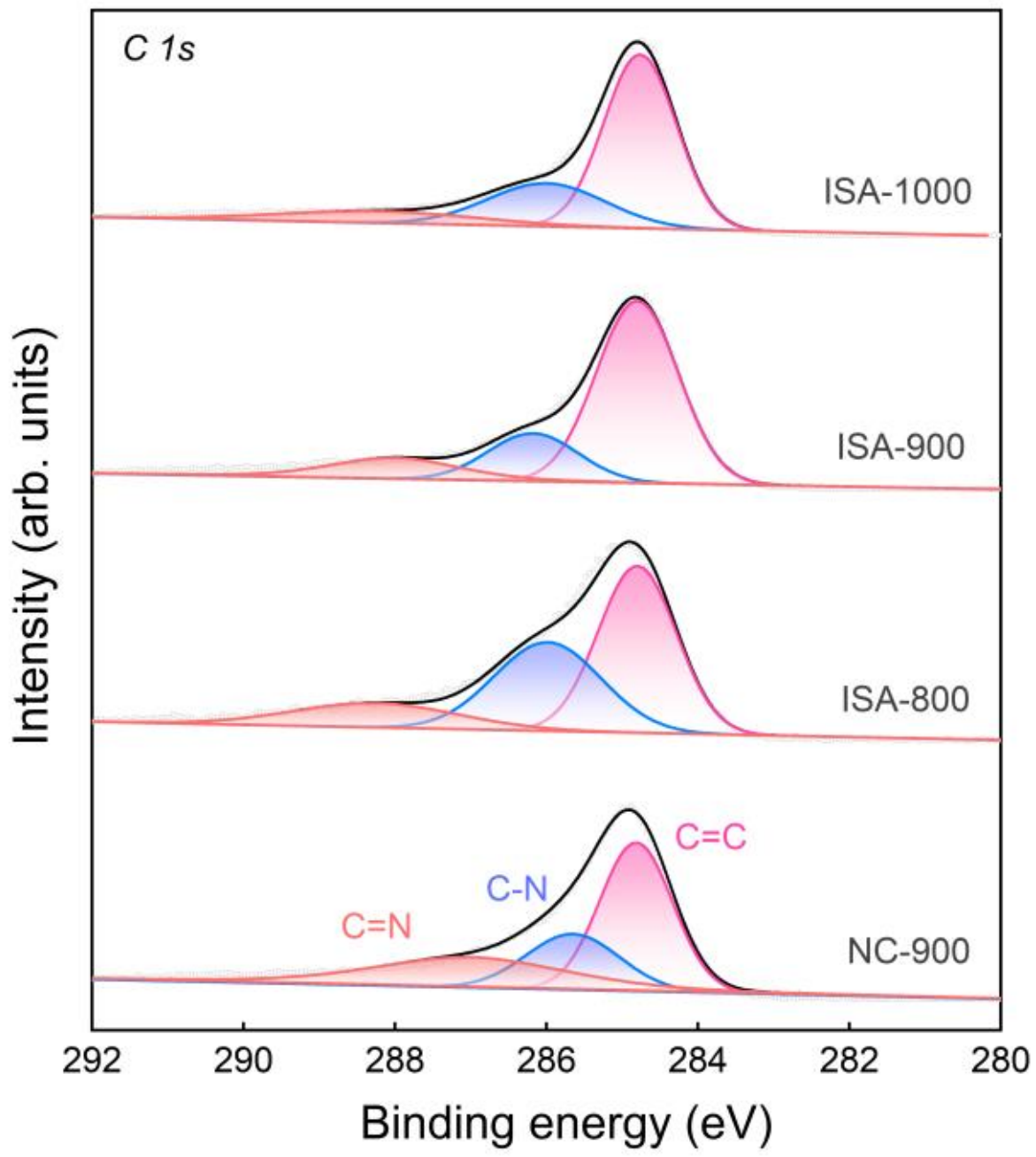

Fig. S8. C 1s XPS spectra. The spectra exhibit three peaks at 284.5 eV, 285.8 eV, and 287.4 eV, corresponding to C=C, C-N, and C=N bonds, respectively. Source data are provided as a Source Data file.

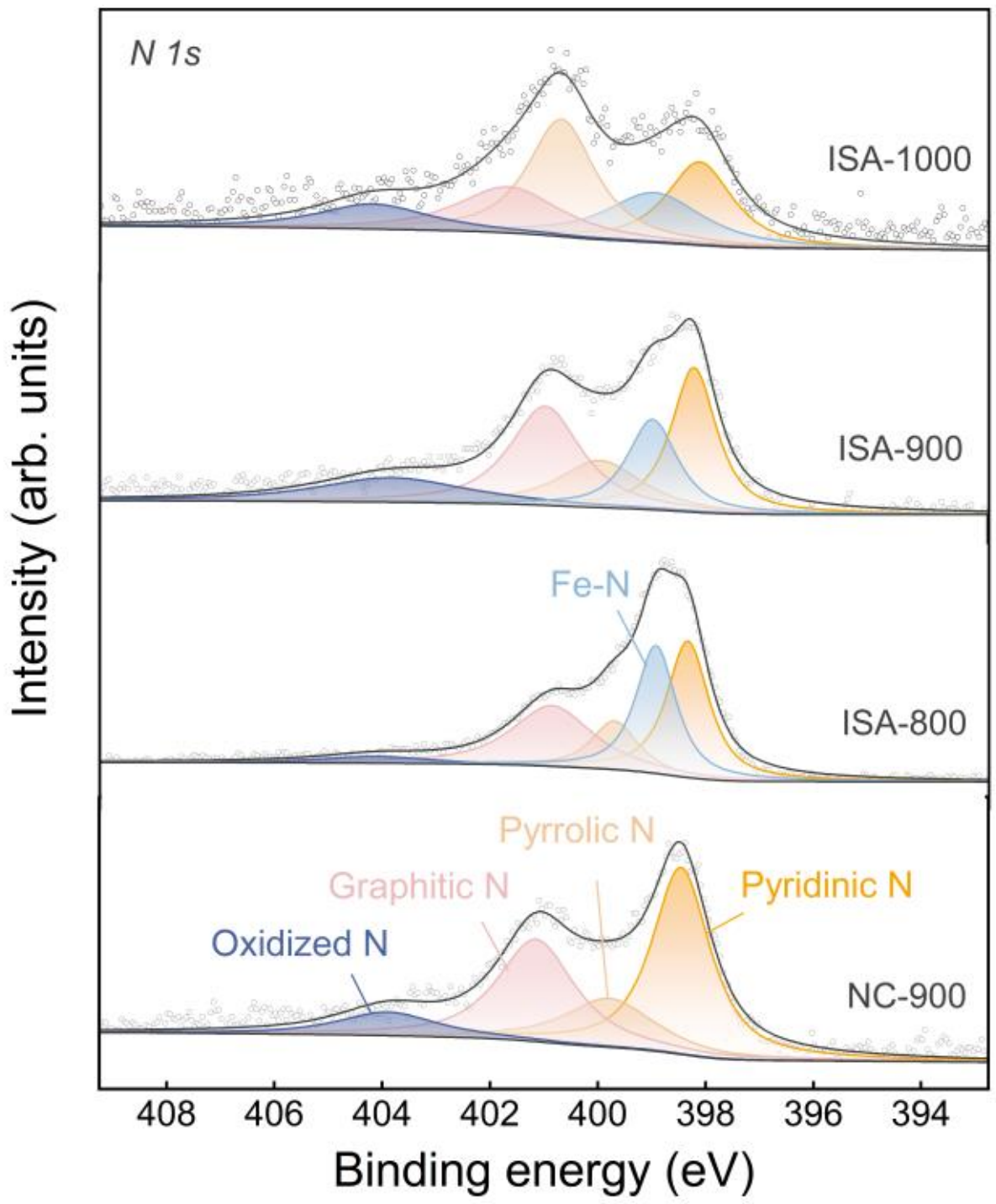

Fig. S9. N 1s XPS spectra. Five different types of N species: pyridinic N (398.33 eV), Fe-N (398.98 eV), pyrrolic N (399.98 eV), graphitic N (400.98 eV), and oxidized N (403.88 eV). Source data are provided as a Source Data file.

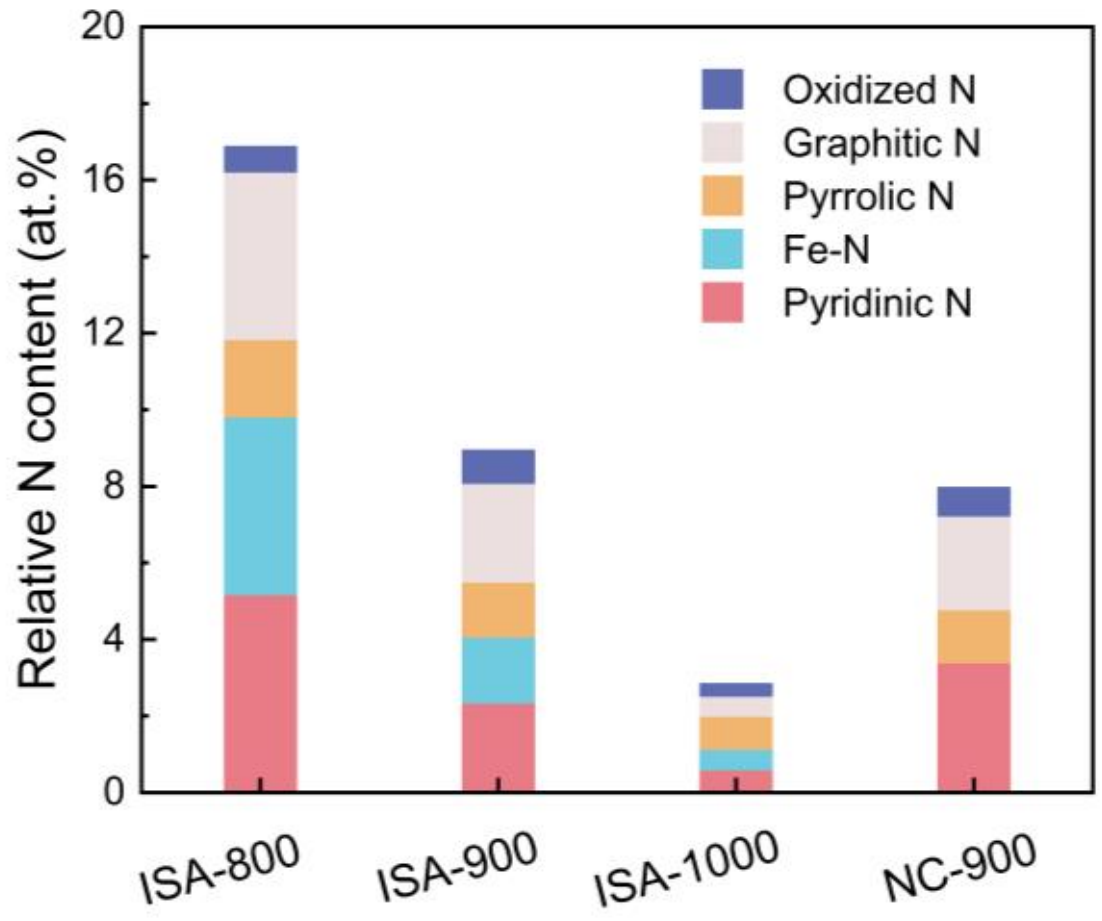

Fig. S10. The contents of different kinds of N. Source data are provided as a Source Data file.

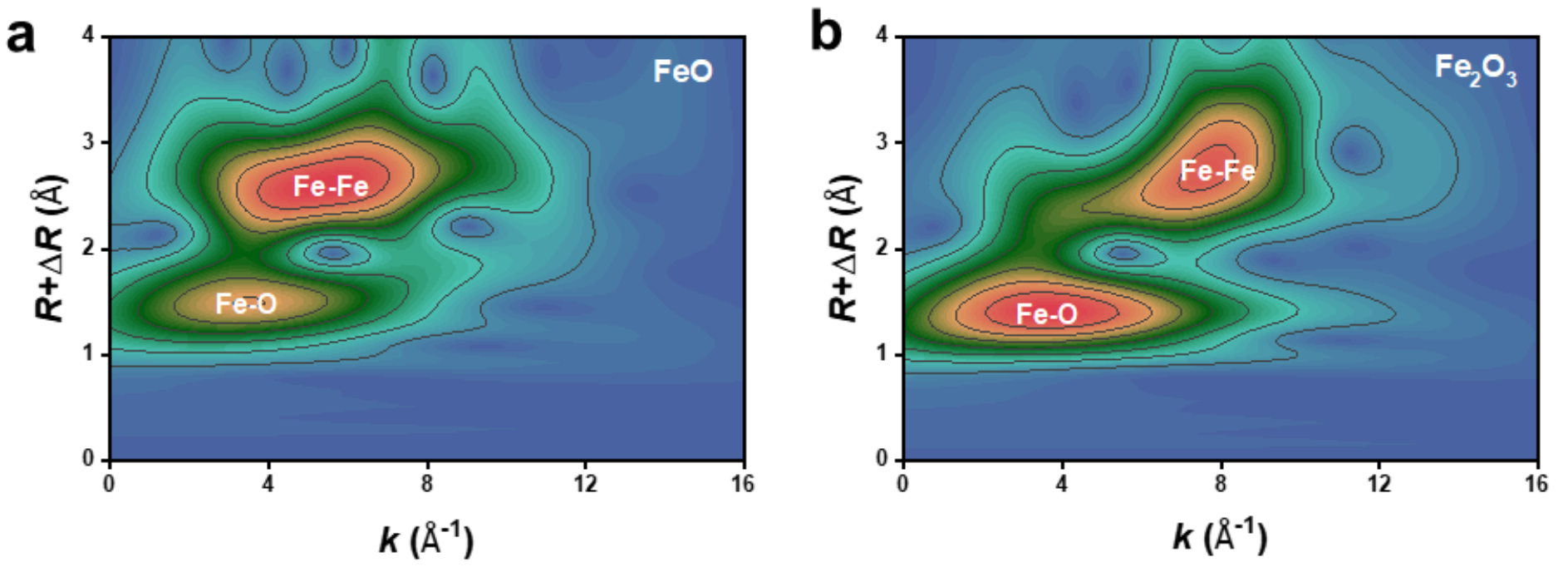

Fig. S11. WT-EXAFS spectra of FeO (a) and Fe_2O_3 (b). Source data are provided as a Source Data file.

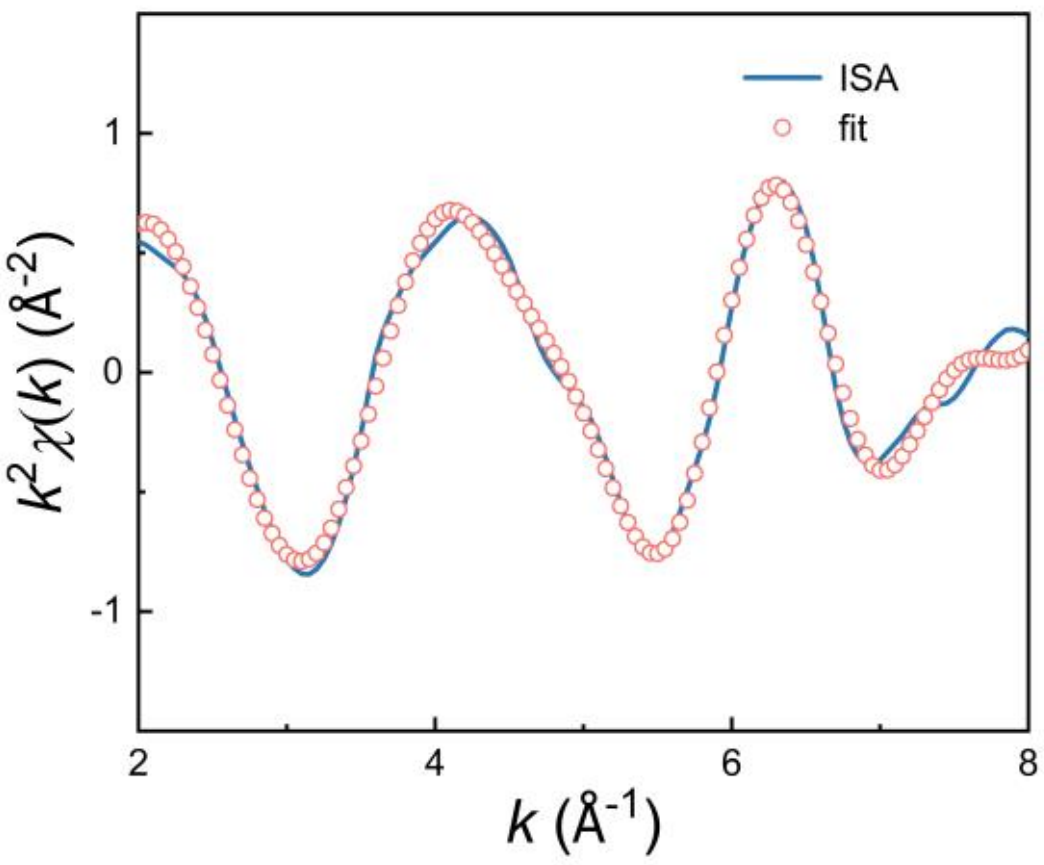

Fig. S12. The corresponding extended X-ray absorption fine structure (EXAFS) *k* space fitting curves of ISA. Source data are provided as a Source Data file.

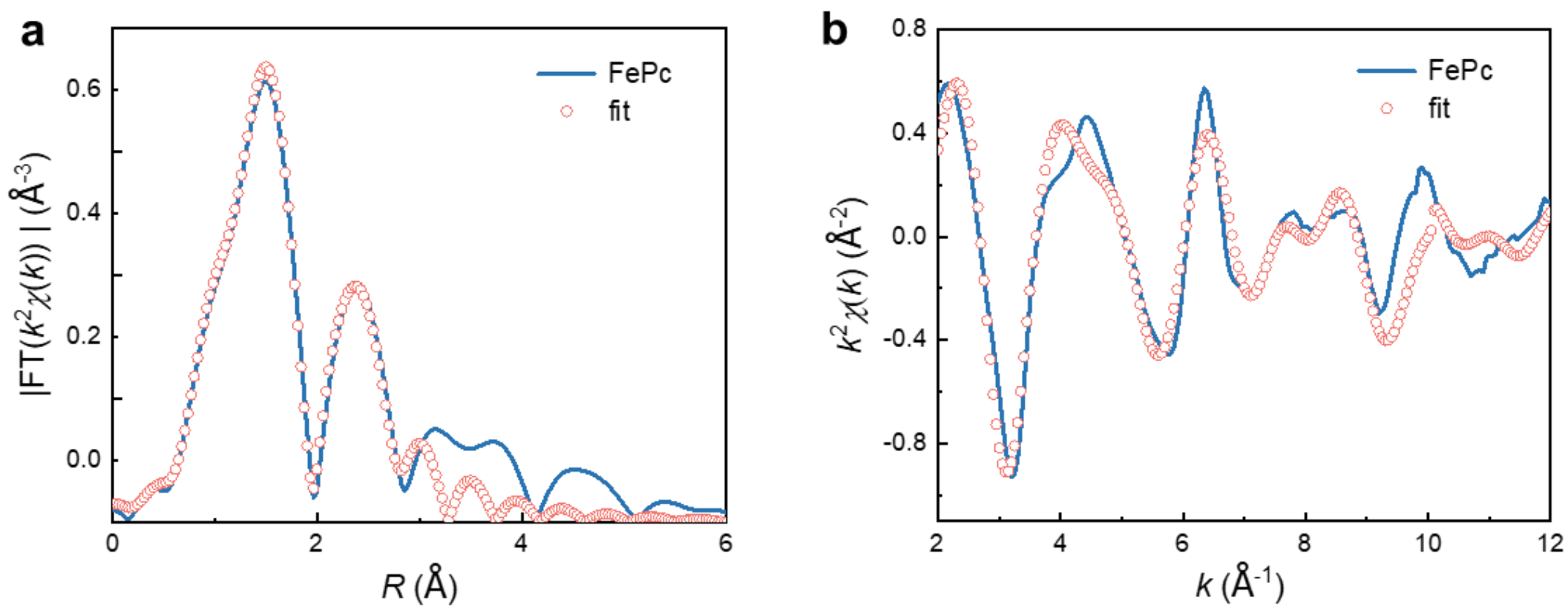

Fig. S13. The corresponding EXAFS R (a) and k (b) space fitting curves of FePc. Source data are provided as a Source Data file.

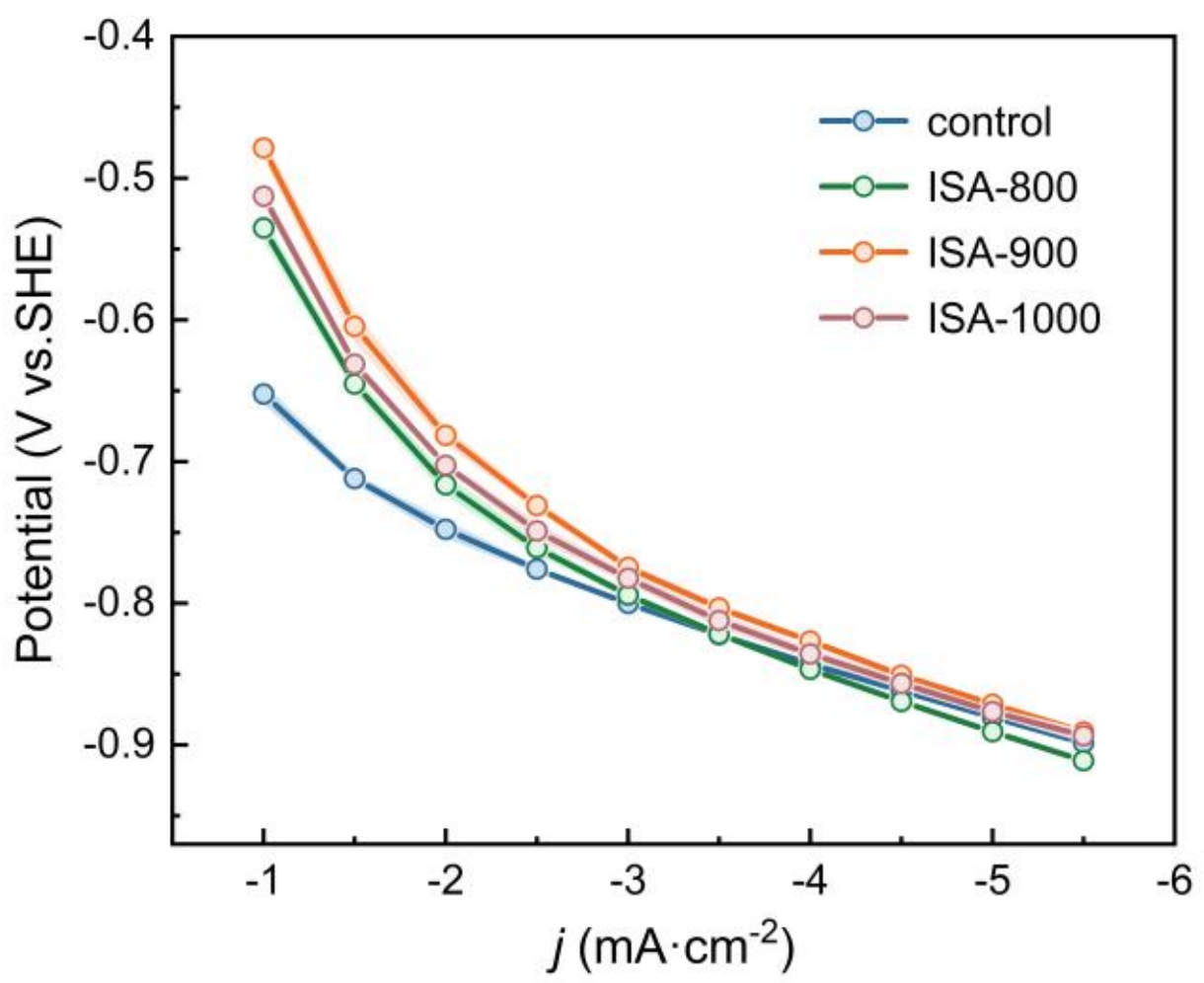

Fig. S14. V-*j* curves of cathode with different kinds of ISA (12.5 mg L^{-1}) addition. The potential is not *iR* corrected. Source data are provided as a Source Data file.

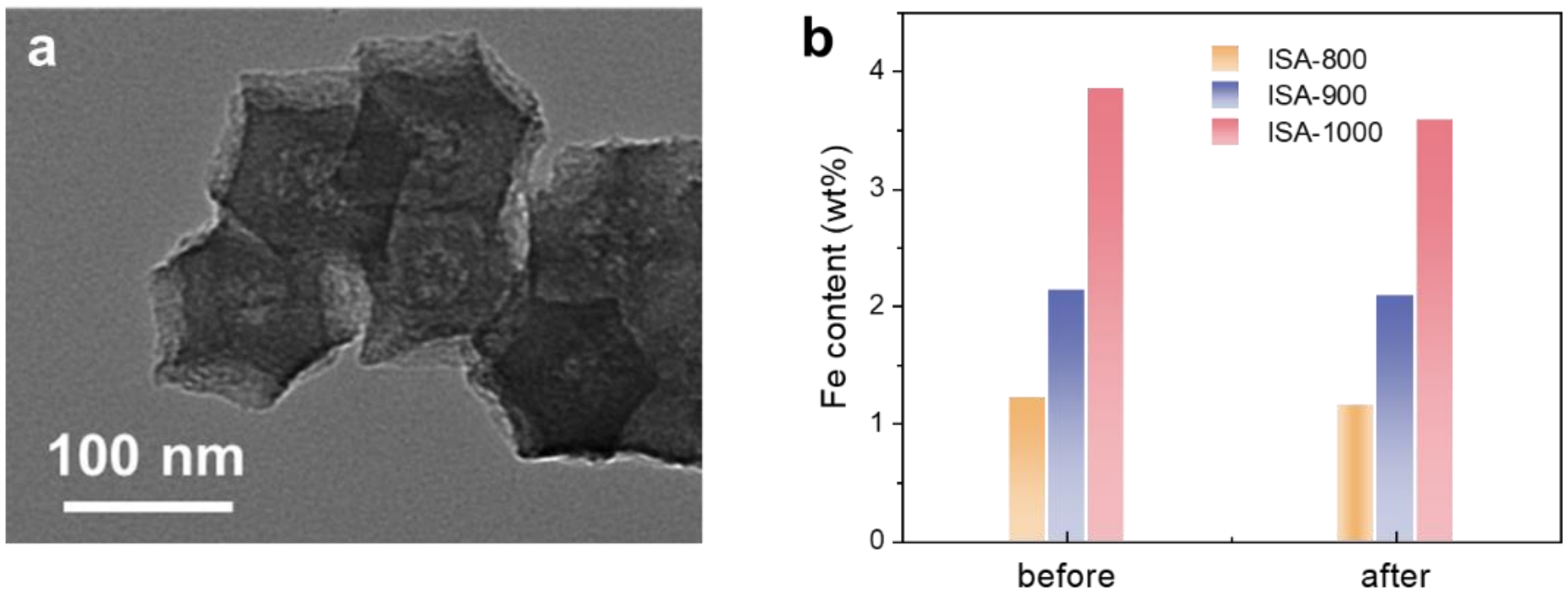

Fig. S15. (a) TEM image of ISA after operation. (b) The Fe content of ISA before and after operation. Source data for b are provided as a Source Data file.

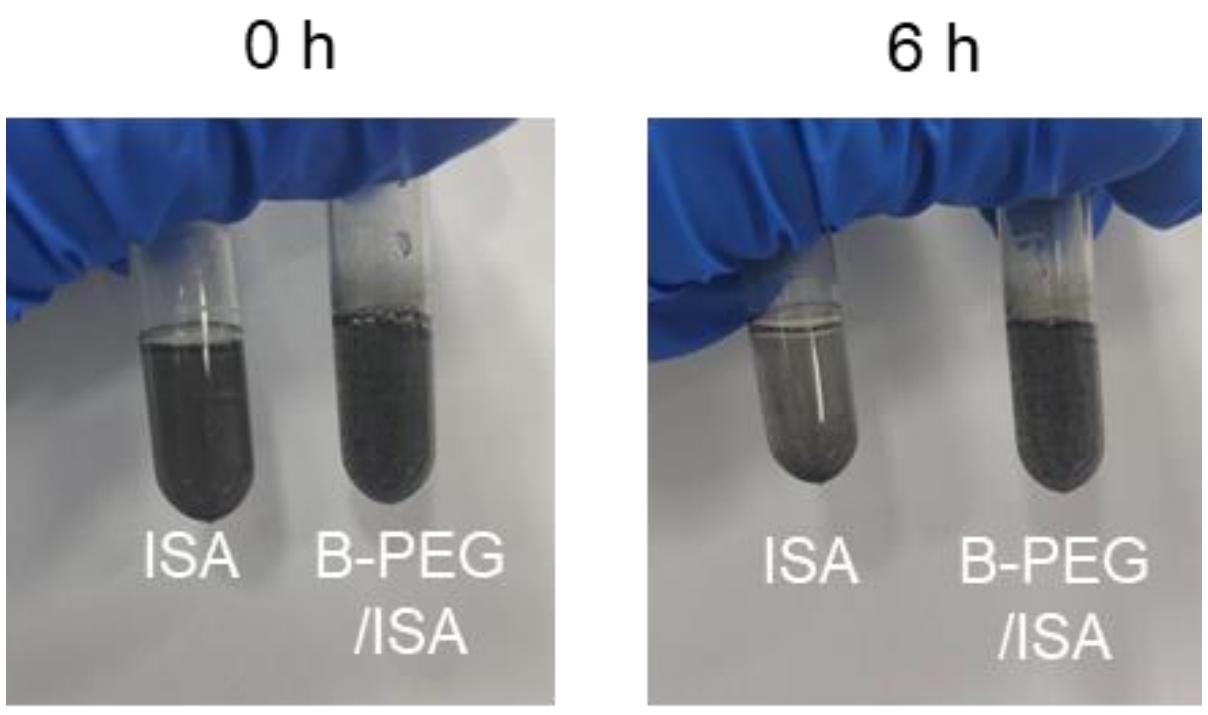

Fig. S16. Stability of ISA and B-PEG/ISA. Photographs of ISA and B-PEG/ISA with a concentration of 2 mg mL^{-1} in PBS for 0 h (left) and 6 h (right).

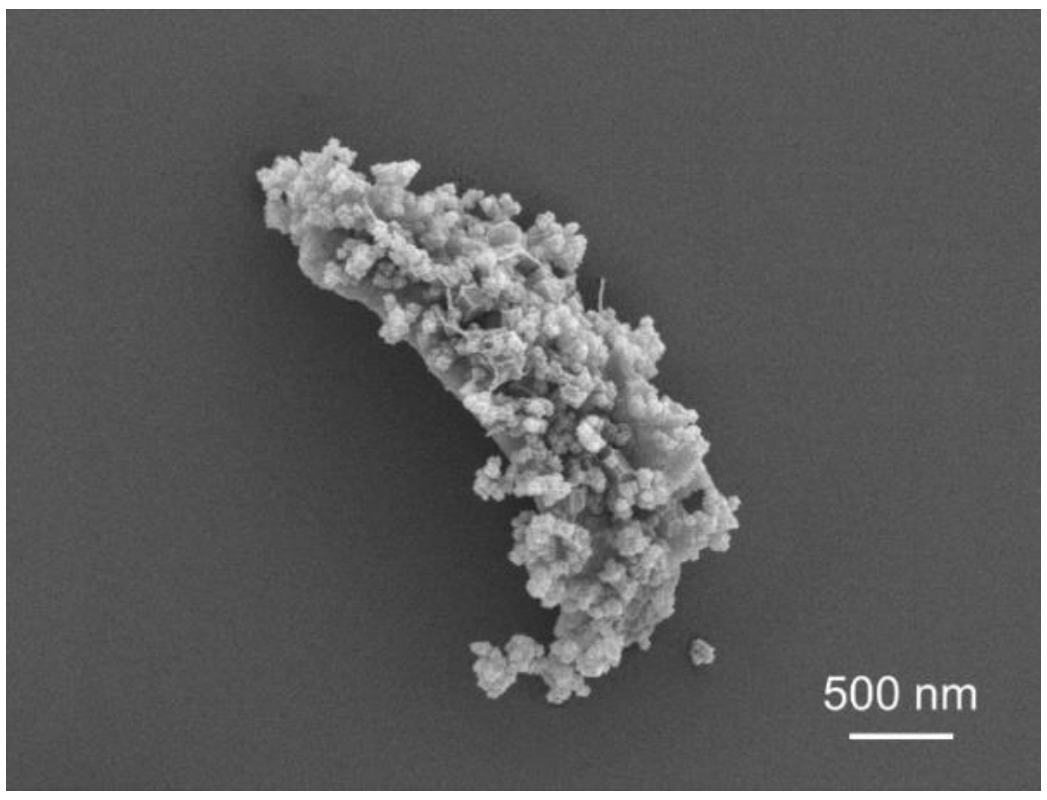

Fig. S17. SEM image of *C.N*@ISA.

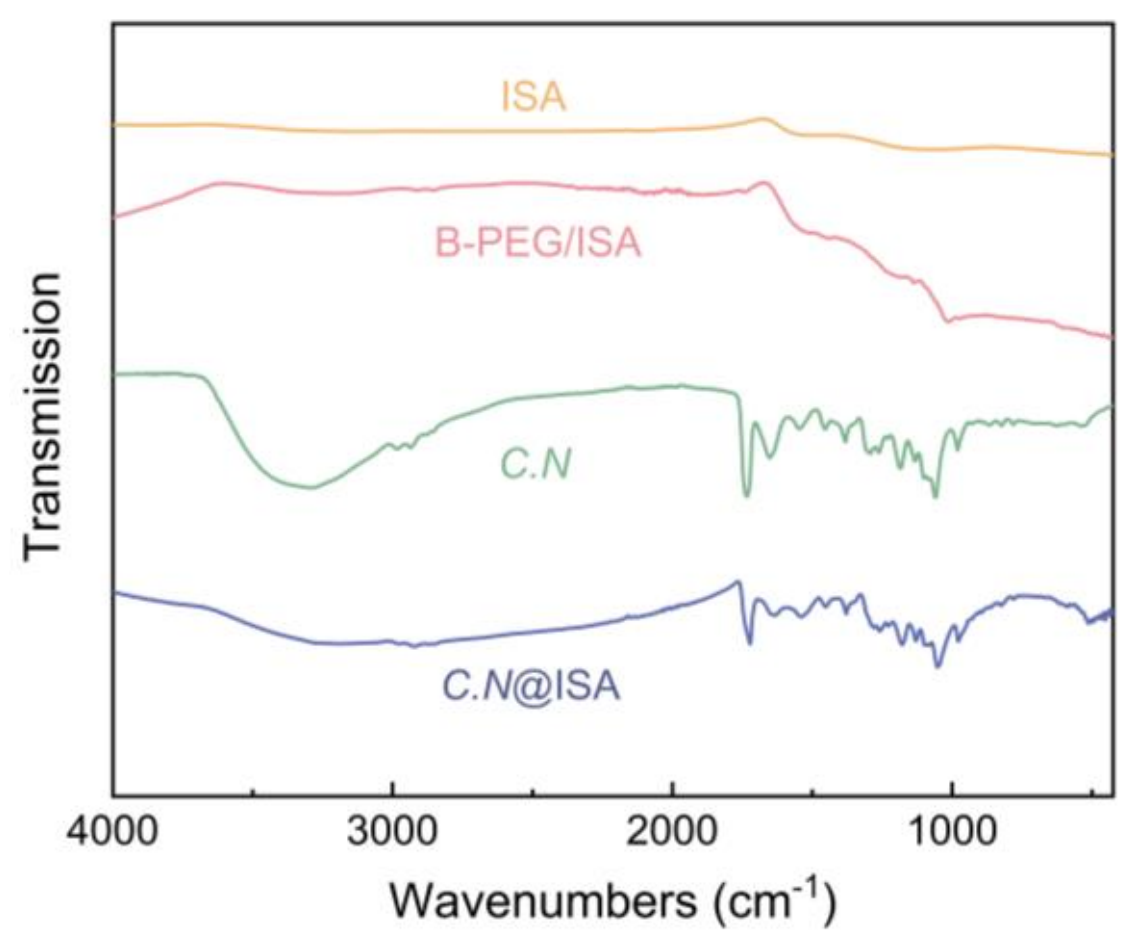

Fig. S18. FTIR spectra of ISA, B-PEG/ISA, *C.N* and *C.N@ISA*. Source data are provided as a Source Data file.

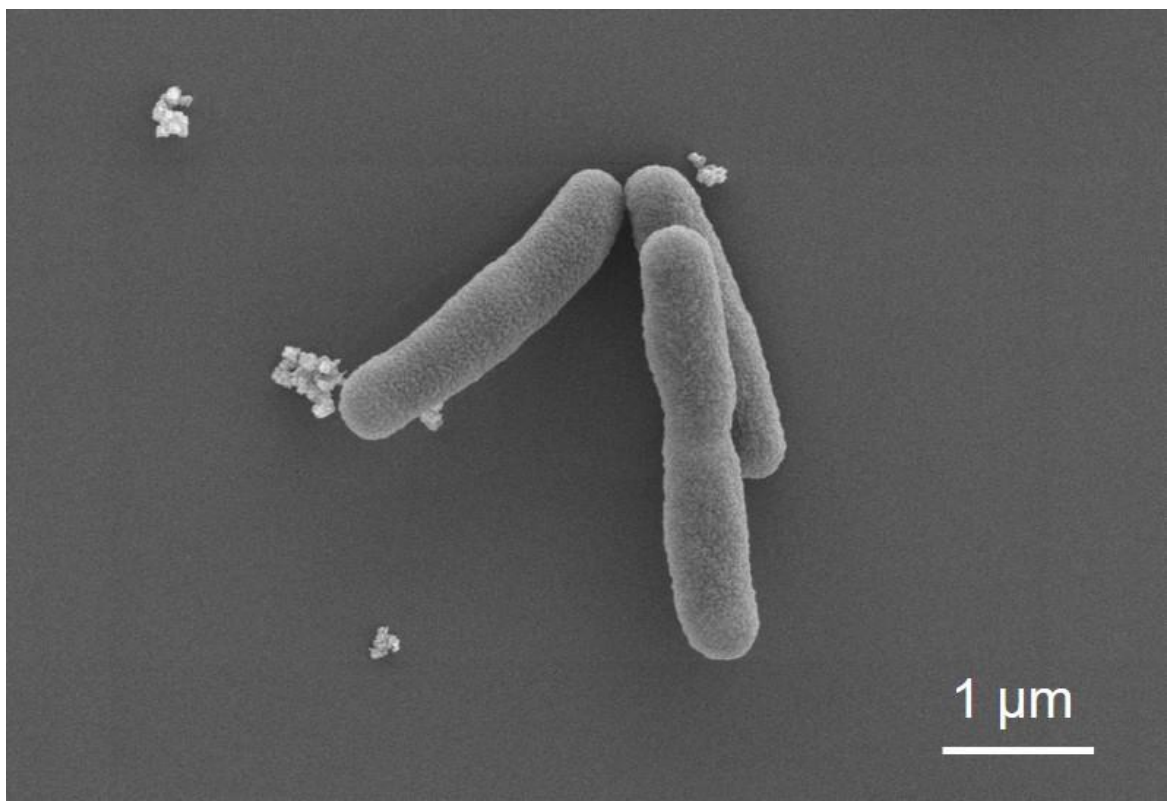

Fig. S19. SEM image of cells after the fructose competitive binding assay. The binding sites of B-PEG/ISA are occupied by D-fructose.

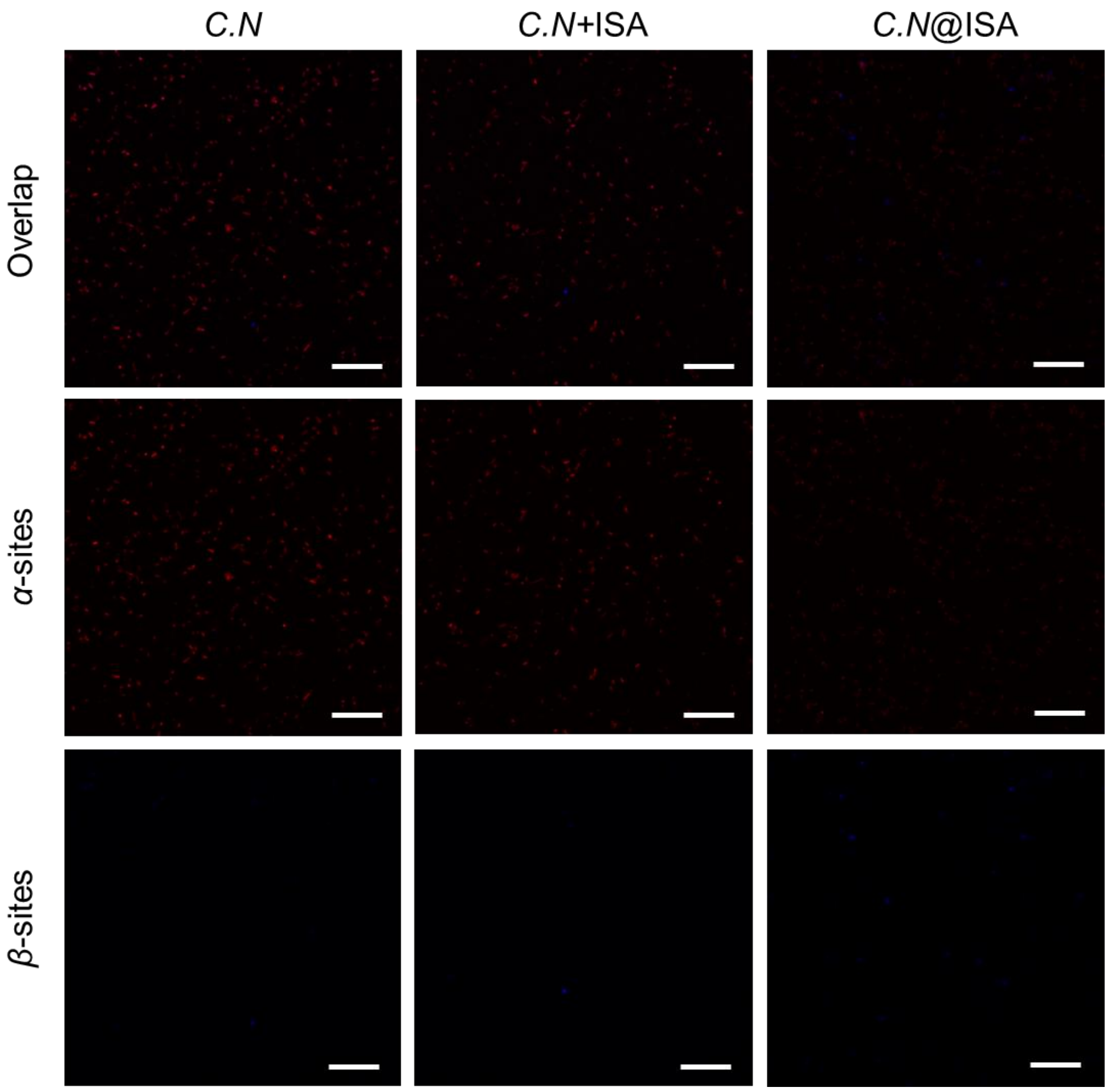

Fig. S20. CLSM images of cells stained for membrane surface polysaccharides.

Scale bar: 20 μm.

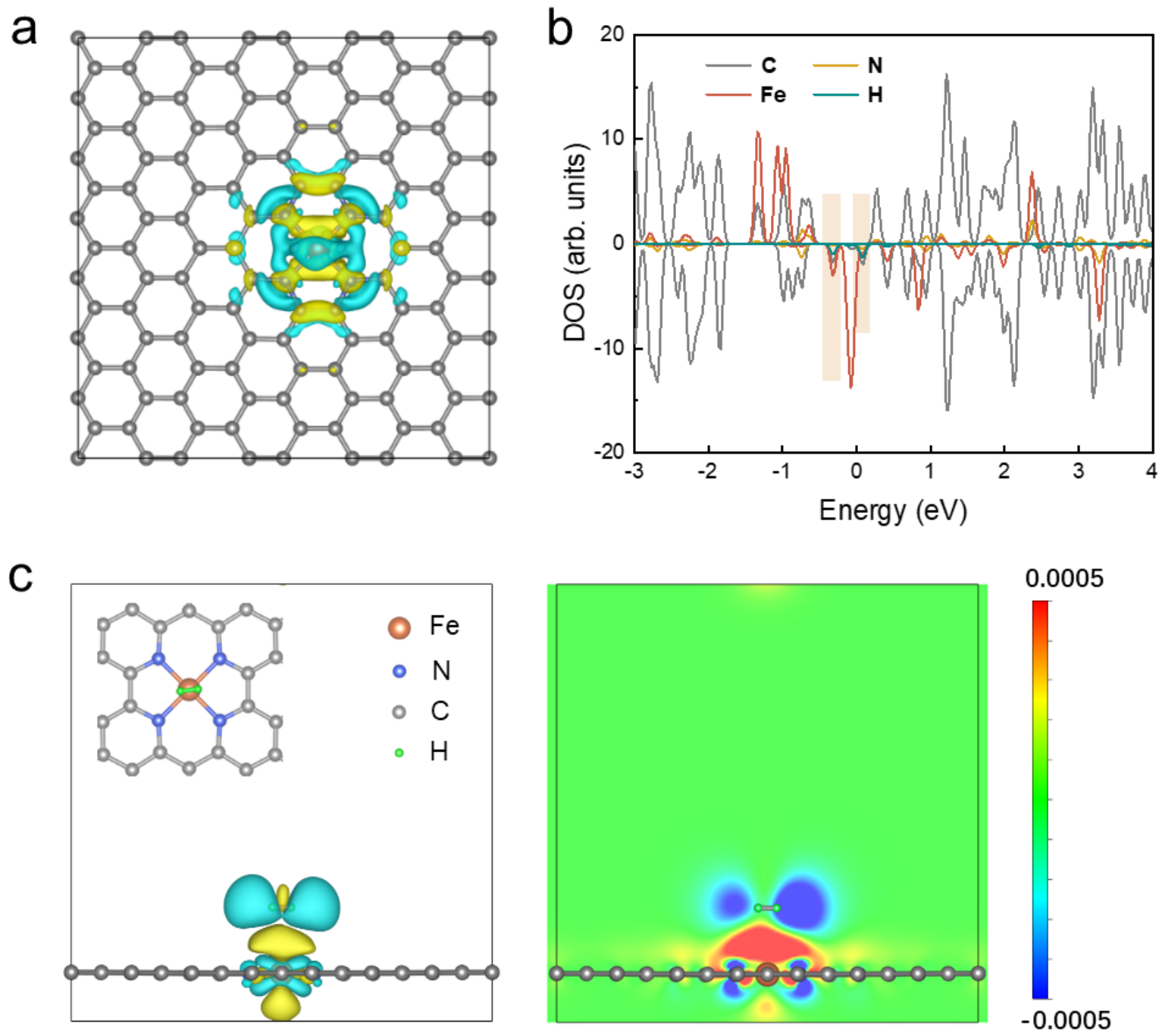

Fig. S21. (a) Differential charge density diagram of Fe-N4pyd-C. (b) DOS of the substrate (Fe-N4pyd-C) after H_2 adsorption. (c) Differential charge density diagram of Fe-N4pyd-C with 1-H_2 molecule. Source data are provided as a Source Data file.

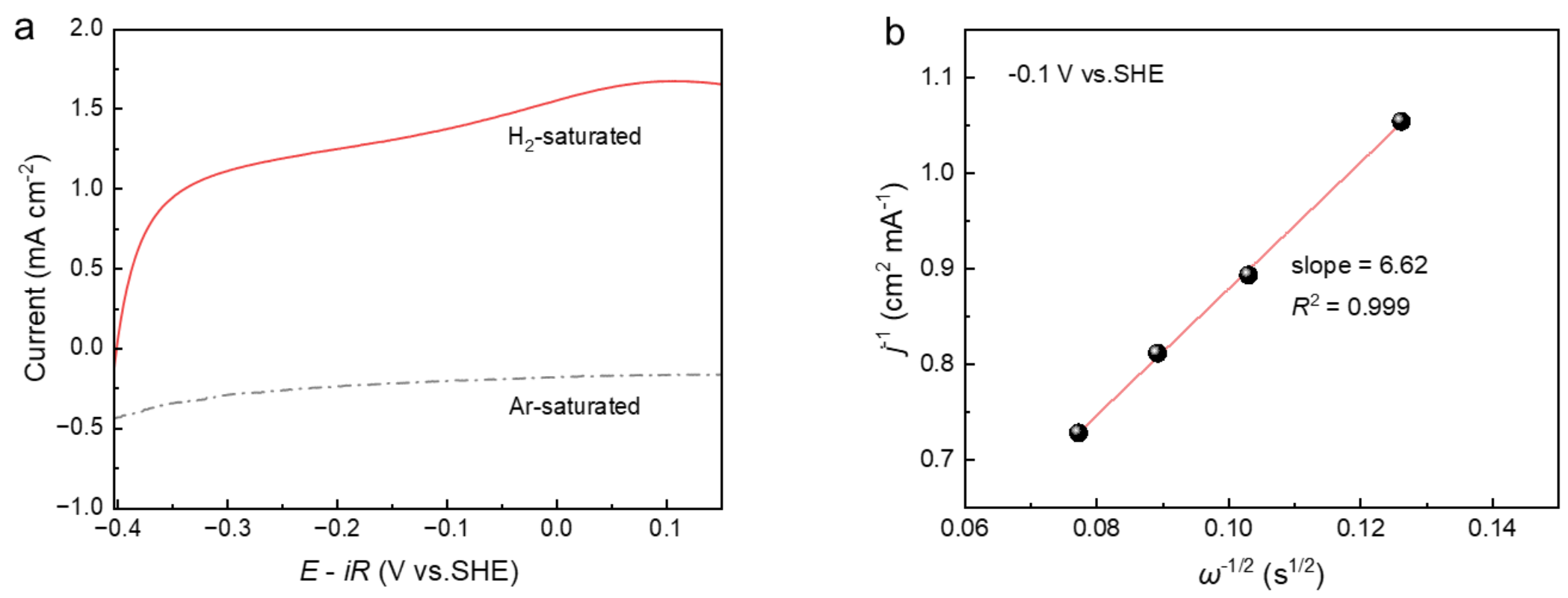

Fig. S22. (a) Polarization curves of ISA acquired in H_2 and Ar-saturated electrolyte. Rotation speed: 1600 rpm. The x-axis shows the potential after 85% *iR* correction. The electrode surface area is 0.1256 cm^2 and the electrolyte is a neutral 0.1 M PBS (pH = 7.0 ± 0.04). The solution resistance for Ar-saturated group: 10.77 ± 0.04 Ω. (b) The corresponding Koutecký-Levich plot at -0.1 V vs.SHE (after 85% *iR* correction). Source data are provided as a Source Data file.

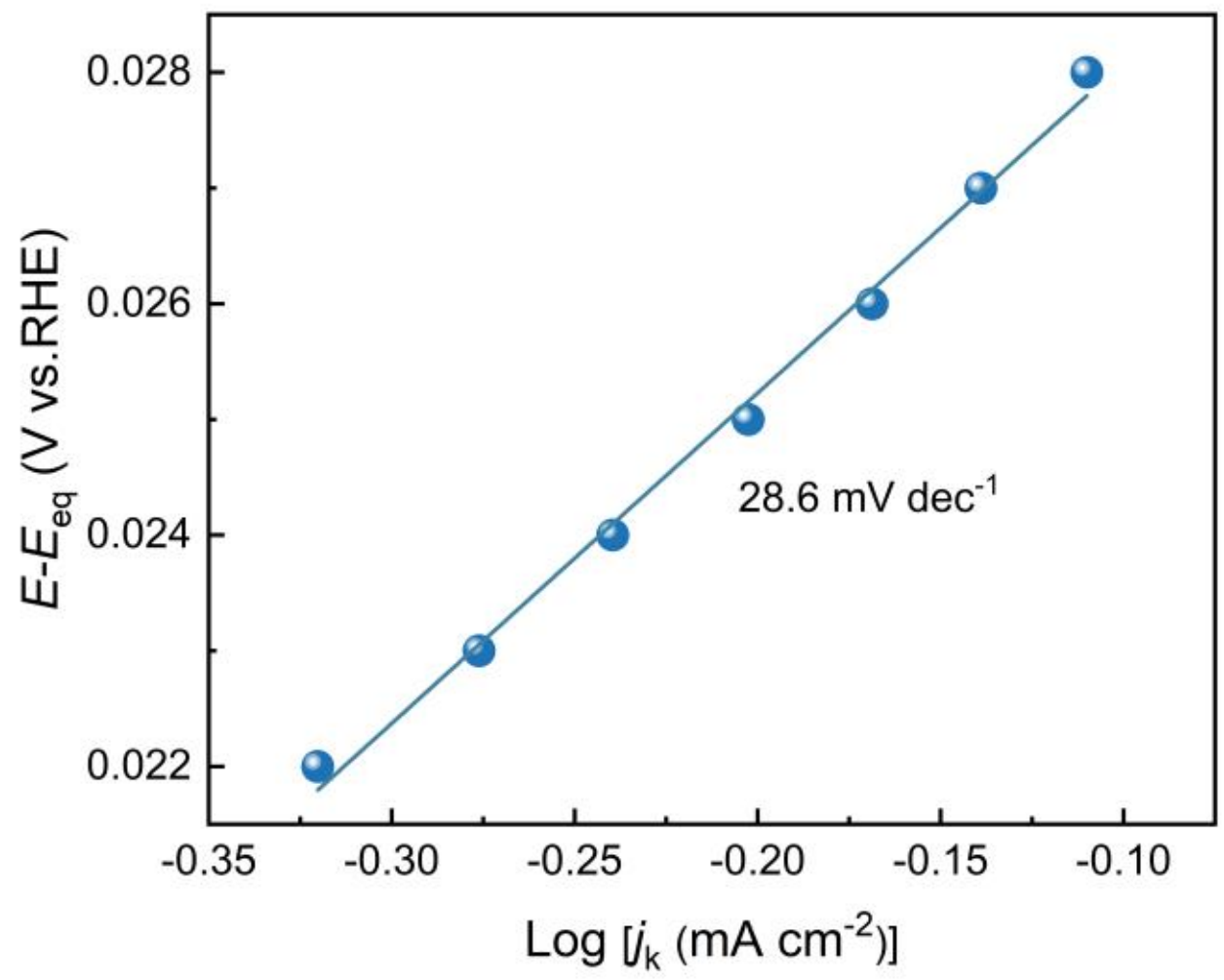

Fig. S23. The corresponding Tafel plot of kinetic current densities for ISA from Fig.4b. Source data are provided as a Source Data file.

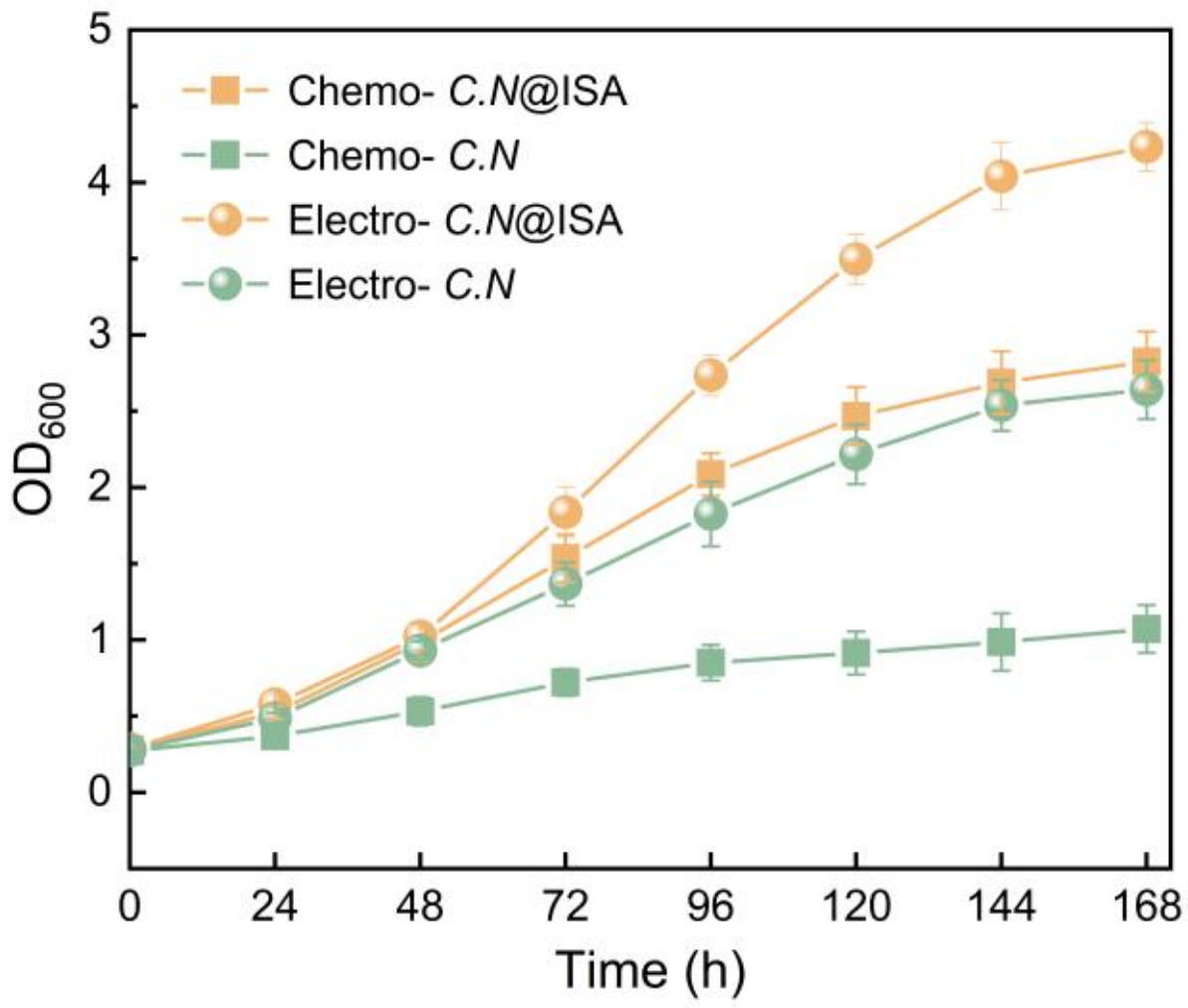

Fig. S24. Time-dependent OD_{600} values in *C. necator* cultures under electroautotrophic and chemoautotrophic conditions. Data are presented as mean ± s.d. from n = 3 independent experiments. Source data are provided as a Source Data file.

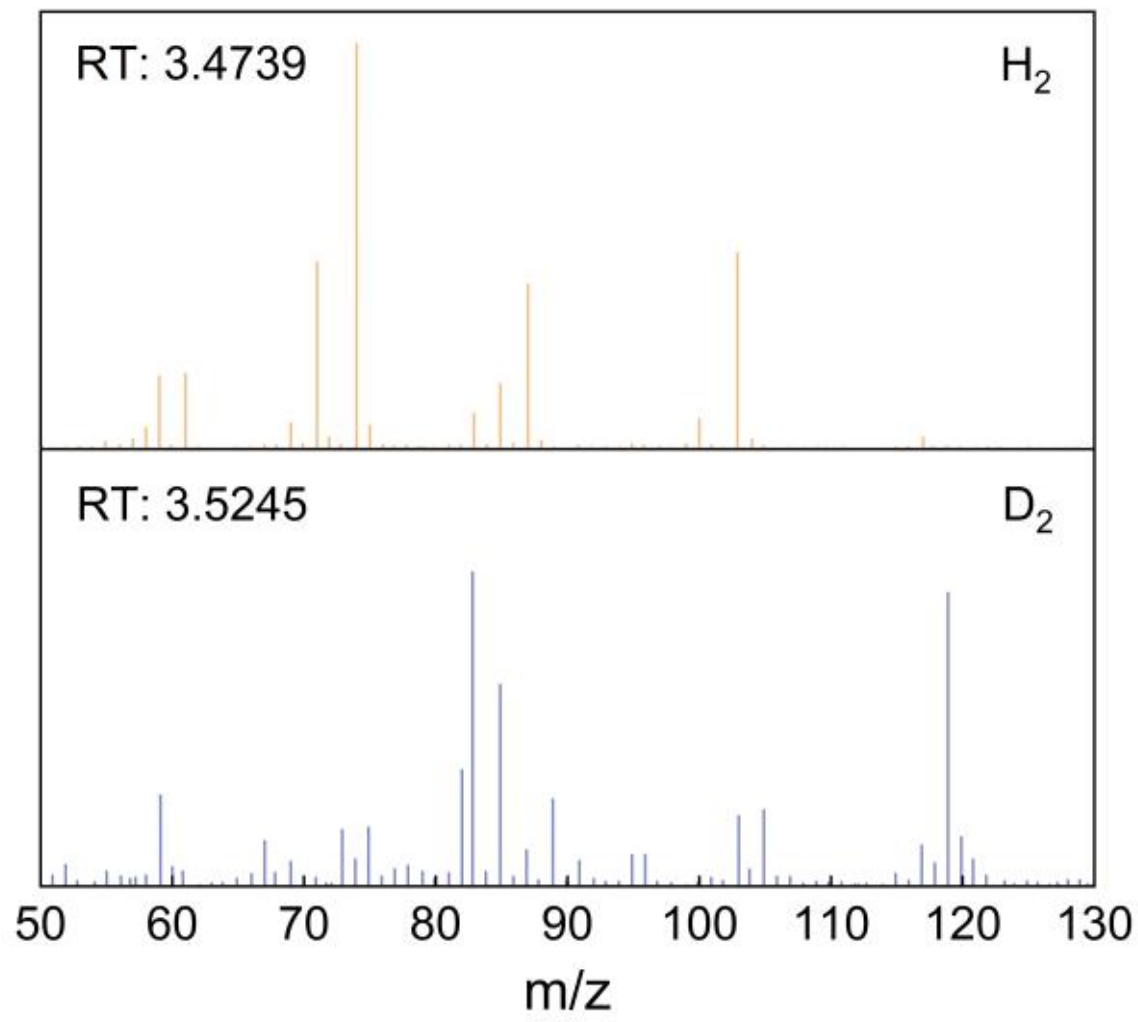

Fig. S25. Comparison of GC–MS spectra of PHB extracted from *C.N*@ISA cultivated under H_2 (top) and D_2 (bottom) atmospheres. Source data are provided as a Source Data file.

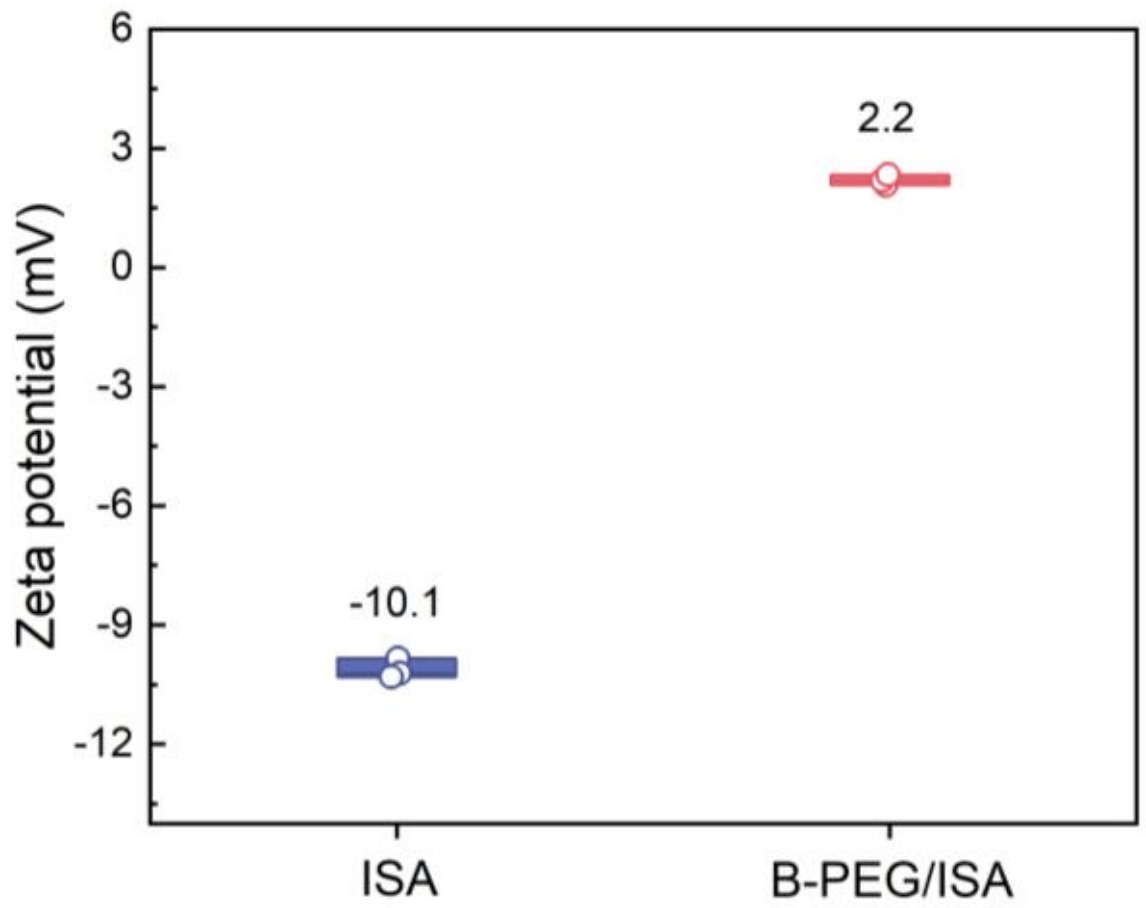

Fig. S26. Zeta potential. Data are presented as mean ± s. d. from n = 3 independent experiments. Source data are provided as a Source Data file.

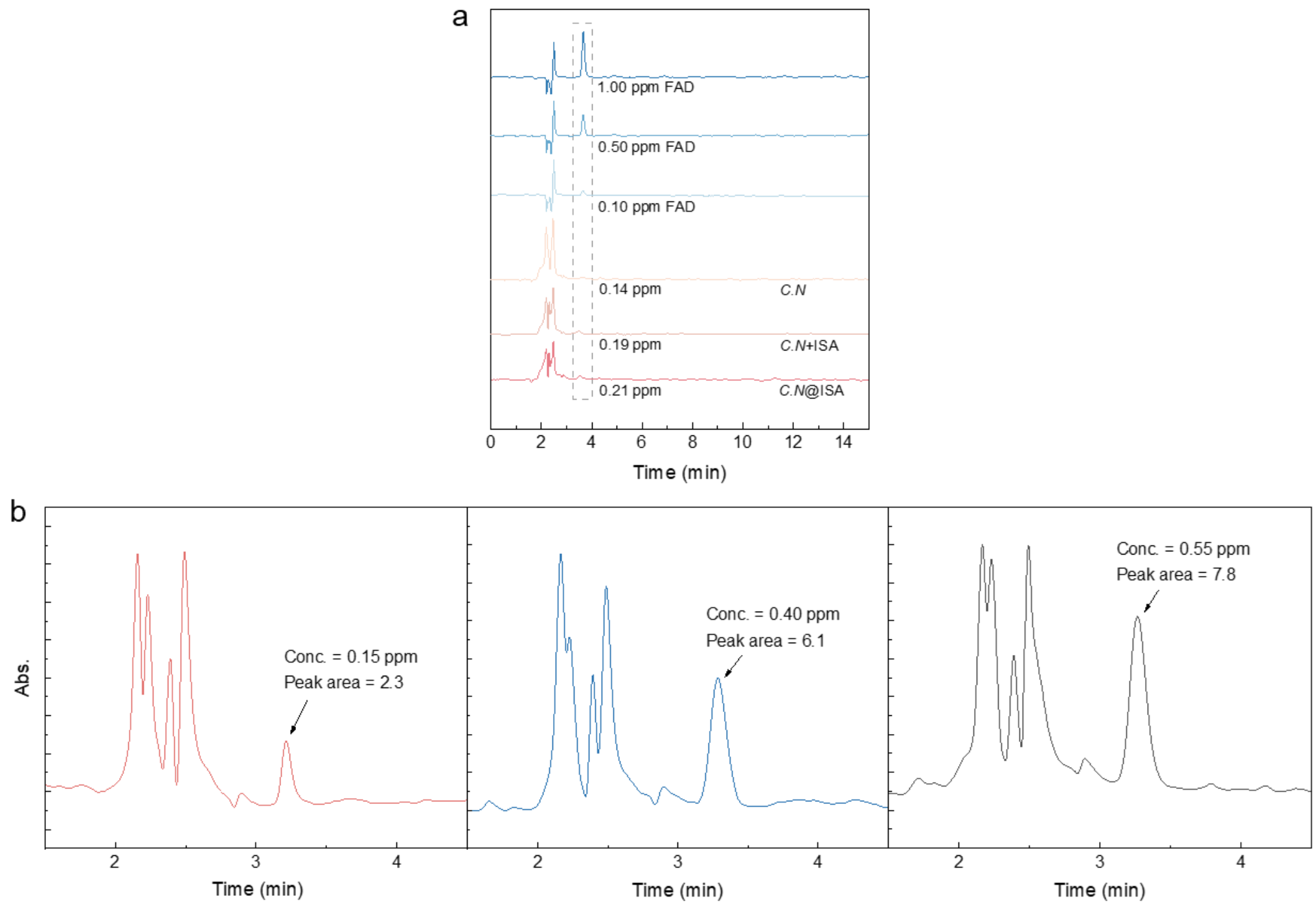

Fig. S27. (a) Liquid chromatographic peak signal and calibration curve of FAD. (b) Standard-addition confirmation of FAD peak assignment. HPLC chromatograms of the biohybrid sample (left, 0.15 ppm endogenous FAD) and the same sample spiked with FAD standard to total concentrations of 0.40 ppm (middle) and 0.55 ppm (right). The target peak (~3.2 min, arrows) increases proportionally with the spike amount (R^2 = 0.998) while maintaining the same retention time, confirming its assignment to FAD. Peaks at shorter retention times (~2.0–2.5 min) correspond to background medium components. Source data are provided as a Source Data file.

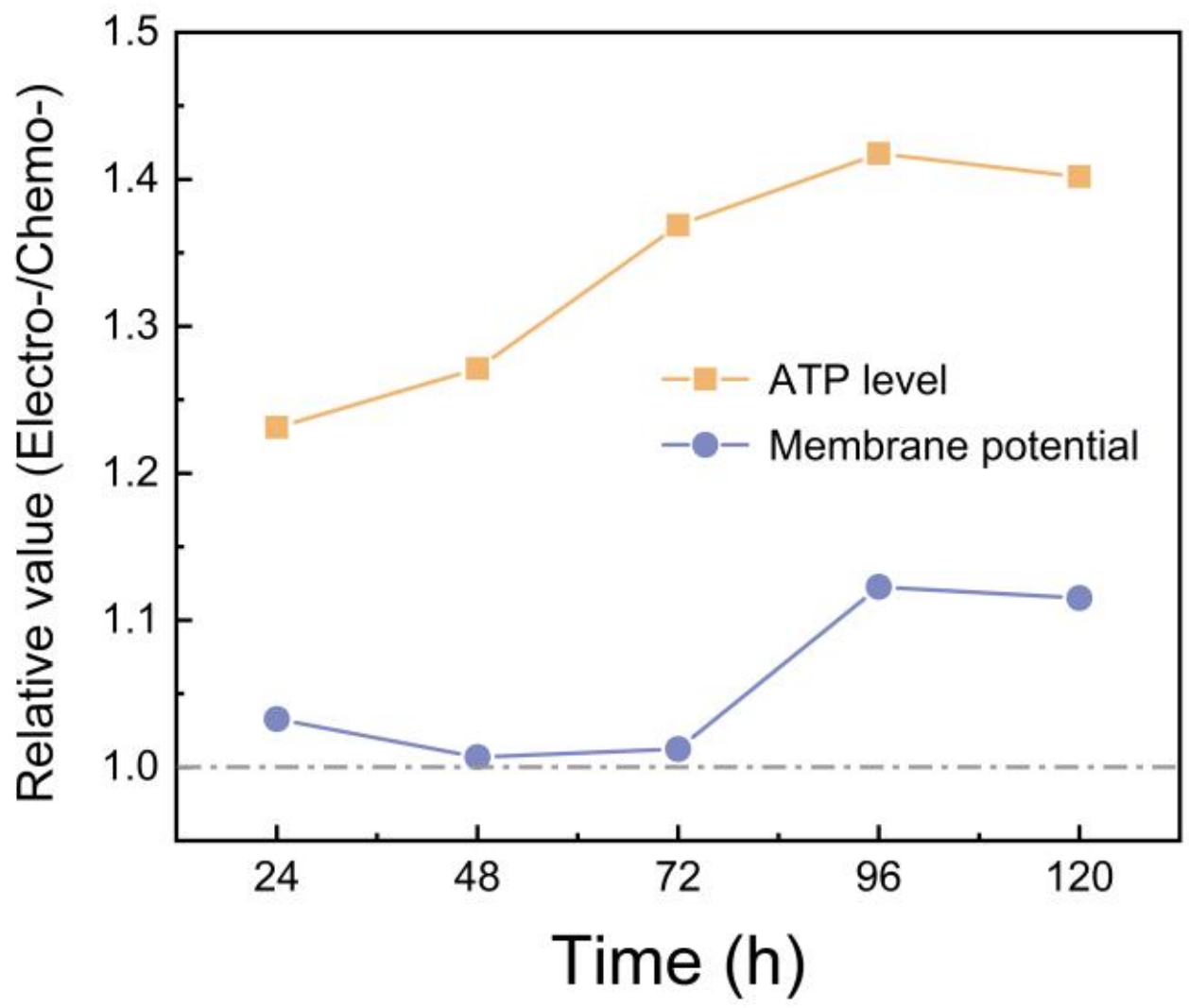

Fig. S28. ATP levels and PMF (as indicated by membrane potential) in *C.N*@ISA under electrochemical and direct H_2 bubbling conditions. The y-axis represents the ratio of the measured values under electrochemical conditions to those under direct H_2 bubbling conditions. Source data are provided as a Source Data file.

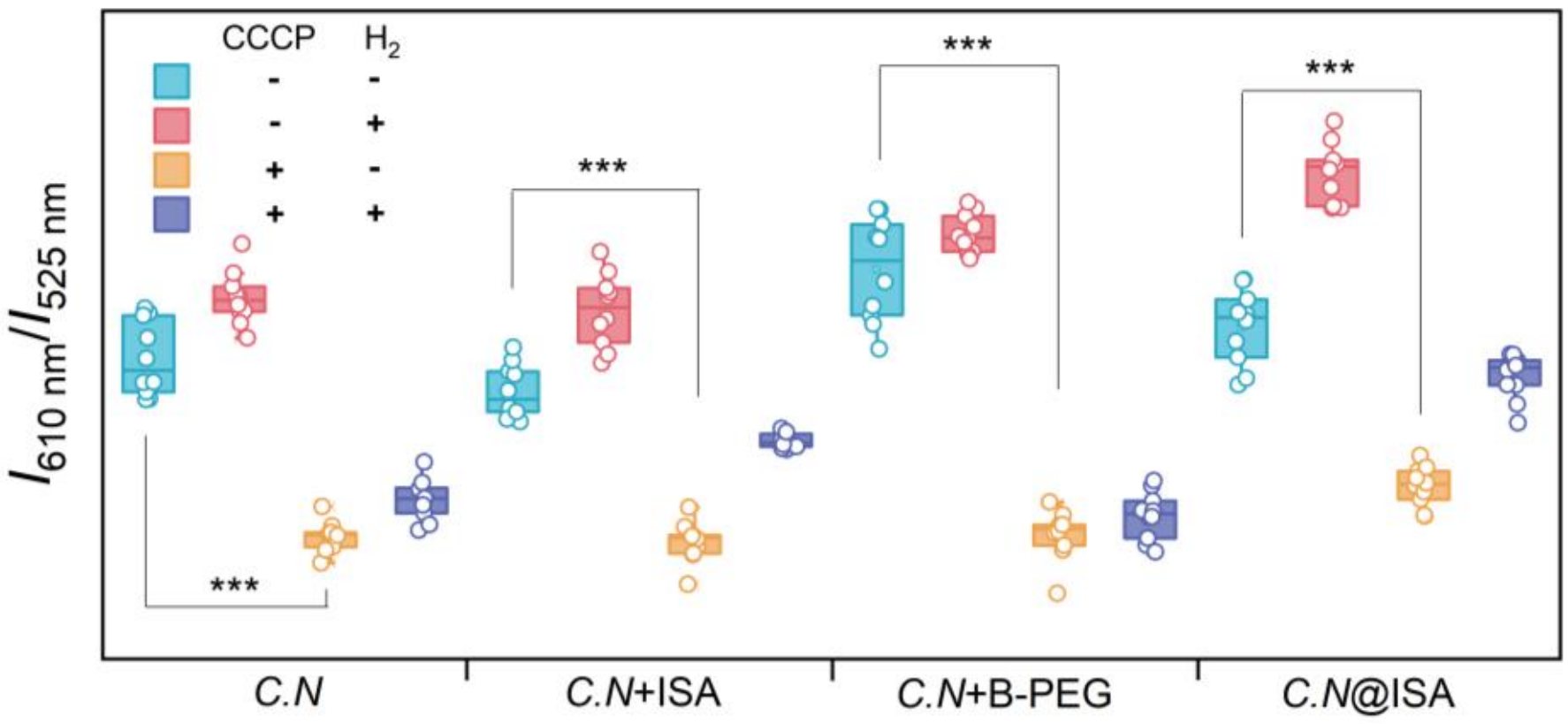

Fig. S29. Membrane potential measured by the ratio of aggregate and monomer fluorescence intensities. Data are presented as mean ± s. d. from n = 10 independent experiments. Source data are provided as a Source Data file.

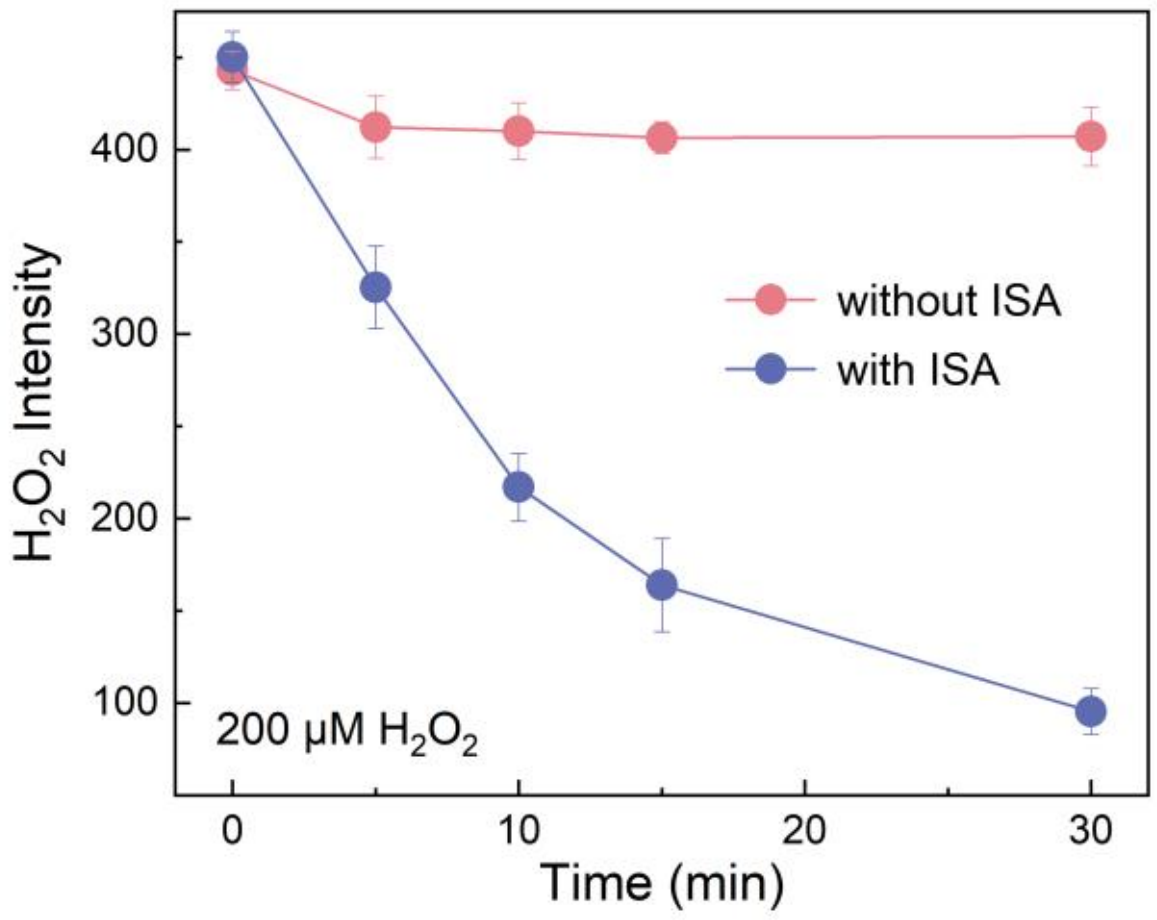

Fig. S30. Effect of ISA on hydrogen peroxide (H_2O_2) scavenging intensity. Data are presented as mean ± s. d. and error bars represent the standard deviation of three independent experiments. Source data are provided as a Source Data file.

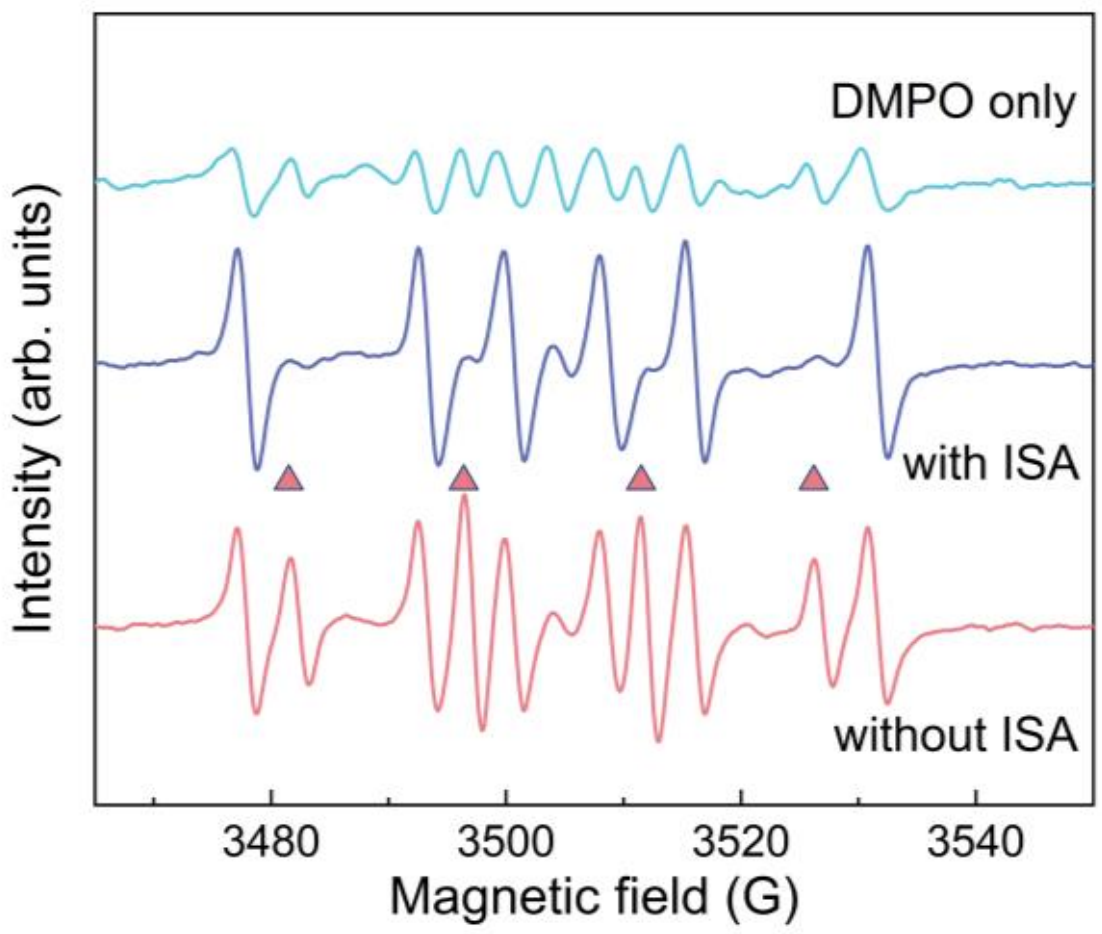

Fig. S31. Effect of ISA on hydroxyl radical (OH·) scavenging determined by EPR spectroscopy. The solid triangle symbol (▲) indicates DMPO-OH•. Source data are provided as a Source Data file.

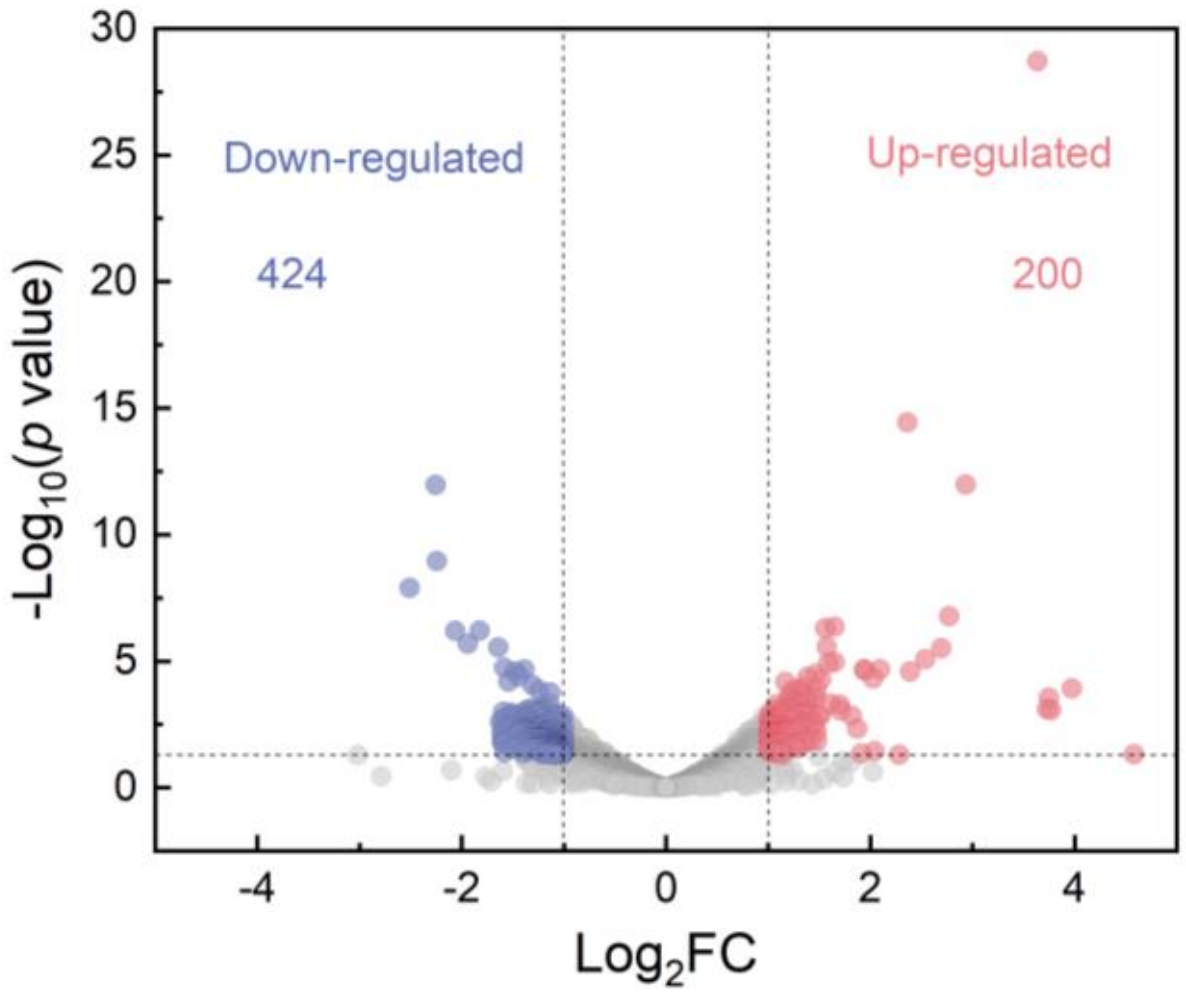

Fig. S32. Volcano plot of the results of the genetic analysis comparing *C.N*@ISA to *C.N* group. FC: Fold change. The two vertical dashed lines represent $Log_2FC = \pm 1$, and the horizontal dashed line corresponds to $-Log_{10}(p \text{ value}) = 1.3$. Source data are provided as a Source Data file.

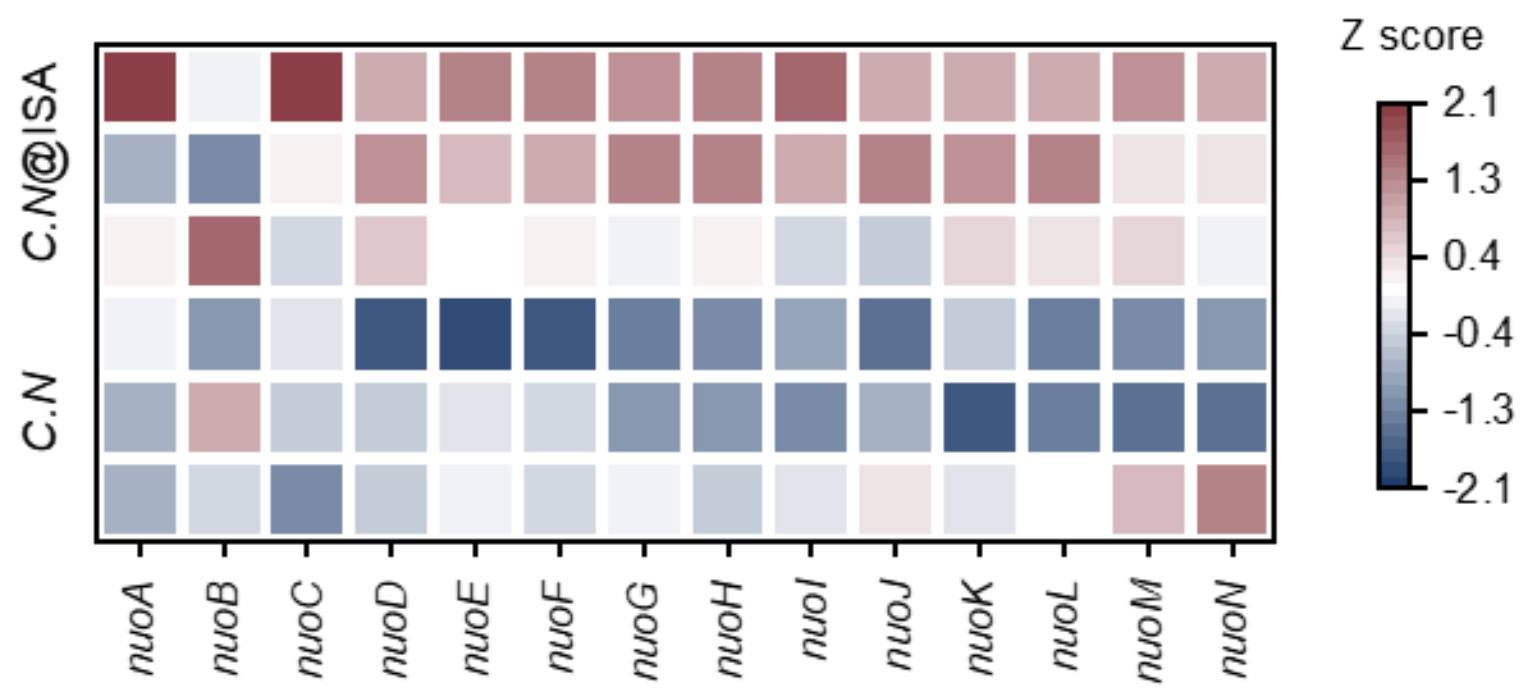

Fig. S33. The expression of genes related to NADH dehydrogenase(*nuoABCDEFGHIJKLMN*). The scale bar represents the Z score of gene expression levels across samples (*C.N* and *C.N*@ISA) and groups (n = 3 independent experiments). Z score is expressed as standardized deviations from their means. Source data are provided as a Source Data file.

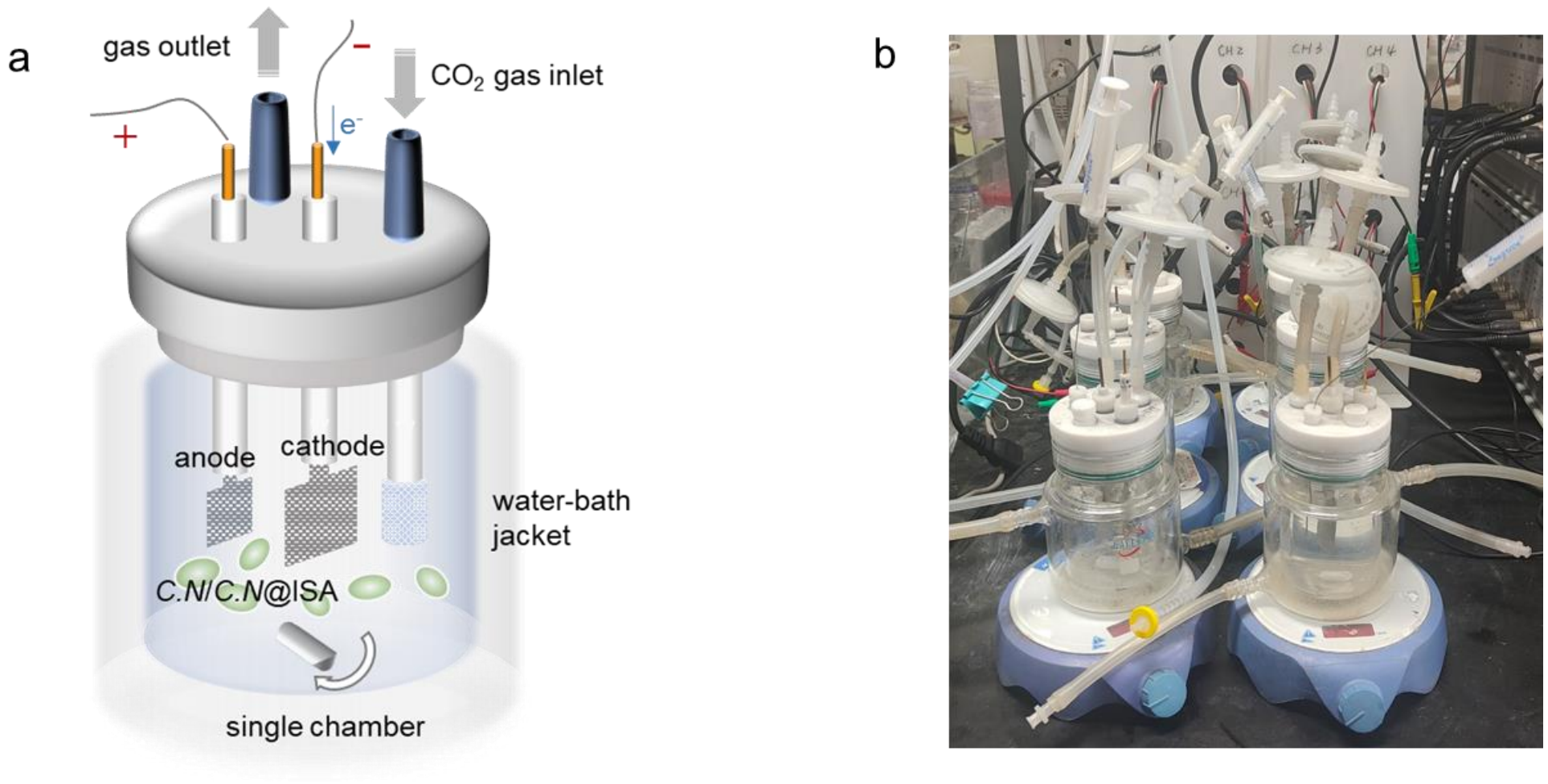

Fig. S34. The integrated inorganic-biological system. (a) Schematic diagram. (b) Photograph of the device.

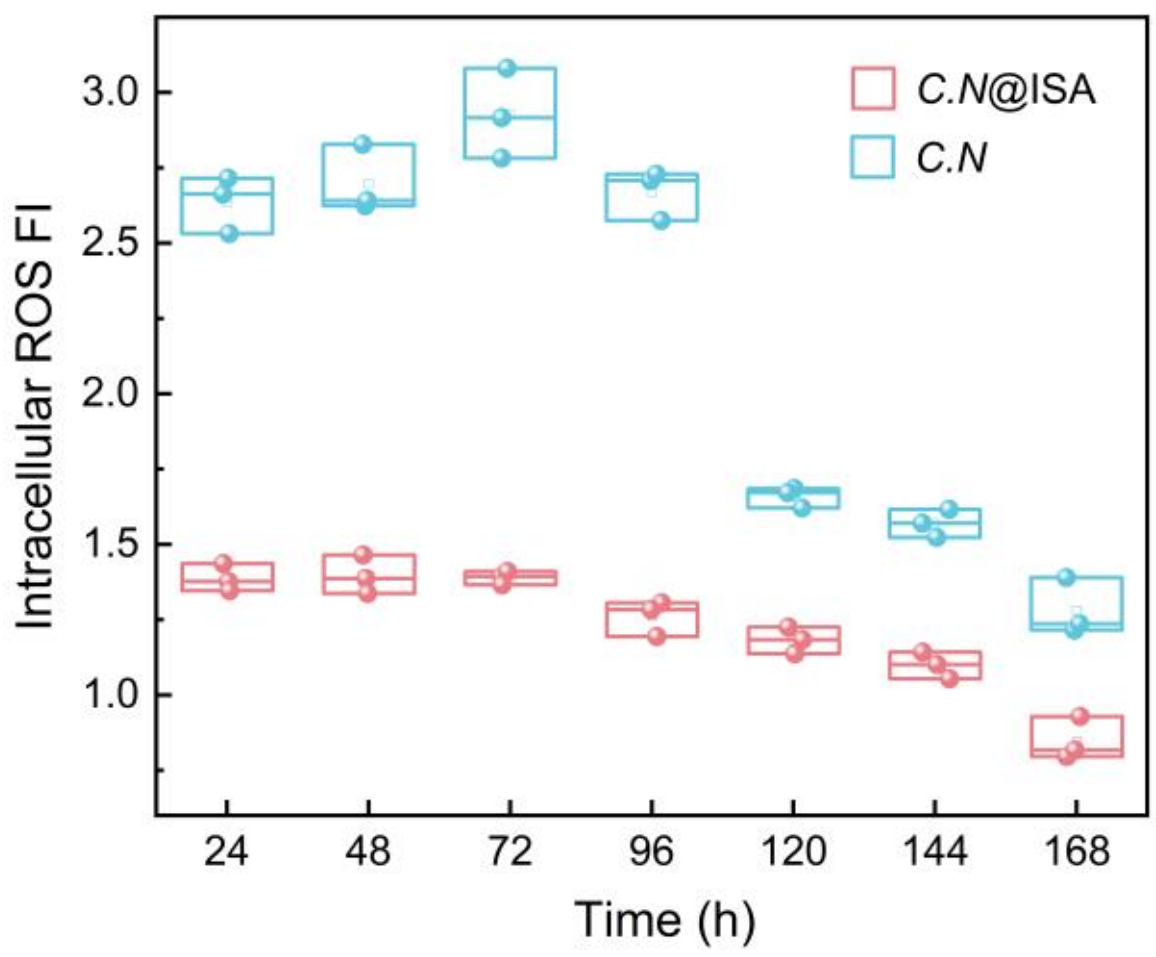

Fig. S35. Intracellular ROS fluorescence intensity (FI) during the operation (n = 3 independent experiments). DCFH-DA was used as a fluorescent probe. Source data are provided as a Source Data file.

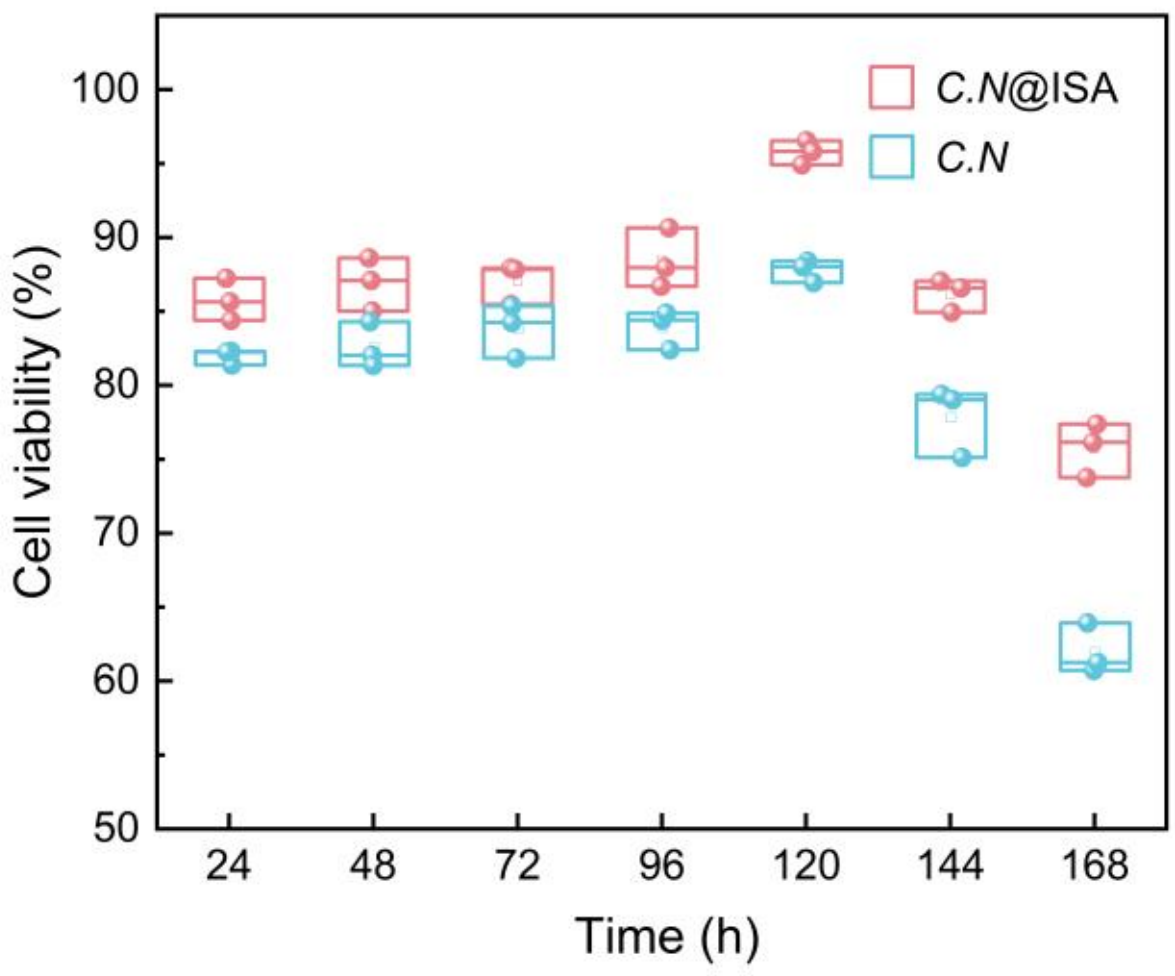

Fig. S36. Cell viability during the operation (n = 3 independent experiments). Cell viability was determined by using dual staining with SYTO 9 nucleic acid stain and propidium iodide (PI). Source data are provided as a Source Data file.

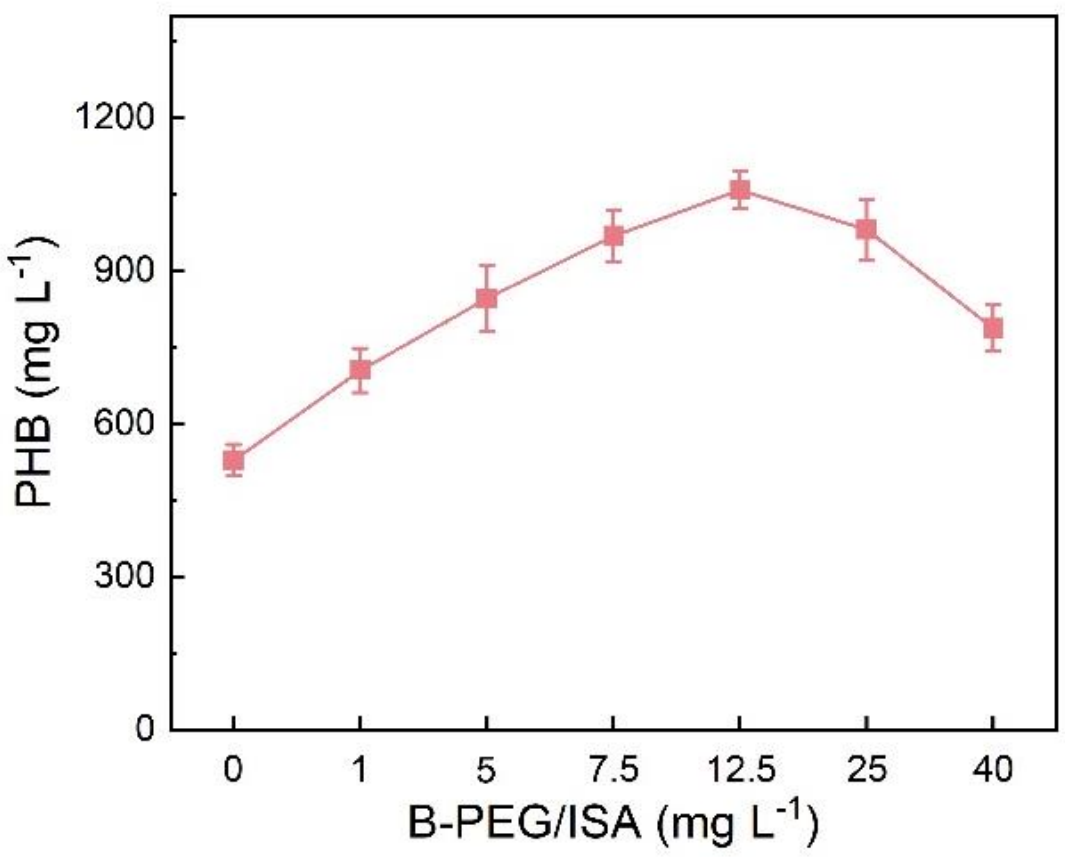

Fig. S37. PHB yield with 0 to 40 mg L^{-1} B-PEG/ISA at 168 h under the applied current density of 4.8 mA cm^{-2}. Data are presented as mean ± s. d. from n = 3 independent experiments. Source data are provided as a Source Data file.

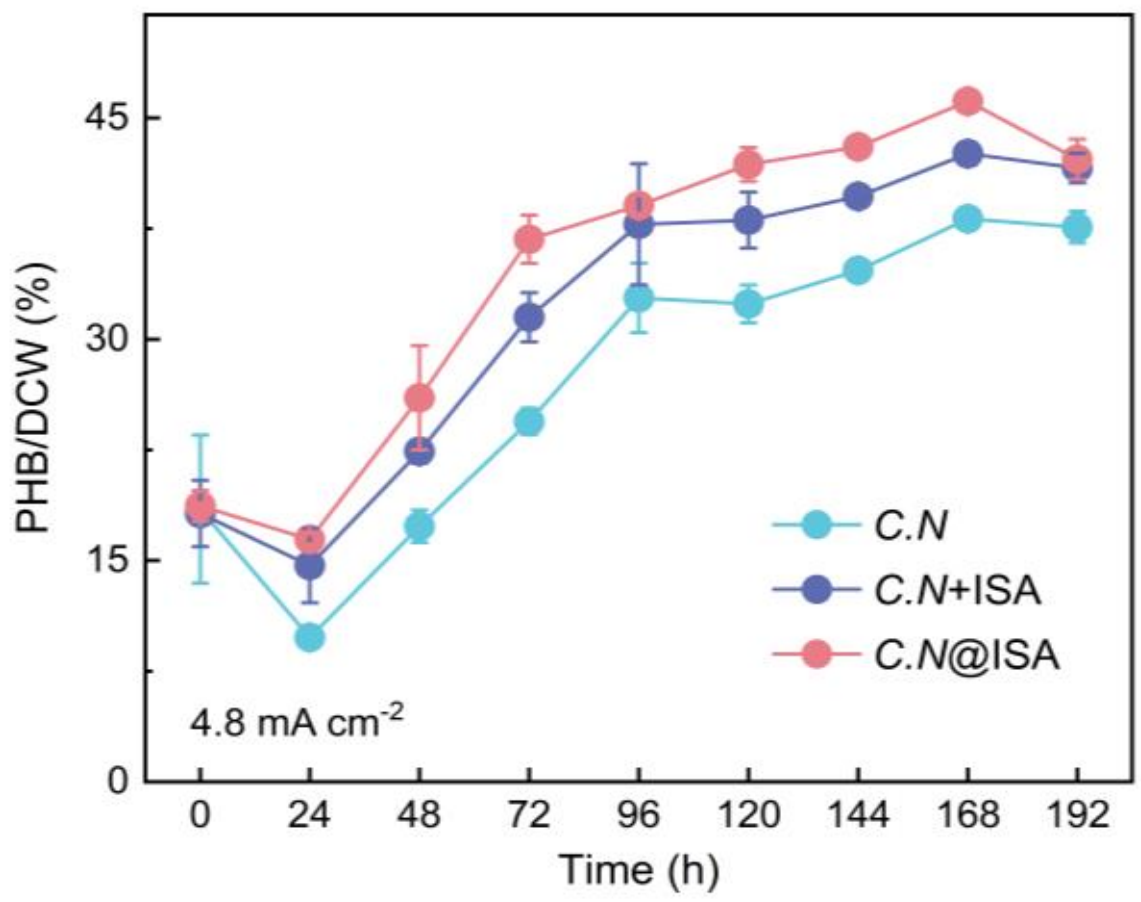

Fig. S38. PHB/DCW of the integrated inorganic-biological system. Data are presented as mean ± s. d. from n = 3 independent experiments. Source data are provided as a Source Data file.

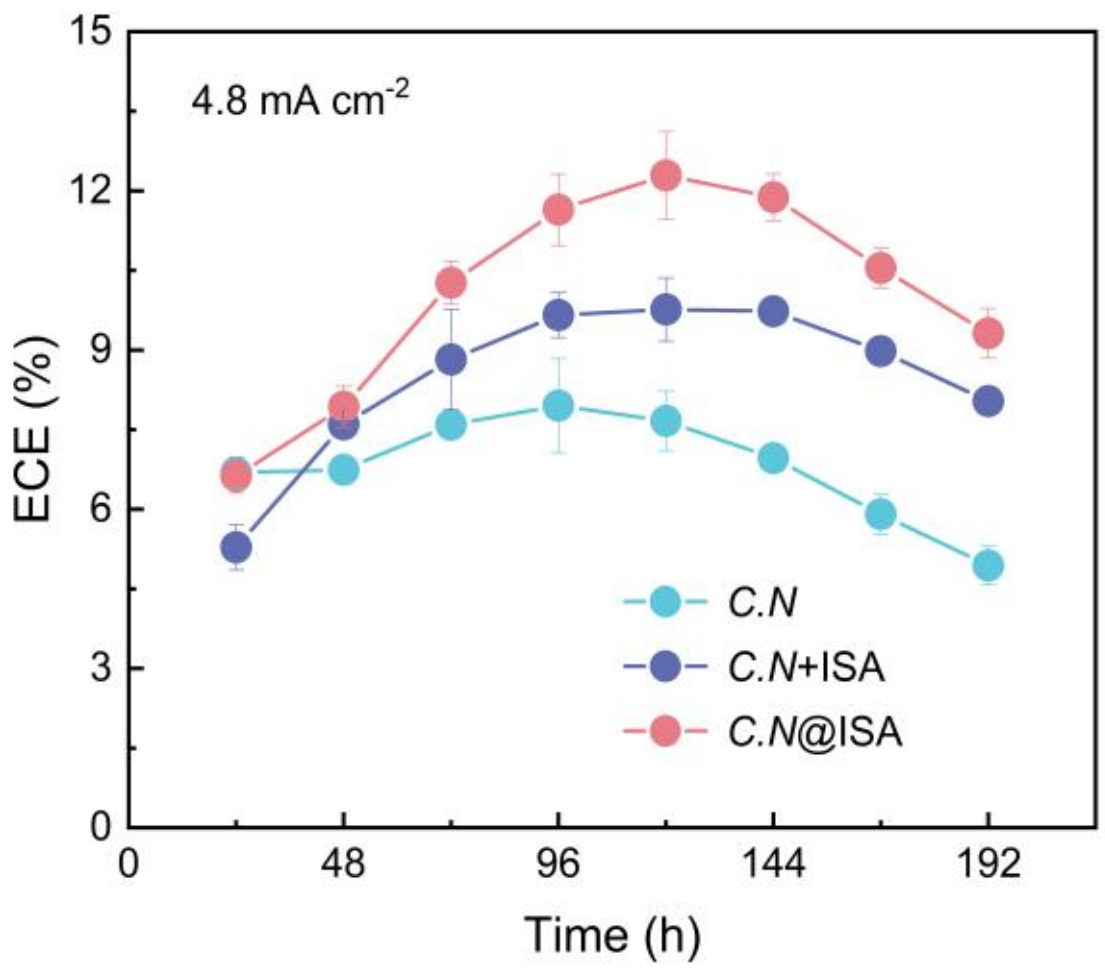

Fig. S39. ECE of the integrated inorganic-biological system. Data are presented as mean ± s.d. from n = 3 independent experiments. Source data are provided as a Source Data file.

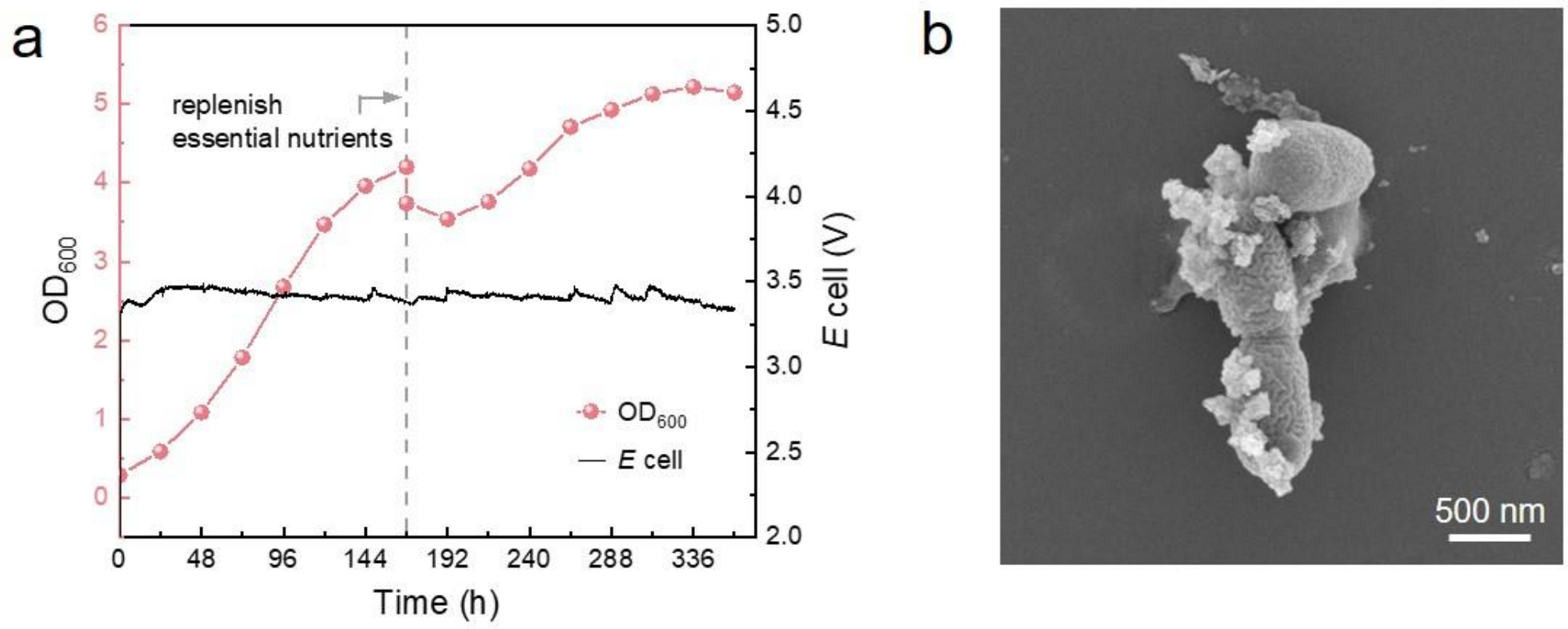

Fig. S40. Long-term stability evaluation of the *C.N*@ISA hybrid system. (a) Continuously monitored OD_{600} and reactor anode–cathode voltage during long-term operation. (b) SEM images of *C.N*@ISA from the integrated system after long-term operation. The E_{cell} is not *iR* corrected. Source data for a are provided as a Source Data file.

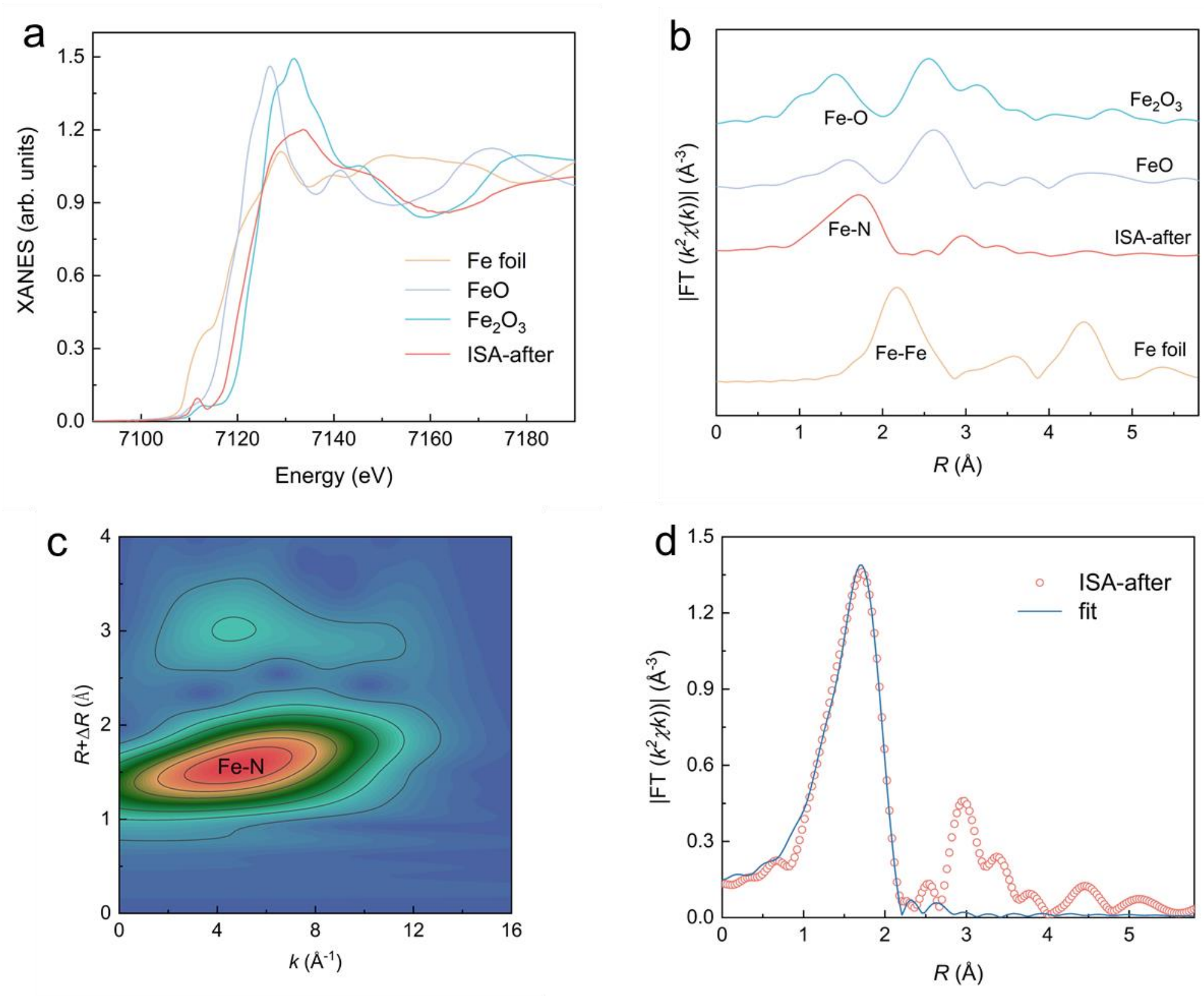

Fig. S41. Long-term stability evaluation of ISA. (a) XANES spectra of ISA-after, Fe foil, Fe_2O_3 and FeO. (b) FT of the Fe K-edge of ISA-after, Fe foil, Fe_2O_3 and FeO. (c) WT-EXAFS contour plots of ISA-after. (d) Corresponding EXAFS *R* space fitting curves of ISA-after. The ISA was detached from the bacterial surface under mild, competitive-diol-mediated conditions to minimize perturbation of the Fe–N coordination environment. Specifically, the post-reaction biohybrids were washed with PBS, then incubated for 20 min in a weakly acidic buffer (pH 6.8) containing fructose (200 mM), which disrupts the reversible phenylboronic acid–cis-diol linkage on the cell surface. After that, intermittent sonication at low power (60 W) was applied for 2 min, followed by differential centrifugation to recover the catalyst (4000 rpm for cell separation, 12000 rpm for ISA separation). Source data are provided as a Source Data file.

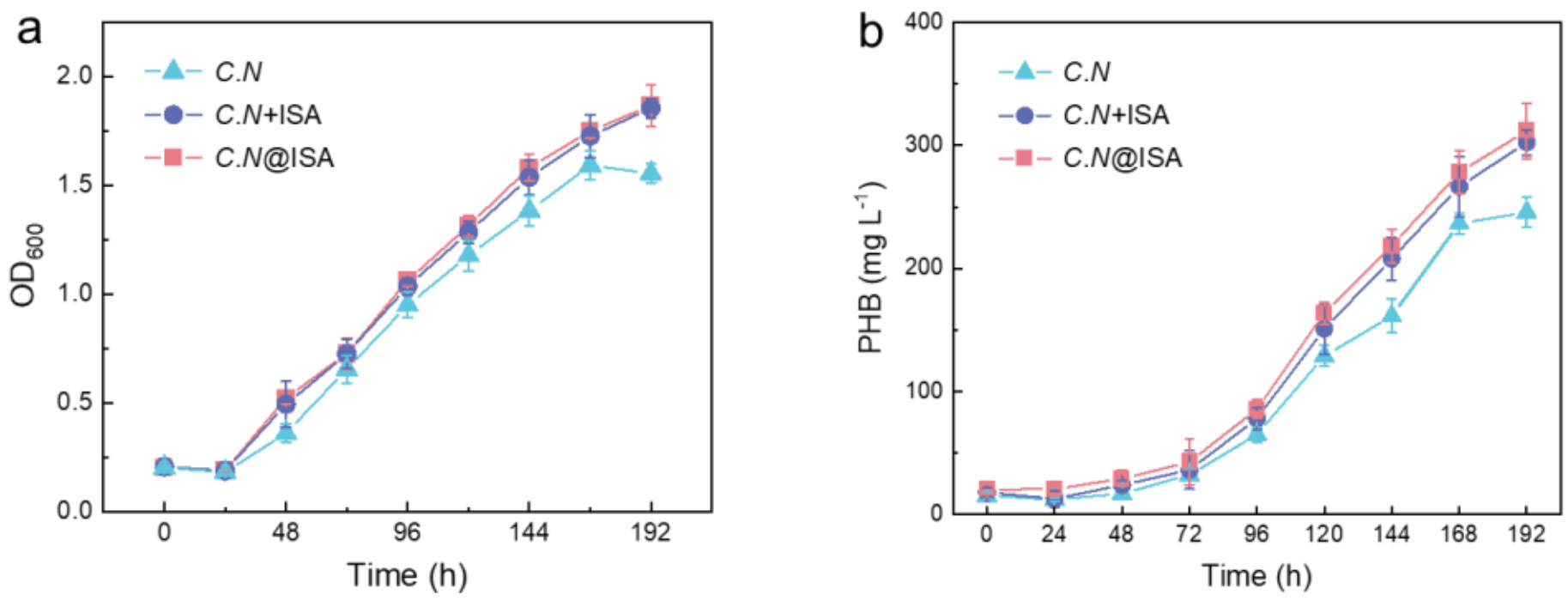

Fig. S42. (a) OD_{600} values and (b) PHB yield within 192 h of the integrated inorganic-biological system operation at 2.4 mA cm^{-2}. Data are presented as mean ± s. d. from n = 3 independent experiments. Source data are provided as a Source Data file.

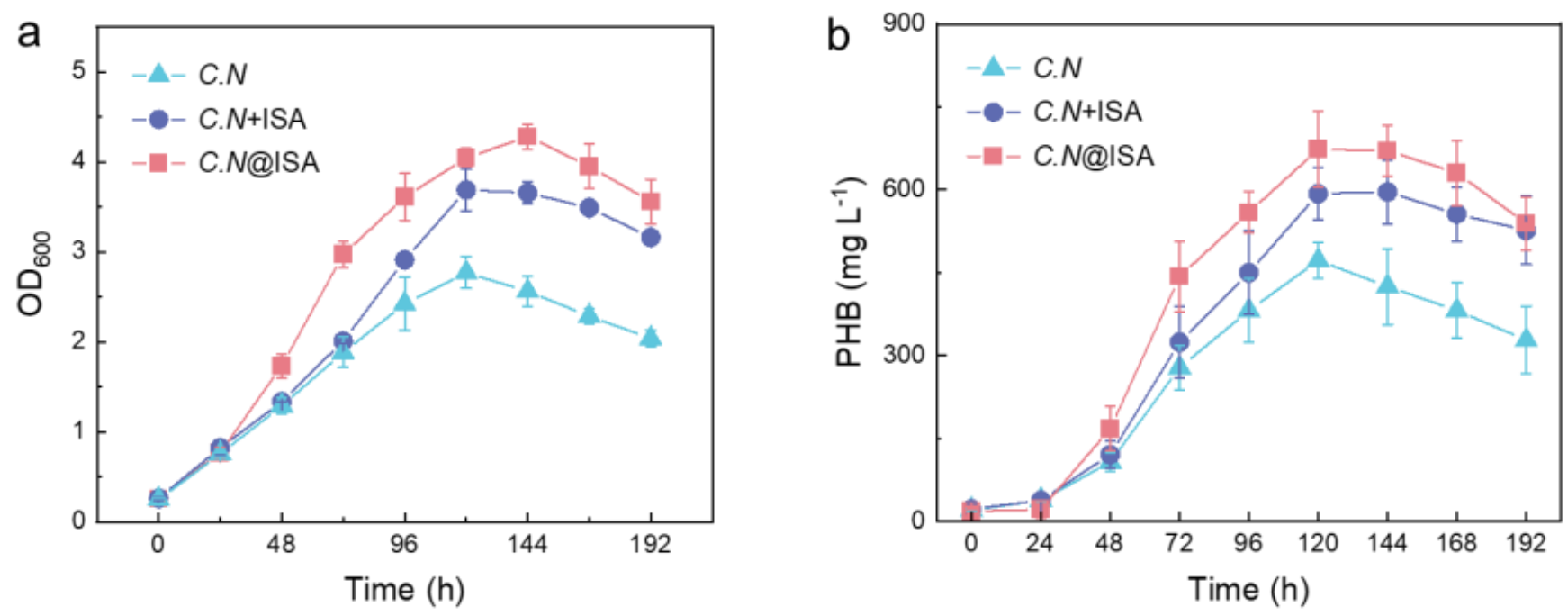

Fig. S43. (a) OD_{600} values and (b) PHB yield within 192 h of the integrated inorganic-biological system operation at 5.6 mA cm^{-2}. Data are presented as mean ± s. d. from n = 3 independent experiments. Source data are provided as a Source Data file.

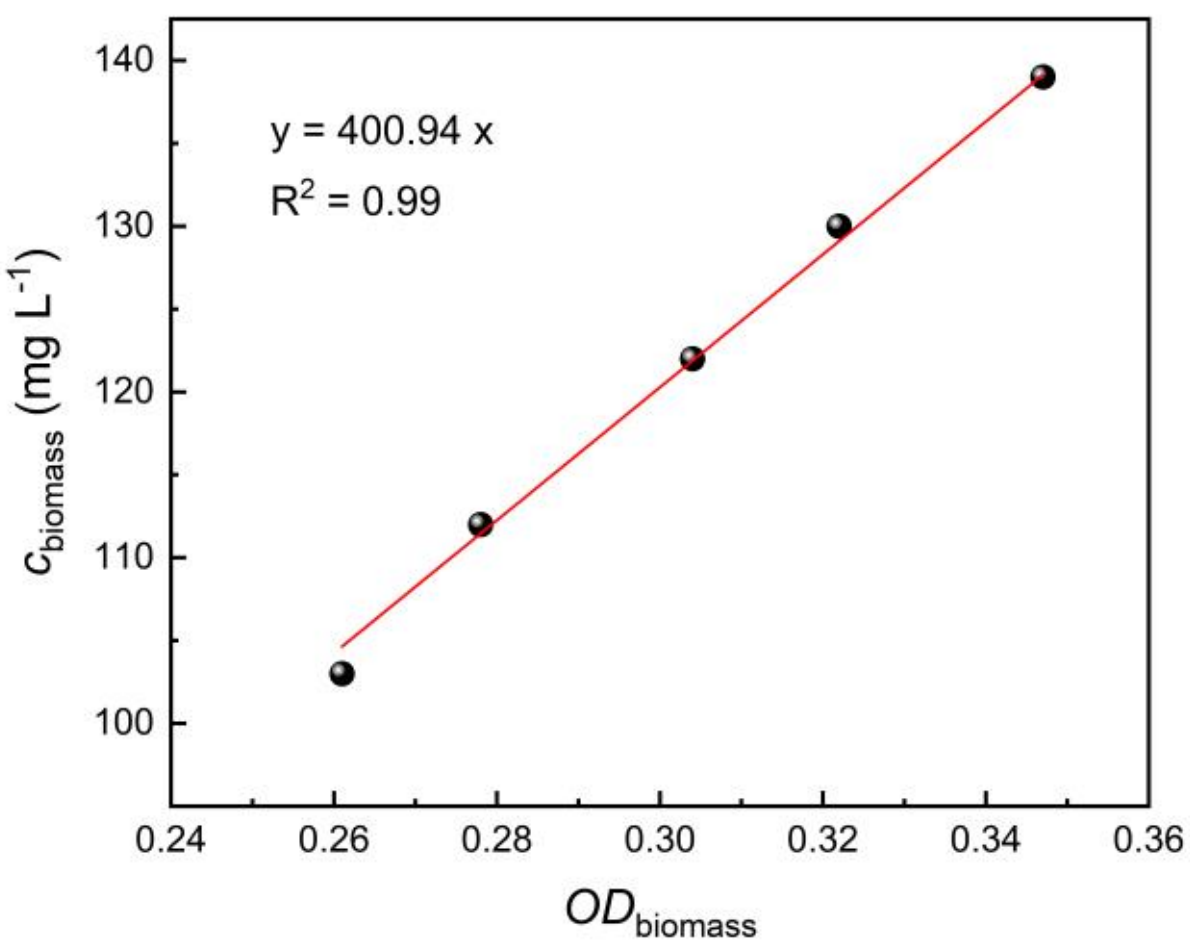

Fig. S44. The fitting plot of biomass coefficient. Source data are provided as a Source Data file.

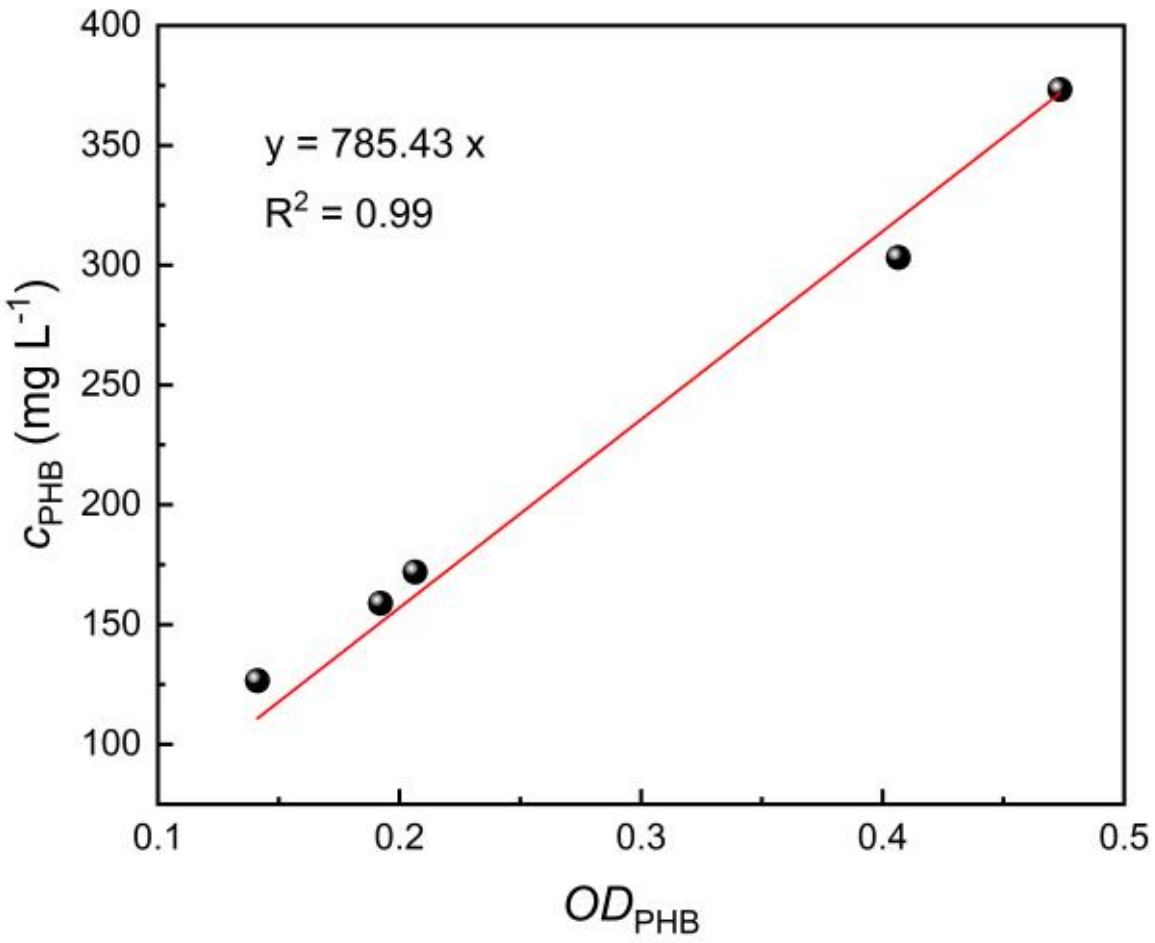

Fig. S45. The fitting plot of PHB scattering coefficient. Source data are provided as a Source Data file.

2. Supplementary Tables

Table S1. EXAFS data fitting results of samples.

Sample	Path	CN[a]	R(Å)[b]	σ^2 (Å^2)[c]	ΔE_0(eV)[d]	R factor
Fe foil	Fe-Fe	8.0*	2.47	0.0051	7.2	0.0026
	Fe-Fe	6.0*	2.85	0.0066		
ISA	Fe-N	3.6	2.02	0.0068	5.0	0.0191
FePc	Fe-N	4.1	2.03	0.0093	7.5	0.0115

[a]CN, coordination number; [b]R, the distance between absorber and backscatter atoms; [c]σ^2, the Debye Waller factor value; [d]ΔE_0, inner potential correction to account for the difference in the inner potential between the sample and the reference compound; R factor indicates the goodness of the fit. * This value was fixed during EXAFS fitting, based on the known structure of Fe.

Table S2. Parameter estimates and fitting errors for the EIS data.

Group	*C.N*	*C.N*+ISA	*C.N*@ISA
R_s (Ω)	20.95 (0.37%)	20.84 (0.42%)	20.07 (0.46%)
Y_0 (S·sec^n)	1.551×10^{-3} (0.54%)	1.698×10^{-3} (0.63%)	1.980×10^{-3} (0.71%)
n	0.855 (0.26%)	0.850 (0.30%)	0.824 (0.35%)
R_{ct} (Ω)	4604 (1.75%)	3573 (1.67%)	2624 (1.76%)

Table S3. Comparison to previous studies of the integrated systems for CO_2 reduction.

Source	This work	Ref [1]	Ref [2]	Ref [3]	Ref [4]	Ref [5]	Ref [6]	Ref [7]
Cell	Single-chamber	Single-chamber	Single-chamber	H-type	Three-chamber	Single-chamber	Three-chamber	Single-chamber
Temp. (°C)	30	30	30	30	n.a.	30	30	30
Cathode (loading)	SS (None)	Co-P (n.a.)	NC (1 mg/cm^2)	Carbon cloth (n.a.)	GDE (0.8 mg/cm^2)	Ni@N-C (2 mg/cm^2)	Sn-based GDE (n.a.)	Platinized titanium (n.a.)
Anode	Pt	CoPi	Pt	Pt	IrO_2	Pt	Platinated titanium	n.a.
E_{cell} (V)	~3.4	2	3.8	n.a.	n.a.	~4	n.a.	n.a.
C_{buffer} (mM)	36	36	72	100	200	36	200	10
Stability	360 h	6 days	48 days (6 cyc.)	120 h	150 h	7 days	2 h	11 days
Electrolyte resistance	n.a.	n.a.	1.8 mS/cm	n.a.	n.a.	n.a.	8.8 mS/cm	n.a.
Membrane type	None	None	None	PEM	AEM + CEM in PSE	None	PEM	None
PHB yield (mg/L)	1060	700	538	485	510	590	56	333
Production rates (mg/L/h)	6.31	4.86	3.2	4.04	4.25	4.10	1.90	1.98
PHB/ DCW (%)	46	n.a.	44	n.a.	n.a.	43	34	43

PEM: Proton exchange membrane. AEM: Anion-exchange membrane. CEM: Cation-exchange membrane. PSE: Porous solid electrolyte.

Table S4. EXAFS data fitting results of samples after operation (ISA-after).

Sample	Path	CN[a]	R(Å)[b]	σ^2 (Å^2)[c]	ΔE_0(eV)[d]	R factor
Fe foil	Fe-Fe	8.0*	2.47	0.0051	-0.5	0.0083
	Fe-Fe	6.0*	2.85	0.0061		
ISA-after	Fe-N	3.7	2.20	0.0070	-5.8	0.0041
FePc	Fe-N	4.0	1.90	0.0068	4.2	0.0142

[a]CN, coordination number; [b]R, the distance between absorber and backscatter atoms; [c]σ^2, the Debye Waller factor value; [d]ΔE_0, inner potential correction to account for the difference in the inner potential between the sample and the reference compound; R factor indicates the goodness of the fit. * This value was fixed during EXAFS fitting, based on the known structure of Fe.

References

1. Liu, C., Colón, B. C., Ziesack, M., Silver, P. A. & Nocera, D. G. Water splitting-biosynthetic system with CO_2 reduction efficiencies exceeding photosynthesis. *Science* **352**, 1210-1213 (2016).
2. Yao, J. N. et al. Efficient CO_2 conversion by biocompatible N-doped carbon nanosheets coupled with *Ralstonia eutropha*: synergistic interactions between microbial and inorganic catalysts. *Green Chem.* **25**, 4760-4768 (2023).
3. Chen, X., Cao, Y., Li, F., Tian, Y. & Song, H. Enzyme-assisted microbial electrosynthesis of poly(3-hydroxybutyrate) via CO_2 bioreduction by engineered *Ralstonia eutropha*. *ACS Catal.* **8**, 4429-4437 (2018).
4. Wi, T. U. et al. Upgrading carbon monoxide to bioplastics via integrated electrochemical reduction and biosynthesis. *Nat. Synth.* **3**, 1392-1403 (2024).
5. Li, Z. J. et al. Water splitting-biosynthetic hybrid system for CO_2 conversion using nickel nanoparticles embedded in N-doped carbon nanotubes. *Chemsuschem* **11**, 2382-2387 (2018).
6. Stöckl, M., Harms, S., Dinges, I., Dimitrova, S. & Holtmann, D. From CO_2 to bioplastic - coupling the electrochemical CO_2 reduction with a microbial product generation by drop-in electrolysis. *Chemsuschem* **14**, 2780-2780 (2021).
7. Langsdorf, A., Schütz, J. P., Ulber, R., Stöckl, M. & Holtmann, D. Production of polyhydroxybutyrate from industrial flue gas by microbial electrosynthesis. *J. CO2 Util.* **83**, 102800 (2024).
