# Supplementary material for: Interface-engineered iron single-atom biohybrids for efficient CO2-to-bioplastic conversion
Source: Nat Commun. 2026 Aug 20;17:9946. doi: 10.1038/s41467-026-76902-9 (PMC13586381; doi:10.1038/s41467-026-76902-9)
Supplement: Supplementary file 2 [file 41467_2026_76902_MOESM2_ESM.pdf]

Description of Additional Supplementary Files

File Name: Supplementary Data 1

Description: The optimized models used for calculations.
