# Supplementary material for: Interface-engineered iron single-atom biohybrids for efficient CO2-to-bioplastic conversion
Source: Nat Commun. 2026 Aug 20;17:9946. doi: 10.1038/s41467-026-76902-9 (PMC13586381; doi:10.1038/s41467-026-76902-9)
Supplement: Supplementary file 4 [file 41467_2026_76902_MOESM5_ESM.pdf]

nature portfolio

Peer Review File

Interface-engineered iron single-atom biohybrids for efficient CO2-to-bioplastic conversion

Corresponding Author: Professor Zhengwei Mao

This file contains all reviewer reports in order by version, followed by all author rebuttals in order by version.

Attachments originally included by the reviewers as part of their assessment can be found at the end of this file.

Version 0:

Reviewer comments:

Reviewer #1

(Remarks to the Author)
This manuscript presents a highly innovative and well-executed study on the construction of an inorganic–biological hybrid system for efficient CO2-to-bioplastic conversion. The authors successfully engineered the microbial surface of Cupriavidus necator with iron single-atom catalysts (ISA) via click chemistry, leading to significantly enhanced H2 activation, electron/proton transfer, and intracellular energy metabolism. The work is comprehensive, combining advanced materials characterization, electrochemical analysis, DFT calculations, and transcriptomics to elucidate the underlying mechanisms. The reported PHB yield and Faradaic efficiency are impressive and represent a state-of-the-art performance in microbial electrosynthesis. The manuscript is generally well-written and supported by extensive supplementary data. However, several points require clarification or additional evidence to fully support the claims. Therefore, the reviewer will recommend a major revision.
The detailed comments:
1. While the DFT calculations and EPR data support H2 dissociation at ISA sites, it remains unclear how the dissociated protons and electrons are efficiently channeled into the microbial electron transport chain. Additional experimental evidence, such as isotopic labeling or real-time monitoring of electron flux, would strengthen this claim.
2. The authors demonstrate excellent electrochemical stability of ISA, but the long-term stability and reusability of the C.N@ISA biohybrid under continuous operation are not thoroughly evaluated. Data on multiple batch cycles or extended operation would be valuable.
3. The CLSM and Raman data suggest selective binding of B-PEG/ISA to α-sites of polysaccharides. However, the possibility of non-specific binding or interference with membrane proteins should be further addressed, such as via competitive binding assays or mutant strains lacking specific polysaccharide motifs.
4. The ROS scavenging data are supportive but qualitative. Quantification of intracellular ROS levels and correlation with cell viability under operational conditions would provide more direct evidence of the protective role of ISA.
5. The discrepancy between the theoretical and experimental FE values, especially for C.N, warrants further discussion. Are there other electron sinks or metabolic side reactions not accounted for in the model?

Reviewer #2

(Remarks to the Author)
This manuscript presents an exceptionally innovative and systematic study that establishes a new paradigm for inorganic–biological hybrid systems. The authors have successfully engineered a microbial interface by covalently anchoring iron single-atom catalysts (ISA) onto the membrane of Cupriavidus necator, enabling highly efficient CO_2-to-PHB conversion. The work introduces an original concept of "single-atom catalysis–microbial membrane interface engineering," offering a transformative enhancement strategy for H_2-mediated electromicrobial synthesis. The innovation of this work lies in its elegant integration of single-atom catalysis with microbial metabolism through precise interface design. The ISA functions as an artificial hydrogenase to create a localized H_2-rich and proton-rich microenvironment, simultaneously facilitating electron/proton transfer and mitigating reactive oxygen species. This dual functionality significantly strengthens bioenergetic coupling and metabolic flux toward CO_2 fixation. The research achieves benchmark-level PHB production and Faradaic efficiency, far exceeding most previously reported systems. Scientifically, the manuscript offers a new conceptual and technical framework for addressing two long-standing bottlenecks—limited H_2 bioavailability and sluggish hydrogenase

kinetics—in electromicrobial CO_2 utilization. From the perspective of potential impact, this work could serve as a cornerstone for future carbon-negative biomanufacturing strategies coupling renewable energy with microbial catalysis.
Overall, the manuscript is logically structured, experimentally comprehensive, and methodologically rigorous. The figures are of high quality and clearly support the authors' conclusions. The combination of advanced characterization (HAADF-STEM, XANES/EXAFS, EIS, CLSM), theoretical DFT calculations, and transcriptomic analysis demonstrates impressive depth and technical sophistication. The study is complete and compelling, and the conclusions are well supported by solid experimental evidence. I therefore recommend publication after minor revisions.

Comments and Questions
1. The iron signal in the EDS of Figure S1 is not very obvious. Are there higher-resolution images, such as HAADF-STEM EDS mapping, to further confirm the presence and distribution of iron species?
2. The manuscript includes several control materials (e.g., Fe-free and different calcination temperatures). How did the authors determine the optimal ISA configuration (Fe-N_4pyd-C) for subsequent experiments? Were other possible Fe coordination structures considered in the DFT screening?
3. In the theoretical section, how was it determined that H_2 adsorption occurs primarily at the Fe atom? Please also provide the Bader charge of the Fe site and its neighboring atoms. Moreover, adsorption energies were calculated for three models, yet energy barrier calculations were performed for only two—please clarify the reason for this selection.
4. What is the intrinsic conductivity of the B-PEG linker used, and how does it quantitatively contribute to promoting electron/proton transfer? Since extracellular polysaccharides are typically insulating, could the anchoring of B-PEG/ISA to such substrates partially limit charge transport?
5. In the electrochemical setup, how did the authors experimentally simulate the H_2-derived electron supply using electrical current? Please clarify the mechanistic connection between current density and biological electron uptake.
6. In Figure 4h, $K_3Fe(CN)_6$ is used as a redox mediator. Please elaborate on the rationale for this selection and whether similar effects could be achieved with other mediators.
7. The validation of membrane potential is very interesting. Please describe the detailed operating conditions (e.g., dye concentration, incubation time). The authors also mention the proton motive force (PMF); could the relative contributions of membrane potential and proton gradient to PMF be quantitatively compared?
8. Please describe how dry cell weight was measured. Considering that ISA is attached to the membrane, could this additional mass cause a slight overestimation in the dry weight, thereby affecting PHB/DCW calculations?
9. Regarding Faradaic efficiency, what is the exact physical meaning of FE and FET in Fig. 6, and how are they correlated? The text suggests that FET decreases for ISA-modified cells, but the experimental FE increases—please clarify this apparent inconsistency.

Reviewer #3

(Remarks to the Author)
The authors presented a new route to link the single-atom iron to C. necator by a organic linker based on click reaction, which showed the enhancement of PHB production with FE about 44.6%. The possible mechanisms were postulated based on the electrochemical analysis.
However, besides the linkage, the novelty of the work is weak comparing to previous work, such as by Liu et al. (10.1126/science.aaf5039) etc. And the details of the evidence should also be checked as well as calculations based on empirical equations.
1. For the calculation of biomass and energy in ECE and FE calculations, the cited equations and parameters were obtained by the measurement under similar conditions. But due to the cultivation conditions' changes and cell components varied a lot (such as PHB content, etc), it's better to use direct measurements, other than an empirical equation. If using the empirical equation, please refer to citations to adjust the parameter for different cultivation conditions.
2. In the Figure S26, why the stainless steel was used as the WE? And why the free C.N@ISA was used for the test? It's better to compared with C.N@ISA immobilized on the electrode. Or the significant advantages of free C.N@ISA should be well presented. The stability of the system should be proved as long as the cultivation, i.e. 192h, not just 30 min in Fig 4h.
3. The biological mechanisms were not confirmed. Although some postulations were made based on the literatures and measurements of heme, reduction power, gene expressions, but they were all not the direct evidence for the electrons' functions. With both hydrogen and electrodes, the contribution for direct H2 and electrochemical should be analyzed, with more control or blank experiments.
4. Some literatures were not cited rightly, or read carefully. For example, in the last two line in the first page of introduction section, the ref 19 is talking about a direct electron supply by the organic semiconductors, no relation to single-atom metal catalysts or H2 dissociation energy barrier.
In brief, the manuscript presented plenty of data and tests, while the key contributions should be reorganized and the a logical chain from the single-atom iron to hybrid system should be proposed.

Reviewer #4

(Remarks to the Author)
The authors report an iron single-atom biohybrid constructed via click chemistry and claim high CO_2-to-PHB production and Faradaic efficiency. However, the manuscript faces some major design flaws, method inconsistencies, and some mechanism interpretations are not supported by the data. In several cases the experiments are inconsistent. Overall, the work falls short of the quality needed for NC, and I provide some detailed comments below.

1. A major problem the authors mentioned about ROS generated at the anode severely hindering microbial electrosynthesis is largely created by the reactor design in this study. They use a single-chamber electrochemical reactor, in which O_2 evolved at the anode can freely diffuse to the cathode. In conventional microbial electrosynthesis, a membrane-separated two-chamber configuration is used specifically to prevent such O_2 crossover, so ROS are not a dominant limitation. As shown in Fig. 6c, most electrons in their system appear to be consumed by ORR, which is thermodynamically favored over proton reduction. In other words, the reactor is essentially running OER and ORR in a loop, wasting electrons and energy. This design creates an artificial ROS problem and undermines the efficiency claims. The authors probably can redo the tests in a standard two-chamber reactor.

2. The reference electrode and potential scale should be reported consistently. The text around the CV data indicates that potentials are referenced to saturated Ag/AgCl, but this is easy to miss and is not clearly reflected in all figure captions. Given that the mechanistic arguments rely on potential windows (e.g., for HOR/HER), the reference scale should be explicitly stated where electrochemical data are plotted.

3. The electrochemical characterization used to support the mechanism doesn't seem to be representative of the actual microbial electrosynthesis system. The reactor configuration for CO_2-to-PHB conversion (method 4.4) uses a stainless-steel mesh cathode and planktonic C.N or C.N@ISA in a single-chamber cell. In contrast, the CV/EIS experiments (method 4.8) employ carbon cloth electrodes pre-loaded with B-PEG/ISA and then colonized by cells to form a C.N@ISA-loaded bioelectrode. These are fundamentally different electrode architectures, with different surface areas, transport properties, and cell/material interactions. If microbial electrosynthesis performance is dominated by planktonic C.N/ISA hybrids in the bulk, then the behavior of a pre-formed C.N@ISA biofilm on carbon cloth is not relevant. If the authors instead claim that biofilms on the stainless-steel mesh dominate, then method 4.4 does not recreate the same structure as method 4.8. In either case, the electrochemical results in Fig. 4 are decoupled from the actual reactor configuration and cannot be used to justify the proposed promotion mechanism.

4. The stability claims for ISA are not convincing in the context of the actual reactor conditions. The authors show that ISA morphology and Fe content remain unchanged after 120 h of electrolysis, but this test appears to be under HER-like conditions without significant O_2. It is well known that Fe–N–C catalysts degrade much more rapidly under ORR, and in the authors' single-chamber system O_2 is inevitably produced at the anode and can diffuse to the cathode. Furthermore, if the core functionality is attributed to planktonic C.N–ISA hybrids, then electrode-level HER/ORR stability is not directly relevant to the biological performance. The stability discussion therefore does not substantiate the claims being made.

5. The mechanistic narrative around H_2 dissociation, H radical formation, and H^+ supply is not supported by the data. Different electrode preparation methods are used for different panels in Fig. 4, making mechanistic interpretation very difficult. Specifically,

- The RRDE data in Fig. 4b do not show the typical mass-transport-limited plateau expected for HOR; the continued rise in current at higher potentials is more consistent with oxidation of partially reduced Fe sites and other side processes, potentially overestimating "HOR activity" differences with and without ISA.
- Even if ISA on an electrode increases HOR current, this does not demonstrate that planktonic C.N–ISA hybrids in bulk solution can provide additional H^+ to the cells. Spontaneous dissociation of H_2 (bond energy = 436 kJ mol^{-1}) by planktonic hybrids without a clear energy input is hard to believe, and in a buffered medium any H^+ produced locally would be rapidly neutralized.
- The DMPO-H· EPR signals used to claim "hydrogen radical" formation seem suspicious: similar signals appear in the DMPO blank, and side reactions in the electrochemical cell can easily generate DMPO-H· artifacts. This does not prove the existence of bulk H radicals under their conditions.
- The DFT calculations are performed on Fe-N_4 motifs in vacuum and track H_2 adsorption and dissociation to H* on the surface. While this may have some qualitative relevance for H_2 binding, it does not justify conclusions about free H· or H^+ in buffered solution, nor does it demonstrate that the energetic barriers are overcome in the actual reactor. H*, H· and H^+ are distinct species and should not be conflated, and RRDE alone cannot distinguish between them.

Taken together, the combination of RRDE, EPR and DFT does not provide robust evidence for the claimed H radical formation or enhanced H^+ supply. A more conservative interpretation would be improved local H_2 transport at the ISA surface, but even that is not cleanly linked to the planktonic hybrid system used in the reactor.

6. The EIS data in Fig. 4g are also difficult to interpret. Over the wide frequency range of 0.01–100 kHz, one would typically expect to resolve at least separate high-frequency charge-transfer and lower-frequency transport contributions. The presence of a single semicircle and the chosen equivalent circuit model therefore require more justification. As presented, the EIS analysis does not convincingly support the claim of significantly enhanced electron transfer.

7. The performance comparison in Fig. 6b is not rigorous. Comparing final PHB concentrations across different studies without normalizing for electrode area, operating time, or cell density is not meaningful. The apparent "record" performance could be largely due to longer operation or larger electrode area. Production rates (e.g., mg L^{-1} day^{-1} or mg cm^{-2} day^{-1}) should be used for fair comparison, and even the underlying reactor designs differ substantially.

Version 1:

Reviewer comments:

Reviewer #1

(Remarks to the Author)
The reviewers were satisfied with the revisions, noting that the authors have thoroughly and effectively addressed all the points raised during the initial review. Based on this positive assessment, they have recommended the manuscript for acceptance.

Reviewer #2

(Remarks to the Author)
The authors have addressed my concerns.

Reviewer #3

(Remarks to the Author)
The revised version meets most of my concerns. If possible, some self-evaluated words can be removed, such as "record-high". In fact, these kind of conclusions can only be obtained under a series of limitations. For example, the PHB production by C. necator has already been over 200 g/L for industrialization, and a few gram/L levels are very common in lab scale reports, even in some hybrid systems.

Reviewer #4

(Remarks to the Author)
Please see attached review comments. There are some graphs so PDF attached.

Reviewer #5

(Remarks to the Author)
To avoid bias, I first read the original manuscript and the revised version, without consulting the prior reviewer reports. In my opinion, this manuscript describes an interface engineering strategy that anchors iron single-atom catalysts onto Cupriavidus necator via click chemistry, achieving efficiency CO_2-to-PHB conversion with enhanced Faradaic efficiency. Then I carefully read the rebuttal letter, the authors have conducted a careful revision, addressing the major concerns (including some questions raised in my independent review before read the rebuttal letter) through substantial new experimental data, including extended stability tests, isotopic labeling, quantitative ROS measurements, competitive binding assays, and a recalibration of biomass quantification. The core findings are now methodologically sound and convincingly supported.
I have found some additional questions in the manuscript that touch upon the long-term chemical stability of the active sites, the partitioning of electron flux, etc. These points do not undermine the validity of the conclusions, but should be properly addressed before publication in Nature Communications. Here are my specific comments:
1. The 360-hour fed-batch experiment demonstrates sustained ISA decoration on cells, yet the chemical state of the Fe–N4 moieties after prolonged operation remains uncharacterized. Is there any indirect or direct evidence indicating whether the coordination environment undergoes gradual alteration under continuous exposure to bacterial metabolites, local pH fluctuations, and reactive oxygen species?
2. The detection of flavin adenine dinucleotide (FAD) and its correlation with enhanced current responses support a dual electron-transfer mechanism. However, the relative quantitative contribution of each pathway remains ambiguous. Can the authors provide a more clarification regarding what fraction of the total electron flux is attributable to FAD-mediated shuttling versus direct hydrogen oxidation at the ISA interface?
3. In Fig. S28, the elevated ATP levels and proton motive force observed in the electrochemical system compared to direct H_2 bubbling suggest a “metabolic rewiring” effect. Is this phenomenon primarily a consequence of the local proton gradient generated by ISA, or could the electrochemical environment itself on the microbial energy metabolism?
4. The nanozyme-like ROS scavenging activity of ISA is presented as a protective mechanism. Given that C. necator is a facultative autotrophic bacterium that relies on oxidative phosphorylation for ATP generation, is there any risk that excessive ROS scavenging could inadvertently interfere with essential redox signaling or limit the availability of reactive oxygen species required for normal metabolic function?
5. The covalent anchoring of ISA to surface polysaccharides places the catalyst in close proximity to the cell membrane. Beyond the established hydrogen-enrichment effect, could the physical presence of ISA alter the conformation, lateral mobility, or activity of adjacent membrane proteins? Is there any experimental evidence that direct protein–material interactions contribute to the observed metabolic enhancement?
6. The authors have revised Fig. 6b to present PHB productivity rates rather than cumulative titers, which improves the fairness of performance comparisons. However, the term “record-high” still appears multiple times in the manuscript. Given the inherent variability in reactor design, operating conditions, and cultivation durations across different studies, the reviewer recommend replacing “record-high” with more qualified words to avoid unnecessary overstatement.

Version 2:

Reviewer comments:

Reviewer #4

(Remarks to the Author)
The authors have made substantial revisions that reasonably addressed most major concerns.
Before publication, I suggest authors soften some claims on mechanisms, as they are not fully supported by experiments yet. For example, the molecular electron/proton transfer pathway from ISA to intracellular metabolism is still not directly resolved.

Reviewer #5

(Remarks to the Author)
All the comments have been well addressed. I thus recommend the publication of this work as is.

Open Access This Peer Review File is licensed under a Creative Commons Attribution 4.0 International License, which permits use, sharing, adaptation, distribution and reproduction in any medium or format, as long as you give appropriate credit to the original author(s) and the source, provide a link to the Creative Commons license, and indicate if changes were made.
In cases where reviewers are anonymous, credit should be given to 'Anonymous Referee' and the source.
The images or other third party material in this Peer Review File are included in the article's Creative Commons license, unless indicated otherwise in a credit line to the material. If material is not included in the article's Creative Commons license and your intended use is not permitted by statutory regulation or exceeds the permitted use, you will need to obtain permission directly from the copyright holder.
To view a copy of this license, visit https://creativecommons.org/licenses/by/4.0/

Response to reviews

We would like to express our sincere gratitude to the reviewers for their profound insights and constructive critiques of our manuscript. Addressing these queries and conducting the supplementary experiments has enabled us to gain a deeper and more comprehensive understanding of our research findings. We have provided point-by-point responses to all comments and have performed a thorough revision of the manuscript to address the concerns raised. We believe that these modifications have significantly enhanced the overall quality and scientific rigor of our work. For clarity, the reviewers' original comments are presented in *italics and underlined*, while all corresponding revisions in the text are indicated by **yellow highlighting**.

Reviewer #1 (Remarks to the Author):

This manuscript presents a highly innovative and well-executed study on the construction of an inorganic–biological hybrid system for efficient CO2-to-bioplastic conversion. The authors successfully engineered the microbial surface of Cupriavidus necator with iron single-atom catalysts (ISA) via click chemistry, leading to significantly enhanced H2 activation, electron/proton transfer, and intracellular energy metabolism. The work is comprehensive, combining advanced materials characterization, electrochemical analysis, DFT calculations, and transcriptomics to elucidate the underlying mechanisms. The reported PHB yield and Faradaic efficiency are impressive and represent a state-of-the-art performance in microbial electrosynthesis. The manuscript is generally well-written and supported by extensive supplementary data. However, several points require clarification or additional evidence to fully support the claims. Therefore, the reviewer will recommend a major revision.

Response:

We sincerely thank the reviewer for the highly positive evaluation and the encouraging comments on our work. We are particularly grateful for the recognition of our study's innovation in constructing the inorganic–biological hybrid system and the "state-of-the-art performance" achieved in microbial electrosynthesis. We have taken the reviewer's constructive criticisms and suggestions very seriously. In response to the specific points raised, we have performed a series of rigorous supplementary experiments. These additional data have allowed us to clarify the underlying mechanisms and significantly strengthen the conclusions of the manuscript. Below, we provide a point-by-point response to the specific comments and have revised the manuscript accordingly. We believe that these revisions have further elevated the quality and scientific rigor of our work.

The detailed comments:

1. *While the DFT calculations and EPR data support H_2 dissociation at ISA sites, it remains unclear how the dissociated protons and electrons are efficiently channeled into the microbial electron transport chain. Additional experimental evidence, such as isotopic labeling or real-time monitoring of electron flux, would strengthen this claim.*

Response:

We sincerely appreciate the reviewer's insightful comment regarding the transport pathways of dissociated protons and electrons. We agree that clarifying the entry mechanism of these species into microbial metabolism is critical. To address this, we conducted additional isotopic labeling experiments and mechanistic investigations.

1. Verification of Proton Transfer via Isotopic Labeling (D_2 Experiments)

To definitively trace the fate of dissociated protons, we conducted independent gas-fermentation experiments using deuterium (D_2) as the sole energy source. As shown in Fig. S25,

a comparison of the GC–MS spectra of PHB extracted from *C. necator* incubated with H_2 (top) versus D_2 (bottom) reveals a distinct mass shift towards higher m/z values in the D_2 group. Specifically, the emergence of dominant peaks at higher molecular weights (*e.g.,* around m/z 119-120) and altered fragmentation patterns confirm the substantial incorporation of deuterium into the polymer backbone. This provides direct chemical evidence that protons dissociated at the interface are not merely dissipating into the bulk solution but are effectively internalized and utilized by *C. necator* for NAD(P)H regeneration and subsequent biosynthesis, thereby validating the H_2 dissociation and proton transfer pathway.

2. Elucidation of the Electron Transfer Mechanism

To elucidate the electron transfer pathway, we integrated our experimental findings with recent breakthroughs in *C. necator* biohybrids. Our High-performance liquid chromatography (HPLC) analysis of the reactor supernatant provides decisive evidence for a mediator-based mechanism; as shown in Fig. S27, we detected a distinct chromatographic peak with a retention time matching that of the flavin adenine dinucleotide (FAD) standard. These results are consistent with the cyclic voltammetry (CV) data obtained from the bacterial suspension, where the presence of FAD was further verified by the positions of its characteristic redox peaks. Fu et al. (*Nat. Chem.* **15**, 1400-1407 (2023)) and Tremblay et al. (*Nat. Commun.* **16**, 8141 (2025)) reported that *C. necator* achieves significant interfacial electron uptake via self-secreted redox mediators like flavins. Consequently, we propose a FAD-Mediated Electron Transfer mechanism at the *C.N*–ISA interface, where secreted FAD molecules function as diffusible electron shuttles conveying electrons from the ISA surface to the microbial electron transport chain (presumed to enter at the quinone pool), thereby driving efficient CO_2 fixation.

In summary, our additional data confirms a synergistic mechanism: protons are transferred and assimilated into metabolites (verified by D_2 labeling), while electrons are efficiently

shuttled from the ISA catalyst to the bacterial ETC via self-secreted FAD mediators, explaining the system's high efficiency. Related discussions have been added to the revised manuscript (Section 2.3, 2.4 and Method 4.7, 4.8).

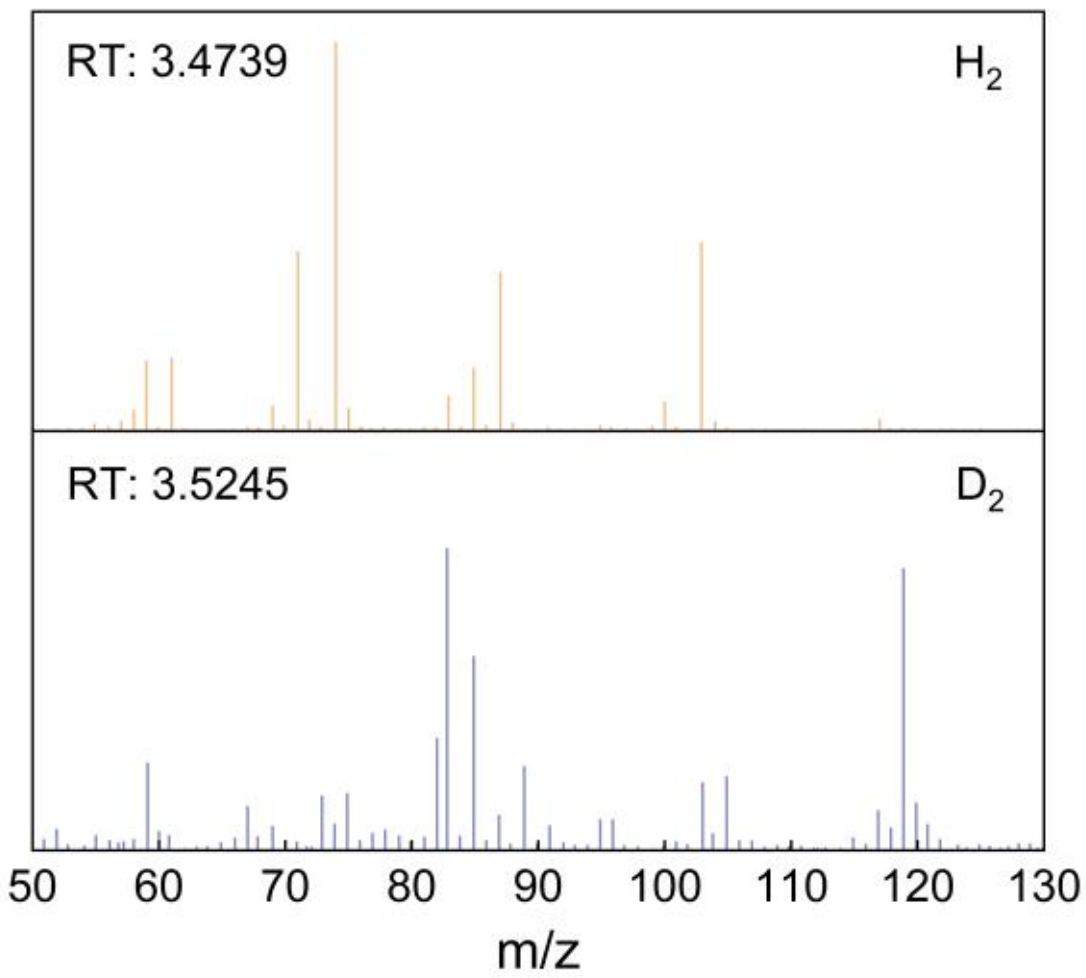

Fig. S25. Comparison of GC–MS spectra of PHB extracted from *C.N*@ISA cultivated with H_2 (top) or D_2 (bottom) supply.

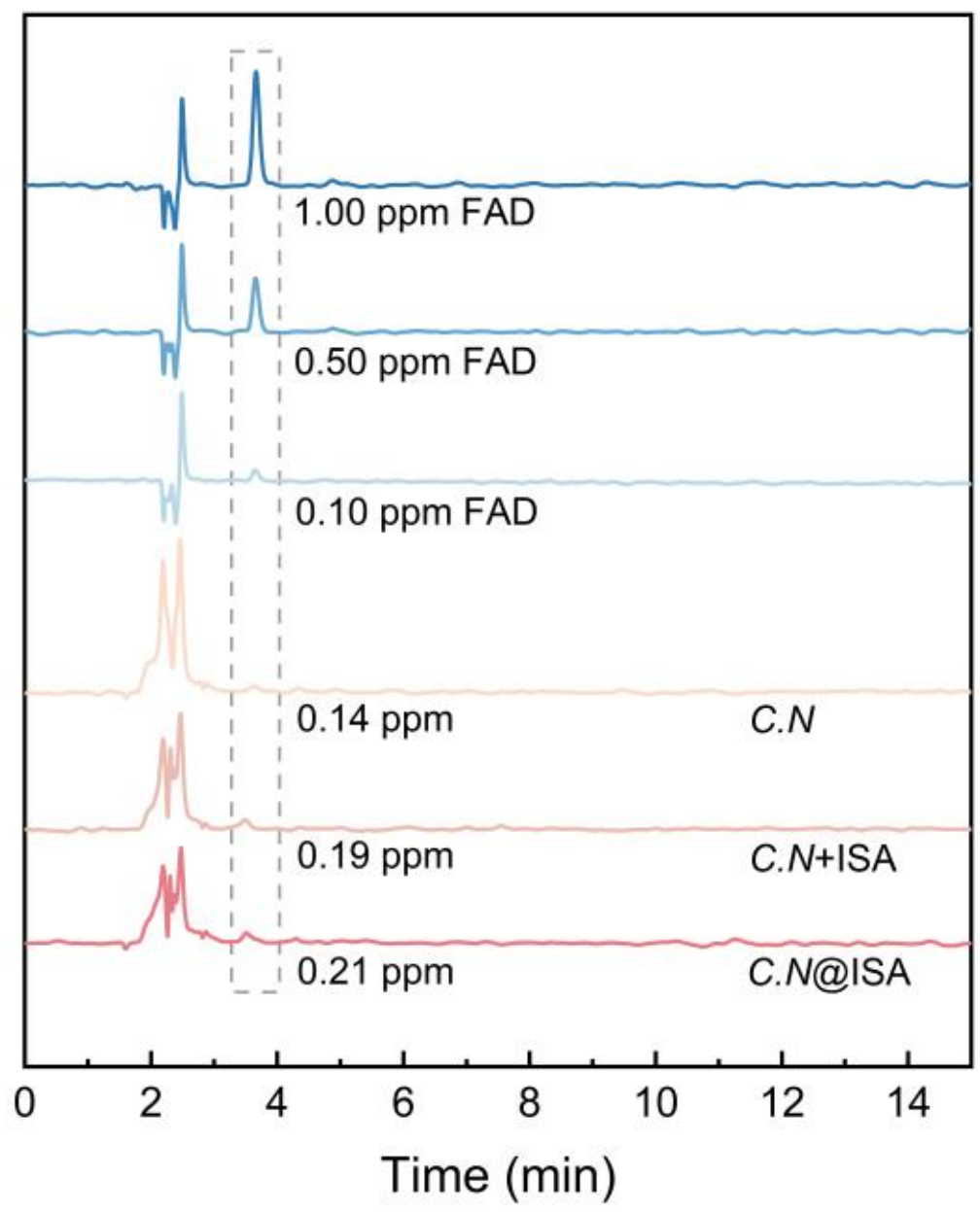

Fig. S27. Liquid chromatographic peak signal and calibration curve of FAD.

2. *The authors demonstrate excellent electrochemical stability of ISA, but the long-term stability and reusability of the C.N@ISA biohybrid under continuous operation are not thoroughly evaluated. Data on multiple batch cycles or extended operation would be valuable.*

Response:

We sincerely thank the reviewer for raising this important point regarding the operational reliability of the *C.N*@ISA biohybrid. We agree that demonstrating the stability of the hybrid under extended cultivation is essential for evaluating its practical applicability. In response, we conducted an extended fed-batch operation experiment and scanning electron microscopy characterization of the bacterial morphology after the prolonged operation, and the results have been incorporated into Fig. S40 of the revised manuscript.

In this test, the *C.N*@ISA hybrid was operated continuously for 168 hours, after which fresh medium was added to replenish essential nutrients. The hybrid then continued to function stably for an additional 192 hours, resulting in a cumulative operational period of 360 hours (approximately 15 days). Throughout this extended duration, the biohybrid maintained robust metabolic activity with no apparent decline in catalytic performance. Morphological characterization of the *C.N*@ISA biohybrid via scanning electron microscopy (SEM) after extended operation revealed that, although the process involves active bacterial growth and division, the surfaces of newborn bacteria remain persistently decorated with ISA. These observations provide compelling evidence that the inorganic–biological interface, engineered via click chemistry, remains highly robust and stable even under prolonged agitation.

Regarding the reviewer's question on "reusability", we wish to clarify that the current limitation arises from the nature of the product (PHB) rather than the stability of the hybrid system itself. PHB is an intracellular biopolymer that accumulates to approximately 46% of the

dry cell weight in our system. Its extraction inherently requires cell lysis, which renders the specific cell biomass non-reusable for subsequent cycles. However, our new 360-hour experimental data clearly demonstrates that the *C.N*@ISA hybrid possesses the structural and functional stability required for long-term operation. Thus, while the specific application of PHB production restricts cellular recycling, the hybrid platform itself exhibits exceptional engineering durability suitable for continuous or fed-batch processes. In scenarios involving extracellular or secretory products that circumvent the need for cell lysis or separation, the robust ISA-biohybrid interface facilitates direct recycling and sustained operational stability within the catalytic system.

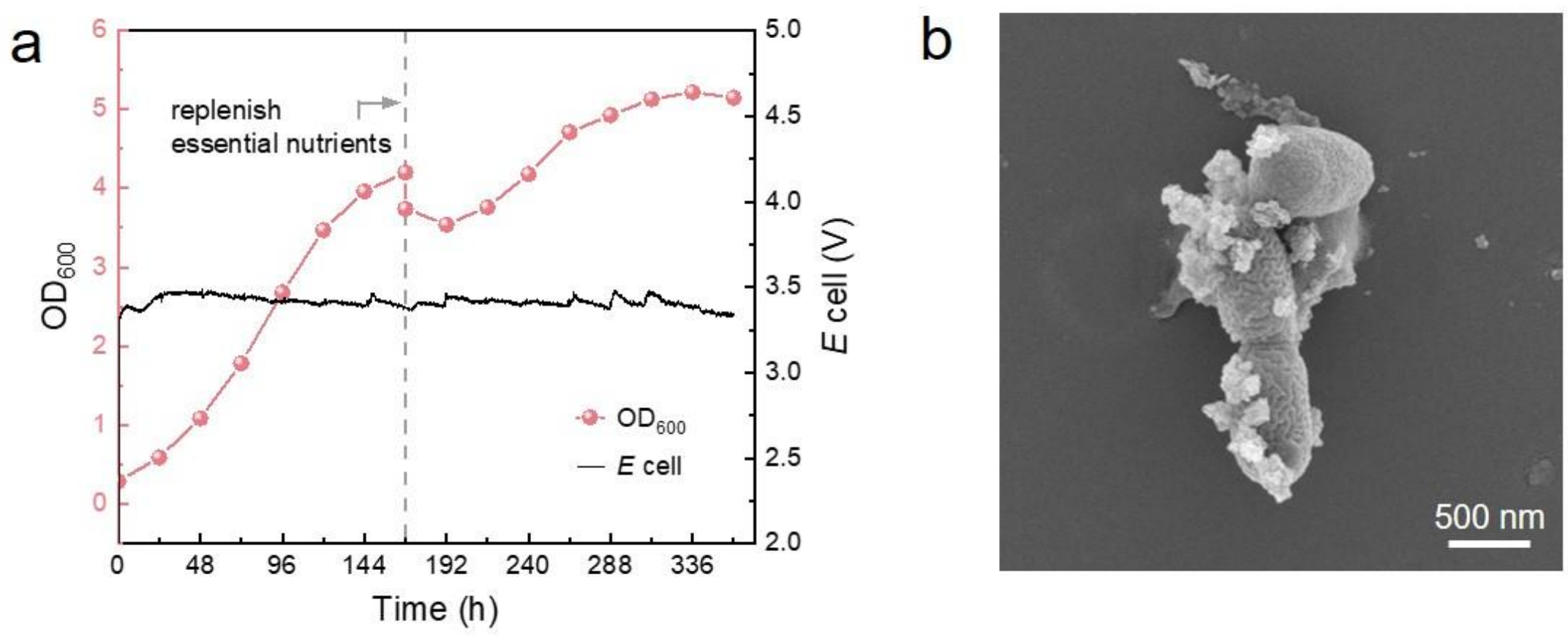

Fig. S40. Long-term stability evaluation of the *C.N*@ISA hybrid system. (a) Continuously monitored OD_{600} and reactor anode-cathode voltage during long-term operation. (b) SEM images of *C.N*@ISA from the integrated system after long-term operation.

3. *<u>The CLSM and Raman data suggest selective binding of B-PEG/ISA to α-sites of polysaccharides. However, the possibility of non-specific binding or interference with membrane proteins should be further addressed, such as via competitive binding assays or mutant strains lacking specific polysaccharide motifs.</u>*

Response:

We sincerely thank the reviewer for this constructive suggestion. To further validate the binding specificity of the B-PEG/ISA to the bacterial cell wall and rule out non-specific physical adsorption or membrane protein interference, we performed a competitive binding assay using D-fructose as a molecular blocker.

The phenylboronic acid (PBA) groups on B-PEG are designed to form covalent bonds with *cis*-diol-containing motifs, such as the polysaccharides present on the surface of *C. necator* (*C.N*). D-fructose is a well-known competitive inhibitor that exhibits an exceptionally high affinity for PBA, significantly higher than that of most cell-surface polysaccharides. By pre-incubating B-PEG/ISA with an excess of D-fructose, the active binding sites on the PBA moieties should be "masked", thereby preventing specific attachment to the bacteria if the mechanism is indeed boronic acid-mediated.

As shown in the newly provided SEM images (Fig. S19), in stark contrast to the original *C.N*@ISA hybrid where the catalyst uniformly coats the bacterial surface, the D-fructose-treated B-PEG/ISA failed to anchor onto the *C.N* cells. The ISA particles were observed as isolated, irregular clusters and aggregates dispersed on the substrate, completely detached from the bacterial cell bodies. These morphological results provide direct evidence that the assembly of the *C.N*@ISA hybrid is driven by specific boronic acid-saccharide interactions. If the binding were primarily driven by non-specific forces (such as hydrophobic interactions, electrostatic attraction, or non-specific protein binding), the addition of D-fructose would not have been able to so effectively block the hybrid formation.

The competitive inhibition by D-fructose successfully "turned off" the anchoring process, confirming that the B-PEG/ISA selectively targets the polysaccharide motifs on the cell wall. We have included these SEM images and a detailed discussion of the competitive assay in the

revised version (Section 2.2, Fig. S19) to address the reviewer's concern regarding binding specificity.

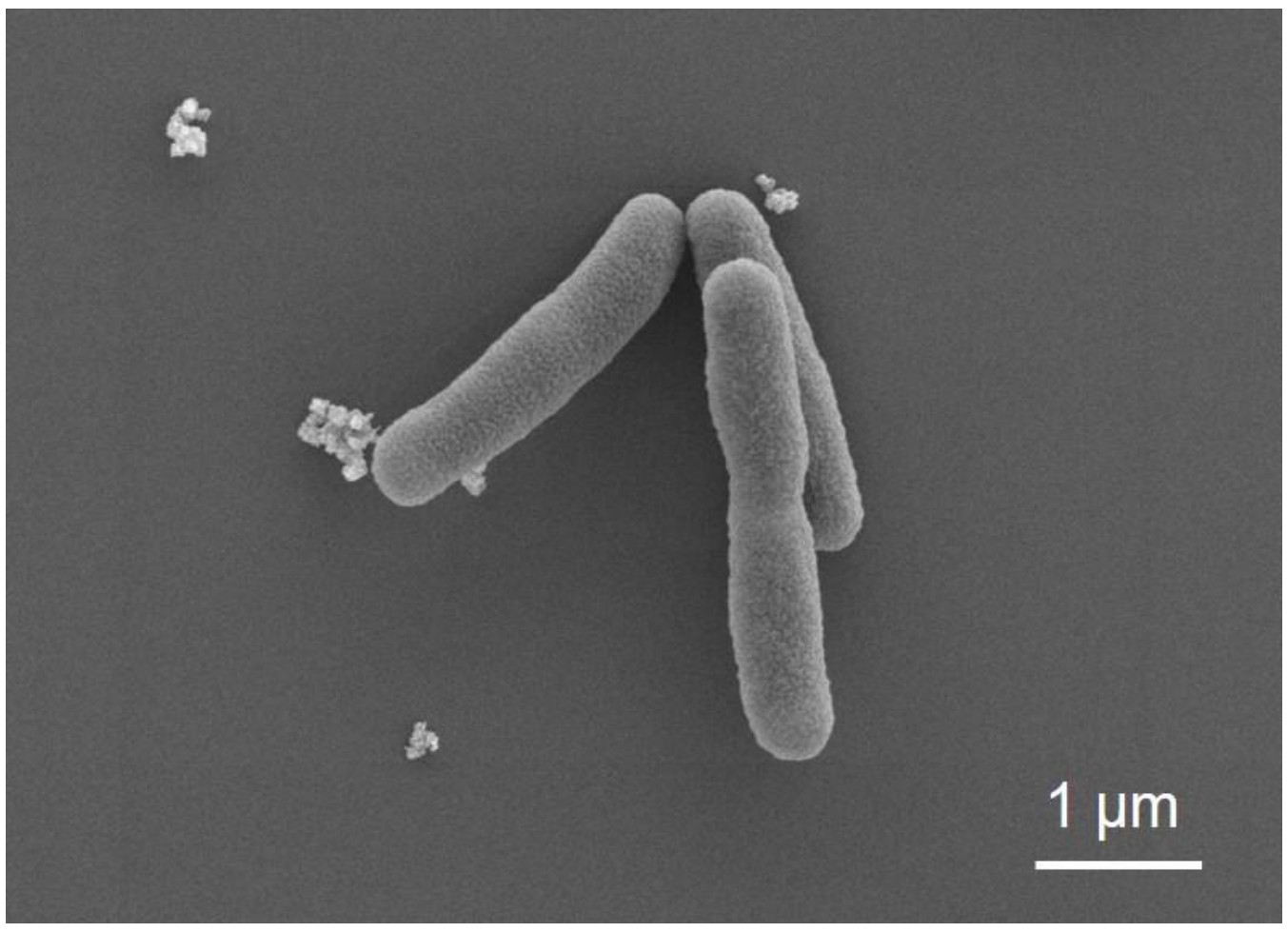

Fig. S19. SEM image of cells from the fructose competitive binding assay (Binding sites of B-PEG/ISA are occupied by D-fructose).

4. *The ROS scavenging data are supportive but qualitative. Quantification of intracellular ROS levels and correlation with cell viability under operational conditions would provide more direct evidence of the protective role of ISA.*

Response:

We sincerely appreciate and fully agree with the reviewer’s insightful comment. In response, we performed additional quantitative intracellular ROS measurements under operational conditions using the DCFH-DA fluorescent probe. Additionally, we determined cell viability using dual staining with SYTO 9 nucleic acid stain and propidium iodide (PI).

Our measurements show that *C. necator* cells with ISA anchored on the membrane (*C.N*@ISA) exhibit substantially reduced intracellular ROS accumulation, with DCFH-DA fluorescence decreasing by approximately 20–50% relative to cells without ISA anchoring (Fig. S35). This result indicates that ISA confers a pronounced cytoprotective nanozyme-like effect,

mitigating intracellular oxidative stress during H_2-driven biotransformation. To directly link this reduction in ROS to cellular robustness, we simultaneously quantified cell viability under identical operational conditions. The results showed that the cell viability of the *C.N*@ISA group was higher than that of the *C.N* group, demonstrating that the *C.N*@ISA group maintains significantly higher metabolic integrity relative to the control group. Together, these quantitative datasets establish a clear correlation: ISA anchoring lowers intracellular ROS levels and simultaneously preserves cell viability during electrosynthetic operation.

The resulting dataset has been incorporated into Fig. S35 and Fig. S36 of the revised Supplementary Information. We have integrated these findings into the revised manuscript and expanded the discussion to clarify that the ISA component not only mediates H_2 activation and electron supply but also provides a cytoprotective nanozyme-like function that stabilizes intracellular redox homeostasis. This added evidence directly addresses the reviewer's concern and substantially strengthens the mechanistic understanding of how ISA enhances cellular performance within the hybrid system.

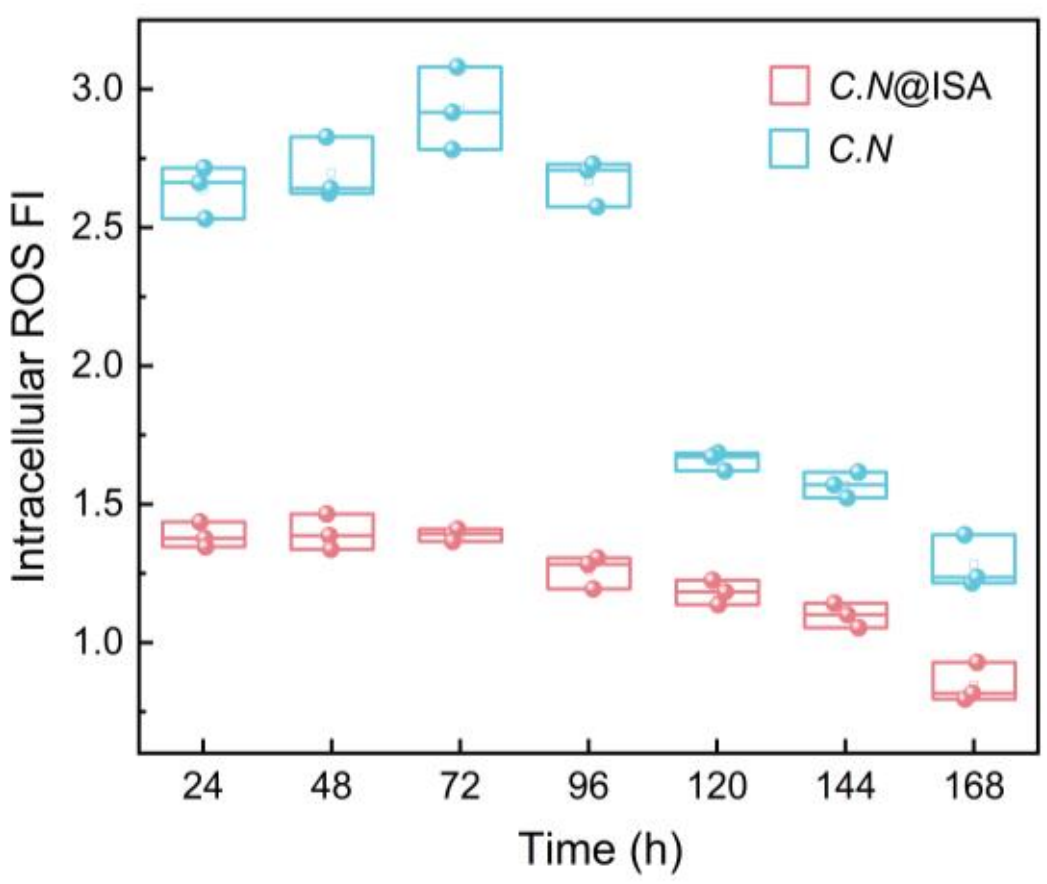

Fig. S35. Intracellular ROS fluorescence intensity (FI) during the operation.

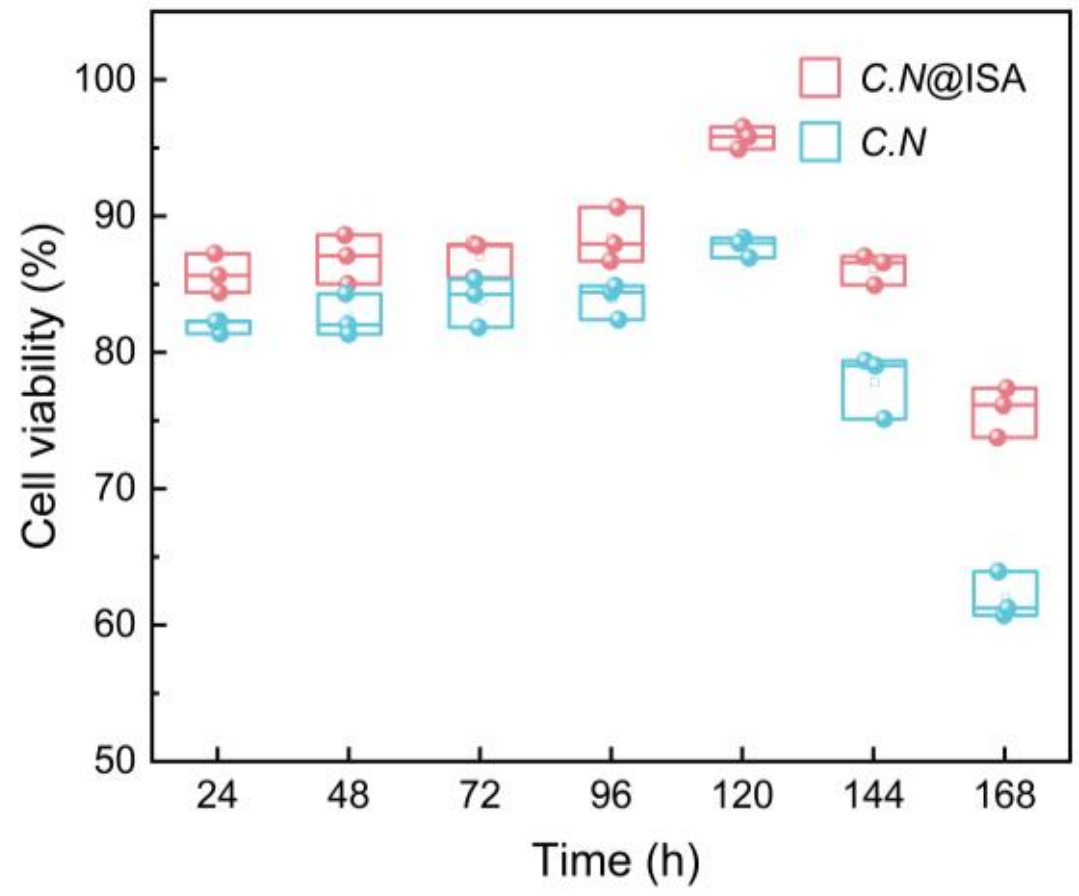

Fig. S36. Cell viability during the operation.

5. *The discrepancy between the theoretical and experimental FE values, especially for C.N, warrants further discussion. Are there other electron sinks or metabolic side reactions not accounted for in the model?*

Response:

We thank the reviewer for this critical question regarding the electron balance. We agree that the discrepancy between the theoretical Faradaic Efficiency (FE_T: ~58.8% for native *C.N*) and the experimental value (FE) warrants a rigorous and transparent discussion. In addressing this point, and incorporating a related methodological suggestion from Reviewer 3, we have revised our approach to biomass quantification. We have re-calibrated the biomass and PHB coefficients specifically for our hybrid system following the methodology of Liu et al. (*Science*, **352**, 1210-1213 (2016)). By analyzing early-stage and late-stage samples, we derived system-specific coefficients that decouple the optical contributions:

- Biomass Coefficient: 401 mg biomass/L/$OD_{biomass}$
- PHB Scattering Coefficient: 785 mg PHB/L/OD_{PHB}

Based on these re-calibrated parameters, we established a new calculation formula:

$$c_{\mathrm{biomass}}\ (\mathrm{mg/L}) = (\mathrm{OD}_{\mathrm{total}} - c_{\mathrm{PHB}}/785) \times 401$$

where c_{PHB} is the concentration determined by HPLC. By applying this more accurate formula, the experimental FE for the *C.N*@ISA system is now corrected to 42.0%. Regarding the remaining gap between the experimental FE (42.0%) and the theoretical limit (~53.3% for *C.N*@ISA), we attribute it to two primary electron sinks: Physical H_2 loss and unaccounted metabolic consumption.

For the native *C.N* system, the dominant sink is the physical escape of H_2 gas due to its low solubility (~1 mM) and the lack of an efficient interface, which allows a significant portion of the H_2 generated at the electrode to diffuse into the headspace before it can be captured by the suspended bacteria. In contrast, the *C.N*@ISA system creates a localized, high-concentration H_2 microenvironment directly at the cell surface, drastically reducing this physical loss. Additionally, the theoretical model assumes that all assimilated electrons are directed toward biomass and PHB synthesis. This assumption neglects the metabolic energy required for stress responses and essential non-growth-associated maintenance. Specifically, non-growth-associated maintenance encompasses basal energetic costs such as maintaining transmembrane ion gradients, repairing damaged cellular components, and sustaining macromolecular turnover. As detailed in our response to Point 4, native *C.N* cells experience high intracellular ROS levels, which forces the diversion of reducing equivalents (NADH/NADPH) toward detoxification pathways rather than PHB synthesis. In contrast, the dual ability of the ISA-anchored biohybrid to mitigate physical H_2 mass transfer limitations and reduce metabolic diversion allows the *C.N*@ISA system to significantly narrow the efficiency gap, achieving an experimental FE of 42.0% compared to the theoretical 53.3%. We have updated the Section 2.6 to explicitly outline these electron sinks.

Reviewer #2 (Remarks to the Author):

This manuscript presents an exceptionally innovative and systematic study that establishes a new paradigm for inorganic–biological hybrid systems. The authors have successfully engineered a microbial interface by covalently anchoring iron single-atom catalysts (ISA) onto the membrane of Cupriavidus necator, enabling highly efficient CO_2-to-PHB conversion. The work introduces an original concept of "single-atom catalysis–microbial membrane interface engineering," offering a transformative enhancement strategy for H_2-mediated electromicrobial synthesis. The innovation of this work lies in its elegant integration of single-atom catalysis with microbial metabolism through precise interface design. The ISA functions as an artificial hydrogenase to create a localized H_2-rich and proton-rich microenvironment, simultaneously facilitating electron/proton transfer and mitigating reactive oxygen species. This dual functionality significantly strengthens bioenergetic coupling and metabolic flux toward CO_2 fixation. The research achieves benchmark-level PHB production and Faradaic efficiency, far exceeding most previously reported systems. Scientifically, the manuscript offers a new conceptual and technical framework for addressing two long-standing bottlenecks—limited H_2 bioavailability and sluggish hydrogenase kinetics—in electromicrobial CO_2 utilization. From the perspective of potential impact, this work could serve as a cornerstone for future carbon-negative biomanufacturing strategies coupling renewable energy with microbial catalysis.

Overall, the manuscript is logically structured, experimentally comprehensive, and methodologically rigorous. The figures are of high quality and clearly support the authors' conclusions. The combination of advanced characterization (HAADF-STEM, XANES/EXAFS, EIS, CLSM), theoretical DFT calculations, and transcriptomic analysis demonstrates impressive depth and technical sophistication. The study is complete and compelling, and the conclusions are well supported by solid experimental evidence. I therefore recommend

publication after minor revisions.

Response:

We genuinely appreciate Reviewer #2's exceptionally positive assessment, recognizing our work as a "new paradigm" and a "cornerstone" for future carbon-negative biomanufacturing. We are deeply encouraged by the endorsement of our "single-atom catalysis–microbial membrane interface engineering" strategy and the comprehensive experimental design. We have carefully addressed the suggested revisions to further refine the manuscript and ensure it meets the highest scientific standards.

Comments and Questions

1. *The iron signal in the EDS of Figure S1 is not very obvious. Are there higher-resolution images, such as HAADF-STEM EDS mapping, to further confirm the presence and distribution of iron species?*

Response:

We sincerely appreciate the reviewer's professional suggestion to further clarify the dispersion of iron species. In response, we have performed high-resolution HAADF–STEM coupled with EDS mapping.

As shown in the updated Fig. S2, the HAADF image and corresponding elemental maps clearly demonstrate that the Fe signals are uniformly and atomically dispersed across the nitrogen-doped carbon matrix. Importantly, no bright clusters or nanoparticle aggregations were observed, providing direct visual evidence for the single-atom nature of the catalyst. This result is in excellent agreement with our EXAFS analysis, which identifies the predominant Fe−Nx coordination. We believe this high-resolution imaging significantly strengthens the evidence for the atomic dispersion of our ISA sites.

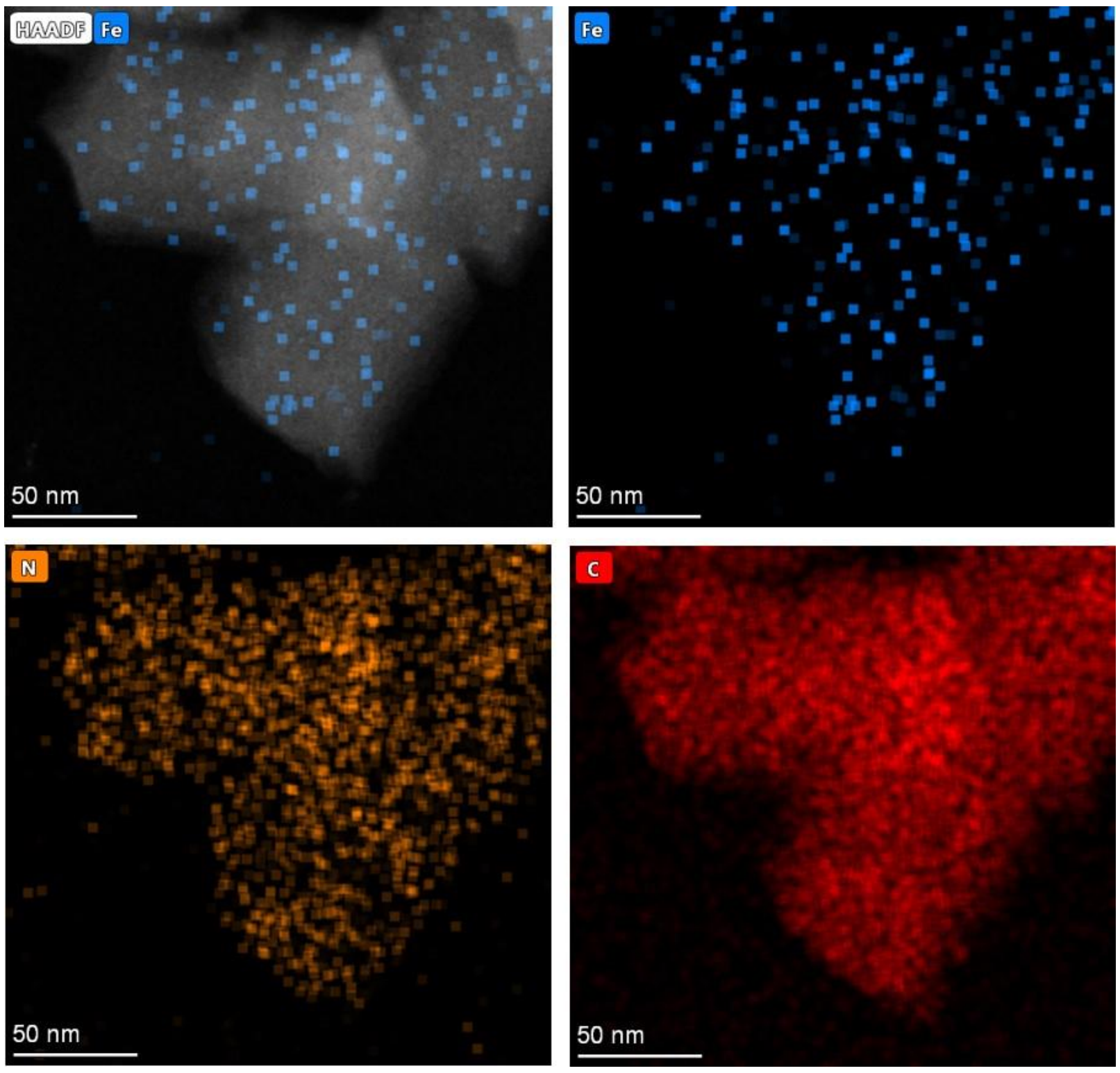

Fig. S2. Dark-field STEM image and the EDS elemental mappings for ISA.

2. *The manuscript includes several control materials (e.g., Fe-free and different calcination temperatures). How did the authors determine the optimal ISA configuration (Fe-N_4pyd-C) for subsequent experiments? Were other possible Fe coordination structures considered in the DFT screening?*

Response:

The identification of Fe–N_4pyd–C as the optimal configuration was established through a rigorous systematic workflow combining experimental optimization, structural characterization, and theoretical screening. Initially, we synthesized a series of control materials by modulating the pyrolysis temperature of the Fe–ZIF8 precursor. As detailed in Section 2.1 and Figs. S3–S10, the pyrolysis temperature plays a critical role in regulating the Fe content, coordination environment, and the degree of graphitization. We evaluated these materials based on their physicochemical properties, particularly electrical conductivity (Fig. S14), which is vital for

the bio-inorganic hybrid interface. The sample obtained at the optimal temperature exhibited superior conductivity and structural integrity, and was thus selected as the optimal ISA material for subsequent biological integration.

Subsequently, we performed advanced structural characterizations on this optimized material to determine the precise coordination environment of the iron sites. Both XPS and EXAFS fitting analyses indicate that the Fe centers are predominantly coordinated with pyridinic nitrogen species, with an average coordination number close to four. These experimental findings provided the necessary structural constraints for our theoretical models.

Guided by these experimental results, we constructed and evaluated multiple plausible coordination structures in our DFT calculations to pinpoint the active center, specifically comparing Fe–N_4pyd–C (pyridinic), Fe–N_4pyr–C (pyrrolic), and Fe–N_3pyd–C (unsaturated). Our calculation results revealed that Fe–N_4pyd–C exhibits the most favorable balance between H_2 adsorption strength and the activation energy barrier. In contrast, the control models showed significant limitations: Fe–N_3pyd–C is prone to catalyst poisoning due to its excessively strong binding affinity for H_2, while Fe–N_4pyr–C shows insufficient capability for H desorption. Consequently, the Fe–N_4pyd–C configuration was determined to be the optimal and physically realistic active site, supported by conclusive experimental spectroscopic evidence and theoretical kinetic/thermodynamic verification.

3. *In the theoretical section, how was it determined that H_2 adsorption occurs primarily at the Fe atom? Please also provide the Bader charge of the Fe site and its neighboring atoms. Moreover, adsorption energies were calculated for three models, yet energy barrier calculations were performed for only two—please clarify the reason for this selection.*

Response:

Thank you for your valuable comments. In our previous calculations, multiple initial adsorption configurations were tested, including H_2 adsorption on Fe sites, adjacent N atoms, and carbon basal planes. In all cases, geometry optimization converged to configurations where H_2 preferentially binds to the Fe center, indicating that Fe serves as the dominant adsorption and activation site.

As illustrated by the differential charge density map and Bader charge analysis (Fig. S21), the Fe center exhibits a significant electron deficiency, primarily attributed to the strong electron-withdrawing effect of the coordinating pyridine nitrogen atoms, which causes the Fe species to reside in a high oxidation state (nominally Fe^{2+}/Fe^{3+}). Such an electron-deficient Fe center possesses a strong affinity for the σ-bonding electrons of H_2, thereby facilitating H_2 adsorption and subsequent activation. Furthermore, the electronic coupling between the Fe center and the carbon framework provides a fast lane for electron shuttling, ensuring the rapid turnover of the catalytic cycle.

In our adsorption energy calculations, the Fe-N_3pyd-C configuration exhibited an excessively strong H_2 adsorption energy of -0.57 eV. According to the Sabatier principle, such a deep potential well leads to hydrogen poisoning of the active sites, as corroborated by recent literature (*Nano Energy* **118**, 109038 (2023)), thereby hindering the subsequent catalytic turnover. Due to this prohibitive adsorption strength, the Fe−N_3pyd−C site was excluded from further transition-state (TS) analysis. In contrast, Fe−N_4pyd−C and Fe−N_4pyr−C presented moderate adsorption energies, providing a balanced energetic profile for evaluating the kinetic barriers of H_2 dissociation.

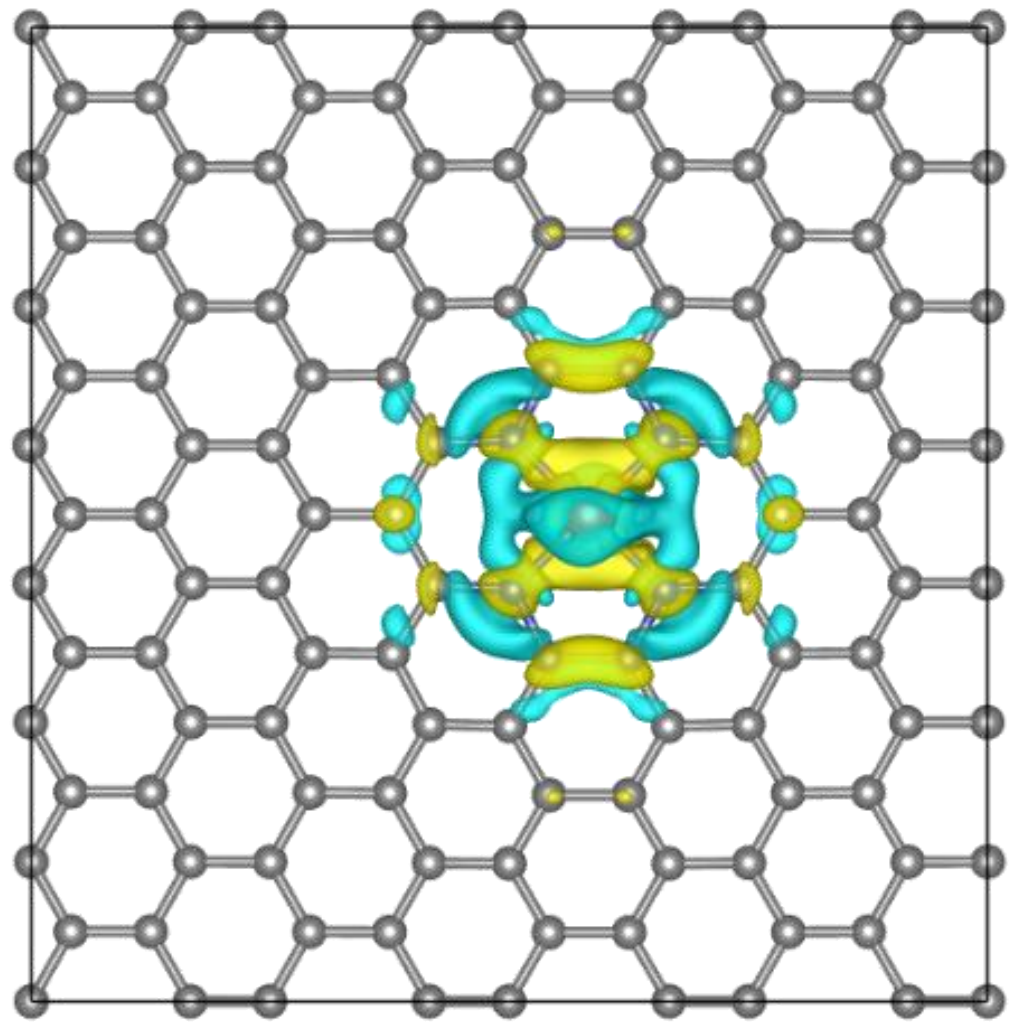

Fig. S21. Differential charge density diagram of Fe-N4pyd-C. Yellow indicates the accumulation of charge, while blue represents the depletion of charge.

4. *What is the intrinsic conductivity of the B-PEG linker used, and how does it quantitatively contribute to promoting electron/proton transfer? Since extracellular polysaccharides are typically insulating, could the anchoring of B-PEG/ISA to such substrates partially limit charge transport?*

Response:

We thank the reviewer for this insightful question regarding the electrical conductivity of the B-PEG linker. We acknowledge that B-PEG is a poly(ethylene glycol) derivative with negligible intrinsic conductivity (typically 10^{-10} to 10^{-12} S/cm). However, the contribution of the B-PEG linker is not to serve as an ohmic conductor, but rather to act as a precision spatial anchor. By covalently tethering the ISA in the immediate vicinity of the microbial cell membrane via click chemistry, the linker maintains the catalytic centers at a nanoscopic distance from the cell surface. This strategic positioning addresses the H_2 mass transfer limitations inherent in bulk biohybrid systems, by enabling a transition from passive bulk diffusion to an

active interfacial capture mechanism.

Although extracellular polysaccharides (EPS) and B-PEG are non-conductive, they do not obstruct energy flux in our system because the transfer of reducing equivalents is mediated by diffusible chemical species that readily permeate through these molecular matrices. To further substantiate the mechanism of charge transport across this interface, we performed supplementary cyclic voltammetry (CV) tests on bacterial suspension from the operating integrated system and conducted high-performance liquid chromatography (HPLC) analysis on the supernatant after oscillation treatment of the suspension. These investigations successfully detected flavin adenine dinucleotide (FAD), a self-secreted redox mediator (Fig. S27). This result confirms that the electron transfer is facilitated by a mediated electron transfer pathway, which remains highly efficient even across non-conductive PEG or EPS matrices due to the rapid permeation of FAD. By establishing a localized high-concentration H_2 and FAD mediated electron transfer at the cellular periphery, we significantly minimize mass transfer resistance and maximize productivity. We have incorporate these discussions in the revised section 2.4.

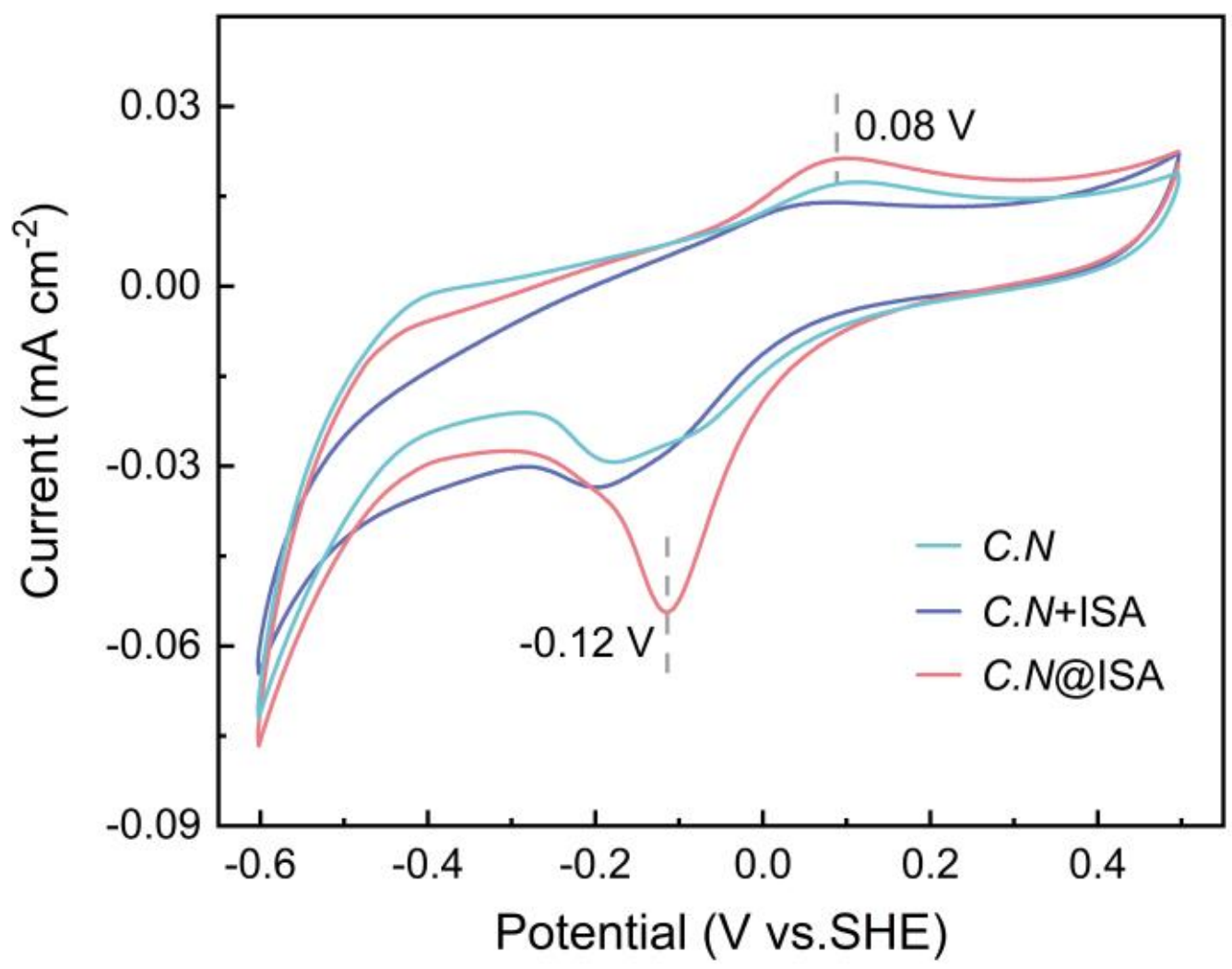

Fig. 4f. CV curves of the operating integrated system. *C.N*@ISA, *C.N*+ISA, *C.N* are all suspended in the electrolyte.

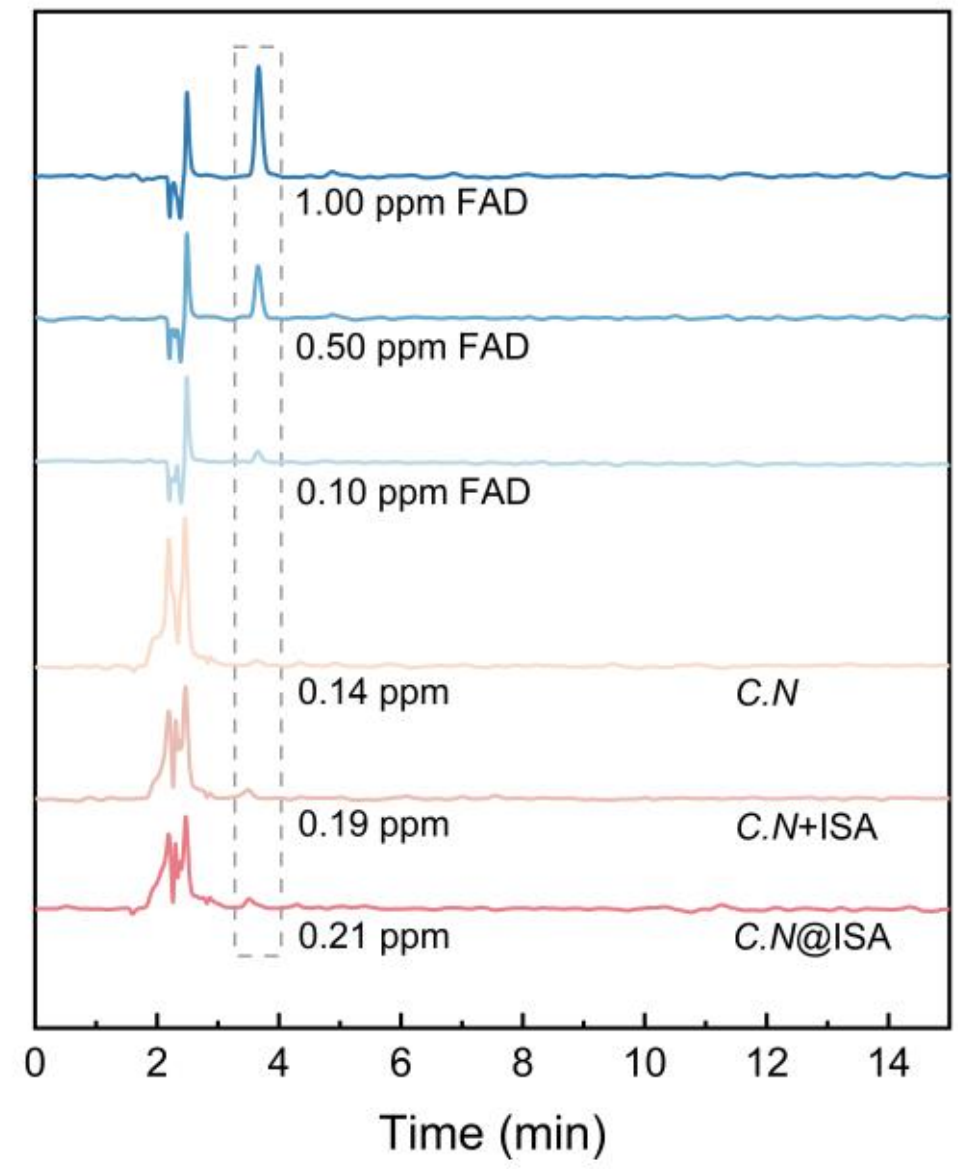

Fig. S27. Liquid chromatographic peak signal and calibration curve of FAD.

5. *In the electrochemical setup, how did the authors experimentally simulate the H_2-derived electron supply using electrical current? Please clarify the mechanistic connection between current density and biological electron uptake.*

Response:

We thank the reviewer for this question regarding the relationship between electrochemical parameters and biological activity. In our setup, the electrical current is not a simulation but the direct stoichiometric driver for the generation of reducing equivalents, primarily through the hydrogen evolution reaction (HER). Within an appropriate potential range, the rate of H_2 generation is directly proportional to the applied current. While pioneering systems such as that described by Liu et al. ((*Science*, **352**, 1210-1213 (2016)) demonstrated that biomass accumulation scales linearly with the amount of charge passed when H_2 generation is the rate-limiting step, our work focuses on the nanoscopic mechanistic connection between electron

carrier (H_2) and biological electron uptake at the interface.

Although *C. necator* secretes certain redox mediators such as FAD, their concentration is relatively low (on the order of 0.1 ppm). The current density associated with the redox transitions of these electron mediators is negligible compared to the HER current. Consequently, the current density primarily governs the flux of H_2 evolution. By anchoring the ISA directly to the microbial membrane, we establish a localized H_2-enriched microenvironment where H_2 molecules are effectively captured and utilized by the bacteria. This minimizes the loss of reducing equivalents to the headspace. Consequently, the current density in our system acts as a tunable "throttle" for the interfacial supply of H_2, which in turn drives CO_2 fixation. The localized capture mechanism established by the anchored ISA prevents the mass transfer bottlenecks typical of non-hybridized suspension cultures, thereby ensuring that the high current densities we apply are efficiently translated into record-high volumetric productivity of PHB. Therefore, while the electrical current in our system is indirectly related to the rate of biological electron (reducing equivalent) uptake, the strategic modification of the bacteria with ISA significantly strengthens the correlation between the applied current and the rate at which the bacteria acquire energy.

6. *In Figure 4h, $K_3Fe(CN)_6$ is used as a redox mediator. Please elaborate on the rationale for this selection and whether similar effects could be achieved with other mediators.*

Response:

Thank you very much for your valuable comments. We selected $K_3Fe(CN)_6$ as the redox mediator primarily because it is a well-established, rapid-response probe characterized by high electrochemical reversibility, fast electron-transfer kinetics, and excellent chemical stability in neutral aqueous media.

Upon the addition of $K_3Fe(CN)_6$, an increase in current was observed. The bare carbon cloth electrode exhibited the lowest current increment, indicating its limited intrinsic catalytic activity toward the reduction of $[Fe(CN)_6]^{3-}$. In contrast, electrodes modified with ISA or *C.N* showed higher current responses, suggesting that these components may provide additional redox-active sites that facilitate the reduction of $[Fe(CN)_6]^{3-}$. Notably, compared with the other groups, the *C.N*@ISA group displayed a markedly higher current response, which may be attributed to electron uptake by the cells. Efficient electron transfer relies on the functionalized interface uniquely present in the *C.N*@ISA group. Therefore, the largest current response observed for the *C.N*@ISA group reflects a mediator-assisted electron transfer process, supporting the enhanced electron-transfer kinetics enabled by the engineered interface. These discussions have been incorporated in revised section 2.3.

While other soluble redox mediators—such as humic acids—could in principle also shuttle electrons, their heterogeneous electron-transfer kinetics are generally slower. Moreover, humic acids may be actively taken up or metabolically utilized by bacteria, or may bind nonspecifically to membrane proteins. These interactions introduce confounding effects, making it difficult to decouple pure interfacial electron-transfer kinetics from biological uptake or metabolism. Additionally, organic mediators often exhibit poor stability, potentially undergoing side reactions or adsorption, which complicates data interpretation. Therefore, although other mediators could theoretically be used to demonstrate electron shuttling, $K_3Fe(CN)_6$ was selected for its optimal combination of electrochemical reversibility, fast kinetics, chemical stability, and minimal interference with biological systems.

7. *The validation of membrane potential is very interesting. Please describe the detailed operating conditions (e.g., dye concentration, incubation time). The authors also mention*

<u>the proton motive force (PMF); could the relative contributions of membrane potential and proton gradient to PMF be quantitatively compared?</u>

Response:

Thank you for your valuable comments. The membrane potential was measured as follows: Bacterial suspensions from different groups (*C.N*, *C.N*+B-PEG, *C.N*+ISA, and *C.N*@ISA, with an OD600 of approximately 0.1) were incubated with 4 μM diethyloxacarbocyanine iodide ($DiOC_2(3)$) at 37 °C for 15 min to allow dye loading. The suspensions were then gassed with H_2 for 15 min. A negative control was included by adding the protonophore CCCP (60 μM, 5 min incubation) between the dye loading and H_2 exposure steps. Fluorescence intensity was measured using a microplate reader (Varioskan Lux, Thermo Fisher) at excitation/emission wavelengths of 484 nm/525 nm (green) and 484 nm/610 nm (red). Membrane potential was quantified as the ratio of red fluorescence (610 nm) to green fluorescence (525 nm).

The proton motive force (PMF) can be expressed as:

$$\Delta p = \Delta\psi - (2.303RT/F)\,\Delta pH$$

where $\Delta\psi$ is the membrane potential and ΔpH is the transmembrane proton gradient. 2.3RT/F ≈ 60 mV at 30 °C.

From the PMF equation, $\Delta\psi$ and ΔpH are two orthogonal components of the proton motive force. While both components contribute to the energetic state of the cell, we focused on $\Delta\psi$ because it typically constitutes the dominant component of the PMF in neutral-pH biological systems (ΔpH ≈ 0.4~0.6) like our reactor environment. More importantly, from a mechanistic standpoint, the interfacial proton generation enabled by the ISA directly induces an immediate charge separation at the membrane surface. In our hybrid system, the “hard-wired” ISA–membrane interface ensures that dissociated protons are delivered to the juxtamembrane region, where they first contribute to a localized positive charge excess, thereby manifesting primarily

as an increase in $\Delta\psi$. Although $\Delta\psi$ and ΔpH are dynamically coupled and difficult to measure simultaneously in real-time without perturbing the system, the significant enhancement in $\Delta\psi$ observed in the *C.N*@ISA group provides a sensitive and representative indicator of the intensified PMF. This localized enrichment of reducing equivalents directly drives ATP synthesis and metabolic flux, which correlates with the observed record-high PHB production.

8. *Please describe how dry cell weight was measured. Considering that ISA is attached to the membrane, could this additional mass cause a slight overestimation in the dry weight, thereby affecting PHB/DCW calculations?*

Response:

Thank you for your valuable comments. In our study, the dry cell weight (DCW) was not determined by direct weighing for each individual sample. Instead, DCW was estimated using an empirical calibration curve established in preliminary experiments, which correlates optical density at 600 nm (OD_{600}) with experimentally measured dry cell weight. Briefly, cells were harvested at different OD_{600} values, washed, dried to constant weight, and used to construct a standard OD–DCW calibration curve (Added to revised Supplementary Method 7). This calibration was then applied throughout the study to calculate DCW from the measured OD_{600} values using the corresponding empirical equation. Therefore, the DCW values reported in this work are derived from OD measurements rather than direct gravimetric determination for each sample. Additionally, the attachment of ISA to the cell membrane was found to have a negligible effect on OD_{600}, as evidenced by the comparable optical densities of the bacterial suspension before and after the addition of B-PEG/ISA. Consequently, ISA attachment does not lead to a noticeable overestimation of DCW, and thus has a negligible impact on the calculated PHB/DCW values.

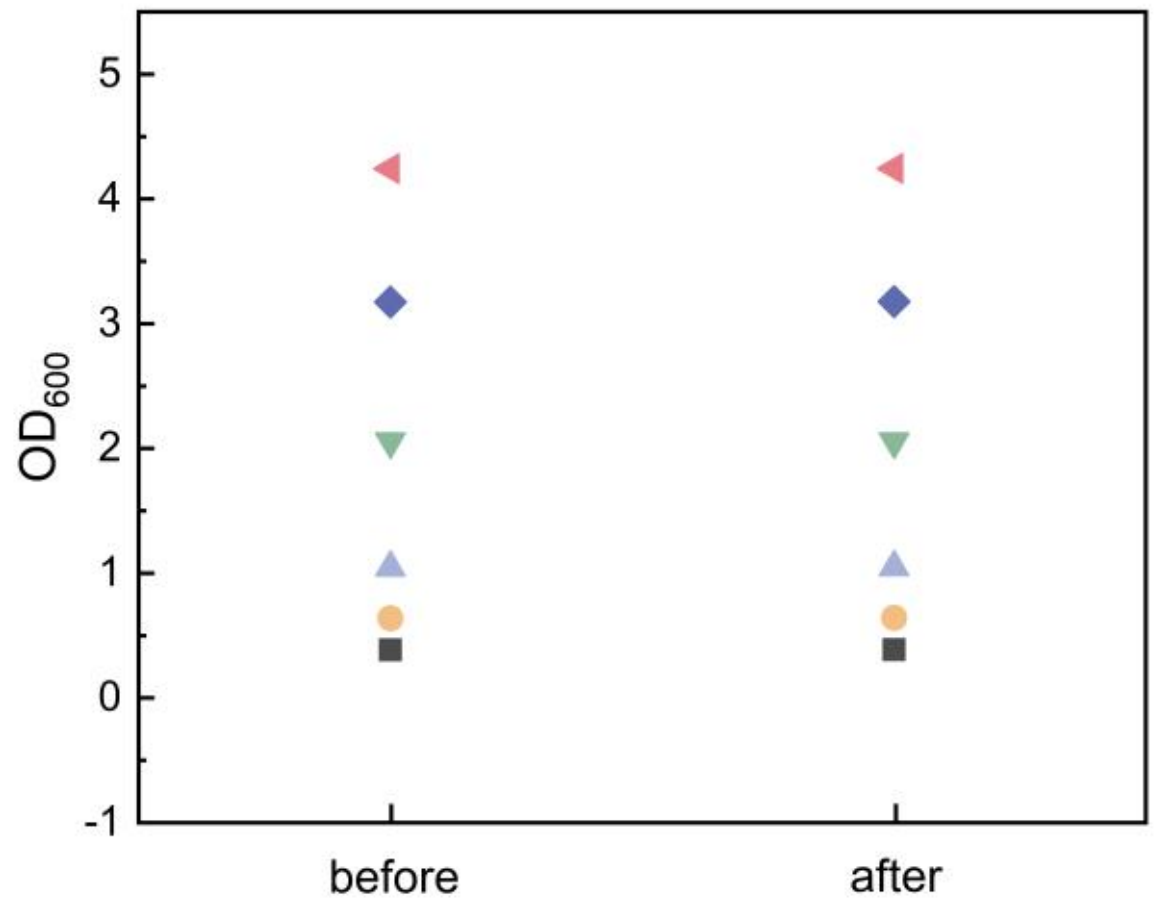

Fig. R1. Optical density at 600 nm (OD_{600}) of bacterial suspensions before and after the addition of B-PEG/ISA (12.5 mg L^{-1}) across different bacterial concentrations.

9. *Regarding Faradaic efficiency, what is the exact physical meaning of FE and FE_T in Fig. 6, and how are they correlated? The text suggests that FET decreases for ISA-modified cells, but the experimental FE increases—please clarify this apparent inconsistency.*

Response:

We appreciate the reviewer's acute observation regarding the relationship between experimental Faradaic efficiency (FE) and its theoretical limit (FE_T), as this apparent trade-off underscores the specific metabolic rewiring mechanism at play in our biohybrid system.

Regarding the physical definitions, the Experimental Faradaic Efficiency (FE) represents the actual percentage of electrons consumed by the biohybrid system that are ultimately recovered in the final product (PHB), thereby reflecting the real-world performance after accounting for all metabolic losses such as ROS generation and cell maintenance. The Theoretical Faradaic Efficiency (FE_T) defines the stoichiometric upper limit of electron conversion efficiency, which is determined strictly by the specific entry point of electrons into

the electron transport chain (ETC) and the resulting P/O ratio (ATP generated per electron pair), assuming ideal conditions without side reactions.

The apparent trade-off where FE_T decreases while experimental FE increases highlights the specific metabolic rewiring mechanism induced by the ISA modification. The decrease in FE_T (from 58.8% to 53.3%) is thermodynamically expected because ISA injects electrons directly into the quinone pool, bypassing Complex I; this shortens the proton-pumping pathway and lowers the P/O ratio from 2.5 to 2.0, necessitating a slightly higher fraction of electrons for ATP generation rather than PHB synthesis. However, the experimental FE significantly increases (from 26.0% to 42.0%) because the hybrid system drastically improves the actual electron utilization rate: while the native control suffers from severe energy losses and utilizes only ~44% of its theoretical potential, the *C.N*@ISA hybrid—aided by efficient interfacial electron transfer and ROS scavenging—realizes ~79% of its theoretical capacity. Thus, the kinetic benefits of reduced metabolic waste vastly outweigh the minor thermodynamic penalty of the bypassed proton pump.

Reviewer #3 (Remarks to the Author):

The authors presented a new route to link the single-atom iron to C. necator by a organic linker based on click reaction, which showed the enhancement of PHB production with FE about 44.6%. The possible mechanisms were postulated based on the electrochemical analysis.

However, besides the linkage, the novelty of the work is weak comparing to previous work, such as by Liu et al. (10.1126/science.aaf5039) etc. And the details of the evidence should also be checked as well as calculations based on empirical equations.

Response:

We sincerely appreciate the reviewer's positive evaluation of our click-chemistry-based

iron single-atom hybrid system and grateful for the reference to the pioneering work by Liu et al. (*Science*, **352**, 1210-1213 (2016)). We fully acknowledge that Liu et al. established the foundational paradigm of water splitting–biosynthetic systems. Their core contribution lay in developing biocompatible Co-P catalysts to overcome toxicity and ROS issues during hydrogen evolution, thereby proving the feasibility of hydrogen-mediated electron transfer.

Mechanistically, our system shares the same fundamental principle as Liu's, relying on hydrogen (H_2) as a diffusive electron carrier. However, our work is not a mere replication but represents a strategic shift from addressing "Hydrogen Production" to enhancing "Hydrogen Utilization". While Liu et al. focused on the biocompatibility of the H_2 -production side, our study tackles the bottleneck of H_2 uptake efficiency on the utilization side. By engineering the interface with iron single-atom catalysts (ISAs), we create a localized microenvironment enriched with H_2 directly at the cell surface. This design shifts the paradigm from passive diffusion in the bulk solution to active interfacial H_2 capture and utilization. Therefore, the novelty of our work lies in this functional leap—optimizing the microscopic mass transfer and metabolic flux to maximize the potential of biohybrid systems.

1. *For the calculation of biomass and energy in ECE and FE calculations, the cited equations and parameters were obtained by the measurement under similar conditions. But due to the cultivation conditions' changes and cell components varied a lot (such as PHB content, etc), it's better to use direct measurements, other than an empirical equation. If using the empirical equation, please refer to citations to adjust the parameter for different cultivation conditions.*

Response:

We greatly appreciate the reviewer's suggestion to verify the biomass quantification

method. We acknowledge that the empirical equation from literature may introduce errors due to differences in cultivation conditions and optical instruments. Furthermore, in our hybrid system, the presence of surface-anchored ISA precludes the use of simple gravimetric subtraction (DCW - PHB), as the weight of the inorganic catalyst would lead to an overestimation of the cellular biomass.

Therefore, to accurately quantify the active biomass of *C. necator* in our specific system, we performed a rigorous parameter calibration experiment using *C. necator* cells (without ISA) following the methodology established by Liu et al. (*Science*, **352**, 1210-1213 (2016)). This allowed us to derive system-specific coefficients that decouple the optical contributions of cellular biomass and intracellular PHB granules:

Biomass Coefficient Determination: During the initial 24 h of cultivation, the growth is characterized predominantly by biomass expansion while PHB accumulation remains negligible. Under these specific conditions, the measured OD_{600} values correlate exclusively with the concentration of bacterial biomass. This growth phase was therefore utilized to accurately determine the contribution of biomass to the optical density. By correlating OD_{600} with the dry cell weight (DCW) during this period, we derived a specific biomass coefficient of 401 mg biomass/L/OD. This value is comparable to the literature reported value of 448 mg biomass/L/OD.

PHB Scattering Coefficient Determination: Starting from the 48 h mark, PHB begins to accumulate significantly within the cells. During this phase, the measured OD_{600} represents the joint contribution of both bacterial biomass and intracellular PHB granules. By applying the previously determined biomass coefficient, we decoupled the optical density contributions and correlated the OD_{600} values with the respective accumulations of biomass and PHB. This approach allowed us to isolate the specific optical density contribution attributed to PHB. Using

late-stage samples (48–120 h) with varying PHB contents (quantified by HPLC), we isolated the optical density contribution of PHB (OD_{PHB}) and derived a specific coefficient of 785 mg PHB/L/OD_{PHB} (*vs* the literature value of 831 mg PHB/L/OD_{PHB}).

Based on these experimentally determined parameters, we updated the biomass calculation formula in the revised manuscript:

$$c_{biomass}\ (mg/L) = (OD_{total} - c_{PHB}/785) \times 401$$

where c_{PHB} is the concentration determined by HPLC. We believe this calibrated approach effectively eliminates errors caused by the mass of ISA materials and variations in optical measurements, providing a highly accurate estimation of the active biocatalyst.

We have updated the calculation model in Supplementary Method 8 using these experimentally validated coefficients. This ensures that our reported FE and ECE values accurately reflect the electron allocation in the *C.N*@ISA system. The data of FE and ECE based on the updated coefficients and corresponding revised figures have been added in the revised Manuscript (Section 2.6) and Supplementary information (Supplementary Method 8).

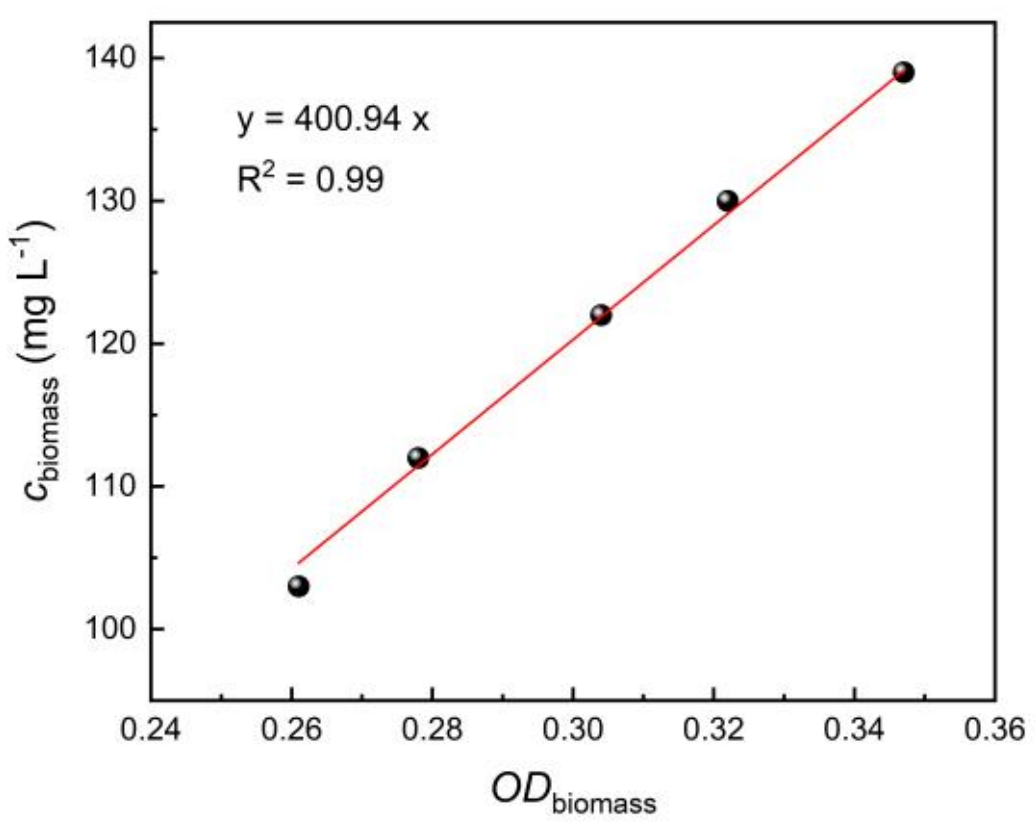

Fig. S43. The fitting plot of biomass coefficient.

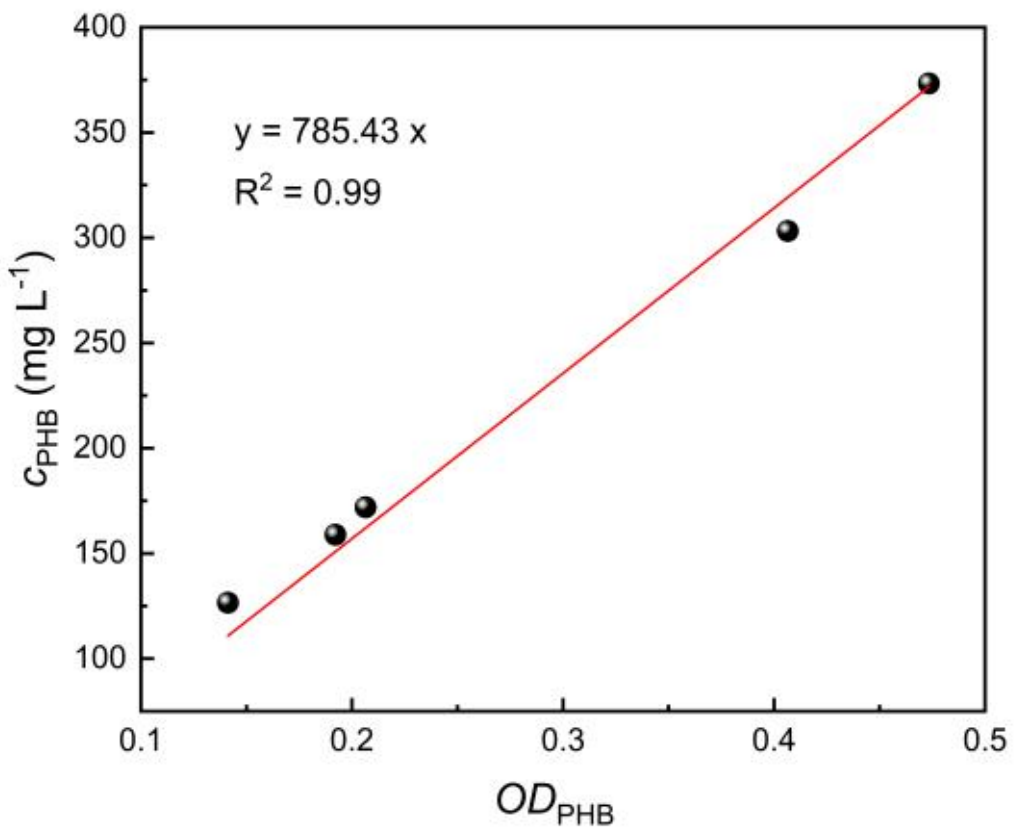

Fig. S44. The fitting plot of PHB scattering coefficient.

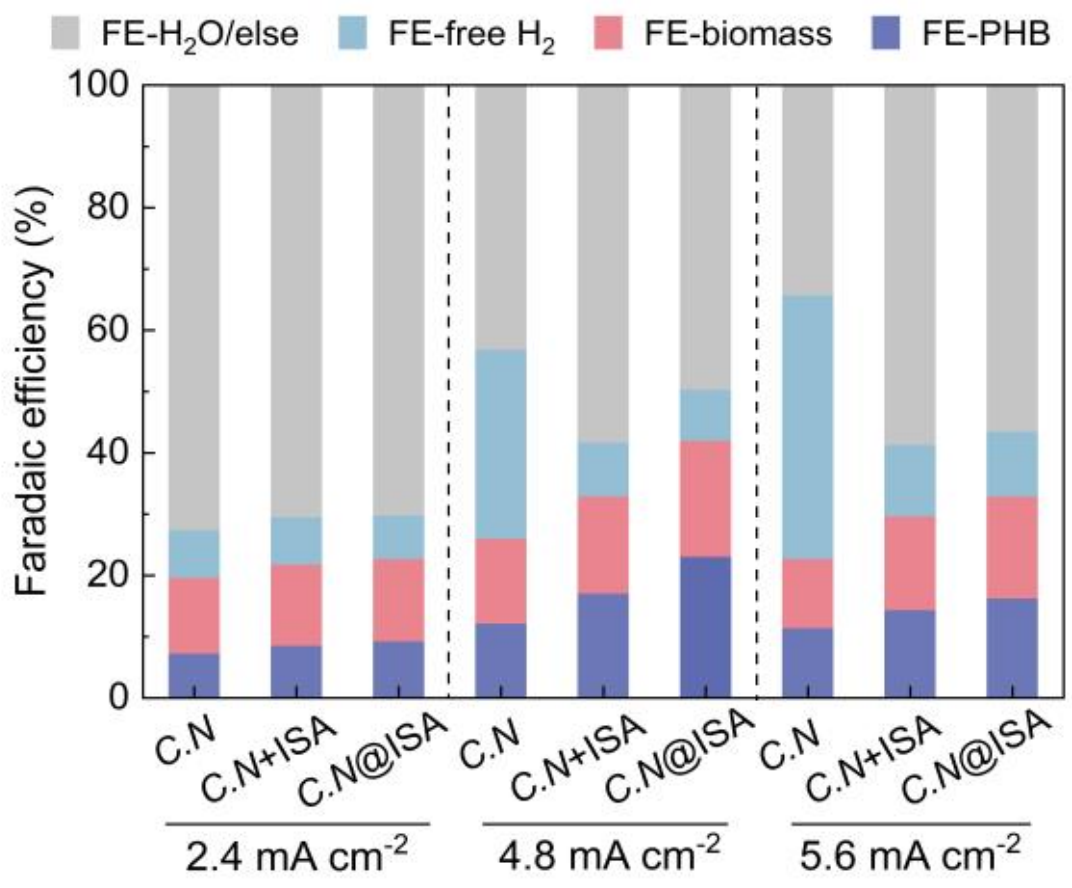

Fig. 6c. Distribution of cathodic electron utilization toward H_2 release, residual H_2, biomass formation, and PHB synthesis at different current densities.

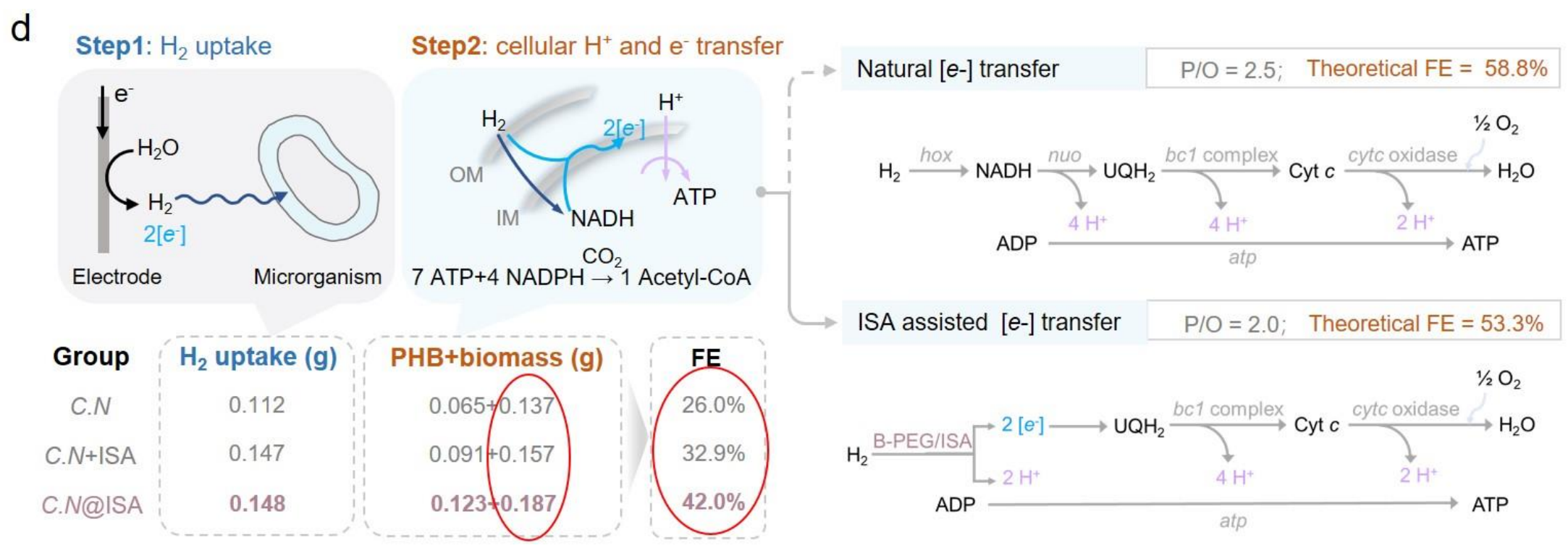

Fig. 6d. Analysis of electron allocation pathway (The sections highlighted in red indicate the modifications made within the manuscript).

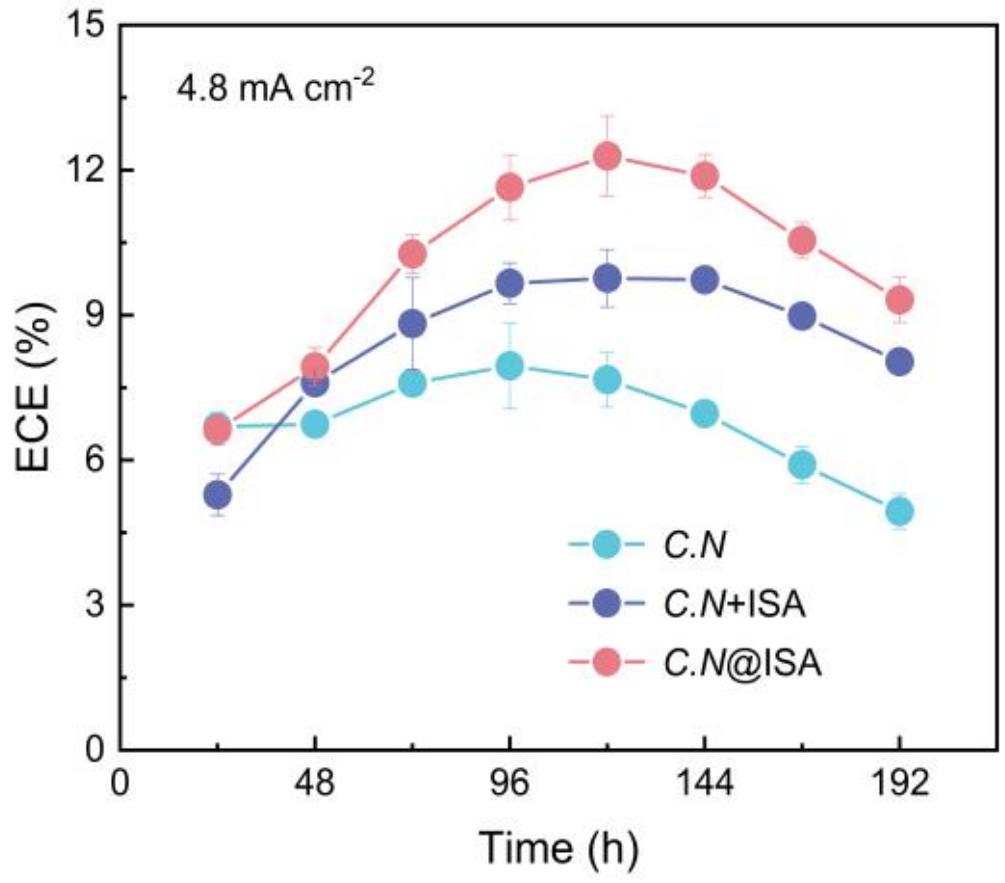

Fig. S39. ECE of the integrated inorganic-biological system.

2. *In the Figure S26, why the stainless steel was used as the WE? And why the free C.N@ISA was used for the test? It's better to compared with C.N@ISA immobilized on the electrode. Or the significant advantages of free C.N@ISA should be well presented. The stability of the system should be proved as long as the cultivation, i.e. 192h, not just 30 min in Fig 4h.*

Response:

We appreciate the reviewer's specific questions regarding the reactor configuration and stability evaluation.

We strategically selected a reactor configuration comprising a stainless steel (SS) working electrode and a planktonic *C. necator* culture. Our primary objective was to elucidate the mechanism of enhanced H_2 capture and utilization by the *C.N*@ISA hybrid, rather than to optimize upstream HER electrode materials. Therefore, we deliberately chose SS for its exceptional corrosion resistance and electrochemical stability, avoiding complex HER catalysts (e.g., Ni/Co-based alloys) that could interfere with the catalytic behavior of the ISAs, ensuring that the observed enhancements are strictly attributable to the biohybrid interface.

Furthermore, *C. necator* is naturally planktonic, distinguishing it from electroactive

bacteria such as *Geobacter* that depend on contact-mediated electron transfer. This characteristic allows the system to utilize the entire three-dimensional reactor volume instead of being confined to a two-dimensional electrode surface. Our interface engineering strategy involves anchoring ISAs directly onto the bacterial surface to overcome traditional mass transfer limitations. By establishing a localized H_2 microenvironment that remains associated with the cells during their movement, this approach ensures the efficient capture and utilization of reducing equivalents. Consequently, the system maximizes volumetric productivity, achieving a high cell density ($OD_{600} \approx 4.87$) and a record-high PHB yield (1058.8 mg L^{-1}). These explanations have been incorporated in the revised manuscript in section 2.6.

Regarding stability, we apologize for any confusion regarding the stability data and appreciate the opportunity to clarify this point. Fig. 4h (30 min) was conducted solely as a kinetic study to elucidate electron transfer mechanisms. The actual operational stability was demonstrated over the full 192 h cultivation period (Fig. 6a and Figs. S41/S42), where biomass and PHB production increased continuously. Besides this biological stability, we have additionally conducted new fed-batch experiments and performed a 360 h long-term chronopotentiometry test (new Fig. S40) during the revision, which demonstrates that the *C.N*@ISA system maintains stable performance for over 360 hours. The 360 h stability evaluation have been added in the revised manuscript (Section 2.6) and Supplementary information.

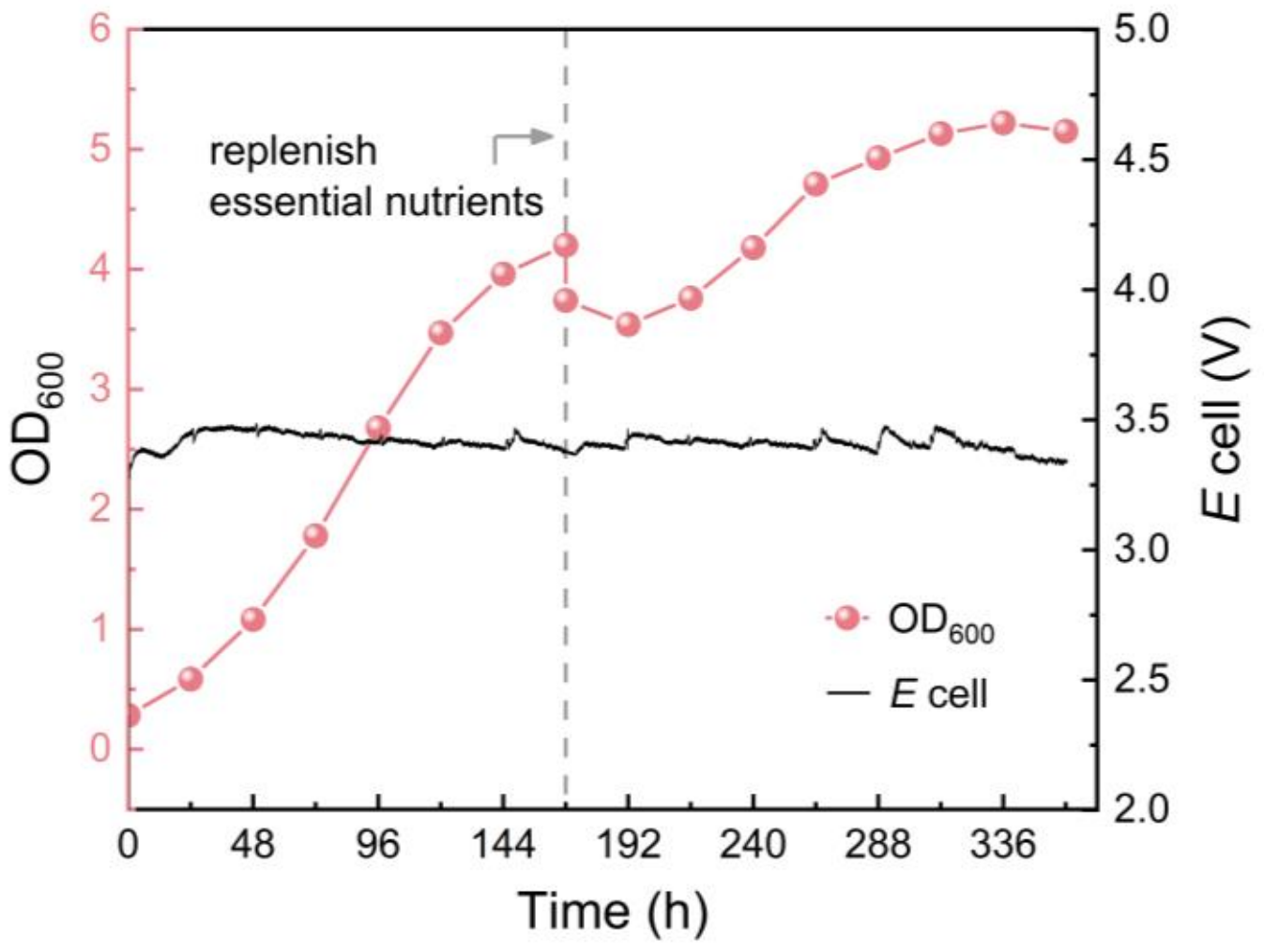

Fig. S40. Long-term stability evaluation of the *C.N*@ISA hybrid system.

3. *<u>The biological mechanisms were not confirmed. Although some postulations were made based on the literatures and measurements of heme, reduction power, gene expressions, but they were all not the direct evidence for the electrons' functions. With both hydrogen and electrodes, the contribution for direct H_2 and electrochemical should be analyzed, with more control or blank experiments.</u>*

Response:

We sincerely appreciate the reviewer's insightful and constructive comments regarding the biological mechanisms and the need for direct evidence of electron functions. We fully agree that a rigorous distinction between the contributions of bulk H_2 gas and electrochemical interfacial effects is paramount. To address this, we have systematically reinforced our mechanistic framework through new comparative experiments, isotopic tracing, genetic verification, and advanced metabolic analysis.

First, to distinguish the interfacial electrochemical effects from simple H_2 supply, we compared the growth kinetics of *C.N* and *C.N*@ISA in an Electrochemical System (in situ H_2

electro-generation) and a Direct H_2 Bubbling System (chemoautotrophic supply) under equivalent H_2 saturation levels. As shown in Fig. S24, the optical density (OD_{600}) of the *C.N*@ISA group was significantly higher than that of the *C.N* group in both electrochemical system and direct H_2 bubbling system. This disparity suggests that the ISA anchored on the cell membrane functions as an interfacial hydrogen utilization hub. It captures H_2 from the bulk solution, creating a H_2-enriched microenvironment, thereby overcoming mass-transport limitations and providing a localized, high-flux supply of active hydrogen species directly to the microbial respiratory chain. By comparing the electrochemical hydrogen supply system with the external supply system, the results indicate that under the same amount of hydrogen supply, the microbial growth in the electrochemical system is superior to that in the external supply system. This demonstrates that the electrochemical environment provides synergistic benefits beyond mere H_2 availability.

To provide direct chemical evidence of this internalized hydrogen utilization, we conducted isotopic tracing experiments using D_2. The GC-MS spectra of PHB extracted from *C.N* incubated with D_2 (Fig. S25) reveal a distinct mass shift towards higher m/z values compared to the H_2 control. The emergence of dominant peaks at m/z 119–120 and the altered fragmentation patterns confirm the substantial incorporation of deuterium into the biopolymer backbone. This solid evidence proves that hydrogen activated at the bio-inorganic interface is not merely a transient species but is effectively internalized and utilized for NAD(P)H regeneration and subsequent biosynthesis. Furthermore, we verified this via a hydrogenase-deficient mutant strain (*C.N*ΔMBHΔSH). While its growth was severely impaired under chemoautotrophic conditions, the covalent anchoring of ISA largely restored its growth and metabolic activity (Fig. 5f). This genetic restoration demonstrates that the ISA can functionally mimic membrane-bound hydrogenases, directly facilitating the entry of hydrogen-derived

reducing equivalents into the cell.

Furthermore, we identified an electron transfer pathway assisted by self-secreted mediators. Utilizing HPLC and CV (revised Fig. 4f and Fig. S27), we detected distinct chromatographic and redox couple peaks matching authentic flavin adenine dinucleotide (FAD) standards, confirming active flavin secretion in bacterial suspension of *C.N*@ISA group. This dual-track mechanism, combining ISA-facilitated HOR and FAD-assisted electron transfer, is consistent with recent literature (*Nat. Chem.*, **15**, 1400-1407 (2023); *Nat. Commun.* **16**, 8141 (2025)), highlighting the synergistic mechanisms stimulated by the microenvironment.

Finally, to address the systemic efficiency of the electrochemical system, we integrated the concept of "Metabolic Rewiring" as proposed in recent pioneering work (*Proc. Natl Acad. Sci. USA* **120**, e2308373120 (2023)). Our data indicates that the electrochemical interface does not merely provide substrates; it fundamentally alters the microbial energy landscape. We observed significantly elevated ATP levels and a more robust proton motive force (PMF) in *C.N*@ISA in the electrochemical system compared to direct H_2 bubbling system. This synergy between the ISA-engineered interface and the FAD-assisted driving force enhances the efficiency of converting reducing equivalents into biological energy (ATP), thereby driving the record-high PHB production.

In summary, by combining comparative experiments, isotopic tracing, genetic verification and mediator detection, we have provided converging lines of direct evidence for the biological mechanisms through which ISA-biohybrids utilize hydrogen-derived energy in the integrated electrochemical system. Related discussions have been added to the revised manuscript (Section 2.3, 2.4 and 2.5).

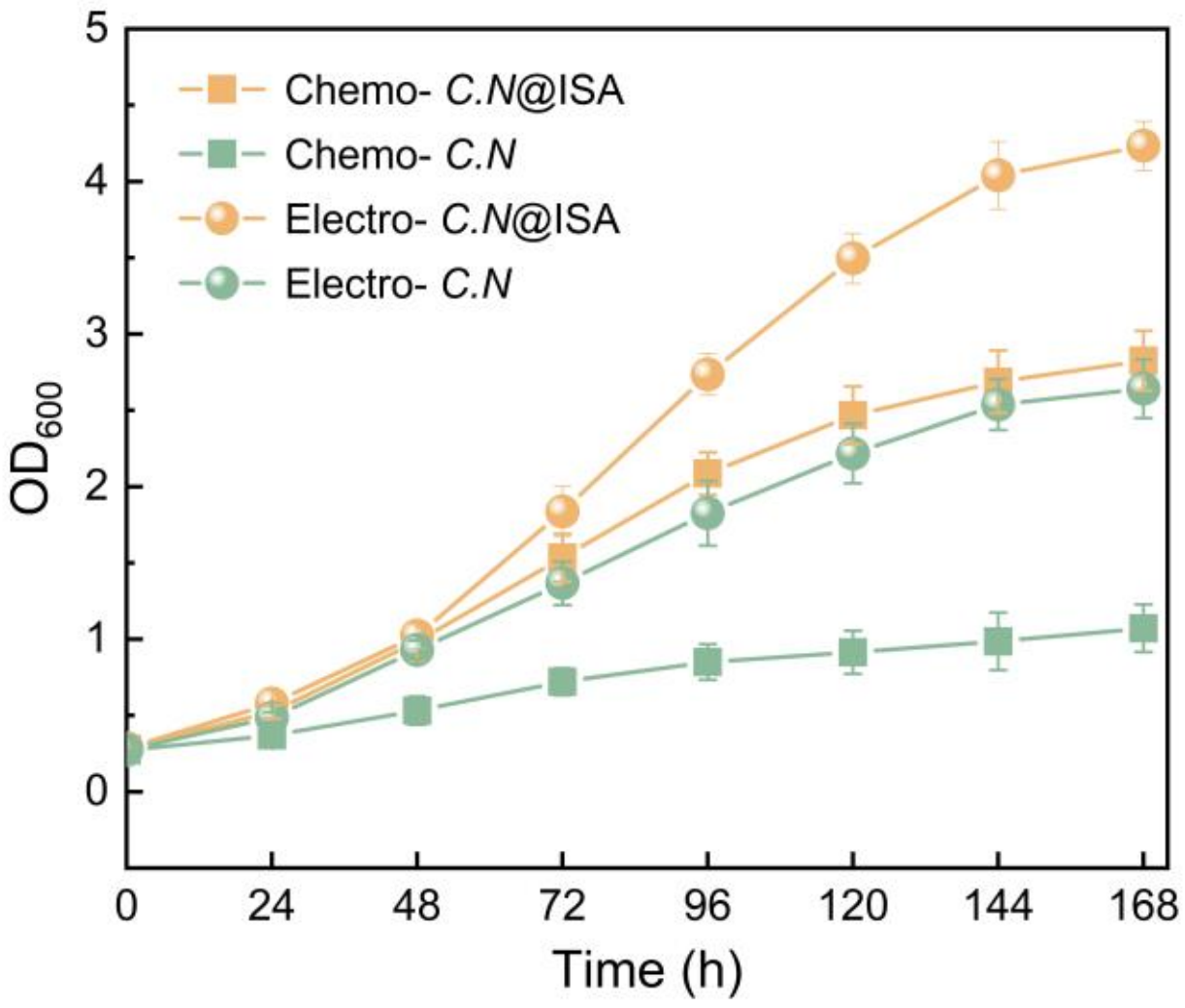

Fig. S24. Time-dependent OD_{600} values in *C. necator* cultures under electroautotrophic and chemoautotrophic conditions.

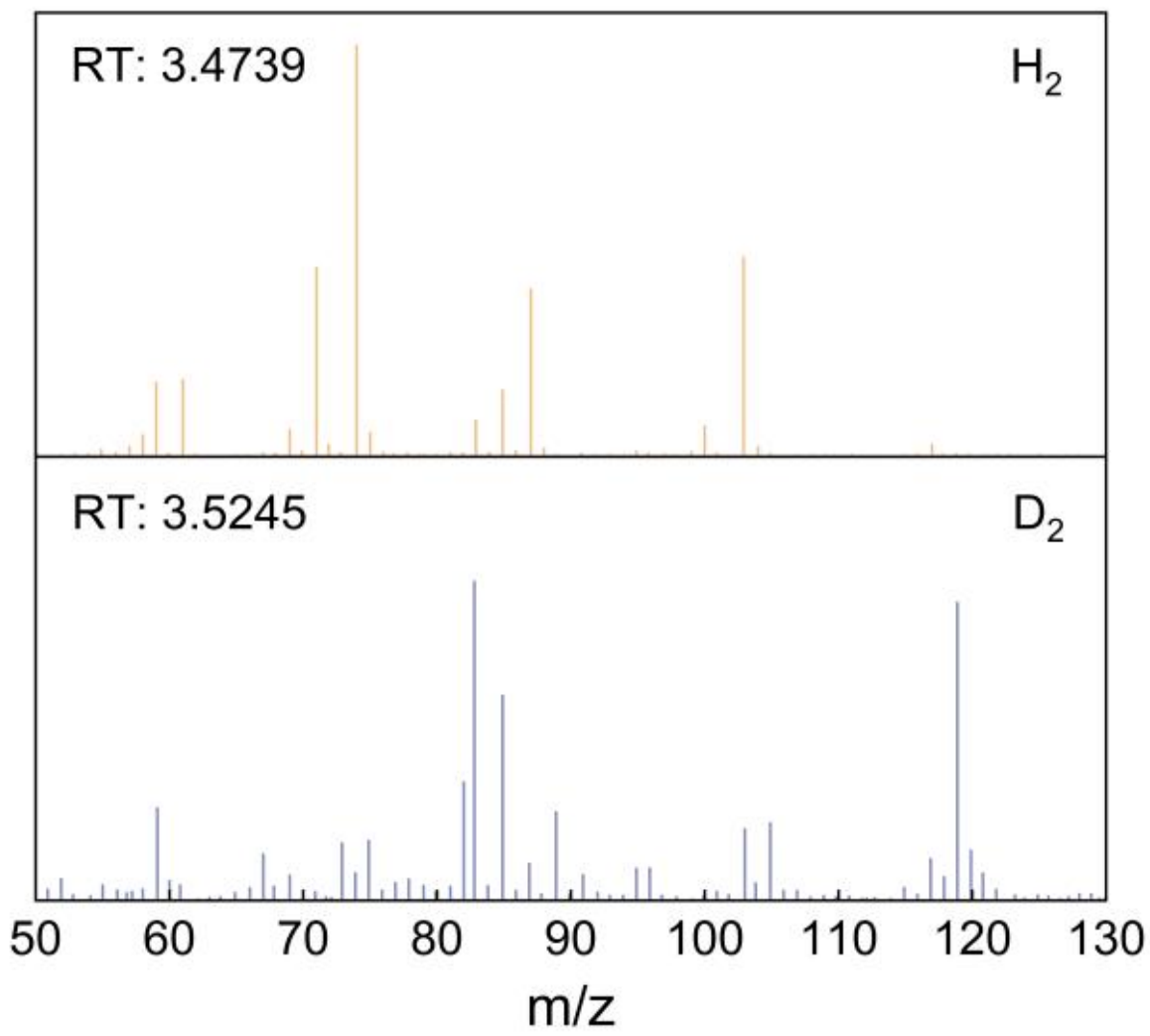

Fig. S25. Comparison of GC-MS spectra of PHB extracted from *C.N*@ISA cultivated under H_2 (top) and D_2 (bottom) atmospheres.

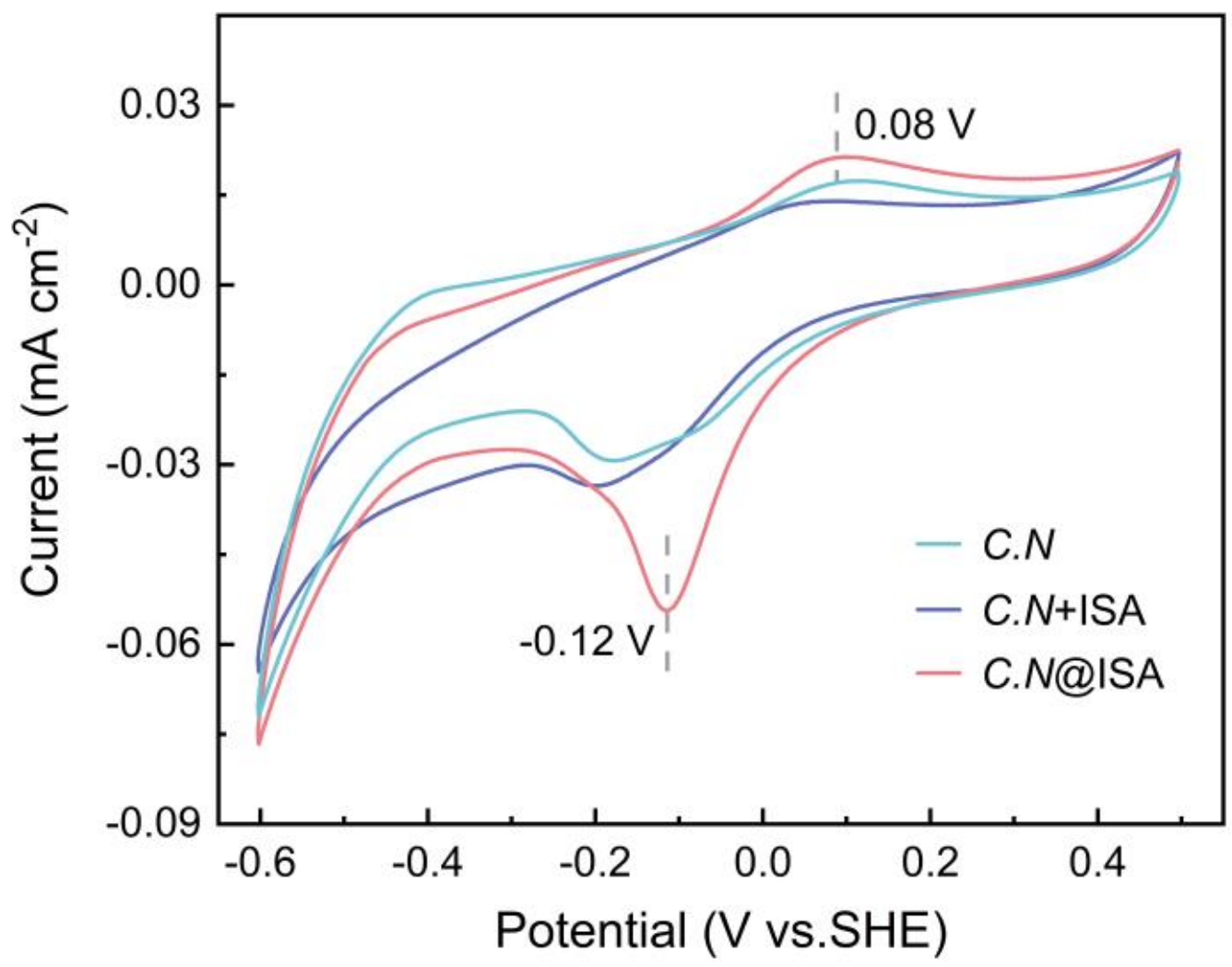

Fig. 4f. CV curves of the operating integrated system. *C.N*@ISA, *C.N*+ISA, *C.N* are all suspended in the electrolyte.

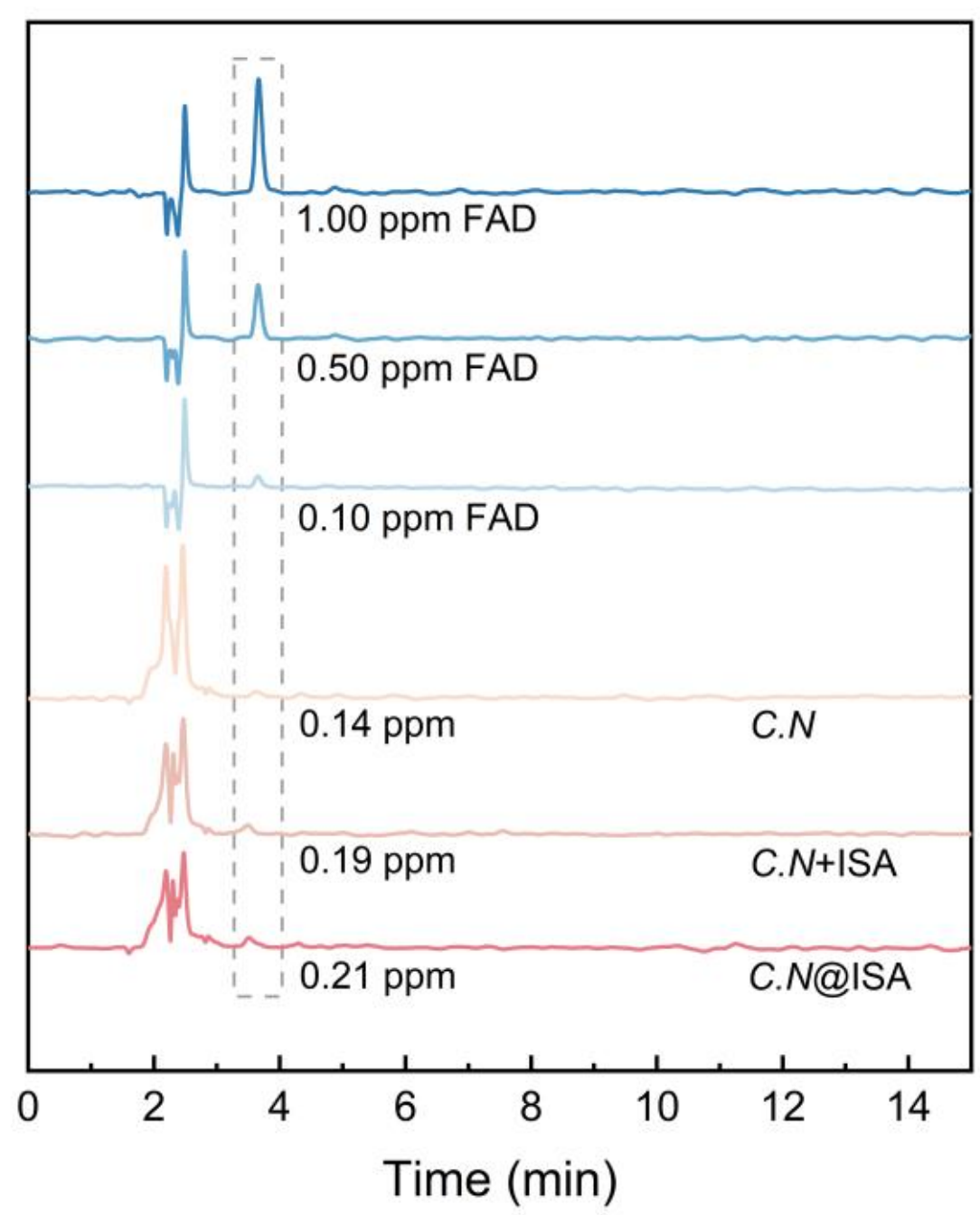

Fig. S27. Liquid chromatographic peak signal and calibration curve of FAD.

4. *Some literatures were not cited rightly, or read carefully. For example, in the last two line in the first page of introduction section, the ref 19 is talking about a direct electron supply by the organic semiconductors, no relation to single-atom metal catalysts or H_2 dissociation energy barrier.*

Response:

We sincerely thank the reviewer for the meticulous reading of our manuscript and for pointing out these citation inaccuracies. We apologize for the oversight in the original submission.

In accordance with your suggestion, we have replaced **Ref 19** with a more relevant study that directly addresses the role of single-atom catalysts in lowering the H_2 dissociation energy barrier: *ACS Catal.* **14**, 2194-2201 (2024).

Furthermore, we have performed a comprehensive internal audit of all references throughout the manuscript, and the following are other citations that have been revised:

1. **Ref 4:** Replaced with the foundational work on microbial electrosynthesis: Nevin, K. P., et al. *mBio* **1**, e00103-10 (2010), which more accurately supports the conceptual framework of feeding microbes electricity for CO_2 conversion.
2. **Ref 13:** Replaced with a comprehensive review on hydrogenase overproduction: Fan, Q., et al. *Int. J. Mol. Sci.* **21,** 5890 (2020), providing a more relevant biotechnological overview.
3. **Ref 18:** Replaced with Wang, L. F. & Yang, R. T. J. *Phys. Chem. C* **113**, 21883-21888 (2009), which specifically discusses the hydrogen storage properties of N-doped carbon materials.
4. **Ref 36**: Replaced with **Ref 28**, ensuring internal consistency.
5. **Ref 38:** Replaced with Tremblay, P. L. et al. *Nat. Commun.* **16**, 8141 (2025); **Ref 39:** Replaced with Fu, B. et al. *Nat. Chem.* **15**, 1400-1407 (2023). Both studies reported that *C. necator*

utilizes secreted flavins as diffusible redox mediators to facilitate interfacial electron uptake from inorganic materials.

We have meticulously cross-checked the updated reference list against the revised text to prevent any further discrepancies.

In brief, the manuscript presented plenty of data and tests, while the key contributions should be reorganized and the logical chain from the single-atom iron to hybrid system should be proposed.

Response:

We sincerely thank the reviewer for recognizing the extensive data and experimental validation presented in our manuscript. We agree that reorganizing the key contributions and the logical chain from the material design (ISA) to the biological enhancement (hybrid system) is essential for the clarity and impact of this work. Following the reviewer's suggestion, we have reorganized the manuscript to center around a clear "Structure-Interface-Mechanism-Performance" logical chain.

The proposed logical chain is as follows:

1. Atomic Site Design (The Source): We utilized ISA as the catalytic center. The ISA structure provides maximal atom economy and uniform $Fe-N_4$ sites with high intrinsic activity for capturing and activating hydrogen species.
2. Interface Engineering (The Bridge): Instead of relying on passive diffusion in bulk solution, we employed click chemistry to covalently anchor ISA directly onto the *C. necator* surface. This creates an abiotic-biotic interface that overcomes the mass transfer limitations inherent in suspension cultures.
3. Localized Microenvironment (The Mechanism): This precise proximity creates a high-

concentration localized H_2/proton microenvironment directly at the cell membrane. As confirmed by our supplementary D_2 labeling, HPLC and CV analyses, this ensures that the reducing equivalents are captured and utilized by the microbial electron transport chain with minimal loss to the bulk environment.

4. Systemic Output (The Performance): The end of this logical chain is the recorded high PHB volumetric productivity and sustained stability.

Reviewer #4 (Remarks to the Author):

The authors report an iron single-atom biohybrid constructed via click chemistry and claim high CO_2-to-PHB production and Faradaic efficiency. However, the manuscript faces some major design flaws, method inconsistencies, and some mechanism interpretations are not supported by the data. In several cases the experiments are inconsistent. Overall, the work falls short of the quality needed for NC, and I provide some detailed comments below.

Response:

We sincerely thank the reviewer for the critical and comprehensive assessment of our work. We take the comments very seriously. In this revision, we have performed a fundamental restructuring of the manuscript to address the concerns regarding reactor design, methodological consistency, and mechanistic clarity. We believe the revised manuscript now presents a robust and rigorously validated study.

1. *A major problem the authors mentioned about ROS generated at the anode severely hindering microbial electrosynthesis is largely created by the reactor design in this study.*

They use a single-chamber electrochemical reactor, in which O_2 evolved at the anode can freely diffuse to the cathode. In conventional microbial electrosynthesis, a membrane-separated two-chamber configuration is used specifically to prevent such O_2 crossover, so ROS are not a dominant limitation. As shown in Fig. 6c, most electrons in their system appear to be consumed by ORR, which is thermodynamically favored over proton reduction. In other words, the reactor is essentially running OER and ORR in a loop, wasting electrons and energy. This design creates an artificial ROS problem and undermines the efficiency claims. The authors probably can redo the tests in a standard two-chamber reactor.

Response:

We sincerely thank the reviewer for this critical and insightful comment regarding the reactor configuration. We acknowledge that two-chamber reactors with ion-exchange membranes (*e.g.*, Nafion) are the standard convention for *strictly anaerobic* microbial electrosynthesis (MES) to prevent oxygen toxicity. However, the choice of a single-chamber, membrane-free configuration in our study was a deliberate design decision and driven by the specific metabolic requirements of *Cupriavidus necator* and the pursuit of a scalable engineering architecture. Our reasoning is detailed as follows:

1. Biological Requirement of O_2 for *C. necator*: Unlike strict anaerobes (e.g., methanogens or *Sporomusa ovata*) typically used in MES, *C. necator* is a facultative chemolithoautotroph (a hydrogen-oxidizing "Knallgas" bacterium). To efficiently fix CO_2 via the Calvin-Benson-Bassham (CBB) cycle, it necessitates O_2 as the terminal electron acceptor for oxidative phosphorylation to generate sufficient ATP [*Proc. Natl Acad. Sci. USA* 112, 2337-2342 (2015); *Science* **352**, 1210-1213 (2016)]. In a strict anaerobic cathode chamber of a two-chamber system, *C. necator* suffers from severe ATP limitation, leading to poor growth and product synthesis. Therefore, maintaining a microaerobic environment is metabolically essential for this

strain. Our single-chamber design strategically utilizes the O_2 evolved at the anode to support bacterial respiration at the cathode, creating a self-sustaining, aerated bio-electrochemical system without the need for external O_2 sparging.

2. Engineering Advantages and Scalability: From the perspectives of energy efficiency and industrial scale-up, membrane-free single-chamber reactors offer superior performance. Ion-exchange membranes introduce significant internal ohmic resistance, which drastically increases the cell voltage required to drive the reaction and lowers the overall energy efficiency [*J. Photoch. Photobio. A* **358**, 411-415 (2018)*;* *Science* **352**, 1210-1213 (2016)]. Previous studies on *C. necator*-based hybrids have explicitly favored single-chamber designs to minimize this voltage penalty and simplify reactor maintenance [*Curr. Opin. Chem. Biol.* 41, 107–113 (2017)]. Eliminating the membrane not only enhances energy efficiency but also significantly reduces capital and operational costs.

3. The Role of ISA in Resolving the "Oxygen Paradox": The single-chamber design inherently presents a trade-off: While it provides necessary O_2 and lowers resistance, it also risks generating toxic reactive oxygen species (ROS) via abiotic oxygen reduction (ORR) at the cathode. This "oxygen paradox" is precisely the challenge our work aims to solve. Rather than reverting to a high-resistance two-chamber system, we engineered the ISA (Iron Single Atom) interface to act as a specific scavenger. The ISA interface allows us to retain the engineering benefits of the single-chamber architecture while detoxifying the inevitable ROS byproducts. Regarding the electron consumption shown in Fig. 6c, it is important to note that while a portion of the electron flux is diverted to ORR, a significant fraction of this O_2 consumption is biological respiration, which is highly productive as it yields the ATP necessary for CO_2 fixation. Our ISA strategy ensures that abiotic, ROS-generating ORR is suppressed or that generated ROS are rapidly scavenged, thereby maximizing the effective electron flux toward biological

utilization.

In summary, the single-chamber design in our study is not a structural flaw but a target architecture optimized for efficient bio-electrosynthesis using *C. necator*. Our work demonstrates a material-based strategy to bridge the gap between electrochemical efficiency and biological compatibility. We have revised the Introduction and Section 2.6 to incorporate all above discussions.

2. *The reference electrode and potential scale should be reported consistently. The text around the CV data indicates that potentials are referenced to saturated Ag/AgCl, but this is easy to miss and is not clearly reflected in all figure captions. Given that the mechanistic arguments rely on potential windows (e.g., for HOR/HER), the reference scale should be explicitly stated where electrochemical data are plotted.*

Response:

We thank the reviewer for pointing out this important detail regarding data presentation standards. We agree that explicitly stating the reference electrode and potential scale is crucial for accurately interpreting the electrochemical mechanisms, particularly for identifying the thermodynamic windows of HOR and HER. For better comparability with previous studies, all electrode potentials have been standardized to the standard hydrogen electrode (SHE) scale and explicitly annotated across all methods (Methods 4.7 and 4.8) and figures (Fig. 4) in the revised version.

3. *The electrochemical characterization used to support the mechanism doesn't seem to be representative of the actual microbial electrosynthesis system. The reactor configuration for CO_2-to-PHB conversion (method 4.4) uses a stainless-steel mesh cathode and*

planktonic C.N or C.N@ISA in a single-chamber cell. In contrast, the CV/EIS experiments (method 4.8) employ carbon cloth electrodes pre-loaded with B-PEG/ISA and then colonized by cells to form a C.N@ISA-loaded bioelectrode. These are fundamentally different electrode architectures, with different surface areas, transport properties, and cell/material interactions. If microbial electrosynthesis performance is dominated by planktonic C.N/ISA hybrids in the bulk, then the behavior of a pre-formed C.N@ISA biofilm on carbon cloth is not relevant. If the authors instead claim that biofilms on the stainless-steel mesh dominate, then method 4.4 does not recreate the same structure as method 4.8. In either case, the electrochemical results in Fig. 4 are decoupled from the actual reactor configuration and cannot be used to justify the proposed promotion mechanism.

Response:

We are grateful to the reviewer for identifying this critical discrepancy. We agree that the electrode architecture used in our initial electrochemical characterization (biofilm on carbon cloth) did not accurately represent the actual conditions in the microbial electrosynthesis reactor (planktonic cells with stainless-steel mesh). We have thoroughly addressed this by revising the experimental setup and the corresponding manuscript sections.

We confirm that our CO_2-to-PHB conversion system is dominated by planktonic *C.N*@ISA hybrids suspended in the bulk solution, rather than a biofilm attached to the electrode. In our actual reactor configuration (Method 4.4), the stainless-steel mesh primarily functions to generate reducing equivalents (H_2/electrons), and we observed negligible biomass attachment to the mesh surface after operation. Our original intention in using the carbon cloth electrode colonized by cells (Method 4.8) was to maximize the electrochemical signal-to-noise ratio to sensitively detect any potential surface redox-active proteins or secreted electron mediators associated with the bacteria. However, we fully accept the reviewer's critique that this

“electrode-biofilm” model artificially enhances direct electron transfer pathways that are likely negligible in our actual “electrode-suspension” production system. Relying on this data could indeed lead to a misinterpretation of the mechanism. To provide a representative mechanistic analysis, we have repeated the electrochemical characterization using conditions identical to the actual reactor. We replaced the carbon cloth with the stainless-steel mesh used in the production reactor. We tested planktonic *C.N*@ISA suspensions rather than pre-formed biofilms. In the revised CV curves (Fig. 4f), we observed a pair of redox signals with a reduction peak at −0.12 V and an oxidation peak at 0.08 V (vs. SHE), which are tentatively attributed to flavin species (Fig. 4f). The presence of flavins was further confirmed by HPLC analysis, where a chromatographic peak with a retention time matching that of the flavin adenine dinucleotide (FAD) standard was detected (Fig. S27). These results are in good agreement with recent literature reports [*Nat. Chem.* **15**, 1400-1407 (2023); *Nat. Commun.* **16**, 8141 (2025)]. In addition, the FAD concentration in the *C.N*@ISA group was higher than that in the *C.N*+ISA and *C.N* groups, supporting a synergistic effect whereby membrane-anchored ISA-facilitated H_2 oxidation cooperates with FAD-mediated electron transfer to accelerate electron flux into microbial metabolism.

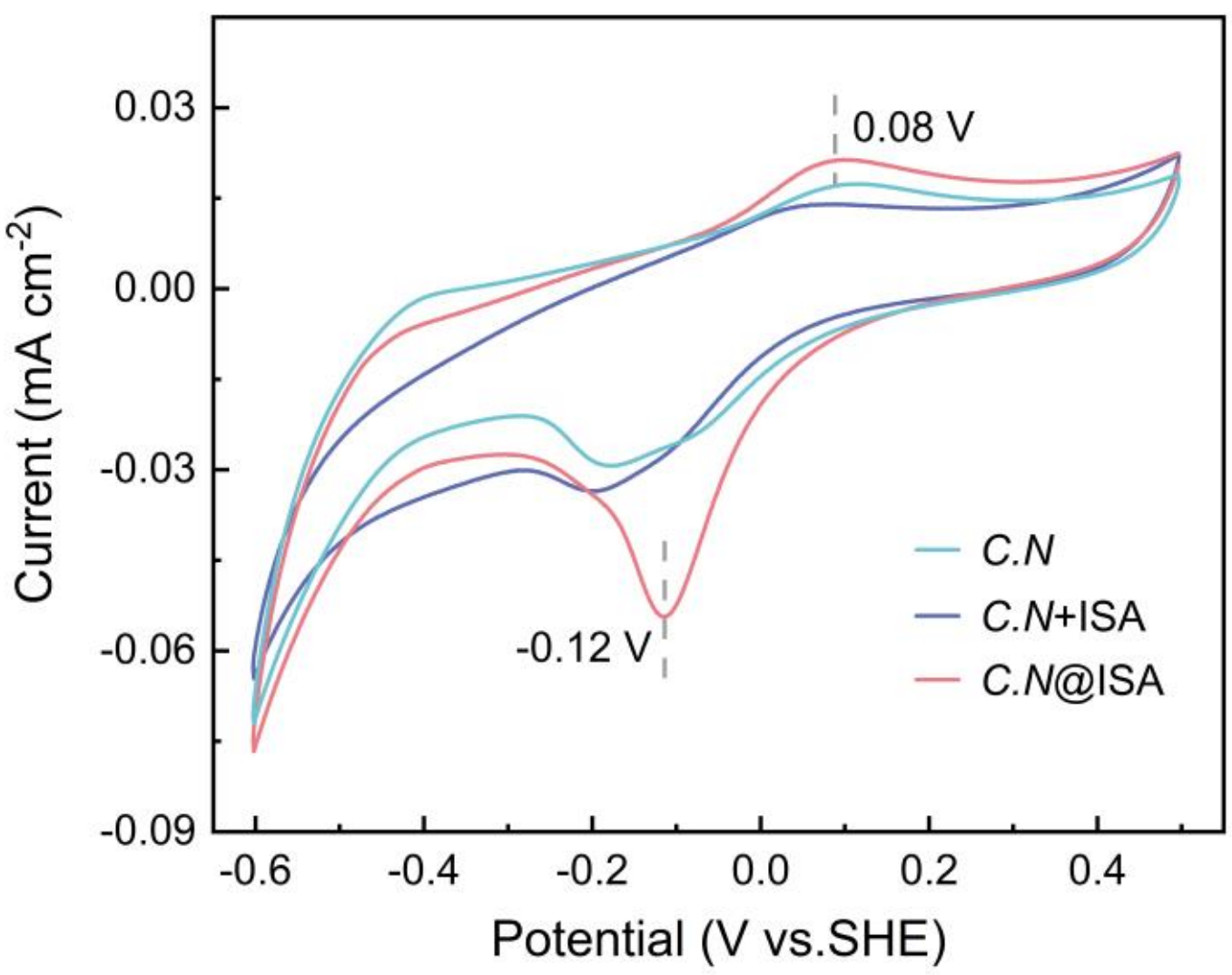

Fig. 4f. CV curves of the operating integrated system. *C.N*@ISA, *C.N*+ISA, *C.N* are all suspended in the electrolyte.

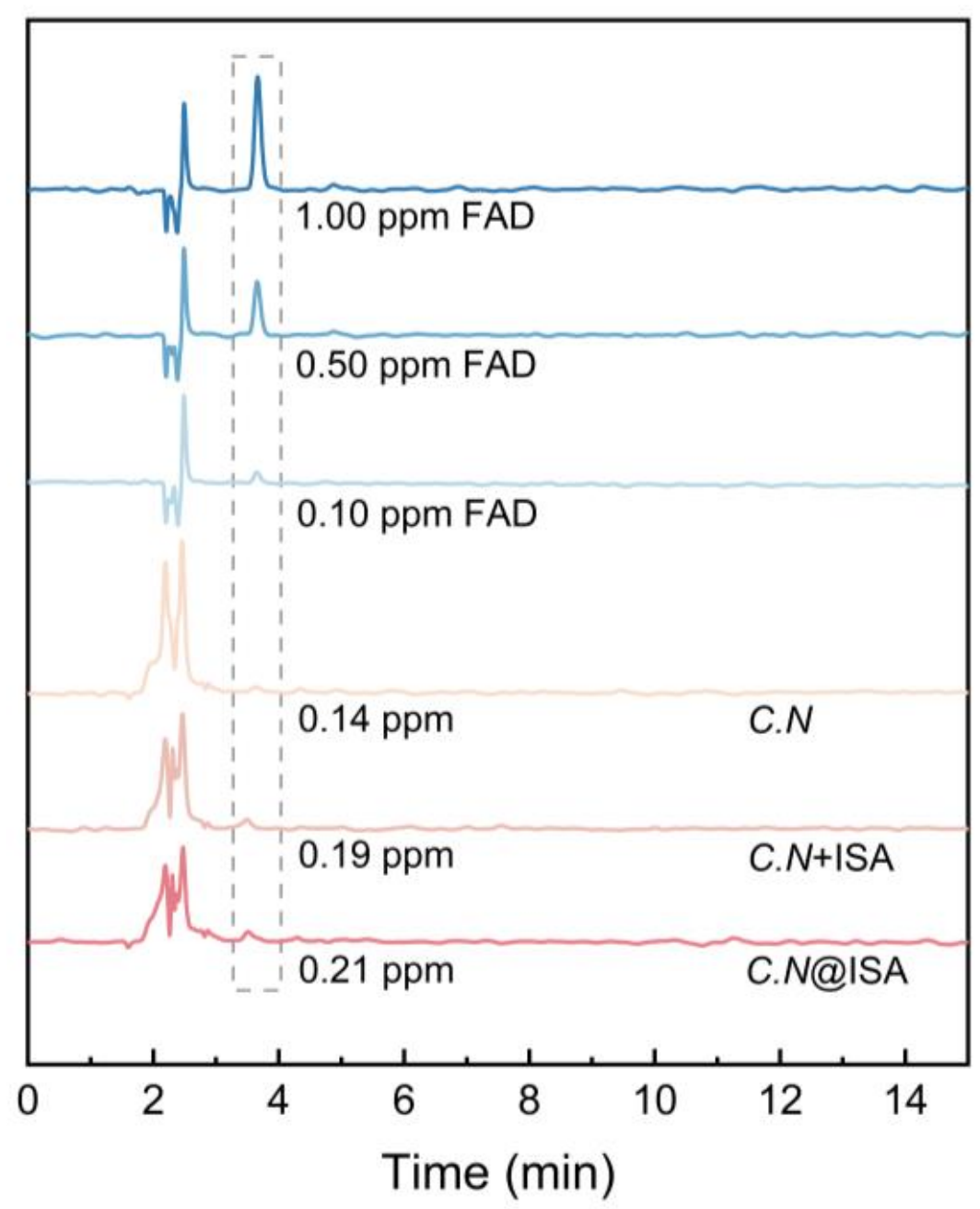

Fig. S27. Liquid chromatographic peak signal and calibration curve of FAD.

Regarding the electrochemical impedance spectroscopy (EIS) analysis, we agree that the impedance behavior of a dense biofilm on carbon cloth cannot be extrapolated to a planktonic

system. Therefore, we performed additional EIS measurements in the planktonic cell suspension system with stainless-steel mesh electrode. As shown in new Fig. 4g, the presence of a large circular arc (R_{ct} > 2000 Ω) may be attributed to the fact that direct electron transfer pathways in the actual "electrode–planktonic bacteria" system are negligible. These results are consistent with a H_2-mediated electron transfer mechanism dominating in our system.

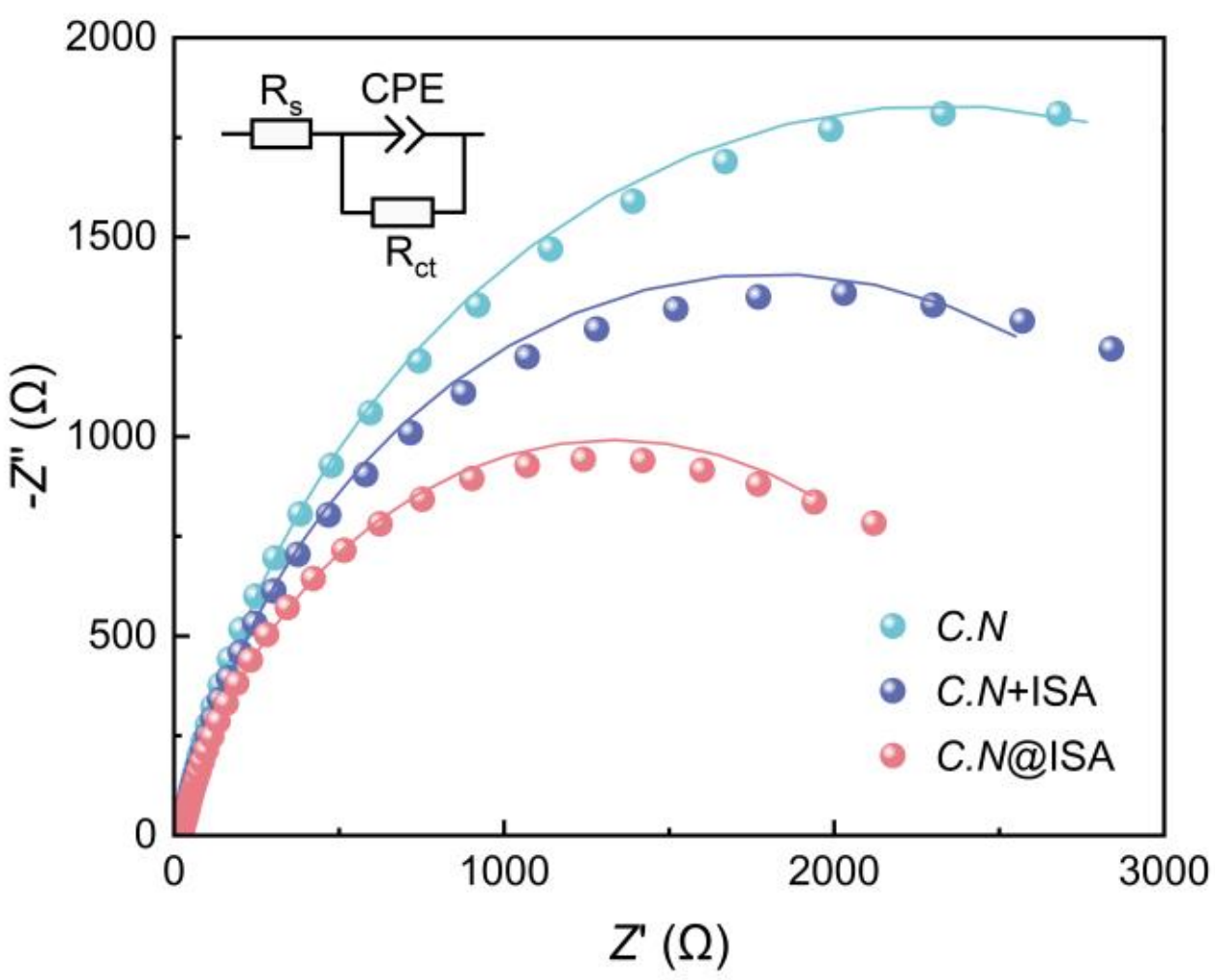

Fig. 4g. Nyquist plots and equivalent circuit fitting plots of the operating integrated system. *C.N*@ISA, *C.N*+ISA, *C.N* are all suspended in the electrolyte.

We believe these revisions align the mechanistic characterization strictly with the reactor performance data, ensuring a consistent and accurate narrative. We have revised the relevant discussion in Section 2.4 accordingly.

4. *The stability claims for ISA are not convincing in the context of the actual reactor conditions. The authors show that ISA morphology and Fe content remain unchanged after 120 h of electrolysis, but this test appears to be under HER-like conditions without significant O_2. It is well known that Fe–N–C catalysts degrade much more rapidly under ORR, and in the*

authors' single-chamber system O_2 is inevitably produced at the anode and can diffuse to the cathode. Furthermore, if the core functionality is attributed to planktonic C.N–ISA hybrids, then electrode-level HER/ORR stability is not directly relevant to the biological performance. The stability discussion therefore does not substantiate the claims being made.

Response:

We thank the reviewer for this critical assessment regarding the stability of our system. We apologize for any potential misunderstanding caused by the insufficient description of the ISA stability tests in the original manuscript. The stability of the ISA was rigorously evaluated within a single chamber electrochemical reactor. This configuration accurately simulates the integrated operational environment where oxygen is continuously evolved at the anode and subsequently diffuses toward the cathode. Under these oxygen-containing electrochemical conditions, the ISA maintained excellent stability.

Building upon the catalyst stability, we demonstrated the robustness of the *C.N*@ISA biohybrid in the original manuscript. During the 192-hour cultivation, the biohybrid exhibited high cellular activity with continuous increases in both biomass accumulation and PHB production (Fig. 6a and Figs. S41/S42). Furthermore, we have provided new data from extended fed-batch cultivation lasting 360 hours in the revised manuscript (Fig. S40). These results collectively demonstrate that the *C.N*@ISA system remains operationally stable for over 360 hours of continuous cultivation. These findings confirm that both the ISA catalyst and the engineered inorganic biological interface exhibit exceptional stability under realistic operating conditions. The corresponding discussion has been integrated into Section 2.6 of the revised manuscript.

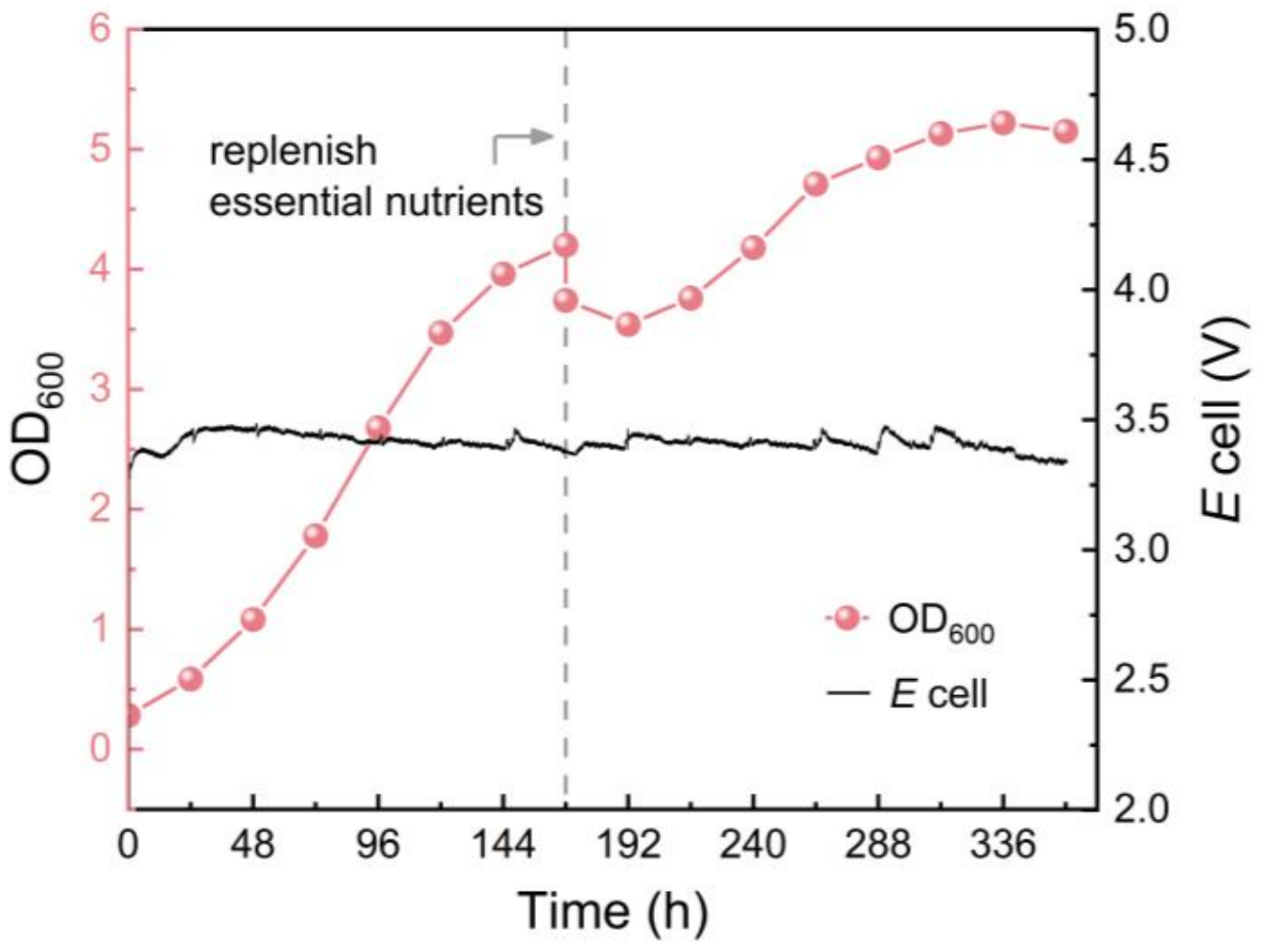

Fig. S40. Long-term stability evaluation of the *C.N*@ISA hybrid system.

5. *The mechanistic narrative around H_2 dissociation, H radical formation, and H^+ supply is not supported by the data. Different electrode preparation methods are used for different panels in Fig. 4, making mechanistic interpretation very difficult. Specifically,*

• The RRDE data in Fig. 4b do not show the typical mass-transport-limited plateau expected for HOR; the continued rise in current at higher potentials is more consistent with oxidation of partially reduced Fe sites and other side processes, potentially overestimating "HOR activity" differences with and without ISA.

Response:

We sincerely appreciate the reviewer's valuable feedback regarding the HOR polarization curves. The atypical current response observed in the initial submission was likely attributed to suboptimal experimental protocols during electrode preparation and inconsistent H_2 saturation within the electrolyte. To resolve this, we have repeated the RRDE experiments under rigorously optimized and controlled conditions. The revised Fig. 4b now displays a well defined limiting current plateau where the current density exhibits a clear dependence on the rotation

speed. We also observe that the polarization curves exhibit a broad mixed kinetic-diffusion controlled region. This behavior is consistent with the porous architecture of the catalyst layer and the corresponding distribution of active sites rather than the redox transitions of partially reduced metal centers. The Koutecký-Levich plots provided in Fig. S22 show excellent linearity, further confirming that the measured currents originate from genuine H_2 oxidation. Additionally, the Tafel slope of approximately 28.6 mV dec^{-1} extracted from the kinetic controlled region in Fig. S23 is consistent with a dissociative hydrogen activation mechanism. We have refined the mechanistic discussion and updated Section 4.7 and Fig. 4b in the revised manuscript.

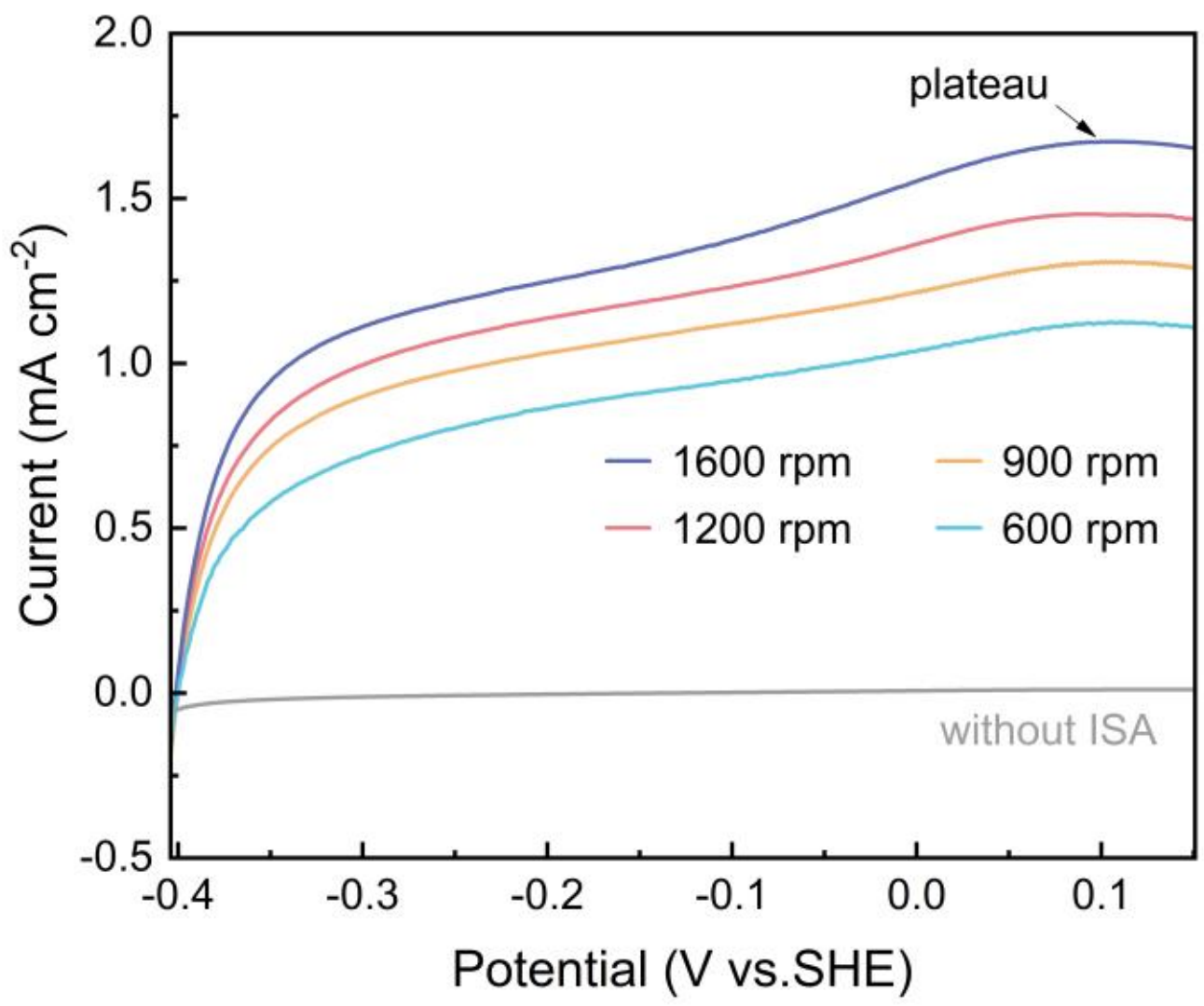

Fig. 4b. Polarization curves of ISA recorded at rotation speeds from 600-1600 rpm. The solid gray line shows the polarization curve of glassy carbon electrode without ISA at 1600 rpm in a H_2-saturated electrolyte.

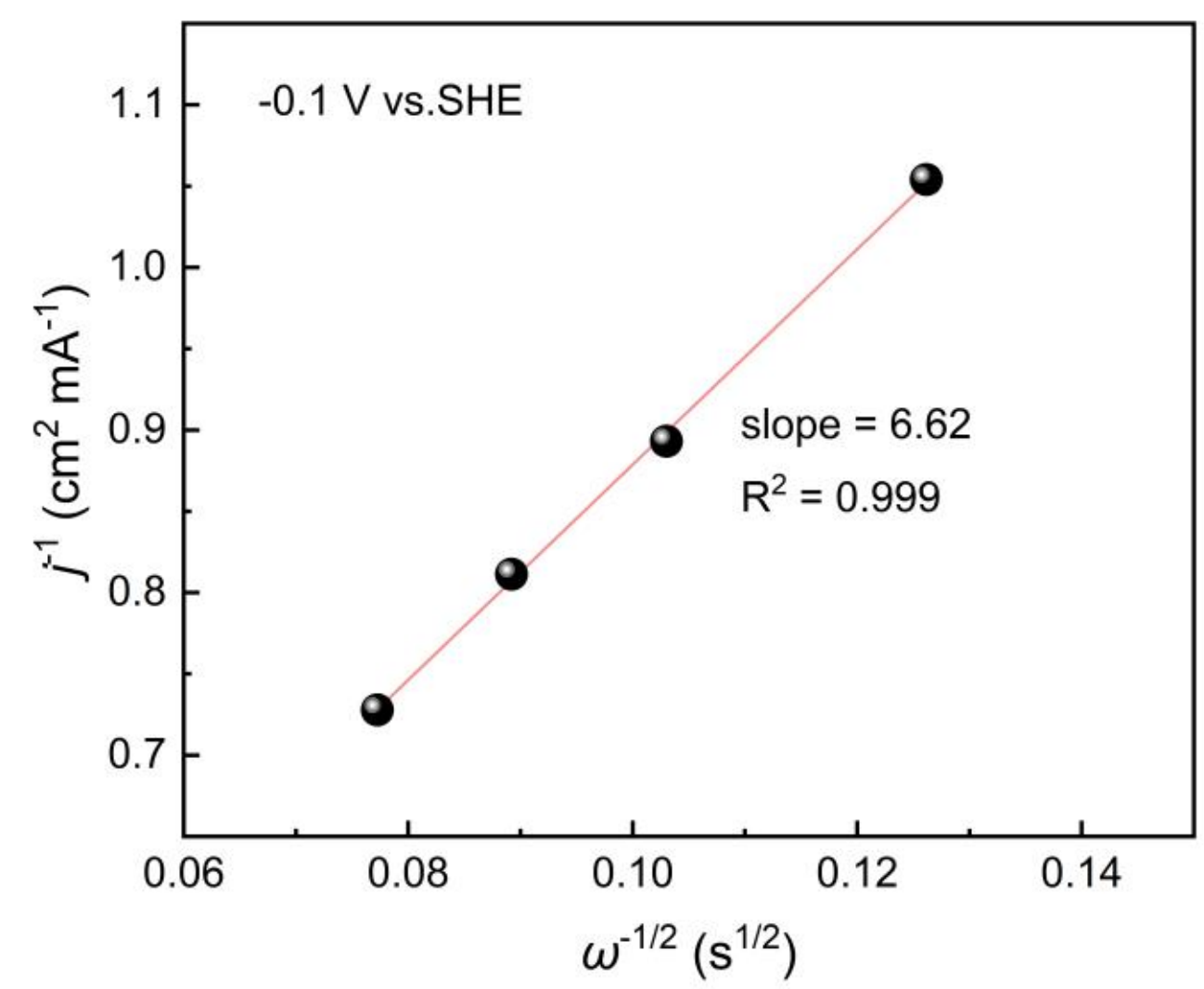

Fig. S22. The corresponding Koutecký-Levich plot at -0.1 V vs.SHE.

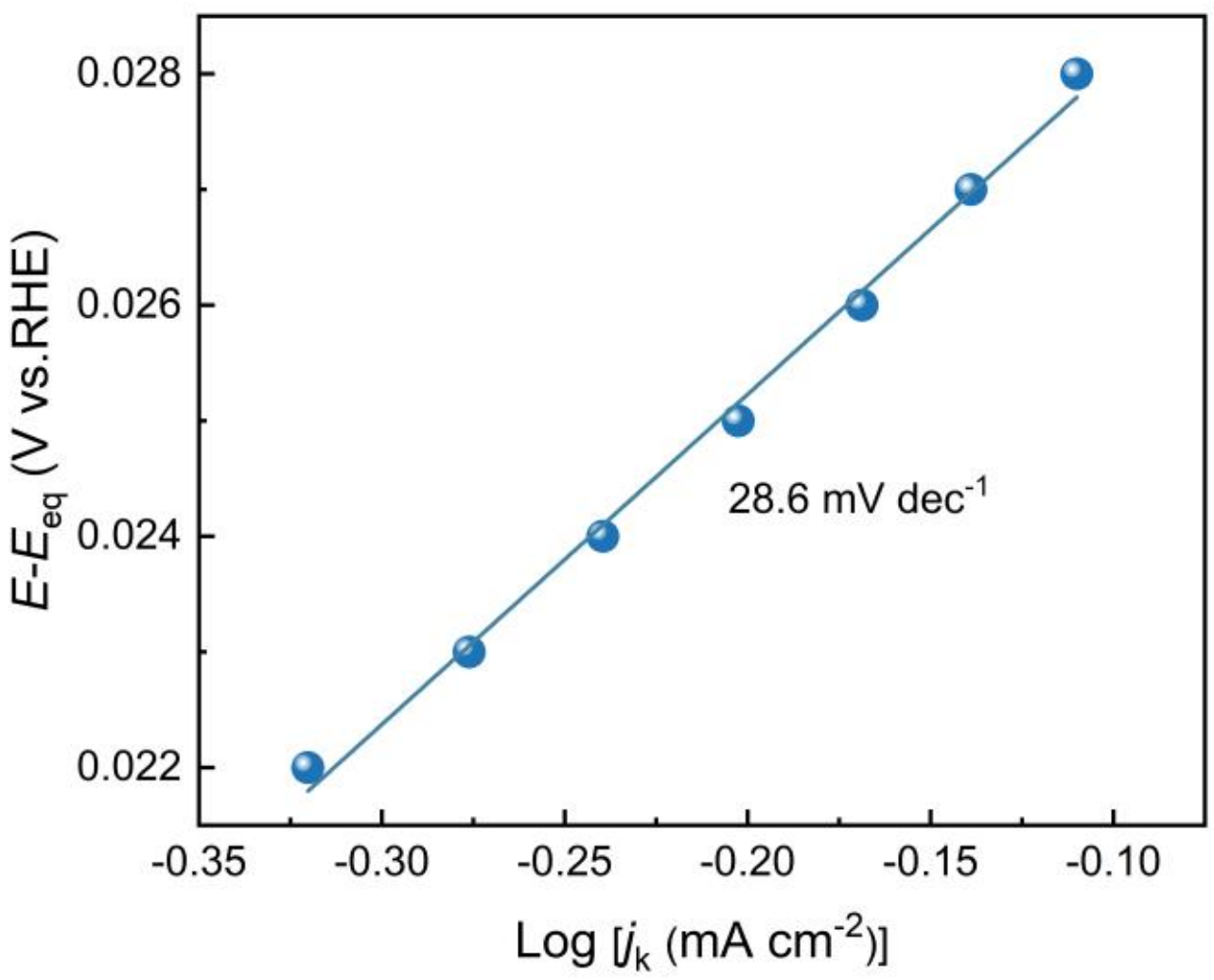

Fig. S23. The corresponding Tafel plot of kinetic current densities for ISA from Fig.4b.

• Even if ISA on an electrode increases HOR current, this does not demonstrate that planktonic C.N–ISA hybrids in bulk solution can provide additional H^+ to the cells. Spontaneous dissociation of H_2 (bond energy = 436 kJ mol^{-1}) by planktonic hybrids without a clear energy input is hard to believe, and in a buffered medium any H^+ produced locally would be rapidly neutralized.

Response:

We thank the reviewer for this insightful comment, which addresses the fundamental thermodynamic and kinetic principles underlying our biohybrid design. We agree that spontaneous H_2 dissociation is thermodynamically unfavorable in isolation and that the buffering capacity of the bulk solution remains a pertinent consideration. Nevertheless, we respectfully maintain that the efficacy of the planktonic *C.N*@ISA hybrids is underpinned by the synergistic coupling of catalytic activation with cellular metabolism, which establishes a localized non-equilibrium state at the bio-inorganic interface.

1. **Thermodynamic Driving Force provided by Cellular Metabolism**: The reviewer correctly notes that breaking the H-H bond requires energy. However, in our biohybrid system, the ISA catalyst does not operate in isolation. The oxidation of H_2 catalyzed by ISA is thermodynamically coupled to the intracellular electron transport chain (ETC) of *C. necator*. Since *C. necator* is facultatively aerobic (microaerophilic in our reactor), the overall reaction is essentially the oxidation of H_2 coupled to the reduction of terminal acceptors (e.g., O_2 or metabolic intermediates), which is highly exergonic ($\Delta G \approx -237$ kJ/mol for $H_2 + 0.5O_2 \rightarrow H_2O$). The cells act as a continuous “sink” for electrons and protons, providing the necessary thermodynamic driving force to pull the reaction forward. The ISA functions similarly to a natural hydrogenase enzyme: it does not provide energy but lowers the kinetic activation barrier (activation energy) to allow reaction to proceed at ambient conditions.

2**. Localized Proton Accumulation overcoming Buffer Capacity**: We acknowledge the reviewer’s concern regarding proton neutralization in a buffered medium. However, the key to our system is the localized surface microenvironment. The ISA is covalently anchored directly onto the cell membrane via the B-PEG linker. To address this exact concern, we performed confocal laser scanning microscopy (CLSM) using a pH-sensitive fluorescent probe (Fig. 4d).

The results showed a distinct, elevated fluorescence intensity specifically at the *C.N*@ISA cell surface compared to the bulk solution or unmodified cells. This confirms that despite the bulk buffer, the rate of localized H^+ generation by ISA at the membrane interface is sufficiently fast to establish a local proton gradient (part of the proton motive force, PMF) before neutralization occurs. These localized H^+ are then immediately available for uptake by ATP synthase or transport proteins, mimicking the periplasmic proton accumulation in natural respiration [*Nat. Chem.* **15**, 1400-1407 (2023)].

We have revised Section 2.3 to incorporate the discussion.

• The DMPO-H· EPR signals used to claim "hydrogen radical" formation seem suspicious: similar signals appear in the DMPO blank, and side reactions in the electrochemical cell can easily generate DMPO-H· artifacts. This does not prove the existence of bulk H radicals under their conditions.

Response:

We sincerely appreciate the reviewer's professional caution regarding the EPR identification of hydrogen radicals ($H^•$). We agree that potential artifacts from DMPO blanks or electrochemical side reactions must be rigorously excluded to confirm the existence of reactive intermediates. To address this, we have conducted new, systematic EPR experiments in a non-electrochemical system (direct H_2 bubbling) to isolate the catalytic mechanism from any electrochemical interference. As shown in the updated Fig. 4c, the results provide definitive evidence for the ISA-mediated hydrogen activation. The EPR spectra for DMPO alone and DMPO+H_2 are perfectly silent (flat baselines), ruling out any background noise or contamination. By performing these tests in a non-electrochemical setup, we have also eliminated the possibility of electrochemical side reactions (e.g., water splitting intermediates)

that the reviewer cautioned against

We have revised the relevant discussion in Section 2.3 and the methodology description for Method 4.7 in the manuscript.

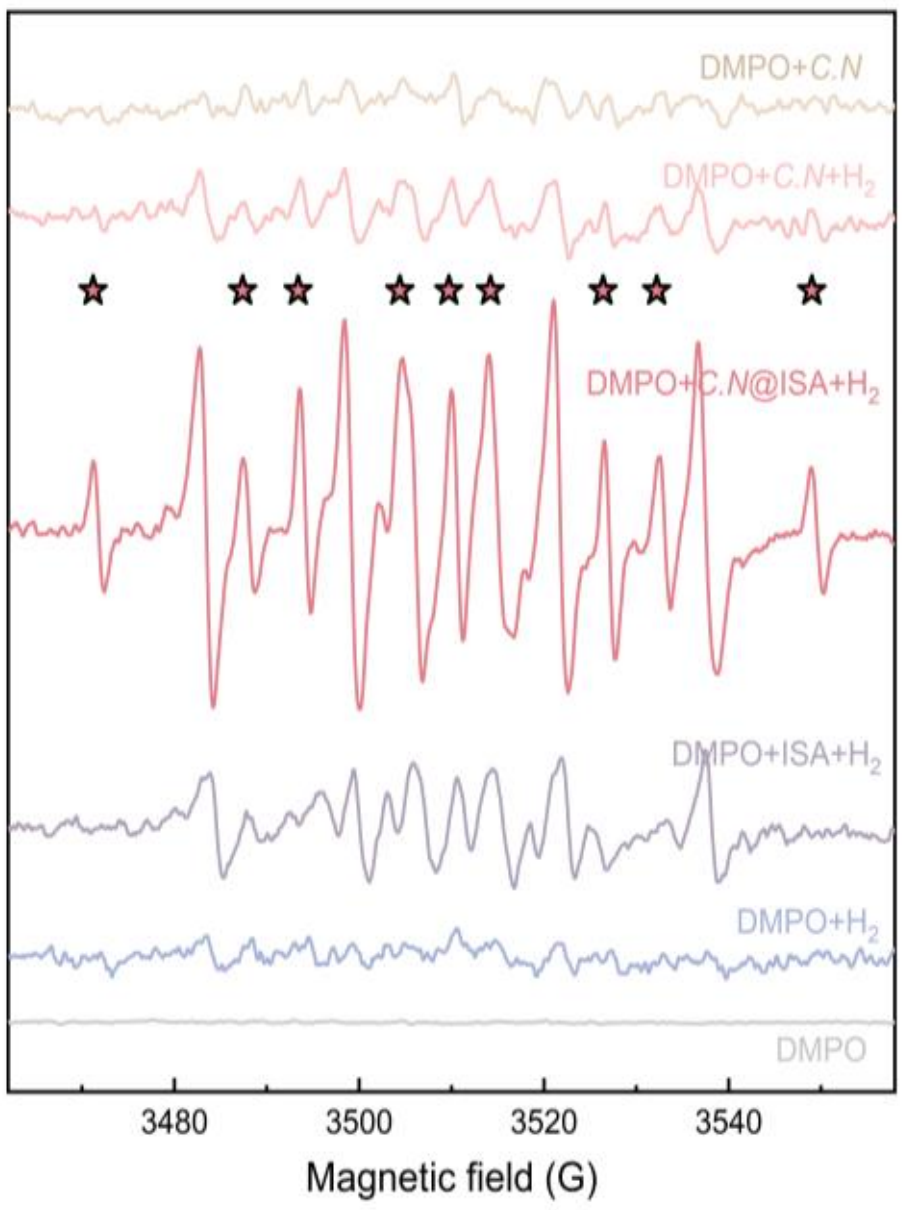

Fig. 4c. Electron paramagnetic resonance (EPR) spectra of spin-trapped adducts. Star symbol (★) indicates DMPO-H signal.

• The DFT calculations are performed on Fe-N_4 motifs in vacuum and track H_2 adsorption and dissociation to H on the surface. While this may have some qualitative relevance for H_2 binding, it does not justify conclusions about free H· or H^+ in buffered solution, nor does it demonstrate that the energetic barriers are overcome in the actual reactor. H*, H· and H^+ are distinct species and should not be conflated, and RRDE alone cannot distinguish between them.*

Response:

We thank the reviewer for the critical and constructive feedback regarding the DFT models and the distinction between hydrogen species. We have addressed these concerns as follows:

1. Clarification on the Computational Model (Solvation Effects): We apologize for the

inadvertent omission of the solvation model details in the original manuscript. The DFT calculations were not performed in vacuum; instead, they were conducted using the Conductor-like Screening Model (COSMO) as an implicit solvation model (ε = 78.54 for water). This approach accounts for the dielectric environment and the polarization effect of the aqueous phase, which enables computational results to more closely align with the qualitative trends observed under realistic conditions compared to vacuum models. We have updated the "DFT calculations and simulations" in Supplementary Information to include these parameters. We acknowledge that while COSMO provides a necessary approximation of the reaction interface, the calculated energetic barriers (ΔG) serve as a thermodynamic benchmark to demonstrate the feasibility of H_2 activation on Fe–N_4 sites under ambient conditions.

2. Distinction Between H*, $H^•$, and H^+: We fully agree that H∗ (adsorbed), $H^•$ (free radical), and H^+ (solvated proton) are distinct species. In our DFT narrative, H∗ refers exclusively to the adsorbed atomic hydrogen on the Fe–N_4 active site. The reference to $H^•$ in our mechanistic discussion was intended to describe the radical-like reactivity of the transiently activated hydrogen, as detected by our EPR spin-trapping experiments (using DMPO), rather than suggesting the existence of stable free radicals in the bulk solution. H^+ is described as the eventual product of the surface-mediated hydrogen oxidation reaction (HOR). We have meticulously revised the manuscript to ensure these terms are not conflated, maintaining a clear distinction between surface intermediates and solvated products.

3. Integration of RRDE, EPR, and Kinetic Evidence: We concede that RRDE measurements alone provide macroscopic kinetic data rather than species identification. However, the convergence of RRDE, Tafel analysis, and EPR provides a robust evidence chain. The EPR signal confirms that the H_2 activation on ISA generates highly reactive intermediates capable of being trapped, while DFT provides the atomic-scale energetic pathway ($H_2 \rightarrow 2H*$).

4. The "Heterolytic-like" Outcome at the Bio-interface: To bridge the gap between molecular-scale simulations and macro-scale observations, we introduced the concept of a 'heterolytic-like outcome'. While the initial activation on the Fe–N_4 motif follows a homolytic-like pathway ($H_2 \rightarrow 2H^*$) as indicated by DFT, the subsequent transfer of these intermediates to the microbial respiratory chain results in the effective separation of protons (H^+) and electrons (e^-). This "functional heterolysis" facilitates the maintenance of the proton motive force (PMF) and drives intracellular metabolism. We have revised the manuscript to clarify that this term describes the integrated interfacial flux, the collective result of catalytic dissociation and biological uptake, rather than a single-step molecular heterolysis event. This distinction directly addresses the transition from the surface intermediates defined in Point 2 to the solvated products utilized by the cell.

These combined theoretical and experimental refinements, we believe, provide a more rigorous justification for the proposed ISA-mediated H_2 activation mechanism. Based on the discussion above, we have updated the caption for Fig. 4e.

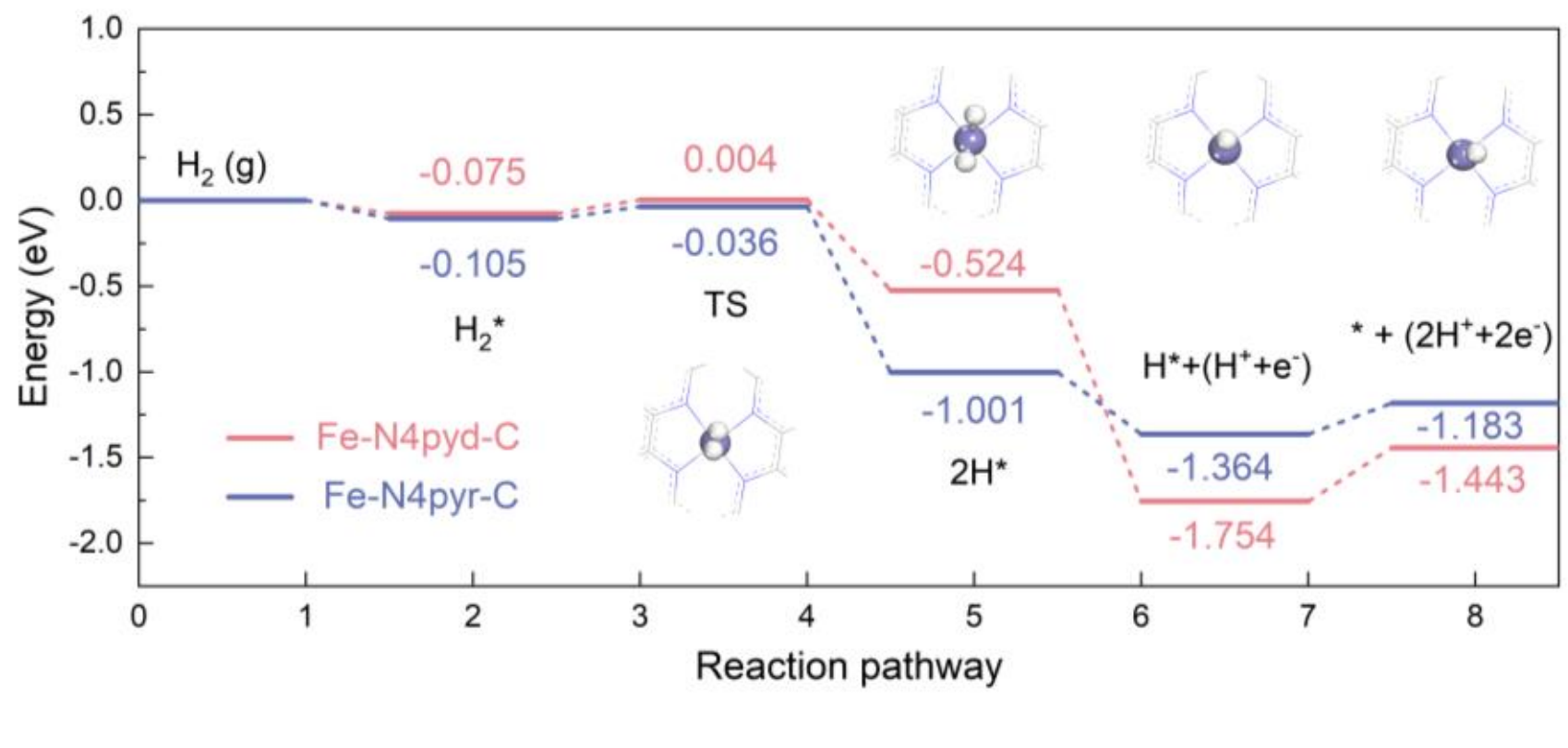

Fig. 4e. Free energy profiles of H_2 dissociation on Fe-N4pyd-C and Fe-N4pyr-C configurations. The illustration profiling the configurations of intermediates on Fe-N4pyd-C. TS: transition state. H*: adsorbed hydrogen.

6. *The EIS data in Fig. 4g are also difficult to interpret. Over the wide frequency range of 0.01–100 kHz, one would typically expect to resolve at least separate high-frequency charge-transfer and lower-frequency transport contributions. The presence of a single semicircle and the chosen equivalent circuit model therefore require more justification. As presented, the EIS analysis does not convincingly support the claim of significantly enhanced electron transfer.*

Response:

We appreciate the reviewer's feedback. We agree that the initial use of a biofilm on carbon cloth was not representative of our actual reactor conditions. Consequently, we have performed new EIS measurements in a suspended-cell setup using a stainless-steel mesh electrode. As shown in new Fig. 4g, no Warburg impedance tail was detected. The possible reason is as follows:

According to the Randles model, the impedance can be written as:

$$Z(\omega) = R_s + [Z_{dl} + (R_{ct} + Z_W(\omega))]^{-1},$$

where the semi-infinite Warburg element is:

$$Z_W(\omega) = \sigma/\sqrt{j\omega}, \quad |Z_W| = \sigma/\sqrt{\omega}$$

A discernible Warburg tail requires that diffusion impedance becomes comparable to or larger than the charge-transfer term at low frequency, i.e., $|Z_W(\omega)| \gtrsim R_{ct}$. However, in our system, the charge transfer resistance is relatively large ($R_{ct} > 2000\ \Omega$), indicating weak interfacial electron transfer between the electrode and the electrolyte. The response is dominated by kinetic/near-blocking behavior and the diffusion contribution is expected to be negligible or experimentally indistinguishable. This is in fact reasonable for our experimental conditions, because direct electron transfer between the electrode and suspended bacteria can be neglected. Although some redox-active species are present in the medium (as indicated by CV and HPLC

results), their bulk concentrations are low (on the order of ~0.1 ppm), and they may undergo adsorption at the microbial interface. This is consistent with the architecture where the system is predominantly mediated by H_2 rather than by direct electron transfer. In the revised version, discussion has been updated in Section 2.4 and Method 4.8 have been updated accordingly.

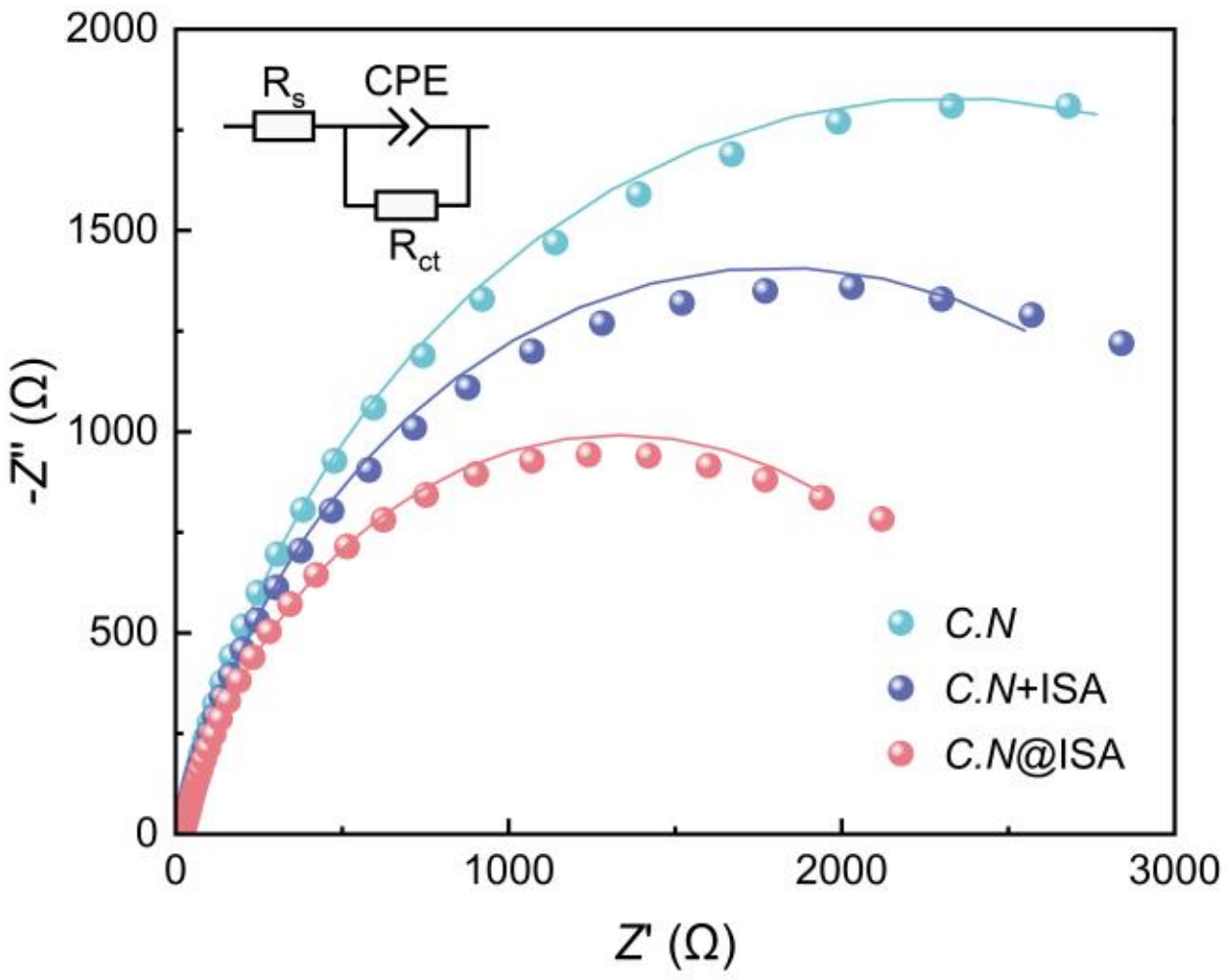

Fig. 4g. Nyquist plots and equivalent circuit fitting plots of the operating integrated system. *C.N*@ISA, *C.N*+ISA, *C.N* are all suspended in the electrolyte.

7. *The performance comparison in Fig. 6b is not rigorous. Comparing final PHB concentrations across different studies without normalizing for electrode area, operating time, or cell density is not meaningful. The apparent "record" performance could be largely due to longer operation or larger electrode area. Production rates (e.g., mg L^{-1} day^{-1} or mg cm^{-2} day^{-1}) should be used for fair comparison, and even the underlying reactor designs differ substantially.*

Response:

We appreciate the reviewer's constructive suggestion. We fully agree that comparing final PHB titers in isolation may yield misleading conclusions due to variations in experimental durations.

Accordingly, we have revised Fig. 6b to present PHB productivity rates, defined as the average rate from the initiation of operation to product quantification, rather than cumulative titers. Furthermore, Table S2 in the Supplementary Information has been systematically updated to compare the productivity rates of the present study with those reported in literature, ensuring a more rigorous and equitable performance benchmark.

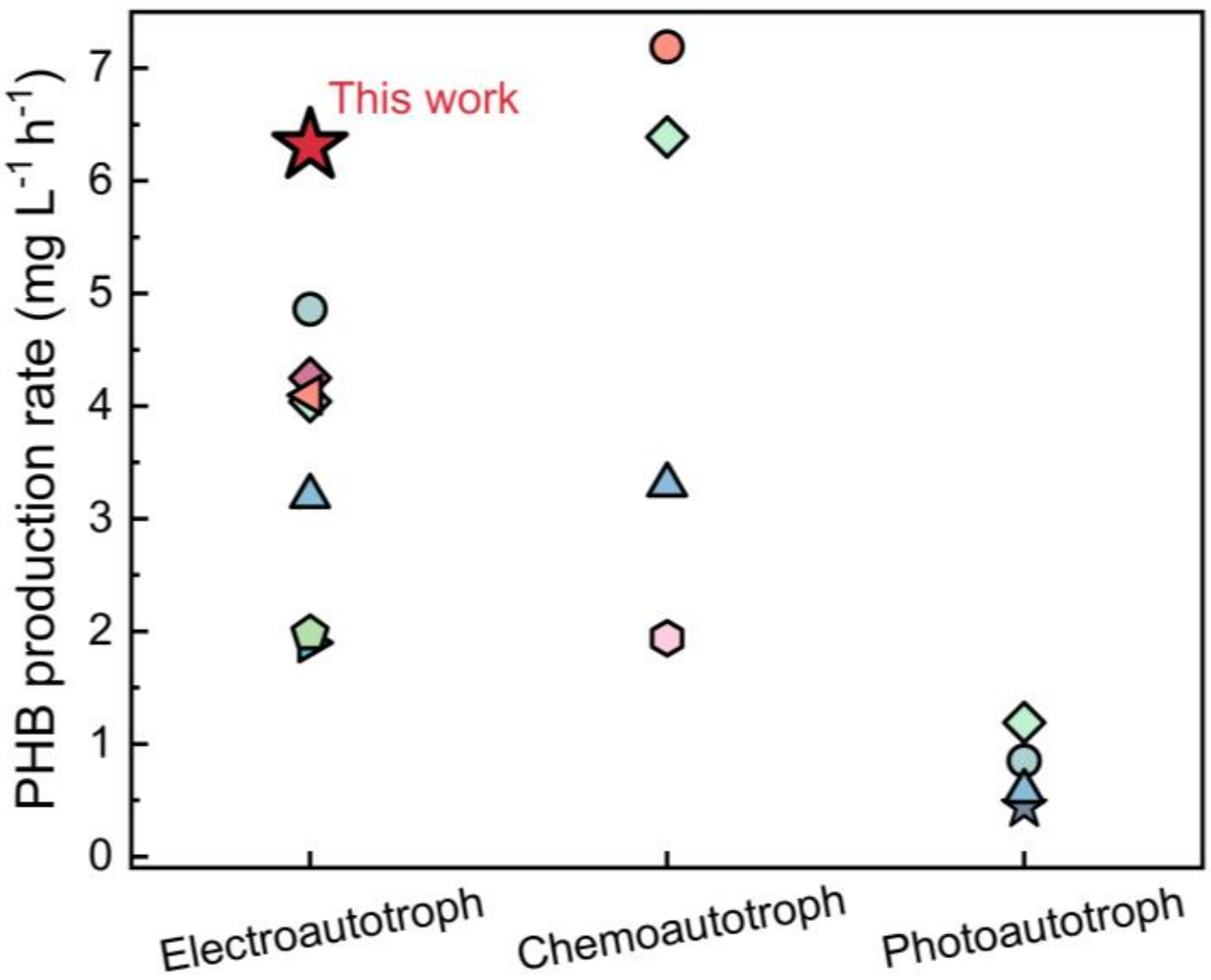

Fig. 6b. Comparison of PHB production rates in this work with previously reported systems.

Response to reviews

We would like to express our sincere gratitude to the reviewers for their profound insights and constructive critiques of our manuscript. Addressing these comments has enabled us to gain a deeper and more comprehensive understanding of our research findings. We have provided point-by-point responses to all comments and have revised the Manuscript and Supplementary Information accordingly. We believe that these modifications have helped improve the overall quality and scientific rigor of the work. For clarity, the reviewers' original comments are presented in *italics and underlined*, and all corresponding revisions in the text are highlighted in **yellow**.

Reviewer #3 (Remarks to the Author):

The revised version meets most of my concerns. If possible, some self-evaluated words can be removed, such as "record high". In fact, the kind of conclusions can only be obtained under a series of limitations. For example, the PHB production by C. necator has already been over 200 g/L for industrialization, and a few gram/L levels are very common in lab scale reports, even in some hybrid systems.

Response:

We thank the reviewer for this helpful comment and agree that expressions such as "record high" are subjective and not appropriate given the variability across systems and conditions. Accordingly, we have removed such wording throughout the manuscript and revised the relevant statements to present the results in a more objective and balanced manner. The specific revisions are as follows:

- **Abstract**: the term "record high" has been removed.
- **Introduction (Paragraph 4)**: "record-high" has been revised to "efficient" ("…led to efficient CO_2-to-PHB conversion").
- **Discussion (Section 2.6, Paragraph 1)**: "record-high" has been removed; "far exceeding previous studies" has been revised to "higher than those reported in several previous studies".
- **Discussion (Section 2.6, Paragraph 3)**: "record-high" has been revised to "enhancing" ("…thereby enhancing biomass accumulation and PHB production…").
- **Conclusion**: "record-high" has been removed.

Reviewer #4 (Remarks to the Author):

I think the manuscript has improved - some issues have been partially addressed, but several key concerns remain unresolved. In particular, some of the newly added supplementary data and cited references do not fully support the authors' statements and, in certain cases, may even contradict the proposed mechanism. More importantly, there are several inconsistencies in experimental data for identical samples between the original submission and the revised manuscript. Such discrepancies substantially reduce confidence in the reliability of the reported results.

Response: We sincerely thank the Reviewer for the rigorous and constructive re-evaluation of our manuscript. We fully acknowledge that the concerns raised — regarding the adequacy of newly added supplementary data and cited references in supporting the proposed mechanism, as well as the inconsistencies in experimental data between manuscript versions — are of fundamental importance to the reliability of our work. The Reviewer's comments have been invaluable in pushing us toward a higher standard of data accuracy and expressional rigor. In this revision, we have carefully re-examined each of these issues and addressed them in the detailed point-by-point response below. We hope our responses adequately resolve the Reviewer's concerns.

Detailed comments:

1. As clarified in the authors' response, the C.N@ISA hybrids are in a planktonic state, and no obvious attachment to the stainless-steel electrode surface was observed. Under such conditions, some experimental observations and interpretations presented by the authors become difficult to explain:

a) If ISA is not in direct contact with the stainless-steel electrode, measuring HOR activity using ISA loaded glassy carbon electrode becomes irrelevant and cannot rigorously demonstrate that ISA promotes H_2 dissociation in the actual system. For Fe single-atom catalysts, enhanced current relative to bare glassy carbon is expected under an applied overpotential simply due to the presence of catalytic sites. However, in the freely suspended C.N@ISA hybrid, no overpotential can be applied to ISA to drive H_2 oxidation. Therefore, the HOR measurements on ISA-modified electrodes cannot directly support the claimed H_2 activation mechanism in the biohybrid system.

Response:

We thank the reviewer for this incisive comment, which has prompted us to sharpen both the

positioning of our electrochemical measurements and the mechanistic reasoning for H_2 activation in the biohybrid system. We fully agree that macroscopic HOR measurements on ISA-modified glassy carbon electrodes cannot replicate the operating conditions of freely suspended C.N@ISA particles, and that this distinction warrants explicit clarification. We address the reviewer's two concerns separately below, and have revised the manuscript accordingly (Section 2.3, paragraph 2, and Methods Section 4.7).

The HOR and RRDE experiments were not designed to mimic the biohybrid working environment, but to serve a distinct and complementary purpose: to establish, under well-defined electrochemical conditions and independent of biological complexity, the *intrinsic* capability of ISA to catalyze H_2 dissociation. This benchmark is methodologically necessary because it isolates the catalytic behavior of ISA from any cellular contribution, thereby providing an unambiguous reference point against which the in-cell observations can be interpreted. We have revised Section 2.3 to state this scope explicitly, simplified the Tafel-based discussion, and moderated the mechanistic language so that the electrochemical data are no longer invoked as direct evidence for the in-cell mechanism, but rather as evidence for the intrinsic H_2-activation competence of the ISA catalyst itself.

We agree with the reviewer that ISA in planktonic C.N@ISA particles does not experience an electrode-imposed overpotential, and this is precisely the point at which the biological component assumes its role. ISA does not function in isolation: its H_2 dissociation activity is coupled, through the cell envelope, to the intracellular electron and proton demand of *C. necator*. Under the microaerophilic conditions of our reactor, H_2 oxidation is ultimately coupled—via $NAD(P)^+$, flavin cofactors, the respiratory chain, and the Calvin–Benson–Bassham cycle—to the reduction of terminal acceptors and to CO_2 fixation, rendering the overall process thermodynamically downhill. The cellular metabolic network therefore acts as a continuous electron and proton sink, providing the thermodynamic driving force that the external circuit supplies in a conventional electrochemical cell. Within this framework, ISA plays a role *kinetically analogous* to that of a natural [NiFe]-hydrogenase: it does not supply reaction energy, but lowers the activation barrier for H_2 heterolytic cleavage, consistent with the DFT-calculated barrier of 0.079 eV (Fig. 4e). We emphasize that we present this as a proposed functional analogy rather than a claim of mechanistic equivalence; the precise molecular pathway by which the dissociated H atoms/protons and electrons are relayed from

the ISA surface to intracellular cofactors remains an open question that we explicitly flag in the revised discussion as warranting dedicated future investigation.

We respectfully note that the manuscript does not rest the mechanistic argument on HOR data alone. Two independent lines of evidence, obtained *on the intact, operating biohybrid system*, directly support H_2 activation at the ISA–cell interface. First, DMPO spin-trapping EPR measurements performed under working conditions reveal a pronounced DMPO–H adduct signal in the C.N@ISA group that is absent in the relevant controls (Fig. 4c), providing direct spectroscopic evidence for H-atom generation specifically associated with the hybrid. Second, intracellular ATP levels are markedly elevated in C.N@ISA relative to controls (Fig. 5b), demonstrating that the activated hydrogen species are productively coupled to microbial energy metabolism rather than being spectroscopically visible but functionally inert. Taken together, the DFT-predicted low dissociation barrier, the EPR-detected H-atom intermediates, and the ATP-level response form a self-consistent chain of evidence—theoretical prediction, spectroscopic capture, and metabolic coupling—that supports the proposed role of ISA in the biohybrid, independently of the HOR benchmark.

In summary, we have (i) repositioned the HOR/RRDE results in Section 2.3 as an intrinsic-activity benchmark rather than a model of the biohybrid; (ii) condensed the Tafel analysis and removed mechanistic claims that required an in-cell applied potential; (iii) added a brief statement in Section 2.3 explaining the metabolic-sink thermodynamic framework; and (iv) explicitly noted, in the discussion, that the molecular-level electron/proton relay between ISA and the intracellular cofactor pool remains to be elucidated. We are grateful to the reviewer for prompting these clarifications, which we believe have materially strengthened the rigor of the mechanistic narrative.

b) The RDE results of bare glassy carbon electrode are inconsistent between the two versions of the manuscript. In the first round of review, the authors attributed the unusual RDE behavior to unoptimized testing conditions and inconsistent H_2 saturation. However, the experimental procedure for the bare glassy carbon electrode appears essentially unchanged. It is therefore unclear how the limiting HOR current for bare glassy carbon could change from 1.2~1.8 mA/cm^2 in the original manuscript to essentially zero with no electrochemical response in the revised version. Whether this results from inaccurate data in the initial submission or incomplete updates to the experimental

method, such discrepancies significantly decrease the confidence in the reliability of the reported results.

Response:

We thank the reviewer for carefully identifying this inconsistency and apologize for the insufficient clarification of the methodological update in our previous response.

The reviewer's observation is correct. The discrepancy arises from a change in electrode preparation between the original submission and the revised version. In the original experiments, a small amount of Pt powder was included in the catalyst ink for both the control ("without ISA") and experimental ("with ISA") groups as a conductive additive intended to improve ink dispersion and electrical contact. As a result, the "bare" electrode in the original submission was in fact Pt-containing, which accounts for the observable current (1.2–1.8 mA cm^{-2}) on what was labeled as the GC control. We recognize that this non-standard additive introduces a non-negligible Pt-derived background to the signal of the ISA-containing electrode as well, and should have been disclosed explicitly.

During revision, we therefore reprepared all electrodes without Pt and repeated the entire RRDE dataset for both the control and the ISA-modified working electrodes. The updated polarization curves (Fig. 4b and Fig. S22) show negligible anodic current on the true bare GC across the tested potential window, while the ISA electrode still exhibits a clear, reproducible anodic response in H_2-saturated electrolyte, reaching ~1.6 mA cm^{-2} at +0.10 V vs. SHE. The Pt-free measurement thus unambiguously attributes the anodic activity to the ISA sites themselves.

We emphasize that this re-measurement changes the magnitude of the measured currents but does not alter the qualitative conclusions of the manuscript. The role of ISA in H_2 activation is supported independently by multiple complementary evidence that do not rely on RRDE absolute currents: (i) pronounced DMPO–H EPR signals directly detecting H• radicals in the biohybrid under working conditions (Fig. 4c); (ii) D_2 incorporation into PHB under deuterium atmosphere providing isotopic tracing of H_2 activation (Fig. S25); and (iii) DFT calculations corroborating a low H_2 dissociation barrier of 0.079 eV on Fe-N_4pyd-C (Fig. 4a, e). Consistent with the reviewer's concern that electrode-based measurements cannot fully represent biohybrid conditions (Comment 1a), we have also moderated the RRDE-based mechanistic discussion (Section 2.3, Paragraph 2) and simplified the Tafel analysis accordingly. All conclusions in the current version of the manuscript

are based on this updated and more rigorous dataset.

c) Even without considering the two critical issues discussed above, the updated RDE polarization curves remain questionable. The current response appears to show two quasi-plateau regions (-0.30 to -0.15 V and after ~0.10 V), while the current slightly decreases beyond ~0.10 V. This behavior resembles a broad oxidation peak rather than a well-defined diffusion-limited plateau, making it difficult to determine whether the Fe sites can be oxidized (please check the Pourbaix diagram of Fe). In the HOR field, the characteristic RDE kinetics have been well established (Energy Environ. Sci., 2024, 17, 3960-4009), yet the updated curves, even under optimized conditions with H_2-saturated electrolyte, cannot resemble those expected for a pure HOR process. This raises questions about whether the observed current is truly dominated by HOR rather than other parasitic processes.

Response:

We thank the reviewer for the this very important comment and directing us to consider the Fe Pourbaix diagram. According to the classical Fe-H_2O Pourbaix diagram, the standard redox potential for Fe^{3+}/Fe^{2+} (+0.77 V vs. SHE) lies well above our tested potential window (-0.4 to +0.15 V vs. SHE), which would thermodynamically preclude significant Fe oxidation under our measurement conditions. However, we acknowledge that the classical diagram is strictly derived for free, hydrated Fe species and bulk Fe oxides, and that the local coordination environment of atomically dispersed Fe-N_4 sites can substantially modulate the Fe^{2+}/Fe^{3+} redox potential. To rigorously address whether Fe oxidation contributes to the observed anodic current independently of any specific thermodynamic assumption, we provide the following experimental analyses:

We conducted a control experiment using the exact same ISA electrode in an Ar-saturated electrolyte. As shown in the newly added Fig. S22a, under Ar saturation the current remains slightly cathodic (negative) across the entire tested potential window (approximately –0.4 to –0.15 mA cm^{-2} from –0.4 to +0.15 V vs. SHE), reflecting residual cathodic background processes without any anodic response. Upon switching to H_2-saturated electrolyte, a pronounced anodic current emerges, increasing from near-zero at –0.4 V to ~1.6 mA cm^{-2} at +0.1 V. The net anodic difference between the two conditions ($\Delta J \approx 1.8$ mA cm^{-2} at +0.1 V) is one order of magnitude larger than the Ar-saturated baseline, confirming that the anodic process is dominated by H_2-dependent oxidation

rather than by any parasitic background.

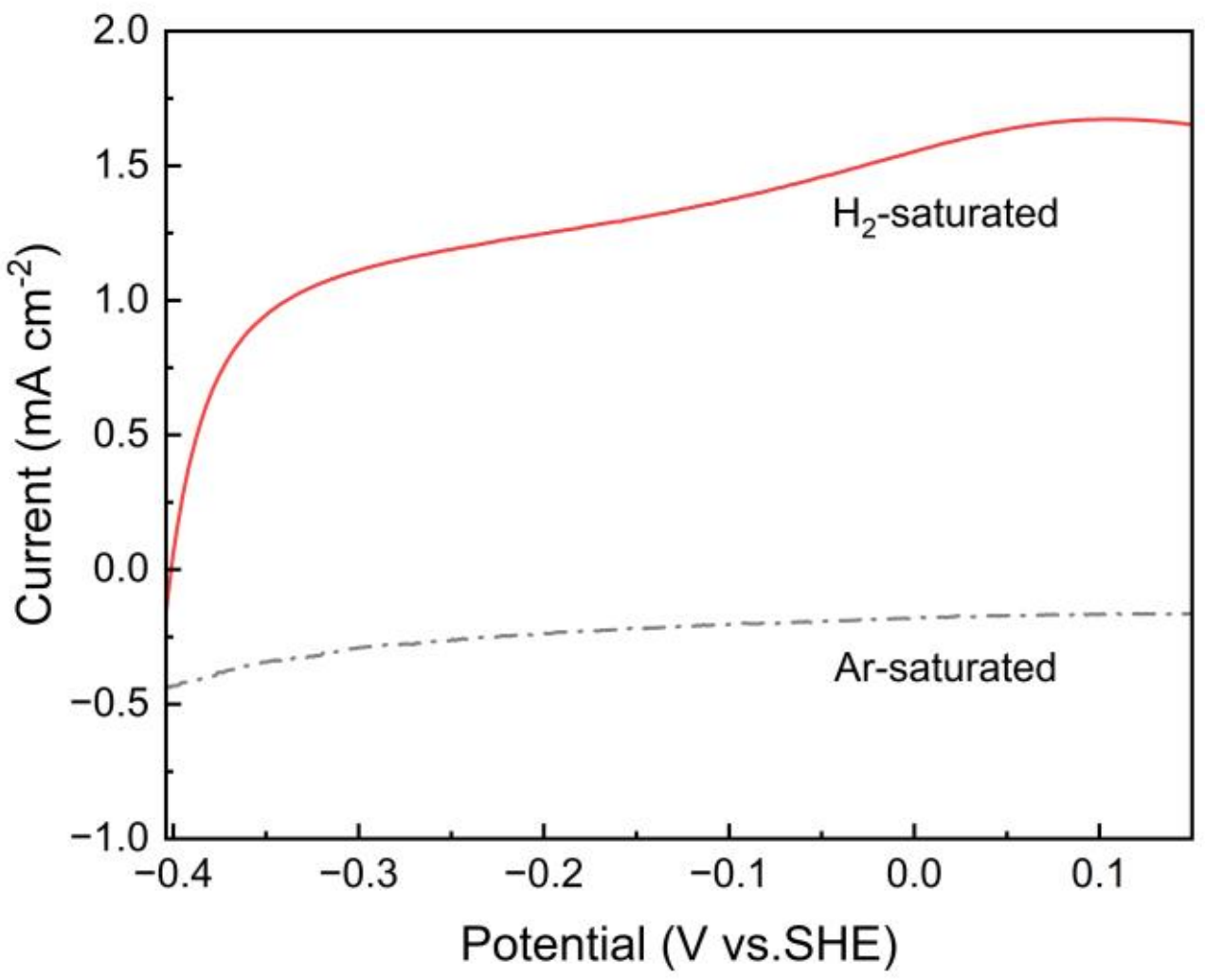

Fig. S22. (a) Polarization curves of ISA acquired in H_2 and Ar-saturated electrolyte. Rotation speed: 1600 rpm. The data were collected with *i*-R correction.

To further evaluate whether the observed anodic current could originate from stoichiometric oxidation of Fe species rather than genuine H_2 electrocatalysis, we performed a quantitative Faradaic charge analysis based on the RRDE measurements. The catalyst loading on the glassy carbon electrode (4 mm in diameter, geometric area ≈ 0.126 cm²) corresponds to ~1.0 µg of Fe. Consistent with the XANES characterization showing an average Fe oxidation state between +2 and +3, the physically relevant upper bound corresponds to a one-electron Fe^{2+}→Fe^{3+} oxidation, yielding a theoretical maximum Faradaic charge of ~1.7×10^{-3} C (this value is itself an overestimate, as only a fraction of the mixed-valence Fe^{2+}/Fe^{3+} population is available for further oxidation). In contrast, integration of the polarization currents in the HOR potential window yields total charges of 6.2×10^{-3}, 7.3×10^{-3}, 8.2×10^{-3}, and 9.2×10^{-3} C at rotation rates of 600, 900, 1200, and 1600 rpm, respectively — all exceeding the stoichiometric upper bound by a factor of 3.6 to 5.4.

Crucially, the measured charge increases monotonically with rotation rate — a defining hallmark of a mass-transport-coupled catalytic process, and one that is fundamentally incompatible with a stoichiometric oxidation confined to a fixed number of Fe sites. Were the anodic current dominated by stoichiometric Fe oxidation, the integrated charge would be determined solely by the amount of available Fe and should therefore be essentially independent of rotation rate. The observed rotation-rate dependence therefore directly evidences a sustained, turnover-based process

fed by the continuous convective supply of dissolved H_2. This conclusion is reinforced by the linear Koutecký–Levich response (Fig. S22b) and the Ar-saturated control (Fig. S22a), which collectively support that the anodic current observed in the H_2-saturated electrolyte is dominated by H_2 electro-oxidation at the Fe-N-C sites.

Taken together, these results suggest that the observed current are most consistent with H_2 oxidation, while contributions from surface processes cannot be entirely excluded. We have revised the corresponding discussion in the manuscript (Section 2.3, Paragraph 2) and Supplementary Information (Fig. S22) to reflect a more cautious and balanced interpretation.

d) The authors emphasize that the C.N@ISA hybrid remains planktonic and does not directly uptake electrons from the electrode. Under such conditions, the charge-transfer resistance (R_{ct}) associated with HER should depend primarily on the stainless-steel electrode and the electrode-electrolyte interface. The planktonic CN, CN+ISA, and CN@ISA should not have significant electrochemical interactions with the electrode. It is therefore difficult to understand why, under the same experimental setup, the authors still report a significant decrease in R_{ct} in the EIS results.

Response:

We thank the reviewer for this insightful comment. We agree that the fitted R_{ct} should not be interpreted as evidence of direct bio-electrocatalytic electron uptake from the stainless-steel electrode, given that the C.N, C.N+ISA, and C.N@ISA cells remain planktonic.

A key question here is why the fitted R_{ct} differs systematically among the three groups despite the shared electrode interface. We attribute this difference to the composition of the electrolyte, rather than to the electrode itself. Specifically, the concentration of extracellular redox-active mediators accumulated during operation differs among the three groups. HPLC and CV analyses (Fig. 4f and Fig. S27) show that the concentration of extracellular flavin species, including FAD, increases monotonically in the order C.N < C.N+ISA < C.N@ISA, which mirrors the monotonic decrease of the fitted R_{ct} from 4604 to 3573 to 2624 Ω (Table S2). Mechanistically, the FAD/$FADH_2$ redox couple introduces an additional Faradaic pathway at the electrode interface that operates in parallel with the pre-existing background processes; in a Randles-type equivalent circuit, this parallel Faradaic branch lowers the total interfacial resistance extracted from the fit, with a larger effect at higher mediator concentration. Secondary contributions, such as partial electrode coverage

by cells or extracellular polymeric substances, cannot be strictly excluded, but these factors alone do not readily account for the strict one-to-one correspondence between mediator abundance and fitted R_{ct}.

Taken together, the EIS results are more appropriately understood as reflecting mediator-modulated interfacial Faradaic processes in the biohybrid electrolyte, rather than HER kinetics or direct electron transfer from the electrode to the cells. This interpretation is fully consistent with the design of our system, in which intracellular reducing equivalents are primarily derived from ISA-facilitated H_2 activation and subsequent metabolic coupling, not from direct electrode-to-cell electron transfer. We have accordingly revised Section 2.4 to clarify this interpretation and to remove any wording that could be read as overstating the mechanistic meaning of the R_{ct} changes.

e) Moreover, the equivalent-circuit fitting for the updated EIS results does not appear well sufficiently justified. and the confidence in the fitting seems limited. The authors should carefully re-examine the fitting procedure, consider alternative models if necessary, and report fitting errors or confidence levels.

Response:

We sincerely thank the reviewer for the careful evaluation of our EIS analysis and for highlighting the need for further clarification of the equivalent circuit model. In response, we have re-examined the fitting procedure, clarified the physical basis of the selected model, and included an error analysis to assess the reliability of the fitting results.

The impedance spectra were fitted using a Randles-type circuit, specifically $R_s(QR_{ct})$ (a solution resistance in series with a parallel constant-phase-element/resistance pair):

R_s: Represents the uncompensated ohmic resistance of the electrolyte and cell configuration.

Q: The non-ideal capacitive behavior at the stainless-steel electrode interface is described using a constant phase element (*CPE*, *Q*) instead of an ideal double-layer capacitor (C_{dl}), reflecting interfacial heterogeneity and surface roughness. This response arises from a combination of factors, including the distribution and dynamics of ions within the electrical double layer.

R_{ct}: It reflects the resistance associated with Faradaic charge transfer processes at the electrode–electrolyte interface, including background reactions and redox-active species present in the solution. In this context, soluble mediators such as FAD are expected to contribute significantly

to the interfacial electron transfer processes.

The parallel configuration of Q and R_{ct} arises from the coexistence of capacitive (non-Faradaic) and Faradaic processes at the interface, which contribute simultaneously to the total current response. We note that introducing additional circuit elements may lead to over-parameterization without clear physical justification. Moreover, no pronounced diffusion-controlled feature was observed within the measured frequency range, supporting the use of this model.

To evaluate the robustness of the fitting, we extracted the parameter values along with their corresponding relative standard errors (RSE, %). As shown in the revised Table S2, the RSE values for the key parameters (R_s, Q, and R_{ct}) are consistently below 2% across all samples, indicating excellent fitting stability and low statistical uncertainty. While low RSE values do not uniquely validate the physical correctness of the selected equivalent circuit, they do demonstrate that the chosen model provides a consistent and reliable mathematical description of the experimental data. Combined with the physically reasonable interpretation of each circuit element, this supports the appropriateness of the R_s (QR_{ct}) model for our system.

Table S2. Parameter estimates and fitting errors for the EIS data.

Group	*C.N*	*C.N*+ISA	*C.N*@ISA
R_s (Ω)	20.95 (0.37%)	20.84 (0.42%)	20.07 (0.46%)
Y_0 (S·sec^n)	1.551×10^{-3} (0.54%)	1.698×10^{-3} (0.63%)	1.980×10^{-3} (0.71%)
n	0.855 (0.26%)	0.850 (0.30%)	0.824 (0.35%)
R_{ct} (Ω)	4604 (1.75%)	3573 (1.67%)	2624 (1.76%)

2. Similar to the baseline issue noted in Comment 1(b), the EPR results also show inconsistencies between the original and revised manuscripts. The noise strength of the DMPO-only blank control differs substantially between the two versions (see figure below). Moreover, the authors state that DMPO+H_2 is also a blank control measured without electrochemical operation, yet its baseline noise strength is markedly higher than the DMPO-only spectrum. The reason for these discrepancies is unclear.

Response:

We thank the reviewer for the careful examination of our EPR spectra and apologize for the

insufficient clarification of the sample preparation in the previous version.

In response to the reviewer's earlier comment regarding the relatively large background noise in the EPR spectra, we carefully investigated the possible causes and suspected that the DMPO reagent used in the previous measurements, having been stored and used over an extended period, may have suffered from potential contamination or partial degradation — a known issue for DMPO, which is sensitive to light, heat, and oxidation. To eliminate this uncertainty, we purchased a brand-new batch of DMPO (J&K Scientific, cat. no. LK11041, ≥97%) and repeated all EPR experiments from scratch using the fresh reagent. The spectra presented in the revised manuscript are therefore entirely new datasets acquired under fully controlled and consistent conditions, which accounts for the differences in baseline and signal features compared with the original version.

We would also like to clarify an important point regarding the sample matrices, which was not explicitly stated in the previous submission. The DMPO-only spectrum was acquired with DMPO dissolved in pure water and was intended as a reagent purity check to verify the absence of artifactual EPR features from the freshly purchased DMPO stock. In contrast, all experimentally meaningful samples (including DMPO + H_2, DMPO + H_2 + C.N, DMPO + H_2 + ISA, and DMPO + H_2 + C.N@ISA) were prepared in the minimal medium (MM) used for bacterial cultivation, in order to reproduce the chemical environment of the biohybrid system.

The elevated baseline of the DMPO + H_2 spectrum relative to the DMPO-only (pure water) reference therefore reflects the background EPR response of the MM matrix, which contains trace paramagnetic species that contribute a broad, unresolved background signal. This type of matrix-derived background is a well-recognized feature in EPR studies of biological or medium-containing systems and does not indicate the presence of spin adducts.

For direct comparability, the four experimental samples that carry the mechanistic conclusions (DMPO + H_2, DMPO + H_2 + C.N, DMPO + H_2 + ISA, and DMPO + H_2 + C.N@ISA) were all prepared in the same MM matrix and measured on the same instrument within the same experimental session using identical acquisition parameters. These four spectra share an equivalent baseline and can therefore be compared directly.

Critically, among these four MM-based spectra, only the DMPO + H_2 + C.N@ISA group exhibits the characteristic 9-line hyperfine pattern of the DMPO–H adduct (Fig. 4c, marked with red stars), which is absent in all three controls. This qualitative spectral feature, being independent

of baseline amplitude, robustly supports the conclusion that H_2 dissociation occurs at the biohybrid interface.

To avoid any further ambiguity, we have added an explicit note in Section 4.7 specifying the solvent matrix, sample preparation protocol, and EPR acquisition parameters for each spectrum shown in Fig. 4c.

3. In the first round of review, we raised concerns regarding the thermodynamics and kinetics of spontaneous H_2 dissociation. The authors responded that the system may form a chemical pathway like a hydrogen-oxygen fuel cell when coupled with facultative microbes. While such a mechanism may be possible in the presence of living cells or extracellular redox mediators, the author's explanation will also imply that spontaneous H_2 dissociation on ISA alone should be extremely difficult in the absence of these biological components. This leads to a critical question: in the EPR experiments, how are the H radicals detected in the DMPO+ISA+H_2 group (even though the signal intensity is relatively weak)? If the H_2 dissociation only by ISA is very difficult or even infeasible, where are the H radicals from?

Response:

We thank the reviewer for raising this important question. A comparison of the EPR spectra from the "DMPO+*C.N*@ISA+H_2" and "DMPO+ISA+H_2" groups reveals a clear difference in their signal features.

In the biohybrid system ("DMPO+*C.N*@ISA+H_2"), a characteristic 9-line EPR spectrum is observed (marked with red stars), which is consistent with the formation of DMPO–H spin adducts. In contrast, the signal in the "DMPO+ISA+H_2" group does not exhibit this 9-line pattern, but instead appears as a weaker 6-line feature.

The 6-line signal may be associated with interactions between the carbon-based catalyst surface and DMPO, potentially involving surface defects, and the possibility of forming a carbon-centered adduct (DMPO–R) cannot be excluded.

Therefore, the absence of a characteristic DMPO-H signal in the "DMPO+ISA+H_2" group suggests that ISA alone is unlikely to efficiently promote H_2 dissociation under these conditions. This observation is consistent with both our previous thermodynamic analysis and the reviewer's perspective, highlighting the importance of the biological environment in facilitating the formation

and utilization of reactive hydrogen species. We appreciate the reviewer for prompting us to clarify this point, which helps to improve the rigor of our mechanistic interpretation.

4. When checking the reference (Nat Commun 16, 8141 (2025)) provided by the authors, the oxidation and reduction potentials of flavins are -0.1V and -0.14V vs. SHE, respectively. The authors may have to explain the redox potential shift in the updated Figure 4f.

Response:

We thank the reviewer for this insightful comment. We agree that the redox potentials of flavins reported in the cited reference (*Nat. Commun.* **16**, 8141 (2025)) fall around -0.10 V and -0.14 V vs. SHE under their specific experimental conditions.

However, it is well established that the redox potentials of flavins are not fixed constants but are highly dependent on the physicochemical conditions and molecular interactions. For example, previous studies on *Cupriavidus necator* have shown that flavin cofactors can exhibit multiple redox transitions over a broad potential window (approximately -0.4 to -0.1 V vs. SHE, pH = 7.5), depending on their redox state and microenvironment (*J. Am. Chem. Soc.* **145**, 25850-25863 (2023)).

In our system, the observed ~20 mV positive shift may arise from several factors. Differences in electrode materials and interfacial properties can influence flavin redox behavior. In addition, variations in solution conditions, including pH and ionic composition, can affect the redox processes of flavins. Furthermore, local microenvironmental effects, such as electrostatic interactions and hydrogen-bonding networks, may alter the stabilization of oxidized and reduced states. Such effects have been widely reported in flavoprotein systems (*Proc. Natl Acad. Sci. USA* 110, 7856-7861 (2013), *Angew. Chem. Int. Ed.* **63**, e202412072 (2024)).

5. Besides, for the HPLC results of FAD detection, obviously, there is a position shift of the claimed FAD peak, we suggest that the authors should be very careful about the attribution of this peak. Maybe NMR could be helpful, but not necessary.

Response:

We thank the reviewer for this valuable comment. We agree that retention time alone in complex biological matrices can be insufficient for unambiguous peak assignment, and that the slight position shift observed in Fig. S27 warrants careful interpretation. This shift is a recognized

matrix effect: the biohybrid samples are derived from a complex minimal medium containing high salt concentrations and cellular byproducts, which slightly alters the local equilibrium on the stationary phase compared with standards dissolved in pure solvent.

To directly confirm the identity of the observed peak as FAD, we performed a standard-addition (spike) experiment (Fig. S27b) in an independent HPLC run. Aliquots of the biohybrid sample (containing an apparent FAD concentration of 0.15 ppm with a peak area of 2.3 by external calibration) were spiked with authentic FAD standard at two levels (0.25 and 0.40 ppm), giving total FAD concentrations of 0.40 and 0.55 ppm. Re-analysis under identical HPLC conditions within the same run yielded peak areas of 6.1 and 7.8, respectively, at the same retention time as the original target peak, with excellent linearity through the origin ($R^2 = 0.998$). No new peaks emerged at retention times distinct from the target peak region upon spiking, and the target peak increased proportionally with the spike amount while retaining its characteristic shape and position, indicating that the spiked FAD co-elutes with the endogenous target peak. Together, these observations directly confirm the assignment of the observed peak to FAD.

We note that the spike experiment was conducted approximately four months after the original HPLC measurements (Fig. S27a), and the absolute retention time of FAD in the spike experiment differs **from** that in the initial analysis due to gradual column aging over this period. This is a well-known phenomenon in reversed-phase HPLC arising from cumulative use of the stationary phase. Importantly, this does not affect the validity of the spike experiment, since all three samples (biohybrid sample, +0.25 ppm spike, +0.40 ppm spike) were analyzed within the same run under identical chromatographic conditions. The co-elution of the endogenous peak with authentic FAD standard within this internally consistent spike experiment provides rigorous identification that does not depend on absolute retention time comparisons across different runs.

Regarding the suggested NMR confirmation: the extracellular FAD concentration in our system is approximately 0.2 ppm (~0.25 μM), more than an order of magnitude below the typical ^{1}H NMR detection limit for dilute aqueous solutions (~10 μM with conventional probes), and direct analysis of complex biological matrices would be dominated by overlapping signals from medium components. NMR is therefore not readily feasible under the current experimental conditions. Nevertheless, the standard-addition result provides rigorous and independent confirmation of the peak assignment to FAD.

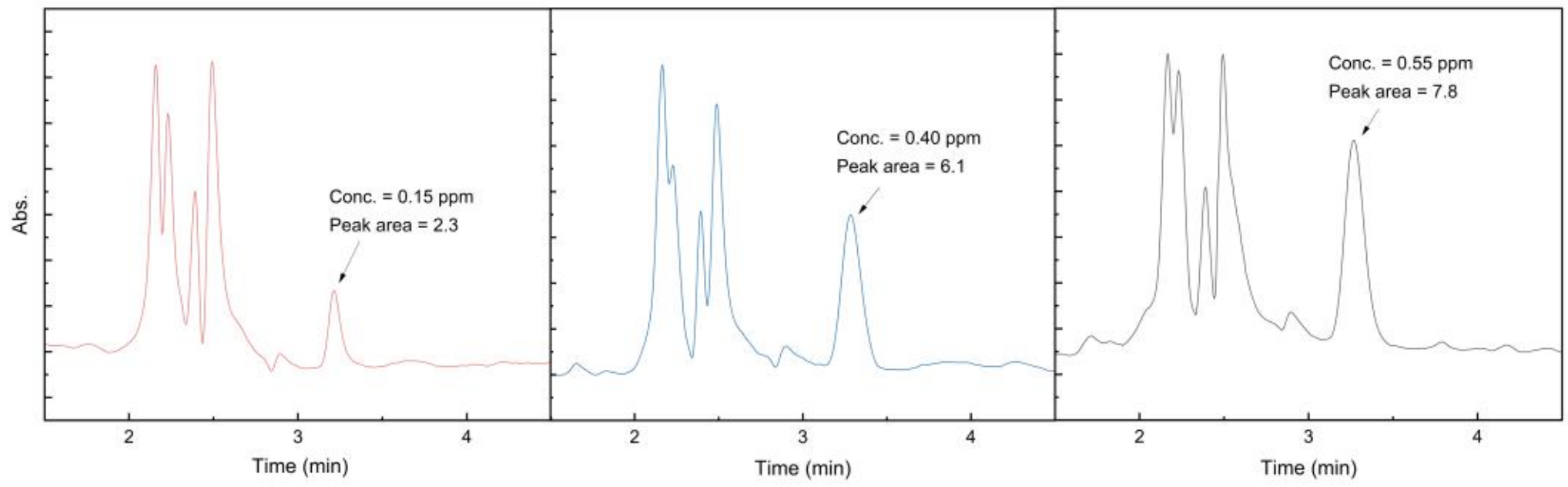

Fig. S27b. Standard-addition confirmation of FAD peak assignment. HPLC chromatograms of the biohybrid sample (left, 0.15 ppm endogenous FAD) and the same sample spiked with FAD standard to total concentrations of 0.40 ppm (middle) and 0.55 ppm (right). The target peak (~3.2 min, arrows) increases proportionally with the spike amount (R^2 = 0.998) while maintaining the same retention time, confirming its assignment to FAD. Peaks at shorter retention times (~2.0–2.5 min) correspond to background medium components.

Reviewer #5 (Remarks to the Author):

To avoid bias, I first read the original manuscript and the revised version, without consulting the prior reviewer reports. In my opinion, this manuscript describes an interface engineering strategy that anchors iron single-atom catalysts onto Cupriavidus necator via click chemistry, achieving efficiency CO2-to-PHB conversion with enhanced Faradaic efficiency. Then I carefully read the rebuttal letter, the authors have conducted a careful revision, addressing the major concerns (including some questions raised in my independent review before read the rebuttal letter) through substantial new experimental data, including extended stability tests, isotopic labeling, quantitative ROS measurements, competitive binding assays, and a recalibration of biomass quantification. The core findings are now methodologically sound and convincingly supported.

I have found some additional questions in the manuscript that touch upon the long-term chemical stability of the active sites, the partitioning of electron flux, etc. These points do not undermine the validity of the conclusions, but should be properly addressed before publication in Nature Communications. Here are my specific comments:

Response:

We sincerely thank the reviewer for the rigorous and unbiased evaluation of our manuscript, and particularly for independently assessing both versions prior to consulting the rebuttal letter. We

are encouraged by the reviewer's recognition of our interface engineering strategy and the acknowledgment that the major concerns have been adequately addressed through the additional experimental evidence, and that the core findings are now methodologically sound and convincingly supported.

We also appreciate the reviewer's further constructive comments regarding the long-term chemical stability of the active sites and the partitioning of electron flux. These points are addressed in detail in our point-by-point responses below.

1. The 360-hour fed-batch experiment demonstrates sustained ISA decoration on cells, yet the chemical state of the Fe–N4 moieties after prolonged operation remains uncharacterized. Is there any indirect or direct evidence indicating whether the coordination environment undergoes gradual alteration under continuous exposure to bacterial metabolites, local pH fluctuations, and reactive oxygen species?

Response:

We thank the reviewer for this insightful question. Following the reviewer's suggestion, the chemical state of the $Fe-N_4$ moieties after prolonged operation (denoted as ISA-after) was characterized via synchrotron radiation X-ray absorption spectroscopy (XAS). The Fe K-edge X-ray absorption near-edge structure (XANES) spectra (Fig. S41a) showed that the absorption edge of ISA-after is located between those of FeO and Fe_2O_3, suggesting that Fe exists in a mixed oxidation state between +2 and +3. The similar Fe K-edge XANES spectrum of ISA-after to that of pristine ISA indicated that the average valence state of Fe is essentially preserved. Fourier transform EXAFS (FT-EXAFS) spectrum (Fig. S41b) revealed distinct peak at 1.70 Å (Fe-N coordination) in ISA-after, indicating that the Fe-N coordination was also maintained after long-term exposure to bacterial metabolites, local pH variation, and ROS stress. Notably, no discernible Fe–Fe scattering signal (2.2 Å) is observed, suggesting that the isolated Fe atoms do not aggregate into Fe clusters or nanoparticles during the 360-hour fed-batch experiment. Moreover, wavelet transform (WT) analysis (Fig. S41c) of the FT-EXAFS spectra provided additional insight into the spatial distribution of the coordination environment. The WT signal of ISA-after did not exhibit the delocalized distribution features of Fe-Fe metallic bonds in the low-k region but instead matched the typical distribution of Fe-N coordination, which was consistent with FT-EXAFS spectrum.

Furthermore, least-squares EXAFS fitting calculations (Table S4) clarified that the first coordination shell of Fe in ISA-after consists of approximately 4 nitrogen atoms with an average Fe-N bond length of 2.2 Å, indicative of a well-defined coordination environment. XANES simulations (Fig. S41d) showed good agreement with the Fe-N_4 structural model, corroborating the maintaining of atomically dispersed Fe-N_4 coordination. Although the existence of Fe-N_4 coordination, the Fe–N peak shifts toward high R(Å), which indicates the Fe–N bond is stretched after prolonged operation. This is because of the sustaining adsorption and desorption of reactive intermediates on active site. Overall, both the post-operation characterizations and the sustained catalytic performance indicate that the Fe single-atom sites retain a largely preserved Fe–N_4 coordination environment during long-term operation in the complex biological system. The relevant discussion has been added into the revised manuscript on **Section 2.3 Paragraph 2.**

To further probe the structural stability of the catalytic centers, the chemical state of the Fe-N_4 moieties after prolonged operation (denoted as ISA-after) was characterized via synchrotron radiation X-ray absorption spectroscopy (Fig. S41). The XANES and EXAFS analysis demonstrates the maintaining of Fe-N_4 structure. Although the existence of Fe-N_4 coordination, the Fe-N peak shifts toward high R(Å), which indicates the Fe-N bond is stretched after prolonged operation.This is because of the sustaining adsorption and desorption of reactive intermediates on active site.

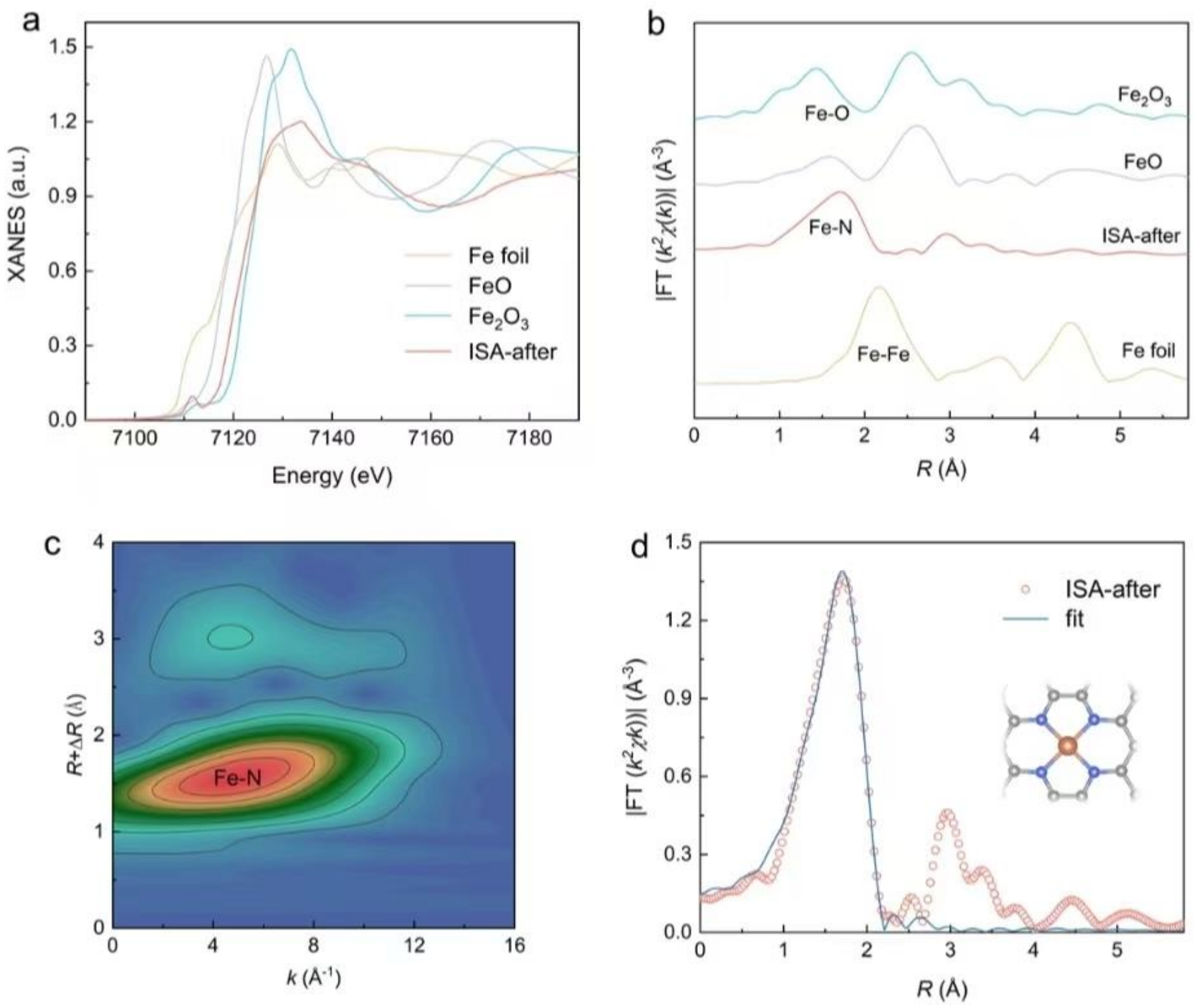

Fig. S41. Long-term stability evaluation of ISA. (a) XANES spectra of ISA-after, Fe foil, Fe_2O_3 and FeO. (b) FT of the Fe K-edge of ISA-after, Fe foil, Fe_2O_3 and FeO. (c) WT-EXAFS contour plots of ISA-after. (d) Corresponding EXAFS *R* space fitting curves of ISA-after.

2. The detection of flavin adenine dinucleotide (FAD) and its correlation with enhanced current responses support a dual electron-transfer mechanism. However, the relative quantitative contribution of each pathway remains ambiguous. Can the authors provide a more clarification regarding what fraction of the total electron flux is attributable to FAD-mediated shuttling versus direct hydrogen oxidation at the ISA interface?

Response:

We thank the reviewer for this insightful comment. Following the reviewer's framework, the two electron-transfer pathways can be distinguished by their dependence on the applied electrical bias: pathway A—direct electron transfer from the electrode to the cell via extracellular FAD as a diffusible mediator, and pathway B—hydrogen-mediated transfer in which the electrode generates H_2 that is subsequently activated at the ISA interface, with FAD potentially serving as a short-range downstream carrier. Because ISA is covalently anchored to the cell membrane, the diffusion distance

for FAD between the ISA site and the intracellular electron transport chain is only a few nanometers, whereas in pathway A, FAD must diffuse across the much larger electrode-to-cell mass-transport boundary layer (typically on the order of tens of micrometers under our stirred conditions). This geometric asymmetry strongly favors pathway B kinetically.

Leveraging this distinction, we compared the growth of C.N@ISA under two H_2-supply modes: (i) *in situ* electrochemical H_2 generation, in which both pathways are operative, and (ii) direct H_2 + O_2 bubbling without applied potential, in which pathway A is effectively switched off because no electrode-derived electrons are available to FAD. The observed OD600 values increase after 168 h were 3.98 under electrochemical conditions and 2.58 under direct H_2 bubbling (Fig. S24), corresponding to approximately 2.58/3.98 × 100% ≈ 65% of the electrochemical growth being attributable to pathway B, with the remaining ~35% representing the electrochemical enhancement that can be broadly assigned to pathway A. This estimate is consistent with our overall mechanistic picture, in which ISA-mediated interfacial H_2 activation (pathway B) provides the dominant electron input while FAD-mediated direct electron transfer from electrode (pathway A) plays a secondary, cooperative role.

We emphasize, however, that this partitioning should be regarded as an order-of-magnitude estimate rather than a precise quantification, for the following reasons. First, the extracellular FAD concentration may differ between the two conditions, as electrochemical polarization may influence microbial flavin secretion; this could alter the absolute magnitude of pathway A even when it is nominally active. Second, the electrochemical system differs from the bubbling system in multiple respects beyond the electron-donor pathway, including local pH, O_2 gradients, near-electrode electric field effects, and local H_2 concentration profiles, each of which may independently influence microbial metabolism. Third, OD_{600} is a terminal phenotypic readout that integrates many metabolic processes non-linearly, and its fractional changes do not map one-to-one onto the underlying electron fluxes.

Taken together, the comparison places a useful bound on the relative contributions of the two pathways and is consistent with pathway B being the dominant electron-input route, but a precise deconvolution of the electron flux is not feasible within the scope of this study. This qualitative conclusion, namely that ISA-facilitated H_2 activation serves as the primary electron-input pathway while FAD-mediated shuttling plays a secondary role, is further supported by several independent

lines of evidence. These include the low DFT-calculated H_2 dissociation barrier (0.079 eV, Fig. 4e), D_2 incorporation into PHB (Fig. S25), and the partial restoration of growth in the hydrogenase-deficient mutant upon ISA modification (Fig. 5f). Section 2.4 has been revised accordingly to incorporate the above discussion.

3. In Fig. S28, the elevated ATP levels and proton motive force observed in the electrochemical system compared to direct H2 bubbling suggest a "metabolic rewiring" effect. Is this phenomenon primarily a consequence of the local proton gradient generated by ISA, or could the electrochemical environment itself on the microbial energy metabolism?

Response:

We thank the reviewer for this thoughtful question. Based on the available data, we agree that the metabolic enhancement observed in Fig. S28 reflects the combined contribution of the ISA-engineered interface and the electrochemical environment, with ISA providing the primary driving force and the electrochemical environment contributing additional effects.

The primary role of the ISA interface is supported by multiple independent lines of evidence. EPR measurements confirm that ISA facilitates H_2 dissociation under biologically relevant conditions (Fig. 4c), and pH-sensitive dye imaging reveals a higher local proton concentration at the surface of C.N@ISA compared with C.N (Fig. 4d), consistent with interfacial H_2 activation. Functionally, ISA modification substantially restores growth of the hydrogenase-deficient mutant under chemoautotrophic conditions (Fig. 5f), demonstrating that ISA can effectively replace native hydrogenase activity. At the transcriptional level, C.N@ISA shows significant upregulation of electron transport chain genes (*qcr*) and ATP synthase genes (*atp*) (Fig. 5g), indicating that the biohybrid cells are metabolically responsive to the enhanced reducing-equivalent input from the ISA interface. Importantly, C.N@ISA consistently outperforms C.N in both the electrochemical system and the direct H_2 bubbling system (Fig. S24), showing that the interfacial benefit of ISA does not require an electrochemical environment to manifest.

The additional contribution of the electrochemical environment is revealed by a separate comparison. Keeping the cell–catalyst configuration the same (C.N@ISA), we compared two modes of H_2 supply: *in situ* electrochemical H_2 generation versus direct H_2 bubbling (Fig. S28). Higher ATP levels and membrane potential were observed under electrochemical conditions,

indicating that the electrochemical environment provides additional metabolic benefits beyond simple H_2 delivery. Plausible contributions include: (1) a locally modulated proton electrochemical gradient in the diffusion layer near the cathode, which may reinforce the proton gradient established at the ISA interface; and (2) weakly polarizing effects of the near-electrode region on membrane-associated redox processes. We note that a mechanistic dissection of these secondary effects is beyond the scope of this study.

Taken together, the data support a picture in which the ISA-engineered interface is the primary driver of enhanced metabolism, while the electrochemical environment provides a secondary, synergistic enhancement. We have clarified this interpretation in the revised manuscript, Section 2.5, Paragraph 2.

4. The nanozyme-like ROS scavenging activity of ISA is presented as a protective mechanism. Given that C. necator is a facultative autotrophic bacterium that relies on oxidative phosphorylation for ATP generation, is there any risk that excessive ROS scavenging could inadvertently interfere with essential redox signaling or limit the availability of reactive oxygen species required for normal metabolic function?

Response:

We thank the reviewer for raising this important point. We agree that basal ROS levels serve essential physiological roles in microbial cells, functioning as redox signaling molecules that regulate gene expression, modulate respiratory activity, and maintain intracellular redox homeostasis. Accordingly, we carefully evaluated whether the nanozyme-like activity of ISA might interfere with these functions. We conclude that such interference is unlikely in our system, for the following reasons.

First, ISA is covalently anchored to the outer surface of the cell membrane via the B-PEG linker, spatially separated from the intracellular compartments where redox signaling primarily occurs, including the cytoplasm, the inner membrane, and the respiratory chain. The most reactive signaling ROS species, such as $\cdot OH$ and $O_2^{-}\cdot$, have very short half-lives and limited diffusion distances, and are therefore typically consumed at or near their site of intracellular generation rather than crossing the outer membrane to reach extracellular ISA. The scavenging activity of ISA is also quantitatively tuned: the observed H_2O_2 decomposition activity (80% removal within 30 min, Fig.

S30) is sufficient to neutralize the exogenous ROS flux generated by the electrochemical environment, but does not establish a globally ROS-depleted medium, and basal intracellular ROS signaling at micromolar steady-state levels remains kinetically insulated from this surface-localized extracellular scavenging.

More directly, functional evidence from the operating biohybrid system indicates that oxidative phosphorylation is not compromised by ISA modification. Transcriptomic analysis (Fig. 5g) shows significant upregulation of electron transport chain genes (*qcr*) and ATP synthase genes (*atp*) in *C.N*@ISA, and this gene-level response is matched at the functional level by elevated intracellular ATP content (Fig. 5b), enhanced proton motive force (Fig. 5c), and increased biomass and PHB production (Fig. 6a). If ROS-dependent redox signaling were substantially perturbed, such a coordinated enhancement of respiration, energetics, and biosynthesis would not be expected.

Taken together, these observations indicate that the nanozyme-like activity of ISA maintains electrochemically generated ROS at a biocompatible level, protecting the biohybrid from exogenous oxidative stress while preserving the intracellular redox homeostasis required for normal metabolic function. We added above discussion in Section 2.5, Paragraph 3 of the revised manuscript.

5. The covalent anchoring of ISA to surface polysaccharides places the catalyst in close proximity to the cell membrane. Beyond the established hydrogen-enrichment effect, could the physical presence of ISA alter the conformation, lateral mobility, or activity of adjacent membrane proteins? Is there any experimental evidence that direct protein–material interactions contribute to the observed metabolic enhancement?

Response:

We thank the reviewer for this insightful question. We agree that materials located at the cell interface may, in principle, influence nearby membrane proteins through steric or electrostatic effects.

Based on our current experimental evidence, ISA is preferentially anchored to surface polysaccharides via boronic acid–diol interactions, as supported by competitive binding and fluorescence staining results (Fig. 3g and S19). In addition, our functional data indicate that the observed enhancement in H_2 utilization, proton motive force, and ATP production is closely associated with the presence of membrane-anchored ISA, suggesting an interface-mediated effect.

At the same time, we acknowledge that the present study does not directly probe membrane protein conformation, mobility, or specific protein–material interactions. Therefore, whether such interactions occur, and to what extent they contribute to the observed metabolic enhancement, remain beyond the scope of the current work, providing inspiration for follow-up studies using specialized techniques for more targeted investigations.

6. The authors have revised Fig. 6b to present PHB productivity rates rather than cumulative titers, which improves the fairness of performance comparisons. However, the term "record high" still appears multiple times in the manuscript. Given the inherent variability in reactor design, operating conditions, and cultivation durations across different studies, the reviewer recommend replacing "record high" with more qualified words to avoid unnecessary overstatement.

Response:

We thank the reviewer for recognizing the improvement in Fig. 6b, and we agree that expressions such as "record high" are subjective and not appropriate given the variability across systems and conditions. Accordingly, we have removed such wording throughout the manuscript and revised the relevant statements to present the results in a more objective and balanced manner. The specific revisions are as follows:

- **Abstract**: the term "record high" has been removed.
- **Introduction (Paragraph 4)**: "record-high" has been revised to "efficient" ("…led to efficient CO_2-to-PHB conversion").
- **Discussion (Section 2.6, Paragraph 1)**: "record-high" has been removed; "far exceeding previous studies" has been revised to "higher than those reported in several previous studies".
- **Discussion (Section 2.6, Paragraph 3)**: "record-high" has been revised to "enhancing" ("…thereby enhancing biomass accumulation and PHB production…").
- **Conclusion**: "record-high" has been removed.

Response letter

Reviewer #4 (Remarks to the Author):

The authors have made substantial revisions that reasonably addressed most major concerns. Before publication, I suggest authors soften some claims on mechanisms, as they are not fully supported by experiments yet. For example, the molecular electron/proton transfer pathway from ISA to intracellular metabolism is still not directly resolved.

Response:

We thank the reviewer for the positive assessment of our revisions and for the suggestion regarding mechanistic claims. In response to the reviewer's concern that the molecular electron/proton transfer pathway from ISA to intracellular metabolism has not been directly resolved, we have revised the relevant sections and tempered the mechanistic claims throughout the manuscript. All changes have been highlighted in the revised manuscript.

I think the manuscript has improved - some issues have been partially addressed, but several key concerns remain unresolved. In particular, some of the newly added supplementary data and cited references do not fully support the authors' statements and, in certain cases, may even contradict the proposed mechanism. More importantly, there are several inconsistencies in experimental data for identical samples between the original submission and the revised manuscript. Such discrepancies substantially reduce confidence in the reliability of the reported results.

Detailed comments:

1. As clarified in the authors' response, the C.N@ISA hybrids are in a planktonic state, and no obvious attachment to the stainless-steel electrode surface was observed. Under such conditions, some experimental observations and interpretations presented by the authors become difficult to explain:
    a) If ISA is not in direct contact with the stainless-steel electrode, measuring HOR activity using ISA loaded glassy carbon electrode becomes irrelevant and cannot rigorously demonstrate that ISA promotes H_2 dissociation in the actual system. For Fe single-atom catalysts, enhanced current relative to bare glassy carbon is expected under an applied overpotential simply due to the presence of catalytic sites. However, in the freely suspended C.N@ISA hybrid, no overpotential can be applied to ISA to drive H_2 oxidation. Therefore, the HOR measurements on ISA-modified electrodes cannot directly support the claimed H_2 activation mechanism in the biohybrid system.
    b) The RDE results of bare glassy carbon electrode are inconsistent between the two versions of the manuscript. In the first round of review, the authors attributed the unusual RDE behavior to unoptimized testing conditions and inconsistent H_2 saturation. However, the experimental procedure for the bare glassy carbon electrode appears essentially unchanged. It is therefore unclear how the limiting HOR current for bare glassy carbon could change from 1.2~1.8 mA/cm^2 in the original manuscript to essentially zero with no electrochemical response in the revised version. Whether this results from inaccurate data in the initial submission or incomplete updates to the experimental method, such discrepancies significantly decrease the confidence in the reliability of the reported results.

4.7 Evaluation of hydrogen availability and oxidation kinetics. Hydrogen oxidation kinetics were studied using a rotating ring-disk electrode (RRDE)[36]. A glassy carbon disk electrode (4 mm diameter) loaded with or without ISA served as the working electrode, with stainless-steel and saturated Ag/AgCl electrodes as counter and reference electrodes, respectively. H_2-saturated PBS was used as the electrolyte. Linear

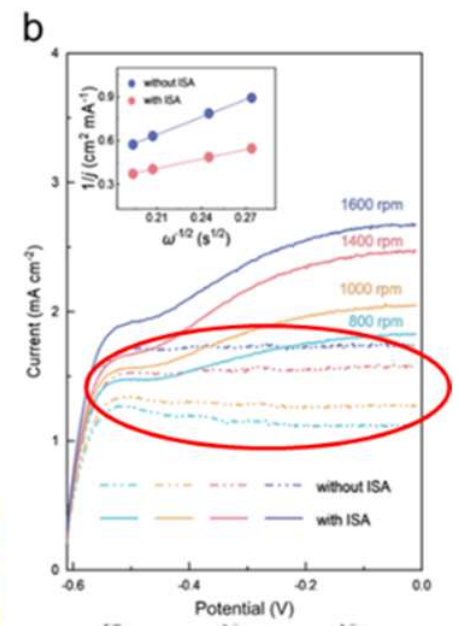

4.7 Evaluation of hydrogen availability and oxidation kinetics. Hydrogen oxidation kinetics were studied using RRDE[28]. A glassy carbon disk electrode (4 mm diameter), either unmodified or coated with ISA (dispersed by ultrasonication in isopropanol containing 0.5 wt% Nafion, with an ISA loading of 0.4 mg cm^{-2}), was used as the working electrode, with Pt wire and saturated Ag/AgCl electrodes as counter and reference electrodes, respectively. H_2-saturated PBS (0.1 M, pH = 7.0) was used as the

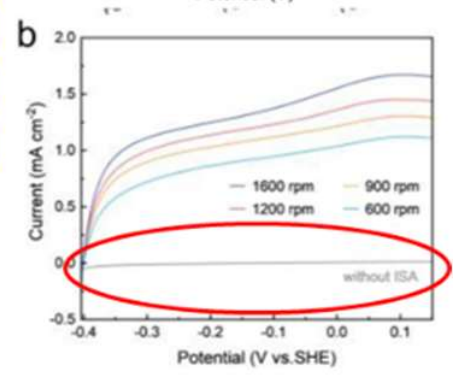

c) Even without considering the two critical issues discussed above, the updated RDE polarization curves remain questionable. The current response appears to show two quasi-plateau regions (-0.30 to -0.15 V and after ~0.10 V), while the current slightly decreases beyond ~0.10 V. This behavior resembles a broad oxidation peak rather than a well-defined diffusion-limited plateau, making it difficult to determine whether the Fe sites can be oxidized (please check the Pourbaix diagram of Fe). In the HOR field, the characteristic RDE kinetics have been well established (Energy Environ. Sci., 2024, 17, 3960-4009), yet the updated curves, even under optimized conditions with H_2-saturated electrolyte, cannot resemble those expected for a pure HOR process. This raises questions about whether the observed current is truly dominated by HOR rather than other parasitic processes.

d) The authors emphasize that the C.N@ISA hybrid remains planktonic and does not directly uptake electrons from the electrode. Under such conditions, the charge-transfer resistance (Rct) associated with HER should depend primarily on the stainless-steel electrode and the electrode-electrolyte interface. The planktonic CN, CN+ISA, and CN@ISA should not have significant electrochemical interactions with the electrode. It is therefore difficult to understand why, under the same experimental setup, the authors still report a significant decrease in Rct in the EIS results.

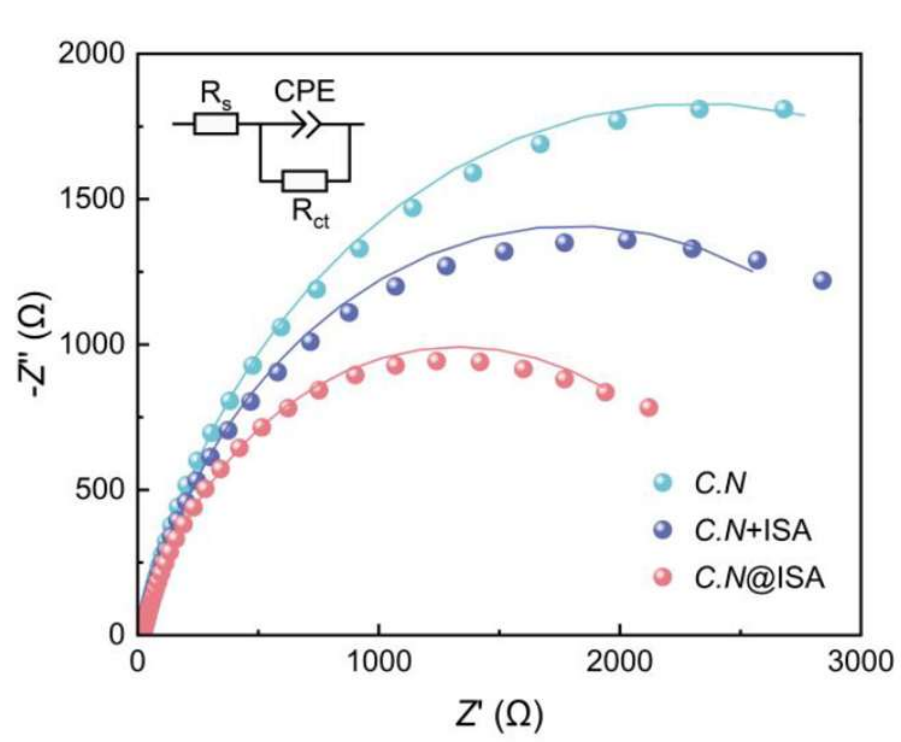

e) Moreover, the equivalent-circuit fitting for the updated EIS results does not appear well sufficiently justified. and the confidence in the fitting seems limited. The authors should carefully re-examine the fitting procedure, consider alternative models if necessary, and report fitting errors or confidence levels.

2. Similar to the baseline issue noted in Comment 1(b), the EPR results also show inconsistencies between the original and revised manuscripts. The noise strength of the DMPO-only blank control differs substantially between the two versions (see figure below). Moreover, the authors state that DMPO+H_2 is also a blank control measured without electrochemical operation, yet its baseline noise strength is markedly higher than the DMPO-only spectrum. The reason for these discrepancies is unclear.

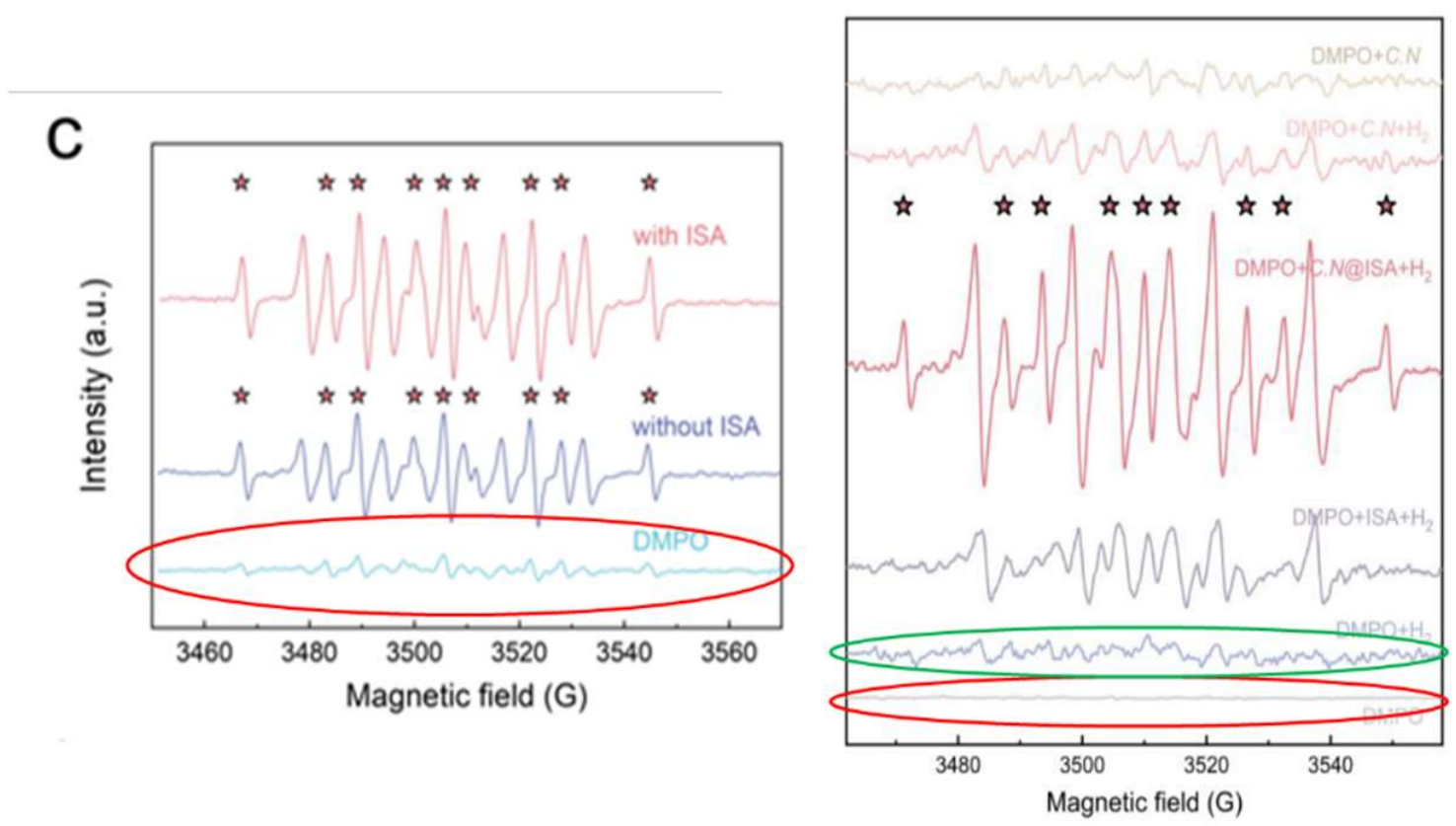

3. In the first round of review, we raised concerns regarding the thermodynamics and kinetics of spontaneous H_2 dissociation. The authors responded that the system may form a chemical pathway like a hydrogen-oxygen fuel cell when coupled with facultative microbes. While such a mechanism may be possible in the presence of living cells or extracellular redox mediators, the author's explanation will also imply that spontaneous H_2 dissociation on ISA alone should be extremely difficult in the absence of these biological components. This leads to a critical question: in the EPR experiments, how are the H radicals detected in the DMPO+ISA+H_2 group (even though the signal intensity is relatively weak)? If the H_2 dissociation only by ISA is very difficult or even infeasible, where are the H radicals from?

4. When checking the reference (*Nat Commun* **16**, 8141 (2025)) provided by the authors, the oxidation and reduction potentials of flavins are -0.1V and -0.14V vs. SHE, respectively. The authors may have to explain the redox potential shift in the updated Figure 4f.

5. Besides, for the HPLC results of FAD detection, obviously, there is a position shift of the claimed FAD peak, we suggest that the authors should be very careful about the attribution of this peak. Maybe NMR could be helpful, but not necessary.

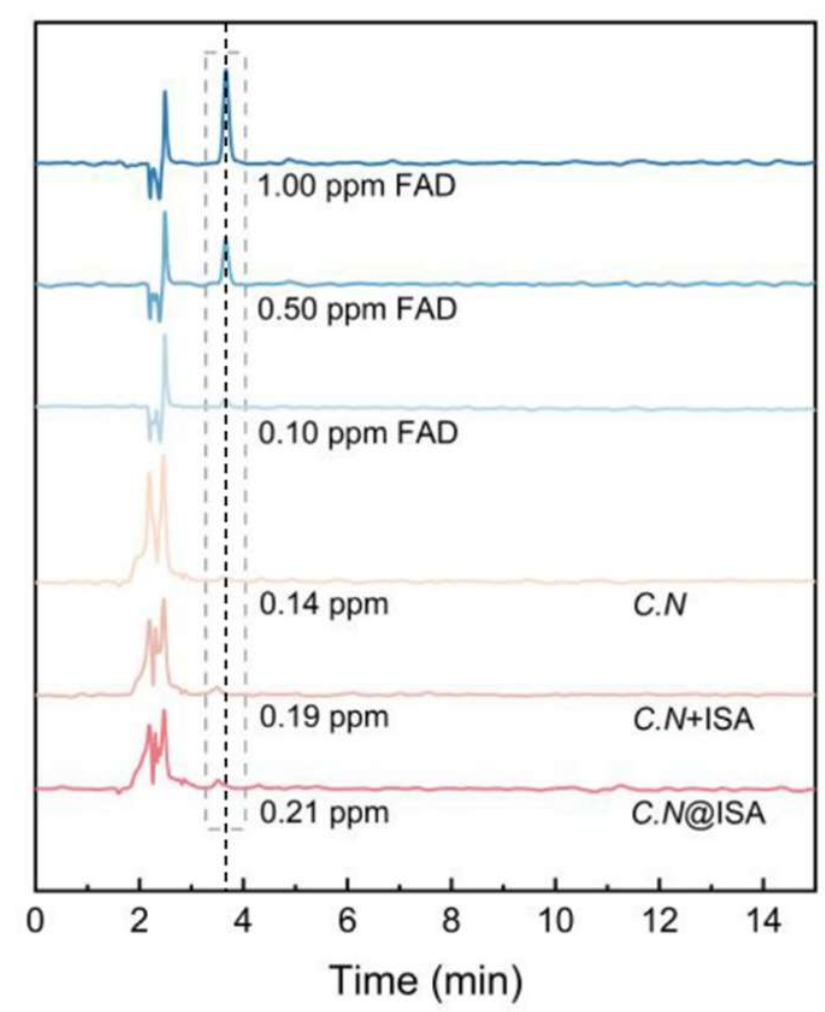

1.00 ppm FAD
0.50 ppm FAD
0.10 ppm FAD
0.14 ppm
C.N
0.19 ppm
C.N+ISA
0.21 ppm
C.N@ISA
0
2
4
6
8
10
12
14
Time (min)
